# Supplementary figures and images for: Proteomic profile of human colon organoids: effects of a multi-mineral intervention alone and in the presence of pro-inflammatory and anti-inflammatory treatments
Source: Front Gastroenterol (Lausanne). 2025 Jul 2;4:1592669. doi: 10.3389/fgstr.2025.1592669 (PMC12952359; doi:10.3389/fgstr.2025.1592669)

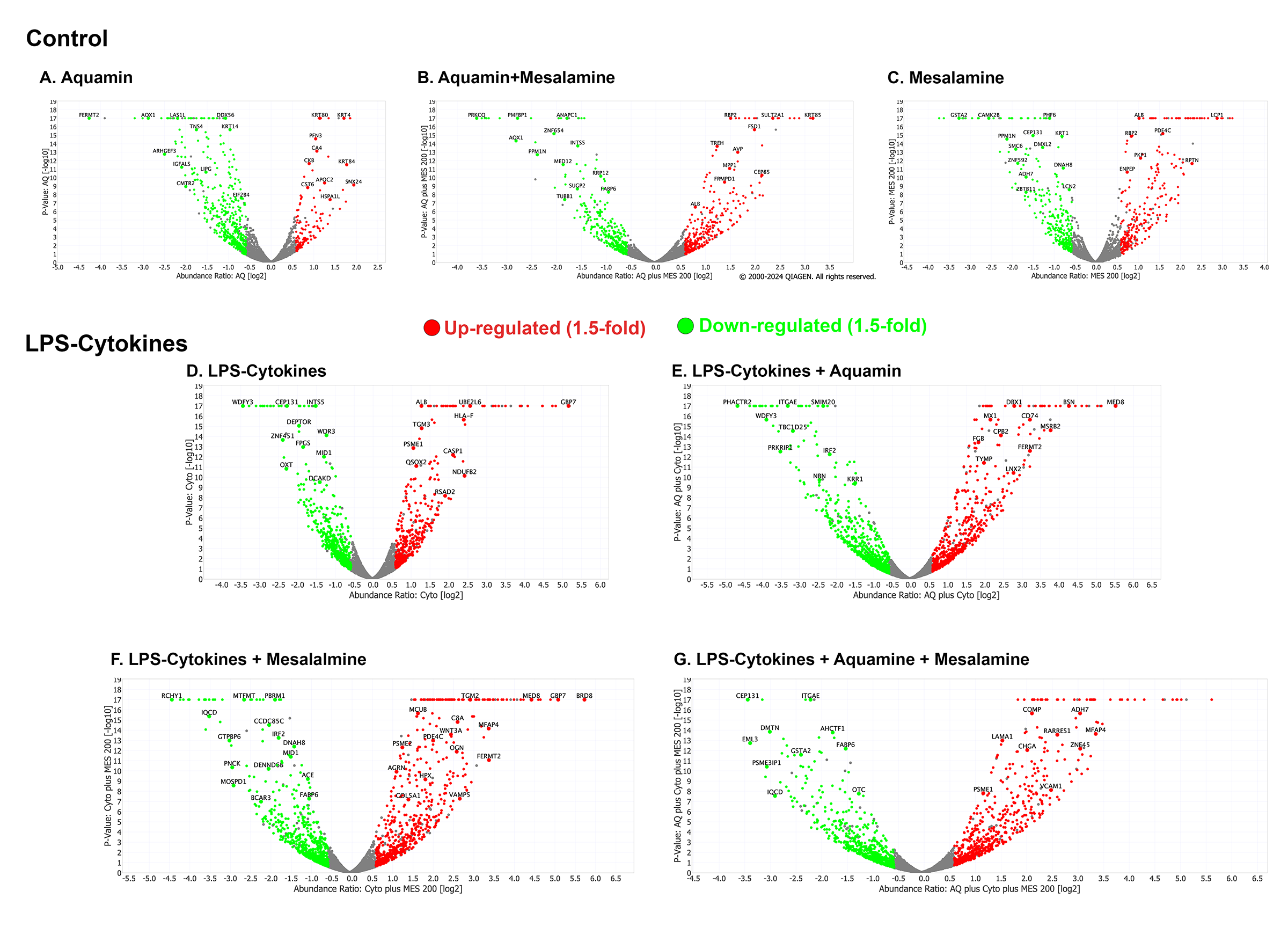

Supplement: Supplementary Figure S1 — Assessment of colon organoid appearance in response to varying doses of Mesalamine using phase-contrast microscopy. At the end of the incubation period, intact colon organoids were examined with phase-contrast microscopy to identify differences across a Mesalamine dose range of 50-250 µg. Under all conditions, a diverse range of sizes and shapes was observed. Scale bar = 500 µm. [file DataSheet2.zip › Image S3.tif]

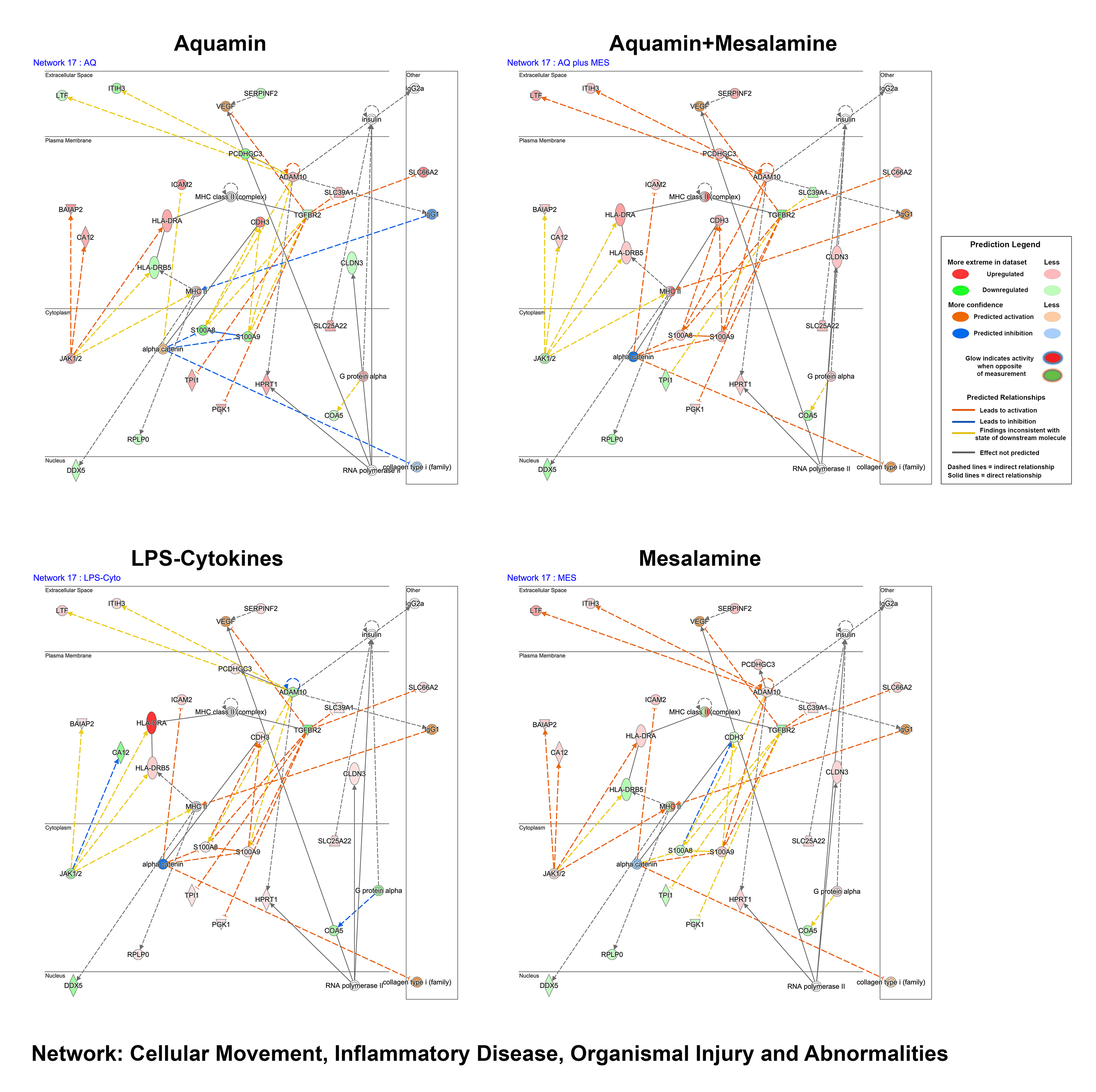

Supplement: Supplementary Figure S1 — Assessment of colon organoid appearance in response to varying doses of Mesalamine using phase-contrast microscopy. At the end of the incubation period, intact colon organoids were examined with phase-contrast microscopy to identify differences across a Mesalamine dose range of 50-250 µg. Under all conditions, a diverse range of sizes and shapes was observed. Scale bar = 500 µm. [file DataSheet2.zip › Image S4.tif]
