# Supplementary material for: Proteomic profile of human colon organoids: effects of a multi-mineral intervention alone and in the presence of pro-inflammatory and anti-inflammatory treatments
Source: Front Gastroenterol (Lausanne). 2025 Jul 2;4:1592669. doi: 10.3389/fgstr.2025.1592669 (PMC12952359; doi:10.3389/fgstr.2025.1592669)
Supplement: Supplementary Table S1 — Mineral composition of Aquamin. [file DataSheet1.zip › Table S1.pdf]

Supplement Table 1. A list of minerals and elements identified in Aquamin® Soluble

Element	μg/g	Element	μg/g	Element	μg/g
Aluminum	21.6	Hafnium	0.038	Rubidium	0.031
Antimony	0.69	Holmium	0.010	Ruthenium	0.137
Arsenic	0.239	Indium	<0.001	Samarium	0.037
Barium	1.76	Iodine	1.81	Scandium	0.469
Beryllium	<0.5	Iridium	<0.001	Selenium	<0.5
Bismuth	<0.5	Iron	143	Silicon	16.8
Boron	13.7	Lanthanum	<0.5	Silver	<0.5
Cadmium	0.220	Lead	0.084	Sodium	2,206
Calcium	117,000	Lithium	<0.5	Strontium	882
Carbon	26,600	Lutetium	<0.001	Sulfur	1,241
Cerium	0.314	Magnesium	10,210	Tantalum	0.043
Cesium	0.001	Manganese	25.4	Tellurium	<0.5
Chloride	612	Mercury	<0.001	Terbium	0.007
Chromium	<0.5	Molybdenum	<0.5	Thallium	<0.5
Cobalt	<0.5	Neodymium	0.170	Thorium	1.30
Copper	<0.5	Nickel	0.75	Thulium	0.004
Dysprosium	0.045	Niobium	<0.5	Tin	0.029
Erbium	0.033	Osmium	<0.001	Titanium	11.4
Europium	0.013	Palladium	0.179	Tungsten	<0.5
Fluoride	3.57	Phosphorous	189	Vanadium	<0.5
Gadolinium	0.044	Platinum	<0.001	Ytterbium	0.030
Gallium	0.307	Potassium	70.0	Yttrium	<0.5
Germanium	<0.001	Praseodymium	0.040	Zinc	6.07
Gold	<0.5	Rhenium	0.001	Zirconium	<0.5
		Rhodium	0.061		

The mineral composition of Aquamin® Soluble was analyzed by Advanced Laboratories, Inc. in Salt Lake City, on behalf of Marigot Limited, Ireland, as detailed in the 2017 Test Certificate for Aquamin® Soluble. The concentrations of individual minerals and trace elements were primarily determined using Inductively Coupled Plasma Optical Emission Spectrometry (ICP-OES), with the exception of carbon, which was measured using LECO analysis; chloride and iodine, which were assessed via titration; and fluoride, evaluated according to AOAC 939.11
