# Supplementary material for: Proteomic profile of human colon organoids: effects of a multi-mineral intervention alone and in the presence of pro-inflammatory and anti-inflammatory treatments
Source: Front Gastroenterol (Lausanne). 2025 Jul 2;4:1592669. doi: 10.3389/fgstr.2025.1592669 (PMC12952359; doi:10.3389/fgstr.2025.1592669)
Supplement: Supplementary Table S1 — Mineral composition of Aquamin. [file DataSheet1.zip › Table S2.pdf]

Supplement Table 2. Demographic characteristics of tissue donors (subjects)

Sample ID	Age (Y)	Sex	Ethnicity	Biopsy Site
Colon-E1*	49	F	White (Not Hispanic)	Ascending colon
Colon-E7*	21	M	White (Not Hispanic)	Ascending colon
Colon-E8*	33	F	White (Not Hispanic)	Ascending colon
Colon-O5	62	M	White (Not Hispanic)	Sigmoid colon

*Source: Gift of Life, Michigan
