# Supplementary material for: Proteomic profile of human colon organoids: effects of a multi-mineral intervention alone and in the presence of pro-inflammatory and anti-inflammatory treatments
Source: Front Gastroenterol (Lausanne). 2025 Jul 2;4:1592669. doi: 10.3389/fgstr.2025.1592669 (PMC12952359; doi:10.3389/fgstr.2025.1592669)
Supplement: Supplementary Table S1 — Mineral composition of Aquamin. [file DataSheet1.zip › Table S3.pdf]

Supplement Table 3. Up-regulated proteins influenced by Aquamin and Mesalamine under control conditions (with 1.5-fold).

A. Common among three groups – Aquamin (AQ), Mesalamine (MES) and Aquamin plus Mesalamine (AQ+MES) [41 proteins]

		Interventions						
		Control			*With LPS & Cytokines*			
Proteins	Genes	**AQ**	**AQ+MES**	**MES**	*LPS-Cyto*	*AQ*	*AQ+MES*	*MES*
Sorting nexin-24	SNX24	3.83*	4.44*	3.16*	5.18*	5.74*	3.20*	4.30*
Putative beta-actin-like protein 3	POTEKP	3.53*	4.54*	4.91*	1.20	1.16	1.95*	5.20*
FXYD domain-containing ion transport regulator 5	FXYD5	3.37*	3.09*	2.13*	2.24*	5.59*	4.94*	4.29*
Keratin, type II cytoskeletal 4	KRT4	3.27*	1.56*	9.03*	0.70	10.27*	1.13	6.70*
Transmembrane and immunoglobulin domain-containing protein 1	TMIGD1	2.53*	7.38*	3.86*	3.17*	3.35*	4.41*	2.89*
Equilibrative nucleoside transporter 1	SLC29A1	2.51*	2.80*	2.70*	4.14*	4.67*	4.44*	3.78*
Solute carrier family 35 member B1	SLC35B1	2.33*	2.96*	3.17*	1.47	2.58*	2.91*	2.01*
Keratin, type I cytoskeletal 23	KRT23	2.31*	1.81*	6.31*	0.44*	2.65*	0.70	2.04*
Triggering receptor expressed on myeloid cells 1	TREM1	2.25*	2.93*	1.88*	1.88*	4.12*	1.94*	3.89*
Myeloid leukemia factor 2	MLF2	2.19*	1.97*	3.37*	2.90*	2.56*	1.98*	2.36*
Small nuclear ribonucleoprotein F	SNRPF	2.07*	1.62*	1.69*	1.59*	0.18*	0.89	0.54
NADH dehydrogenase [ubiquinone] 1 beta subcomplex subunit 2, mitochondrial	NDUFB2	2.06*	1.92*	3.85*	5.33*	9.24*	8.41*	5.38*
Spliceosome-associated protein CWC27 homolog	CWC27	2.02*	1.81*	1.94*	1.33	0.51	0.99	2.11*
Nuclear receptor coactivator 6	NCOA6	2.02*	3.40*	3.87*	2.35*	0.89	2.98*	4.19*
Xaa-Pro aminopeptidase 2	XPNPEP2	2.00*	2.97*	1.79*	1.17	1.85*	1.67*	1.38
Complement component C8 gamma chain	C8G	1.96*	2.13*	4.51*	1.81*	2.79*	1.96*	1.26
Transcription factor ETV6	ETV6	1.90*	1.88*	2.40*	1.54*	1.45	2.02*	1.81
Prostatic acid phosphatase	ACP3	1.83*	8.61*	2.97*	1.52*	2.09*	1.08	1.62
Mitoferrin-2	SLC25A28	1.83*	3.25*	3.19*	1.78*	1.29	3.96*	2.50*
Cystatin-M	CST6	1.81*	3.67*	3.47*	0.54*	1.96*	0.58	1.84*
Transmembrane protein 236	TMEM236	1.79*	4.00*	2.65*	1.34	1.43	2.59*	1.66
RING1 and YY1-binding protein	RYBP	1.77*	1.81*	2.11*	1.28	1.80	0.92	2.13*
Dynein axonemal heavy chain 17	DNAH17	1.74*	2.19*	1.86*	0.67	0.06*	0.20*	0.23*
Semenogelin-1	SEMG1	1.74*	4.10*	2.91*	0.71	2.56*	0.55*	2.67*
F-box only protein 50	NCCRP1	1.73*	2.05*	3.97*	0.77	1.78*	0.88	1.38
Cornulin	CRNN	1.73*	2.38*	5.70*	0.44*	3.63*	0.61	2.57*
Desmocollin-3	DSC3	1.72*	9.40*	34.18*	0.65	1.84*	0.93	0.01*
Bleomycin hydrolase	BLMH	1.70*	1.51*	1.70*	1.98*	1.46	1.45	1.59
Nuclear speckle splicing regulatory protein 1	NSRP1	1.69*	2.06*	2.33*	1.09	0.02*	0.40*	1.14

Cilia- and flagella-associated protein 45	CFAP45	1.69*	1.98*	2.43*	1.19	3.11*	2.25*	2.51*
Acetylcholinesterase	ACHE	1.68*	3.16*	2.26*	1.09	1.69	2.30*	1.62
Solute carrier family 40 member 1	SLC40A1	1.66*	1.70*	1.81*	3.07*	4.44*	3.81*	3.78*
Protein C19orf12	C19orf12	1.61*	2.28*	1.77*	1.55	1.49	1.94*	1.86
Histidine ammonia-lyase	HAL	1.60*	2.11*	5.94*	0.35*	2.50*	0.56	2.12*
Retinoic acid receptor responder protein 1	RARRES1	1.60*	2.72*	3.25*	3.23*	3.43*	6.05*	6.66*
Trehalase	TREH	1.60*	2.35*	1.52*	0.90	1.42*	1.88*	1.13
HLA class II histocompatibility antigen, DM beta chain	HLA-DMB	1.59*	13.66*	8.99*	1.13	1.05	10.01*	8.97*
Small integral membrane protein 20	SMIM20	1.53*	1.65*	1.62*	1.27*	0.19*	0.38*	0.30*
NK-tumor recognition protein	NKTR	1.53*	1.80*	1.57	1.59	1.50	1.67	1.19
Alsin	ALS2	1.51*	1.57	1.67*	1.30	1.37	1.56	1.50
Ubiquinol-cytochrome-c reductase complex assembly factor 6	UQCC6	1.50*	1.63*	1.62*	1.17	0.37*	0.43*	0.39*

B. Up-regulated proteins unique to Aquamin (AQ) [57 proteins]

		Interventions						
		Control			*With LPS & Cytokines*			
Proteins	Genes	**AQ**	AQ+MES	MES	*LPS-Cyto*	*AQ*	*AQ+MES*	*MES*
Keratin, type II cuticular Hb4	KRT84	3.41*	1.07	1.03	1.40	0.95	1.04	3.69*
Mediator of RNA polymerase II transcription subunit 8	MED8	3.06*	0.51*	0.49*	22.20*	45.95*	32.32*	21.41*
Putative histone H2B type 2-C	H2BC20P	2.73*	0.76	0.85	10.59*	34.44*	25.80*	21.48*
Heat shock 70 kDa protein 1-like	HSPA1L	2.61*	1.48	1.29	8.97*	15.03*	9.11*	10.55*
Apolipoprotein C-II	APOC2	2.38*	0.68	0.99	9.94*	21.64*	14.48*	17.84*
Deleted in malignant brain tumors 1 protein	DMBT1	2.24*	0.89	1.16*	1.67*	1.57*	0.60*	0.73*
Stanniocalcin-2	STC2	2.21*	1.18	1.08	1.05	0.95	1.24	0.87
Keratin, type II cytoskeletal 80	KRT80	2.20*	1.11	1.12	0.99	1.72*	0.99	1.07
Cytochrome P450 2C19	CYP2C19	2.12*	1.15	0.61*	1.64*	1.98*	0.88	0.62
Carbonic anhydrase 4	CA4	2.10*	1.45*	1.09	1.18	1.36	0.85	0.69
Profilin-3	PFN3	2.06*	0.44*	0.48*	0.82	0.81	0.84	0.37*
Protein disulfide-isomerase A2	PDIA2	2.04*	1.32	1.24	1.68*	0.72	0.87	1.13
Nucleoplasmin-3	NPM3	1.99*	1.31	1.49	2.28*	0.37*	0.65	0.30*
Group IID secretory phospholipase A2	PLA2G2D	1.94*	0.98	0.85	3.07*	7.57*	9.66*	8.40*
Guanylate-binding protein 4	GBP4	1.91*	0.78	0.94	22.11*	26.71*	25.20*	24.08*
2-aminomuconic semialdehyde dehydrogenase	ALDH8A1	1.90*	1.21	1.32	2.50*	13.33*	30.36*	5.64*
Glycerophosphoinositol inositolphosphodiesterase GDPD2	GDPD2	1.86*	1.32	0.87	0.91	1.44	1.40	0.80
Creatine kinase B-type	CKB	1.86*	1.48*	1.47*	0.69*	0.99	1.05	0.58*
UDP-glucuronosyltransferase 2A3	UGT2A3	1.71*	1.27	0.93	0.94	1.03	0.73	0.51*

Pterin-4-alpha-carbinolamine dehydratase 2	PCBD2	1.71*	1.26	1.07	1.40	1.81*	1.88*	1.22
Bromodomain-containing protein 8	BRD8	1.65*	0.96	0.74	26.61*	35.18*	48.74*	52.93*
Hephaestin	HEPH	1.64*	1.49*	1.05	0.94	1.50*	1.23	0.72*
Neuropilin-2	NRP2	1.63*	1.43	1.45*	1.26	1.69*	1.56	1.33
Olfactomedin-4	OLFM4	1.62*	0.97	0.77*	0.88	1.06	0.78	0.91
Protein PET100 homolog, mitochondrial	PET100	1.62*	1.23	0.99	1.10	1.09	1.11	0.97
Major facilitator superfamily domain-containing protein 8	MFSD8	1.61*	1.27	1.09	2.56*	4.01*	3.96*	3.35*
Transmembrane 4 L6 family member 20	TM4SF20	1.61*	0.93	0.65*	0.63*	1.51*	0.97	0.50*
Aminopeptidase N	ANPEP	1.60*	1.32*	0.97	0.91	0.80	0.95	0.67*
Amiloride-sensitive sodium channel subunit alpha	SCNN1A	1.60*	1.38	0.85	0.99	0.86	1.44	0.65
V-type immunoglobulin domain-containing suppressor of T-cell activation	VSIR	1.60*	1.43	1.49*	2.30*	5.02*	3.78*	3.72*
C-C motif chemokine 15	CCL15	1.60*	1.01	0.78	0.96	0.70	0.82	0.67
Very low-density lipoprotein receptor	VLDLR	1.58*	1.36	1.44*	1.24	1.93*	1.56	1.43
Nuclease EXOG, mitochondrial	EXOG	1.57*	1.36	1.45	2.19*	3.66*	3.66*	3.13*
IgG receptor FcRn large subunit p51	FCGRT	1.56*	1.42	0.97	1.38	1.32	1.30	1.05
Hexokinase HKDC1	HKDC1	1.56*	1.03	0.87	1.07	1.23	0.93	0.82
Exosome complex component RRP42	EXOSC7	1.55*	1.10	1.10	1.22	1.16	1.15	1.05
Matrix-remodeling-associated protein 7	MXRA7	1.55*	1.50	1.19	1.41	1.04	1.02	0.86
Solute carrier family 66 member 2	SLC66A2	1.55*	1.44*	1.44*	1.99*	4.16*	3.88*	3.56*
Epsin-2	EPN2	1.54*	1.34*	1.44*	1.52*	1.37	1.38	1.54*
Sulfiredoxin-1	SRXN1	1.54*	1.21	1.01	1.11	1.42	1.20	1.33
A-kinase anchor protein 9	AKAP9	1.54*	0.94	0.85	1.12	1.04	0.78	0.70
Transmembrane protein 164	TMEM164	1.54*	1.23	0.99	0.79	1.15	1.18	0.69
Telomeric repeat-binding factor 2	TERF2	1.54*	1.23	1.29	1.43	0.60	0.56	0.66
Keratinocyte-associated protein 2	KRTCAP2	1.54*	1.29	1.18	2.55*	4.86*	4.63*	2.87*
PWWP domain-containing DNA repair factor 4	PWWP4	1.54*	1.13	1.24	0.54*	1.27	1.13	0.67
Trefoil factor 1	TFF1	1.54*	1.08	0.91	0.61*	0.63*	0.48*	0.37*
Transmembrane protein 125	TMEM125	1.53*	1.26	0.95	1.26	1.86*	0.98	1.15
Complement C1r subcomponent-like protein	C1RL	1.53*	1.26	1.35	1.05	1.08	0.47*	0.76
Inactive tyrosine-protein kinase transmembrane receptor ROR1	ROR1	1.53*	0.91	1.21	1.01	1.12	0.82	1.05
Matrilysin	MMP7	1.52*	1.02	1.05	1.66*	1.74*	1.24	1.42*
Twisted gastrulation protein homolog 1	TWSG1	1.51*	1.20	1.09	1.12	0.77	0.85	1.00
PC4 and SFRS1-interacting protein	PSIP1	1.51*	1.06	1.31*	1.61*	0.48*	0.47*	1.46*
Trinucleotide repeat-containing gene 6B protein	TNRC6B	1.51*	1.07	0.90	0.59*	0.65	1.27	0.62
DnaJ homolog subfamily B member 6	DNAJB6	1.51*	1.38	1.48	0.98	1.06	1.27	0.98
PAT complex subunit Asterix	WDR83OS	1.51*	1.46	1.31	1.84*	5.64*	4.74*	4.39*

Condensin complex subunit 2	NCAPH	1.51*	1.22	1.07	1.36	2.49*	2.98*	1.72*
Metalloproteinase inhibitor 2	TIMP2	1.50*	1.16	1.12	1.27	1.40	1.26	1.02

C. Up-regulated proteins unique to Aquamin plus Mesalamine (AQ+MES) [113 proteins]

		Interventions						
		Control			*With LPS & Cytokines*			
Proteins	Genes	AQ	**AQ+MES**	MES	*LPS-Cyto*	*AQ*	*AQ+MES*	*MES*
Lysozyme g-like protein 2	LYG2	0.84	31.25*	1.48	0.85	1.13	1.33	1.98*
Keratin, type II cuticular Hb2	KRT82	0.91	23.09*	1.49*	0.74	1.00	0.40*	1.04
Keratin, type II cuticular Hb5	KRT85	0.66*	8.93*	1.06	0.69*	0.55*	0.40*	1.22
Maltase-glucoamylase	MGAM	1.32*	3.05*	1.31*	1.32*	1.62*	1.41*	0.94
55 kDa erythrocyte membrane protein	MPP1	1.16	2.80*	1.47*	1.10	1.36	1.92*	1.21
Dynein regulatory complex protein 10	IQCD	0.69	2.52*	1.32	0.48*	0.17*	0.13*	0.09*
Aspartate dehydrogenase domain-containing protein	ASPDH	1.49*	2.23*	1.22	1.34	1.82*	1.07	1.07
Sorbin and SH3 domain-containing protein 1	SORBS1	0.97	2.18*	1.43	1.40	1.86*	1.35	1.24
Apolipoprotein D	APOD	1.10	2.13*	1.32	4.26*	2.98*	1.00	2.56*
5'-3' exonuclease PLD3	PLD3	1.31*	2.06*	1.16	1.16	1.31	1.17	1.12
Seipin	BSCL2	1.44*	2.03*	1.20	0.85	1.45	1.27	1.26
Guanylate-binding protein 7	GBP7	1.02	2.02*	0.88	35.72*	32.72*	30.59*	33.79*
Olfactory receptor 1M1	OR1M1	1.48*	1.95*	1.32	1.06	1.87*	2.10*	1.19
Protein ABHD13	ABHD13	0.71	1.91*	1.42	0.70	0.92	1.38	1.31
Laminin subunit beta-2	LAMB2	0.67*	1.91*	1.30*	1.38*	1.83*	2.20*	3.05*
Zymogen granule membrane protein 16	ZG16	1.23*	1.91*	1.33*	1.18	1.16	0.87	1.20
Adhesion G-protein coupled receptor F1	ADGRF1	1.32*	1.87*	1.24	1.65*	1.98*	1.80*	1.81*
Melanotransferrin	MELTF	1.46*	1.86*	1.26	1.24	1.78	1.93*	1.35
Glutaminyl-peptide cyclotransferase-like protein	QPCTL	1.26	1.86*	1.14	1.24	1.57	1.58	1.49
Sodium-dependent phosphate transporter 1	SLC20A1	1.47*	1.85*	1.42*	0.90	0.54	0.70	0.57
Neutrophil elastase	ELANE	0.97	1.85*	0.83	1.30	3.28*	0.92	2.27*
Serpin B7	SERPINB7	1.00	1.84*	1.21	1.19	2.08*	2.49*	1.71*
Group 10 secretory phospholipase A2	PLA2G10	1.13	1.83*	1.30	0.87	0.14*	0.82	0.58
Mitochondrial pyruvate carrier 1	MPC1	0.99	1.83*	1.33	1.58	2.75*	2.41*	2.70*
Glutathione S-transferase Mu 4	GSTM4	1.18	1.83*	0.87	0.96	0.87	1.26	0.72
DnaJ homolog subfamily B member 9	DNAJB9	1.26	1.81*	1.41	1.32	1.26	1.76	1.72
Collagen alpha-1(XV) chain	COL15A1	0.83	1.81*	1.43	1.57	1.31	1.62	2.31*
UDP-GlcNAc:betaGal beta-1,3-N-acetylglucosaminyltransferase 7	B3GNT7	1.09	1.78*	1.50*	0.94	1.56	1.64*	1.41
Enhancer of filamentation 1	NEDD9	1.46*	1.77*	1.30	0.93	1.23	1.75*	0.91
Alpha-1-antitrypsin	SERPINA1	1.26*	1.76*	1.41*	2.93*	2.81*	2.44*	2.34*

HLA class II histocompatibility antigen, DR alpha chain	HLA-DRA	1.05	1.75*	1.05	28.26*	24.40*	28.76*	24.96*
1-phosphatidylinositol 4,5-bisphosphate phosphodiesterase delta-1	PLCD1	1.04	1.75*	1.10	0.94	0.93	0.91	0.88
Tigger transposable element-derived protein 3	TIGD3	0.93	1.74*	0.94	1.15	1.30	1.69	1.19
Deoxyhypusine synthase	DHPS	0.95	1.73*	1.01	1.75*	1.58*	1.88*	1.77*
Arylsulfatase D	ARSD	0.83	1.73*	1.02	0.94	0.75	1.04	0.99
Small integral membrane protein 24	SMIM24	1.45	1.71*	0.90	1.51	1.28	1.22	0.90
Sodium-dependent lysophosphatidylcholine symporter 1	MFSD2A	1.38	1.71*	1.13	1.34	0.87	1.25	1.57
Electron transfer flavoprotein regulatory factor 1	ETFRF1	1.41*	1.71*	1.48*	1.68*	1.53	1.47	1.73*
Bromodomain-containing protein 9	BRD9	0.70*	1.71*	1.15	1.38*	2.07*	3.25*	5.06*
Cytochrome P450 4F11	CYP4F11	0.97	1.71*	1.13	1.31	1.55	1.55	1.32
Threonylcarbamoyl-AMP synthase	YRDC	1.20	1.69*	1.25	1.89*	0.94	1.91*	1.10
Superoxide dismutase [Mn], mitochondrial	SOD2	1.20*	1.69*	1.12	1.74*	1.96*	2.13*	2.03*
2-acylglycerol O-acyltransferase 2	MOGAT2	1.18	1.68*	1.15	1.14	1.23	1.38	0.98
Cytokine receptor common subunit gamma	IL2RG	1.27	1.67*	1.27	1.28	1.27	1.55	0.97
Protein MMP24OS	MMP24OS	1.36	1.67*	1.29	1.11	1.61	1.43	1.33
Disco-interacting protein 2 homolog A	DIP2A	1.26	1.67*	1.47*	1.17	1.38	1.38	1.00
PWWP domain-containing protein 2A	PWWP2A	1.12	1.65*	1.22	1.07	0.59	0.61	0.74
Galactosylgalactosylxylosylprotein 3-beta-glucuronosyltransferase 3	B3GAT3	1.46*	1.65*	1.29	1.47*	1.77	2.12*	1.07
E3 ubiquitin ligase RNF121	RNF121	1.22	1.65*	1.28	1.18	1.43	1.63	1.17
Amino acid transporter heavy chain SLC3A1	SLC3A1	1.30	1.64*	1.12	1.08	1.43	1.21	0.94
Protein GUCD1	GUCD1	1.02	1.64*	1.44	0.74	1.48	1.65	1.75
Motile sperm domain-containing protein 1	MOSPD1	1.07	1.64*	1.30	0.65	0.27*	0.58	0.13*
UMP-CMP kinase 2, mitochondrial	CMPK2	0.96	1.64*	1.39	1.53	1.76*	2.93*	2.32*
Putative HLA class I histocompatibility antigen, alpha chain H	HLA-H	1.19	1.64*	1.17	2.96*	2.63*	2.82*	2.42*
Transcription elongation factor SPT4	SUPT4H1	1.14	1.63*	0.98	1.44	1.15	1.33	1.64
Ras-related protein Rab-30	RAB30	1.13	1.62*	1.22	1.04	1.12	1.31	0.98
Voltage-gated potassium channel subunit beta-2	KCNAB2	1.44*	1.62*	1.25	1.16	1.22	0.94	0.98
Arylacetamide deacetylase	AADAC	1.04	1.62	1.20	1.20	1.10	1.11	1.66
ATP-binding cassette sub-family D member 1	ABCD1	1.20	1.61*	1.10	0.92	1.09	1.12	0.80
Signal peptide peptidase-like 2B	SPPL2B	1.26	1.61*	1.48*	1.77*	2.29*	2.26*	2.37*
Immunoglobulin superfamily member 8	IGSF8	1.11	1.61*	1.44*	1.53*	2.10*	1.93*	1.61*
TLC domain-containing protein 4	TLCD4	1.45*	1.60*	1.10	1.12	1.45	1.40	1.02
Tissue factor	F3	0.97	1.60*	1.48*	1.34*	1.23	1.69*	1.58*
Intestinal-type alkaline phosphatase	ALPI	1.25	1.60*	1.15	1.01	1.45	1.54	1.09
Phosphatidylinositol 4-kinase type 2-beta	PI4K2B	1.29	1.60*	1.40	1.23	1.27	1.36	1.01
Tetraspanin-14	TSPAN14	1.01	1.60*	0.95	1.09	1.38*	1.29	1.22

Mitochondrial import receptor subunit TOM40B	TOMM40L	1.43*	1.59*	1.46*	1.94*	2.74*	2.90*	2.29*
Prothrombin	F2	0.84	1.59	1.40	1.51*	2.10*	3.12*	3.81*
Dixin	DIXDC1	1.10	1.59*	1.27	1.43	0.87	1.95*	1.47
Protein FAM83G	FAM83G	1.12	1.58*	1.13	0.85	0.46*	0.56	0.54
Bis(5'-adenosyl)-triphosphatase	FHIT	1.09	1.58*	1.32	1.78*	1.32	1.52	1.77*
HIG1 domain family member 1A, mitochondrial	HIGD1A	1.46	1.58*	1.10	1.43	1.83*	2.31*	1.94*
Pannexin-1	PANX1	0.84	1.58	0.82	1.18	1.37	1.21	1.26
Ubiquinol-cytochrome-c reductase complex assembly factor 3	UQCC3	1.21	1.58	1.28	0.89	0.90	0.92	0.88
Lysosomal thioesterase PPT2	PPT2	0.78	1.58*	0.68	1.00	0.92	0.56	0.82
Proton-coupled folate transporter	SLC46A1	1.06	1.56	1.28	1.04	0.94	0.91	0.83
Phosphatidylethanolamine N-methyltransferase	PEMT	1.16	1.56	1.27	1.00	1.29	1.88*	1.29
STARD3 N-terminal-like protein	STARD3NL	1.12	1.56*	1.24	1.68*	1.74	1.55	1.57
Metallothionein-1E	MT1E	0.72	1.56*	1.26	1.03	0.83	1.26	1.43
Dual oxidase 2	DUOX2	1.39*	1.56*	1.15*	0.94	1.23	1.30	0.88
Collagen alpha-1(VI) chain	COL6A1	0.88	1.56*	1.49*	1.06	1.51*	2.05*	2.64*
Ubiquitin/ISG15-conjugating enzyme E2 L6	UBE2L6	1.34	1.56*	1.29	5.90*	5.70*	6.47*	6.58*
Sacsin	SACS	1.37*	1.56*	1.45*	1.67*	2.82*	2.91*	2.32*
Nidogen-1	NID1	0.75*	1.56*	1.44*	1.41*	1.86*	2.21*	2.89*
PDZK1-interacting protein 1	PDZK1IP1	1.21	1.54*	1.40*	1.11	1.42	1.37	1.40
P2X purinoceptor 4	P2RX4	1.27*	1.54*	1.15	0.99	1.36	1.25	0.92
Neurotensin/neuromedin N	NTS	0.74	1.54*	1.30	1.01	0.50*	1.03	0.72
Solute carrier family 52, riboflavin transporter, member 3	SLC52A3	1.21	1.54	1.45	1.29	1.72	1.52	1.49
b(0,+)-type amino acid transporter 1	SLC7A9	1.23	1.53*	1.26	1.20	1.33	1.37	0.91
Programmed cell death 1 ligand 1	CD274	0.43*	1.53	1.44	16.94*	16.35*	19.58*	15.89*
Protein O-linked-mannose beta-1,4-N-acetylglucosaminyltransferase 2	POMGNT2	1.04	1.53	1.41	1.30	1.55	1.88	1.04
Collagen alpha-1(I) chain	COL1A1	0.89	1.53*	1.40*	1.45*	1.99*	2.46*	3.30*
Mitochondrial adenyl nucleotide antiporter SLC25A25	SLC25A25	1.30	1.53	1.24	1.58*	2.23*	2.58*	1.80*
Acyl-coenzyme A synthetase ACSM3, mitochondrial	ACSM3	1.15	1.53	1.29	1.43	1.41	1.74*	1.81*
Sorcin	SRI	1.45*	1.52*	1.05	0.89	1.13	1.04	0.79
Elongation factor 1-alpha 2	EEF1A2	0.84	1.52*	1.19	1.02	1.66*	1.71*	1.43
Glutaminyl-peptide cyclotransferase	QPCT	1.49*	1.52*	1.48*	0.83	1.22	0.98	1.26
Organic solute transporter subunit beta	SLC51B	1.09	1.52*	1.30*	1.00	1.09	1.25	1.17
Thymidine phosphorylase	TYMP	1.24	1.52*	1.28	4.52*	3.92*	4.88*	4.23*
Proteasome subunit beta type-8	PSMB8	1.37*	1.51*	1.39*	2.00*	1.62*	2.82*	1.92*
Transmembrane protein 171	TMEM171	1.40	1.51	0.90	1.34	1.23	0.97	0.84
GPI inositol-deacylase	PGAP1	1.24	1.51*	1.27	1.13	1.52	1.91*	1.21

Protein S100-A9	S100A9	0.60*	1.51*	1.41*	1.53*	1.35	1.39	2.46*
Exostosin-2	EXT2	1.09	1.51*	1.42*	1.25	1.38	1.43	1.85*
BAH and coiled-coil domain-containing protein 1	BAHCC1	0.54*	1.51*	0.94	0.43*	0.49*	0.40*	0.22*
Broad substrate specificity ATP-binding cassette transporter ABCG2	ABCG2	1.44*	1.51*	1.23	1.08	1.22	1.41	1.17
Mitochondrial fission process protein 1	MTFP1	1.06	1.51*	1.46*	1.44	1.61*	1.62*	1.80*
Rootletin	CROCC	0.49*	1.51*	0.96	0.74	0.50*	0.53*	0.43*
ER membrane protein complex subunit 7	EMC7	0.97	1.51*	1.43*	1.54*	1.50	1.59	1.99*
High affinity copper uptake protein 1	SLC31A1	1.36*	1.51*	1.17	1.17	1.11	1.59*	1.16
Ectonucleotide pyrophosphatase/phosphodiesterase family member 3	ENPP3	1.33	1.50	0.93	1.01	1.39	1.12	1.00
Zymogen granule protein 16 homolog B	EECP	1.16	1.50*	1.36*	1.18	2.47*	1.02	2.66*
Acyl-CoA:lysophosphatidylglycerol acyltransferase 1	LPGAT1	1.10	1.50*	1.18	1.37*	1.49*	1.82*	1.33

D. Up-regulated proteins unique to Mesalamine (MES) [86 proteins]

		Interventions						
		Control			*With LPS & Cytokines*			
Proteins	Genes	AQ	AQ+MES	**MES**	*LPS-Cyto*	*AQ*	*AQ+MES*	*MES*
Complement C1r subcomponent	C1R	0.94	1.31	14.51*	0.63	0.54	1.27	0.15*
Metalloreductase STEAP4	STEAP4	0.92	1.01	12.59*	0.82	0.89	0.98	1.29
Immunoglobulin lambda variable 1-51	IGLV1-51	0.85	1.31	8.76*	0.95	0.92	0.94	0.55*
Immunoglobulin lambda constant 2	IGLC2	0.66*	1.33	4.95*	1.42	3.25*	2.14*	1.55
Protein S100-A7A	S100A7A	0.27*	0.40*	4.36*	0.20*	0.48*	0.17*	0.46*
Histidine-rich glycoprotein	HRG	0.76	1.27	4.14*	0.61	0.90	1.03	1.39
Haptoglobin	HP	0.70*	1.32*	3.72*	1.15	3.32*	1.47*	1.19
Complement factor D	CFD	0.59*	1.43	3.72*	0.81	0.58	1.54	2.05*
Mediator of RNA polymerase II transcription subunit 16	MED16	1.30	1.31	3.61*	0.75	1.32	0.83	0.80
Immunoglobulin heavy constant alpha 1	IGHA1	0.68*	1.29*	3.28*	1.11	2.69*	1.30	2.32*
Mitotic-spindle organizing protein 1	MZT1	1.14	1.09	2.73*	0.92	0.63	0.71	2.13*
Immunoglobulin lambda-1 light chain		0.85	1.19	2.66*	1.84*	3.41*	4.29*	5.54*
Fibrinogen alpha chain	FGA	0.63*	0.68	2.55*	2.12*	0.76	1.05	2.43*
Probable phosphoglycerate mutase 4	PGAM4	0.53*	1.15	2.54*	0.51*	0.63	1.94*	2.77*
Fibrinogen gamma chain	FGG	0.35*	1.28	2.38*	2.58*	3.47*	3.42*	6.21*
Tetranectin	CLEC3B	0.72	1.19	2.37*	2.09*	1.76*	2.13*	2.78*
Serpin B12	SERPINB12	0.96	1.21	2.32*	1.07	3.22*	0.93	2.21*
Ubiquitin thioesterase OTU1	YOD1	1.22	1.23	2.20*	0.87	0.79	0.55	0.91
Tudor domain-containing protein 3	TDRD3	1.39	1.39	2.19*	0.80	0.14*	0.30*	0.42*
Complement C4-A	C4A	0.73*	1.29*	2.17*	1.82*	3.29*	3.86*	4.65*

T-complex protein 10A homolog 1	TCP10L	0.85	1.26	2.12*	0.87	0.83	2.79*	2.40*
Zinc finger protein 687	ZNF687	1.25	1.02	1.97*	0.80	1.33	1.36	0.92
Hemoglobin subunit epsilon	HBE1	0.77	1.36	1.95*	1.80*	2.27*	2.55*	3.81*
Pigment epithelium-derived factor	SERPINF1	0.57*	1.24	1.90*	1.24	0.96	2.19*	3.38*
Filamin-C	FLNC	1.23	0.84	1.90*	2.91*	3.79*	4.00*	2.41*
Alpha-2-macroglobulin	A2M	0.61*	1.48*	1.87*	1.88*	1.77*	2.51*	3.48*
SPARC	SPARC	0.54*	1.40*	1.85*	1.86*	1.88*	3.08*	3.36*
Serpin B3	SERPINB3	0.72*	1.14	1.82*	1.19	2.93*	0.78	1.96*
Immortalization up-regulated protein	IMUP	0.66*	1.26	1.81*	1.60*	1.16	1.10	1.75*
N-acetylglucosamine-1-phosphodiester alpha-N-acetylglucosaminidase	NAGPA	0.82	1.28	1.80*	0.75	0.93	1.45	1.58
Protein BCAP	ODF2L	0.43*	1.24	1.79*	2.23*	2.03*	2.99*	3.70*
N-acetyltransferase ESCO1	ESCO1	1.20	0.85	1.78*	1.70*	1.20	0.90	1.49
Cilia- and flagella-associated protein 100	CFAP100	0.89	1.31	1.78*	1.65*	1.48	2.37*	2.52*
Alpha-fetoprotein	AFP	0.54*	1.16	1.75*	1.41*	1.41*	2.14*	3.27*
Tumor necrosis factor receptor superfamily member 12A	TNFRSF12A	0.88	1.46	1.75*	1.47	1.35	1.30	1.52
Stromal interaction molecule 2	STIM2	0.77	1.30	1.75*	1.84*	1.54	2.36*	3.21*
Alpha-2-HS-glycoprotein	AHSG	0.70*	1.45*	1.73*	1.82*	1.47*	2.35*	2.40*
Laminin subunit alpha-2	LAMA2	1.06	1.20	1.71*	1.33	1.36	1.54	1.76
Thymosin beta-4	TMSB4X	0.55*	0.94	1.71*	1.20	1.01	1.17	1.93*
Protein-glutamine gamma-glutamyltransferase K	TGM1	1.13	1.39	1.71*	0.66	3.12*	0.49*	2.44*
Collagen alpha-1(IV) chain	COL4A1	0.50*	1.17	1.70*	1.82*	2.23*	2.22*	3.55*
Transmembrane protein 51	TMEM51	1.19	1.38	1.70*	1.29	0.27*	0.66	0.80
Serum paraoxonase/arylesterase 1	PON1	0.69	1.33	1.69*	1.47	3.15*	4.26*	4.58*
Lysyl oxidase homolog 3	LOXL3	0.95	1.24	1.68*	0.97	1.28	1.51	1.85*
Plexin domain-containing protein 2	PLXDC2	1.16	1.24	1.67*	2.61*	5.06*	7.61*	8.74*
Glutathione S-transferase Mu 2	GSTM2	0.74	1.19	1.67*	1.22	3.12*	3.94*	3.58*
Complement factor B	CFB	1.12	1.46	1.67*	2.18*	3.23*	2.38*	2.44*
tRNA methyltransferase 10 homolog A	TRMT10A	0.87	1.03	1.66*	0.99	1.01	1.30	2.12*
Gasdermin-A	GSDMA	1.05	1.25	1.66*	1.00	2.19*	0.72	0.83
Crooked neck-like protein 1	CRNKL1	0.97	1.39	1.66*	0.83	0.82	1.05	1.05
Glycoprotein endo-alpha-1,2-mannosidase	MANEA	1.08	1.29	1.66*	0.72	1.03	1.41	1.28
Pro-glucagon	GCG	0.65*	1.31*	1.64*	0.78	0.99	0.72	0.86
HLA class II histocompatibility antigen, DR beta 3 chain	HLA-DRB3	0.90	1.00	1.64*	12.42*	9.92*	10.03*	11.55*
Non-histone chromosomal protein HMG-17	HMGN2	0.56*	1.04	1.61*	0.87	0.80	1.21	1.52
Kynureninase	KYNU	0.82	1.00	1.61*	0.52*	0.92	1.21	1.74*
Vitamin D-binding protein	GC	0.71*	1.25	1.61*	1.69*	1.51*	2.10*	2.04*
Sugar phosphate exchanger 3	SLC37A3	1.09	1.42	1.60*	0.81	1.47	1.15	1.29

Hepatocyte growth factor-like protein	MST1	1.03	1.45*	1.60*	1.62*	2.56*	3.35*	4.24*
Interferon-induced protein with tetratricopeptide repeats 5	IFIT5	1.04	1.34	1.60*	2.00*	1.09	1.96*	2.05*
Cyclin-dependent kinase 2	CDK2	0.76	1.14	1.60*	0.69	1.16	0.98	1.35
Inter-alpha-trypsin inhibitor heavy chain H4	ITIH4	0.56*	1.42	1.59*	2.09*	1.62*	2.06*	3.18*
Probable serine carboxypeptidase CPVL	CPVL	0.97	1.32	1.59*	1.34	1.15	1.65*	2.03*
Mediator of RNA polymerase II transcription subunit 30	MED30	0.87	1.43	1.58*	0.93	0.94	1.28	1.01
Hemopexin	HPX	0.65*	1.28	1.58*	1.68*	1.54	1.99*	3.50*
Collagen alpha-3(VI) chain	COL6A3	0.29*	0.50*	1.58*	1.31	0.81	1.25	1.92*
Insulin-like growth factor-binding protein 4	IGFBP4	0.88	1.45*	1.57*	0.83	0.89	1.38	1.56
Vesicle-associated membrane protein 5	VAMP5	1.13	1.43	1.57	7.65*	6.90*	6.61*	6.29*
Midasin	MDN1	0.66*	1.29	1.56*	1.48*	1.53*	2.03*	2.59*
Nuclear envelope pore membrane protein POM 121	POM121	0.75	1.26	1.56*	0.87	0.22*	1.01	1.38
Uncharacterized protein MISP3	MISP3	1.18	1.36	1.56*	1.30	1.66	1.70	2.06*
Antithrombin-III	SERPINC1	0.69*	1.19	1.55*	1.84*	1.44*	1.87*	2.27*
Serine protease HTRA1	HTRA1	0.84	1.16	1.55*	0.94	1.32	1.42	1.99*
Inter-alpha-trypsin inhibitor heavy chain H1	ITIH1	0.48*	0.96	1.54*	0.48*	0.48*	1.13	1.53
Transcription initiation factor IIA subunit 2	GTF2A2	1.36	1.35	1.54*	1.37	0.59	0.36*	0.48*
Cystatin-S	CST4	0.83	1.14	1.53*	1.06	2.72*	1.49	2.96*
Frizzled-5	FZD5	1.21	1.25	1.53*	1.21	1.09	1.03	1.13
Secretoglobin family 1D member 2	SCGB1D2	0.91	1.39	1.53*	1.04	3.37*	0.82	2.35*
Tumor necrosis factor receptor superfamily member 5	CD40	0.84	1.23	1.53	3.99*	4.56*	4.44*	4.22*
Heat shock 70 kDa protein 13	HSPA13	0.96	1.49*	1.51*	1.27	1.61*	1.99*	2.46*
Vitronectin	VTN	0.53*	1.33	1.51*	1.58*	1.55	1.93*	2.76*
SAP domain-containing ribonucleoprotein	SARNP	1.10	1.28	1.51*	0.98	0.53*	0.92	0.93
Ras-related protein Rab-33B	RAB33B	0.79	0.93	1.51*	0.76	0.86	0.78	0.96
Interferon-induced GTP-binding protein Mx1	MX1	1.07	1.34	1.50*	3.61*	4.42*	5.17*	4.06*
Apolipoprotein E	APOE	0.90	1.42*	1.50*	1.64*	1.69*	1.93*	2.28*
MANSC domain-containing protein 1	MANSC1	0.94	1.34	1.50*	1.10	2.34*	2.35*	1.72*
5-formyltetrahydrofolate cyclo-ligase	MTHFS	1.17	1.44	1.50*	1.19	0.75	0.95	1.15

E. Common up-regulated proteins between Aquamin and Aquamin plus Mesalamine [30 proteins]

		Interventions						
		Control			*With LPS & Cytokines*			
Proteins	Genes	**AQ**	**AQ+MES**	MES	*LPS-Cyto*	*AQ*	*AQ+MES*	*MES*
Fibronectin type III and SPRY domain-containing protein 1	FSD1	3.62*	3.96*	1.02	0.70	2.52*	1.94*	0.49*
Cadherin-17	CDH17	3.59*	3.47*	1.05	0.91	2.69*	2.67*	0.73*

Calcium/manganese antiporter SLC30A10	SLC30A10	3.21*	2.20*	1.22	1.36	2.24*	2.25*	0.82
Putative nucleoside diphosphate kinase	NME2P1	2.99*	1.90*	1.48*	2.39*	0.55*	0.75	0.60*
Sulfate transporter	SLC26A2	2.55*	1.80*	0.90	0.65*	0.48*	0.77	0.44*
Cytochrome b	MT-CYB	2.22*	1.67*	1.38	3.21*	5.40*	5.01*	3.12*
Desmoglein-2	DSG2	2.17*	2.21*	0.93	1.08	2.03*	2.03*	1.45*
Malignant fibrous histiocytoma-amplified sequence 1	MFHAS1	2.14*	1.68*	1.35	1.22	1.41	1.16	1.12
Protocadherin-1	PCDH1	2.10*	2.09*	1.14	0.88	1.93*	1.95*	1.13
Ubiquinone biosynthesis protein COQ4 homolog, mitochondrial	COQ4	1.99*	1.66*	0.97	1.02	0.88	0.76	0.40*
TPA-induced transmembrane protein	TTMP	1.81*	1.96*	1.42	2.23*	4.60*	4.29*	3.70*
Tetraspanin-33	TSPAN33	1.76*	1.53	1.49	2.39*	3.91*	2.87*	2.46*
Membrane-spanning 4-domains subfamily A member 10	MS4A10	1.73*	2.21*	1.28	1.40	2.17*	1.20	1.03
[Pyruvate dehydrogenase [acetyl-transferring]]-phosphatase 2, mitochondrial	PDP2	1.70*	1.56*	1.29	1.09	0.59	0.86	0.90
Sodium/myo-inositol cotransporter	SLC5A3	1.68*	1.70*	1.41	2.45*	3.59*	4.91*	2.00*
Testis-expressed protein 9	TEX9	1.68*	1.53*	0.95	1.42*	1.70*	2.57*	2.77*
Arginase-2, mitochondrial	ARG2	1.67*	1.74*	1.25	1.44	1.55	1.51	0.93
Glycerophosphodiester phosphodiesterase 1	GDE1	1.65*	1.63*	1.36*	2.17*	3.02*	3.04*	1.72*
Sodium-dependent neutral amino acid transporter B(0)AT1	SLC6A19	1.63*	2.12*	0.93	1.71*	1.74*	1.12	0.47*
Protein O-glucosyltransferase 3	POGLUT3	1.62*	1.67*	1.49*	0.96	0.48*	0.78	1.14
Ubiquilin-2	UBQLN2	1.61*	1.52*	1.41*	1.44*	0.79	0.82	0.89
Endoplasmic reticulum membrane adapter protein XK	XK	1.59*	1.81*	1.36	2.33*	3.37*	4.41*	2.84*
Fatty-acid amide hydrolase 2	FAAH2	1.58*	1.52	1.12	1.00	1.24	1.44	0.97
Solute carrier family 53 member 1	XPR1	1.58*	1.51	1.38	1.17	1.61	1.70*	1.32
Homeobox protein DBX1	DBX1	1.57*	1.54*	1.49*	3.07*	6.91*	6.60*	5.35*
Battenin	CLN3	1.57*	2.13*	1.44*	1.94*	1.87*	2.41*	2.39*
Protein YIF1A	YIF1A	1.55*	1.60	1.42	2.21*	3.72*	3.42*	2.33*
Cadherin-3	CDH3	1.54*	1.58*	1.00	1.05	1.39	1.36	0.82
LHFPL tetraspan subfamily member 2 protein	LHFPL2	1.51*	1.61*	1.23	1.92*	2.46*	1.60	1.57
ATP-binding cassette sub-family C member 2	ABCC2	1.50*	2.42*	1.43*	1.53*	1.48*	1.89*	1.60*

F. Common up-regulated proteins between Aquamin and Mesalamine [10 proteins]

		Interventions						
		Control			*With LPS & Cytokines*			
Proteins	Genes	**AQ**	AQ+MES	**MES**	*LPS-Cyto*	*AQ*	*AQ+MES*	*MES*
Arachidonate 12-lipoxygenase, 12R-type	ALOX12B	2.22*	1.37	7.85*	0.47*	2.58*	0.92	1.08
Repetin	RPTN	1.60*	1.00	4.84*	0.42*	2.18*	0.67	3.40*

Serine/threonine-protein kinase 31	STK31	2.24*	1.32	4.57*	0.59*	2.06*	0.73	1.99*
Interferon-related developmental regulator 1	IFRD1	2.60*	0.80	1.96*	3.12*	1.60	1.01	0.77
Apolipoprotein L5	APOL5	1.57*	0.98	1.90*	1.78*	1.11	1.62	1.76
Zinc finger protein 284	ZNF284	1.90*	1.45	1.70*	1.88*	0.68	0.99	2.29*
Meiosis regulator and mRNA stability factor 1	MARF1	1.56*	1.10	1.65*	0.81	1.40	0.92	1.12
Ribonucleoside-diphosphate reductase subunit M2	RRM2	1.74*	1.38	1.58*	0.79	0.67	0.60	0.55*
High affinity cationic amino acid transporter 1	SLC7A1	1.76*	1.42*	1.56*	2.39*	3.41*	3.56*	2.53*
Ferredoxin-2, mitochondrial	FDX2	1.78*	1.45	1.55*	1.59*	2.48*	1.91*	1.27

G. Common up-regulated proteins between Mesalamine and Aquamin plus Mesalamine [187 proteins]

		Interventions						
		Control			*With LPS & Cytokines*			
Proteins	Genes	AQ	**AQ+MES**	**MES**	*LPS-Cyto*	*AQ*	*AQ+MES*	*MES*
C-X-C motif chemokine 10	CXCL10	0.72	34.05*	43.44*	0.97	0.83	30.10*	22.62*
Immunoglobulin kappa variable 4-1	IGKV4-1	0.63*	2.29*	37.17*	0.69	0.85	1.24	0.95
Immunoglobulin heavy constant gamma 4	IGHG4	0.64*	1.95*	32.94*	0.66*	0.72	1.67*	0.96
Complement factor H	CFH	0.62*	2.03*	27.05*	0.74	0.62	1.68*	1.72*
Immunoglobulin heavy variable 3-49	IGHV3-49	0.94	2.96*	25.72*	0.91	1.41	2.01*	0.78
Immunoglobulin heavy variable 3-7	IGHV3-7	0.93	2.12*	23.53*	0.83	1.48	1.53	0.58
CD5 antigen-like	CD5L	0.66	1.53	23.17*	0.77	0.69	0.82	0.29*
Immunoglobulin heavy constant alpha 2	IGHA2	1.13	3.35*	14.54*	0.74	1.90*	1.16	4.45*
Calmodulin-like protein 3	CALML3	1.02	52.36*	12.16*	0.75	1.15	0.24*	0.82
Proline-rich protein 9	PRR9	1.25	15.97*	11.14*	0.74	1.51	0.43*	0.33*
Apolipoprotein A-II	APOA2	0.90	2.24*	10.88*	0.84	1.17	1.89*	2.53*
Retroviral-like aspartic protease 1	ASPRV1	0.92	3.16*	10.29*	0.39*	1.54*	0.49*	1.97*
cAMP-dependent protein kinase inhibitor beta	PKIB	0.88	6.48*	9.41*	2.44*	2.06*	11.12*	18.67*
Apolipoprotein A-IV	APOA4	0.95	3.94*	8.02*	1.53*	1.25	2.60*	3.78*
Plastin-2	LCP1	0.68*	2.28*	7.32*	0.81	0.92	1.85*	2.39*
Alpha-2-macroglobulin-like protein 1	A2ML1	1.27*	2.46*	5.56*	0.41*	1.85*	0.34*	1.36*
Solute carrier family 2, facilitated glucose transporter member 5	SLC2A5	1.25*	6.90*	5.49*	1.35*	1.55*	6.09*	4.52*
Collagen alpha-1(II) chain	COL2A1	1.18	4.35*	5.26*	1.58*	1.09	3.94*	5.38*
Chromogranin-A	CHGA	0.64*	5.56*	5.04*	0.86	1.34	4.03*	6.57*
HLA class II histocompatibility antigen, DM alpha chain	HLA-DMA	1.43	5.47*	4.95*	1.02	1.13	5.22*	4.36*
Complement C2	C2	0.70	2.82*	4.91*	0.68	0.79	3.36*	3.92*
Immunoglobulin kappa constant	IGKC	0.71*	1.56*	4.90*	1.34*	2.92*	1.88*	2.00*
Galectin-7	LGALS7	0.90	4.40*	4.72*	1.18	2.20*	0.97	1.65
Follistatin-related protein 1	FSTL1	0.95	4.04*	4.57*	1.47*	1.61	3.52*	5.73*

Secretogranin-2	SCG2	0.74	3.08*	4.35*	0.99	1.09	3.27*	5.45*
Desmoglein-3	DSG3	0.68	3.24*	4.23*	0.59	0.94	1.57	2.39*
Synaptic vesicle membrane protein VAT-1 homolog-like	VAT1L	0.71	3.05*	4.20*	0.93	1.19	2.75*	4.58*
HLA class II histocompatibility antigen, DR beta 4 chain	HLA-DRB4	0.71	3.84*	4.17*	0.84	1.08	4.68*	3.60*
Centrosomal protein of 85 kDa	CEP85	0.93	4.39*	4.17*	1.04	1.23	2.87*	4.16*
Receptor-type tyrosine-protein phosphatase zeta	PTPRZ1	0.81	2.75*	4.03*	0.97	1.19	2.32*	3.43*
Phospholipase A2, membrane associated	PLA2G2A	1.31	4.25*	3.95*	3.18*	8.37*	15.64*	17.81*
Ferritin light chain	FTL	0.52*	2.72*	3.90*	1.95*	0.58	4.12*	9.36*
Plasmalemma vesicle-associated protein	PLVAP	0.75	3.13*	3.90*	1.02	1.13	3.00*	3.92*
Ubiquitin D	UBD	1.03	4.25*	3.84*	2.84*	3.54*	12.39*	12.06*
Complement C3	C3	0.68*	2.23*	3.76*	2.82*	3.40*	4.41*	3.80*
EH domain-containing protein 3	EHD3	0.76	3.83*	3.66*	1.19	1.06	2.38*	3.59*
Tenascin-X	TNXB	0.89	2.86*	3.62*	0.93	1.02	2.74*	3.87*
2'-5'-oligoadenylate synthase 2	OAS2	0.80	3.38*	3.47*	1.71*	2.58*	9.01*	8.23*
Beta-parvin	PARVB	0.82	2.48*	3.45*	0.96	1.17	2.19*	3.29*
Guanine nucleotide-binding protein subunit alpha-15	GNA15	0.90	2.12*	3.41*	0.64	1.28	1.26	1.03
Ferroxidase HEPHL1	HEPHL1	0.77	61.07*	3.28*	0.93	1.02	0.31*	1.91
Moesin	MSN	0.69*	1.72*	3.19*	1.45*	1.46*	2.21*	3.03*
Phospholipid transfer protein	PLTP	0.70	2.51*	3.16*	0.97	0.87	2.48*	3.00*
Cadherin-13	CDH13	0.84	2.41*	3.15*	0.88	1.01	2.44*	3.83*
Vasopressin-neurophysin 2-copeptin	AVP	0.94	3.13*	3.08*	1.27	1.08	2.41*	3.99*
Fibroleukin	FGL2	0.77	3.23*	3.07*	0.79	0.93	3.82*	3.15*
Membrane primary amine oxidase	AOC3	0.85	2.15*	3.07*	0.93	1.18	1.67	2.86*
Interleukin-1 receptor accessory protein	IL1RAP	0.61*	2.35*	2.99*	0.79	0.92	2.31*	3.58*
cAMP-specific 3',5'-cyclic phosphodiesterase 4C	PDE4C	0.89	2.07*	2.99*	0.79	1.82*	2.80*	3.99*
Myeloblastin	PRTN3	0.84	3.59*	2.95*	0.55*	1.93*	0.45*	1.61
Alpha-1-antichymotrypsin	SERPINA3	1.08	1.90*	2.88*	1.28	3.87*	1.63	2.12*
Plasma kallikrein	KLKB1	0.59*	2.06*	2.86*	0.72	0.82	1.89*	2.72*
Glycolipid transfer protein domain-containing protein 2	GLTPD2	1.47*	2.85*	2.85*	1.93*	2.81*	2.12*	3.41*
Selenoprotein P	SELENOP	0.80	1.87*	2.83*	0.81	1.24	1.76*	1.95*
Protein S100-A3	S100A3	1.12	26.05*	2.82*	0.58*	1.60	1.77	3.12*
A disintegrin and metalloproteinase with thrombospondin motifs 13	ADAMTS13	0.68	2.03*	2.82*	0.65	0.85	2.28*	2.94*
Oncoprotein-induced transcript 3 protein	OIT3	0.70*	2.44*	2.81*	0.81	0.94	2.23*	3.12*
Contactin-1	CNTN1	0.65*	2.25*	2.79*	0.80	0.88	2.29*	3.42*
Retinol-binding protein 4	RBP4	0.67*	2.60*	2.77*	0.92	0.90	2.14*	2.97*
Carboxypeptidase A2	CPA2	1.24	3.77*	2.77*	0.76	1.74	4.32*	2.79*
Mammaglobin-B	SCGB2A1	1.32	5.33*	2.76*	0.64	1.80*	0.64	1.75

Fibromodulin	FMOD	0.64*	2.45*	2.75*	1.06	0.95	2.23*	3.19*
Heparin cofactor 2	SERPIND1	0.75	1.73*	2.75*	2.04*	3.83*	4.33*	7.38*
Collagen alpha-1(XI) chain	COL11A1	0.53*	2.32*	2.75*	0.87	0.94	2.20*	3.10*
Kininogen-1	KNG1	0.47*	2.27*	2.73*	1.71*	0.68	2.29*	2.32*
Alpha-1B-glycoprotein	A1BG	0.55*	1.99*	2.73*	0.77	0.76	2.05*	3.14*
Sulfotransferase 2A1	SULT2A1	1.49*	5.11*	2.72*	1.25	1.90*	2.73*	1.62
All-trans-retinol dehydrogenase [NAD(+)] ADH4	ADH4	1.07	2.55*	2.71*	2.95*	2.62*	2.01*	1.81*
Phosphatidylcholine-sterol acyltransferase	LCAT	0.65*	2.10*	2.67*	0.76	0.94	2.06*	2.94*
C-type mannose receptor 2	MRC2	0.88	2.19*	2.65*	0.96	1.06	2.07*	2.88*
Interferon-induced protein with tetratricopeptide repeats 3	IFIT3	1.24	2.65*	2.64*	1.98*	2.49*	4.45*	4.39*
Mitochondrial glutamate carrier 2	SLC25A18	0.86	1.95*	2.62*	0.94	1.58	2.10*	3.70*
FERM and PDZ domain-containing protein 1	FRMPD1	0.54*	2.61*	2.58*	0.47*	0.94	1.85*	2.22*
Beta-2-glycoprotein 1	APOH	1.26	2.33*	2.52*	2.43*	2.14*	2.35*	3.00*
Cerebellin-4	CBLN4	0.41*	1.58	2.52*	0.57*	0.78	1.73	1.95*
EGF-containing fibulin-like extracellular matrix protein 1	EFEMP1	0.62*	2.26*	2.51*	0.81	0.53*	1.88*	2.37*
Retinoic acid receptor responder protein 2	RARRES2	0.54*	1.61*	2.51*	0.66	0.68	1.46	2.08*
Sex hormone-binding globulin	SHBG	0.50*	2.02*	2.50*	0.70	0.75	1.95*	2.89*
Neural cell adhesion molecule 1	NCAM1	0.68*	2.18*	2.49*	0.81	0.77	2.12*	3.03*
Apolipoprotein C-I	APOC1	0.88	1.73*	2.48*	1.47*	3.07*	4.84*	3.83*
Hepatocyte growth factor activator	HGFAC	0.62*	2.13*	2.48*	0.77*	0.77	2.27*	3.01*
Collagen alpha-2(I) chain	COL1A2	0.53*	2.15*	2.47*	0.72	0.65	1.88*	2.71*
Serum amyloid A-4 protein	SAA4	0.63*	2.09*	2.47*	3.32*	9.01*	8.67*	16.73*
Carboxypeptidase E	CPE	0.79	2.20*	2.47*	0.75	0.99	1.70	2.53*
NEDD4 family-interacting protein 2	NDFIP2	1.07	2.34*	2.44*	0.95	1.12	2.19*	2.96*
Tyrosine-protein kinase receptor Tie-1	TIE1	0.68	1.62*	2.43*	0.79	0.81	2.09*	2.76*
Afamin	AFM	0.52*	2.16*	2.43*	0.71*	0.67*	2.00*	2.67*
Metalloproteinase inhibitor 3	TIMP3	0.80	3.49*	2.39*	0.78	0.88	2.09*	1.51
Latent-transforming growth factor beta-binding protein 4	LTBP4	0.82	2.90*	2.39*	1.01	1.40	1.61	3.16*
Lactotransferrin	LTF	0.98	1.77*	2.37*	2.39*	2.02*	2.78*	3.12*
Collagen alpha-1(XXI) chain	COL21A1	0.75	1.78*	2.36*	0.97	0.96	1.81*	2.34*
TRPM8 channel-associated factor 2	TCAF2	1.14	1.76*	2.34*	1.03	1.43	1.71	1.71
ERC protein 2	ERC2	0.70	2.27*	2.27*	0.79	0.70	1.76	2.58*
Scrapie-responsive protein 1	SCRG1	0.64*	2.21*	2.25*	0.80	0.99	1.82*	2.12*
Lymphocyte antigen 6D	LY6D	0.89	1.56	2.25*	2.10*	2.22*	1.53	1.51
Keratin, type I cuticular Ha4	KRT34	0.84	63.49*	2.24*	0.74	1.05	0.91	3.16*
Cytochrome c oxidase assembly protein COX11, mitochondrial	COX11	1.28	1.96*	2.23*	1.39	2.04*	2.52*	3.10*

Desmoglein-4	DSG4	1.01	65.35*	2.23*	0.77	1.18	1.51	2.16*
Glutathione S-transferase A5	GSTA5	0.75	1.99*	2.22*	0.78	0.90	1.92*	2.62*
Collagen alpha-1(III) chain	COL3A1	0.82	1.61*	2.22*	0.72	0.87	1.66*	2.04*
Pro-opiomelanocortin	POMC	0.96	2.08*	2.21*	2.71*	4.33*	6.13*	10.26*
Procollagen C-endopeptidase enhancer 1	PCOLCE	0.69	1.78*	2.20*	0.96	2.34*	2.41*	2.65*
Dynein axonemal heavy chain 1	DNAH1	0.51*	1.54*	2.15*	1.10	0.62*	2.04*	2.51*
Complement factor I	CFI	0.68	2.00*	2.08*	2.94*	6.10*	6.03*	11.20*
Plakophilin-1	PKP1	0.84	3.19*	2.07*	0.84	2.73*	0.56*	2.07*
Protein piccolo	PCLO	0.56*	1.75*	2.05*	0.55*	0.72	2.03*	2.36*
Cell adhesion molecule 1	CADM1	0.65*	1.89*	2.04*	0.63	0.89	1.76*	2.25*
Insulin-like growth factor II	IGF2	0.62*	2.07*	2.04*	0.74	0.48*	1.65*	2.22*
Keratin, type I cuticular Ha1	KRT31	0.68*	49.23*	2.04*	0.65*	0.92	0.80	2.96*
Albumin	ALB	0.63*	1.73*	2.02*	2.42*	1.96*	2.70*	2.09*
Mucin-3A	MUC3A	1.00	2.47*	2.01*	1.05	1.21	1.75*	1.67*
Interferon-induced transmembrane protein 3	IFITM3	0.94	2.48*	1.97*	1.60*	2.12*	4.68*	3.56*
Protein bassoon	BSN	0.50*	2.53*	1.96*	5.37*	19.19*	17.24*	29.63*
Ubiquitin carboxyl-terminal hydrolase 40	USP40	1.12	1.64*	1.96*	0.80	1.08	0.97	1.03
Inositol-trisphosphate 3-kinase C	ITPKC	1.17	2.04*	1.95*	1.21	1.37	1.86*	1.46
Secretogranin-3	SCG3	1.21	1.79*	1.95*	2.47*	2.79*	3.40*	4.48*
BPI fold-containing family A member 1	BPIFA1	1.11	2.30*	1.94*	1.49*	2.48*	1.40	3.23*
Retinal dehydrogenase 2	ALDH1A2	0.81	1.80*	1.94*	0.84	1.03	1.97*	2.31*
Tropomodulin-2	TMOD2	0.97	2.08*	1.93*	2.25*	1.95*	2.91*	4.70*
Keratin, type I cuticular Ha5	KRT35	1.08	26.02*	1.92*	0.71	0.74	0.68	0.97
Transcription cofactor vestigial-like protein 4	VGLL4	0.92	1.59*	1.92*	0.98	0.94	0.98	0.60
mRNA-decapping enzyme 1B	DCP1B	1.26	2.01*	1.92*	0.58*	1.35	0.76	1.52
Decreased expression in renal and prostate cancer protein	DERPC	1.02	1.57	1.92*	1.41	1.36	1.21	0.86
EF-hand domain-containing protein D1	EFHD1	0.66	43.85*	1.91*	0.69	0.69	0.84	3.11*
Hemoglobin subunit beta	HBB	1.03	2.23*	1.89*	2.18*	2.36*	3.19*	4.34*
Guanidinoacetate N-methyltransferase	GAMT	0.55*	1.98*	1.89*	0.67	0.57	1.54	1.94*
Sterile alpha motif domain-containing protein 9-like	SAMD9L	1.07	1.96*	1.88*	1.41	2.01*	3.01*	3.00*
Tropomyosin beta chain	TPM2	0.68*	1.91*	1.88*	0.86	0.83	1.59	2.29*
Polyglutamine-binding protein 1	PQBP1	1.06	1.73*	1.85*	1.25	0.26*	0.69	1.16
Apolipoprotein L1	APOL1	1.46	1.73*	1.82*	2.68*	3.15*	3.75*	2.64*
Lumican	LUM	0.67*	1.73*	1.81*	1.41*	1.54*	2.50*	3.78*
HLA class II histocompatibility antigen, DP beta 1 chain	HLA-DPB1	0.31*	2.33*	1.80*	15.14*	13.55*	15.68*	15.01*
Nucleolar and spindle-associated protein 1	NUSAP1	1.44*	1.64*	1.78*	1.33	0.54	0.27*	0.86
Retinol-binding protein 2	RBP2	1.29*	2.84*	1.78*	1.20	2.02*	1.36*	1.25*

Secreted and transmembrane protein 1	SECTM1	1.01	1.76*	1.78*	14.70*	8.17*	7.82*	13.21*
Collagen alpha-1(V) chain	COL5A1	1.02	1.90*	1.77*	1.10	1.65*	2.12*	2.62*
Desumoylating isopeptidase 1	DESI1	0.95	1.72*	1.76*	1.06	1.21	1.64	2.27*
Inter-alpha-trypsin inhibitor heavy chain H2	ITIH2	0.86	1.71*	1.75*	1.61*	2.05*	2.42*	3.06*
CD166 antigen	ALCAM	1.12	1.95*	1.74*	2.10*	1.22	2.14*	1.78*
Sulfhydryl oxidase 2	QSOX2	1.47*	1.88*	1.74*	2.20*	3.05*	3.18*	3.04*
Microtubule-associated proteins 1A/1B light chain 3 beta 2	MAP1LC3B2	1.26	1.67*	1.73*	1.34	1.32	1.61	1.83*
Transforming growth factor-beta-induced protein ig-h3	TGFBI	0.93	1.95*	1.72*	1.09	1.36	1.69*	2.50*
Opioid growth factor receptor	OGFR	1.32*	2.03*	1.72*	1.66*	1.39	1.91*	2.03*
DNA-binding protein SMUBP-2	IGHMBP2	0.58*	1.53*	1.72*	2.47*	3.42*	3.38*	5.07*
Mammaglobin-A	SCGB2A2	1.06	1.93*	1.71*	1.32	4.09*	1.54	3.31*
Keratin, type I cuticular Ha3-I	KRT33A	0.87	24.84*	1.71*	0.94	0.91	1.29	2.44*
Neugrin	NGRN	1.04	1.57*	1.71*	1.11	0.65	0.56*	0.90
Sushi domain-containing protein 2	SUSD2	1.30	2.95*	1.70*	1.09	1.57	1.60	0.92
SPARC-related modular calcium-binding protein 2	SMOC2	0.84	1.62*	1.69*	1.38	2.05*	1.83*	1.95*
Transgelin	TAGLN	0.82	2.04*	1.68*	1.62*	1.71	2.44*	2.91*
Cornifin-B	SPRR1B	0.79	1.62*	1.68*	1.14	4.30*	0.46*	3.71*
ER degradation-enhancing alpha-mannosidase-like protein 2	EDEM2	1.23	1.67*	1.68*	1.01	1.30	1.56	1.89*
Alpha-2-antiplasmin	SERPINF2	0.86	1.62*	1.67*	1.84*	2.47*	5.09*	6.18*
Fibrinogen beta chain	FGB	0.56*	2.04*	1.67*	1.92*	3.53*	3.54*	6.17*
Exocyst complex component 3-like protein 4	EXOC3L4	0.82	1.70*	1.67*	0.79	0.71	1.71*	1.62
F-box only protein 2	FBXO2	1.08	2.13*	1.67*	0.90	1.73*	2.19*	1.63
Laminin subunit alpha-1	LAMA1	0.55*	2.01*	1.66*	1.60*	2.30*	2.88*	3.96*
Lymphocyte function-associated antigen 3	CD58	1.37*	1.92*	1.66*	1.76*	2.20*	2.42*	2.22*
Glutamyl aminopeptidase	ENPEP	0.97	2.32*	1.66*	1.38*	1.43*	1.34	0.94
Apolipoprotein C-III	APOC3	0.91	2.22*	1.66*	2.09*	2.28*	2.65*	3.96*
Laminin subunit beta-1	LAMB1	0.54*	2.04*	1.65*	1.58*	2.39*	2.83*	3.78*
Protransforming growth factor alpha	TGFA	1.48*	1.58*	1.65*	1.77*	3.24*	3.42*	3.06*
Ileal sodium/bile acid cotransporter	SLC10A2	1.33*	2.98*	1.64*	1.53*	1.67*	2.16*	1.42
MARVEL domain-containing protein 3	MARVELD3	1.31	1.82*	1.63*	1.72*	0.97	1.89*	1.56
Laminin subunit gamma-1	LAMC1	0.61*	1.97*	1.62*	1.54*	2.28*	2.68*	3.65*
Ethanolaminephosphotransferase 1	SELENOI	1.27	2.10*	1.62*	0.85	1.42	1.42	1.22
Nidogen-2	NID2	0.73	1.89*	1.61*	1.45*	2.02*	2.55*	3.43*
DnaJ homolog subfamily C member 15	DNAJC15	1.21	1.83*	1.61*	2.28*	3.11*	2.09*	2.92*
Trophoblast glycoprotein	TPBG	1.42*	1.68*	1.61*	1.90*	2.67*	2.69*	1.69
Dual oxidase maturation factor 2	DUOXA2	1.45*	1.75*	1.60*	1.11	1.43	1.63*	1.04

Protein preY, mitochondrial	PYURF	1.41*	1.93*	1.58*	1.27	1.06	1.27	1.34
N-acetyllactosaminide beta-1,3-N-acetylglucosaminyltransferase 2	B3GNT2	1.29	1.91*	1.58*	1.36	2.26*	2.13*	2.17*
Leukocyte receptor cluster member 8	LENG8	1.48*	1.79*	1.57*	1.20	0.21*	0.51*	0.13*
Collagen alpha-1(XII) chain	COL12A1	0.71	1.58	1.57*	2.12*	3.69*	6.05*	6.67*
Renin	REN	1.34	1.56*	1.57*	0.66	0.96	0.87	0.83
Growth-regulated alpha protein	CXCL1	0.91	1.50	1.56*	1.02	1.15	1.36	1.44
2'-5'-oligoadenylate synthase-like protein	OASL	1.25	1.70*	1.54*	1.09	1.26	1.71*	1.54
Coiled-coil domain-containing protein 127	CCDC127	1.41	1.65*	1.53	1.85*	2.71*	2.49*	2.29*
Inositol-3-phosphate synthase 1	ISYNA1	1.33*	1.61*	1.53*	2.28*	3.96*	2.92*	3.27*
Succinate dehydrogenase assembly factor 1, mitochondrial	SDHAF1	1.26	1.62*	1.52	1.27	0.77	0.74	1.25
Hemoglobin subunit alpha	HBA1	0.78*	1.53*	1.52*	1.62*	1.74*	2.44*	3.35*
HLA class I histocompatibility antigen, B alpha chain	HLA-B	1.47*	2.37*	1.52*	7.62*	5.91*	7.94*	8.75*
Cysteine protease ATG4C	ATG4C	1.22	1.51	1.51*	1.04	0.50*	0.27*	0.39*
Periostin	POSTN	0.66*	1.63*	1.51*	1.02	2.43*	2.54*	3.38*
Keratinocyte-associated transmembrane protein 2	KCT2	1.12	1.69*	1.51*	2.82*	2.19*	2.57*	2.90*
Ankyrin repeat domain-containing protein 27	ANKRD27	1.10	1.61*	1.51*	1.81*	1.46*	1.06	1.92*
Solute carrier family 66 member 3	SLC66A3	1.36	2.04*	1.50*	0.98	1.35	1.63	1.02

Values represent the abundance ratio from organoids (n=4 subjects) compared to the control. These proteins were up-regulated at a 1.5-fold change (<2% FDR). Corresponding abundance ratios from the other treatment groups are provided for comparison. Proteins common among groups and unique to individual groups under control conditions are presented. *Indicates significance compared to the control (at p<0.05).
