# Supplementary material for: Proteomic profile of human colon organoids: effects of a multi-mineral intervention alone and in the presence of pro-inflammatory and anti-inflammatory treatments
Source: Front Gastroenterol (Lausanne). 2025 Jul 2;4:1592669. doi: 10.3389/fgstr.2025.1592669 (PMC12952359; doi:10.3389/fgstr.2025.1592669)
Supplement: Supplementary Table S1 — Mineral composition of Aquamin. [file DataSheet1.zip › Table S4.pdf]

Supplement Table 4. Down-regulated proteins influenced by Aquamin and Mesalamine under control conditions (with 1.5-fold).

A. Common among three groups – Aquamin (AQ), Mesalamine (MES) and Aquamin plus Mesalamine (AQ+MES) [194 proteins]

		Interventions						
		Control			*With LPS & Cytokines*			
Proteins	Genes	**AQ**	**AQ+MES**	**MES**	*LPS-Cyto*	*AQ*	*AQ+MES*	*MES*
Fermitin family homolog 2	FERMT2	0.05*	0.35*	0.25*	2.05*	9.27*	9.50*	10.36*
Tubulin alpha-3C chain	TUBA3C	0.07*	0.27*	0.11*	1.78*	10.04*	7.83*	8.39*
Telomere-associated protein RIF1	RIF1	0.11*	0.18*	0.18*	0.26*	0.35*	0.29*	0.28*
Calcium/calmodulin-dependent protein kinase type II subunit beta	CAMK2B	0.12*	0.10*	0.17*	0.26*	0.64*	0.53*	0.47*
Aldehyde oxidase	AOX1	0.14*	0.14*	0.27*	2.11*	11.69*	8.47*	9.08*
Survival motor neuron protein	SMN1	0.18*	0.25*	0.29*	0.40*	0.38*	0.38*	0.43*
Rho guanine nucleotide exchange factor 3	ARHGEF3	0.18*	0.44*	0.36*	0.12*	0.48*	0.17*	0.24*
Heparan sulfate glucosamine 3-O-sulfotransferase 1	HS3ST1	0.19*	0.35*	0.29*	1.17	4.51*	3.55*	5.50*
Kelch-like ECH-associated protein 1	KEAP1	0.19*	0.33*	0.37*	0.18*	0.39*	0.32*	0.29*
Rho GTPase-activating protein 29	ARHGAP29	0.19*	0.35*	0.41*	0.33*	0.58	0.50*	0.35*
Zinc finger protein 654	ZNF654	0.19*	0.24*	0.21*	0.15*	0.36*	0.33*	0.29*
Gamma-tubulin complex component 3	TUBGCP3	0.19*	0.27*	0.29*	0.31*	0.86	0.80	0.82
Zinc finger protein 136	ZNF136	0.20*	0.18*	0.20*	0.20*	0.26*	0.23*	0.17*
TBC1 domain family member 25	TBC1D25	0.21*	0.09*	0.08*	0.10*	0.11*	0.15*	0.07*
Keratin, type II cytoskeletal 2 epidermal	KRT2	0.21*	0.26*	0.47*	0.33*	0.50*	0.23*	0.35*
Tubulin beta-1 chain	TUBB1	0.21*	0.28*	0.24*	1.96*	9.06*	7.77*	9.62*
Leydig cell tumor 10 kDa protein homolog	C19orf53	0.21*	0.19*	0.20*	0.43*	0.37*	0.36*	0.45*
Bone morphogenetic protein 1	BMP1	0.22*	0.37*	0.31*	0.91	3.52*	2.50*	3.63*
Ribosomal biogenesis protein LAS1L	LAS1L	0.22*	0.26*	0.25*	0.28*	0.62*	0.51*	0.40*
WD repeat and FYVE domain-containing protein 3	WDFY3	0.23*	0.09*	0.12*	0.09*	0.07*	0.18*	0.06*
Insulin-like growth factor-binding protein complex acid labile subunit	IGFALS	0.23*	0.37*	0.33*	2.38*	12.16*	9.41*	11.08*
Guanine nucleotide-binding protein-like 3-like protein	GNL3L	0.23*	0.38*	0.37*	0.14*	0.22*	0.31*	0.20*
AT-rich interactive domain-containing protein 2	ARID2	0.24*	0.38*	0.30*	0.30*	0.48*	0.35*	0.48*
Filaggrin-2	FLG2	0.24*	0.30*	0.44*	0.94	0.69*	0.34*	0.69*
Mediator of RNA polymerase II transcription subunit 12	MED12	0.25*	0.27*	0.31*	0.42*	0.97	0.81	0.84
Zinc finger protein 592	ZNF592	0.25*	0.28*	0.27*	0.33*	0.59	0.55	0.49*
Histone-lysine N-methyltransferase EHMT2	EHMT2	0.25*	0.28*	0.29*	0.40*	1.50	1.10	1.16
Zinc finger and BTB domain-containing protein 11	ZBTB11	0.25*	0.37*	0.31*	0.47*	0.42*	0.29*	0.49*

Cap-specific mRNA (nucleoside-2'-O-)-methyltransferase 2	CMTR2	0.25*	0.45*	0.57*	0.26*	0.82	0.62	0.87
Protein kinase C theta type	PRKCQ	0.26*	0.08*	0.11*	0.16*	0.14*	0.22*	0.11*
Zinc finger protein with KRAB and SCAN domains 4	ZKSCAN4	0.27*	0.19*	0.22*	0.15*	0.37*	0.19*	0.22*
Activating transcription factor 7-interacting protein 1	ATF7IP	0.27*	0.43*	0.38*	0.47*	0.46*	0.50*	0.46*
Integrin alpha-E	ITGAE	0.27*	0.09*	0.14*	0.13*	0.10*	0.22*	0.09*
Mediator of RNA polymerase II transcription subunit 23	MED23	0.28*	0.39*	0.32*	0.32*	0.85	0.69	0.70
Protein ECT2	ECT2	0.28*	0.38*	0.40*	0.25*	0.64	0.62	0.55*
Oxytocin-neurophysin 1	OXT	0.28*	0.52*	0.47*	0.20*	0.20*	0.50*	0.37*
Structural maintenance of chromosomes protein 6	SMC6	0.28*	0.28*	0.27*	0.41*	1.10	0.92	1.02
All-trans-retinol dehydrogenase [NAD(+)] ADH7	ADH7	0.29*	0.43*	0.31*	2.22*	11.41*	8.22*	10.30*
Trafficking kinesin-binding protein 1	TRAK1	0.29*	0.38*	0.48*	0.36*	0.46*	0.61	0.58
Primary cilium assembly protein FAM149B1	FAM149B1	0.29*	0.24*	0.24*	0.30*	0.85	0.39*	0.60
SURP and G-patch domain-containing protein 2	SUGP2	0.29*	0.33*	0.39*	0.28*	0.24*	0.23*	0.19*
Structural maintenance of chromosomes protein 5	SMC5	0.30*	0.36*	0.38*	0.34*	0.60	0.46*	0.61*
Tensin-4	TNS4	0.30*	0.47*	0.46*	0.43*	0.67	0.52*	0.45*
Transformation/transcription domain-associated protein	TRRAP	0.30*	0.35*	0.40*	0.54*	1.21	1.16	1.04
Biogenesis of lysosome-related organelles complex 1 subunit 3	BLOC1S3	0.30*	0.42*	0.36*	0.49*	0.50*	0.39*	0.62
Betaine--homocysteine S-methyltransferase 1	BHMT	0.31*	0.41*	0.31*	2.44*	13.21*	10.27*	6.92*
General transcription factor 3C polypeptide 3	GTF3C3	0.31*	0.41*	0.39*	0.71	0.94	0.93	0.91
E3 SUMO-protein ligase ZNF451	ZNF451	0.32*	0.37*	0.35*	0.19*	0.43*	0.44*	0.36*
E3 ubiquitin-protein ligase Midline-1	MID1	0.32*	0.37*	0.40*	0.41*	0.38*	0.40*	0.35*
Echinoderm microtubule-associated protein-like 3	EML3	0.32*	0.50*	0.48*	0.47*	0.21*	0.10*	0.30*
PHD finger protein 6	PHF6	0.32*	0.43*	0.46*	0.26*	0.65*	0.64*	0.69*
Anaphase-promoting complex subunit 1	ANAPC1	0.33*	0.29*	0.32*	0.60*	2.35*	1.97*	2.10*
Probable ATP-dependent RNA helicase DDX20	DDX20	0.33*	0.54*	0.49*	0.51*	0.75	0.70	0.73
Matrix metalloproteinase-28	MMP28	0.33*	0.49*	0.56*	0.80	3.03*	2.32*	2.79*
Gametogenetin-binding protein 2	GGNBP2	0.34*	0.37*	0.50*	0.34*	0.51*	0.46*	0.40*
Transmembrane protein 209	TMEM209	0.34*	0.43*	0.44*	0.42*	1.00	0.80	0.87
DNA topoisomerase 2-binding protein 1	TOPBP1	0.34*	0.50*	0.60*	0.31*	0.44*	0.44*	0.51*
Helicase-like transcription factor	HLTF	0.34*	0.41*	0.37*	0.28*	0.52	0.63	0.51
Protein furry homolog	FRY	0.34*	0.64*	0.59*	0.65	0.30*	0.36*	0.55
Centrosomal protein of 131 kDa	CEP131	0.35*	0.36*	0.35*	0.21*	0.33*	0.09*	0.09*
Parathyroid hormone/parathyroid hormone-related peptide receptor	PTH1R	0.35*	0.33*	0.47*	1.60*	8.24*	7.16*	7.08*
Endothelial lipase	LIPG	0.35*	0.46*	0.43*	1.12	4.07*	3.10*	3.50*
NEDD4-binding protein 3	N4BP3	0.35*	0.47*	0.43*	0.20*	0.36*	0.29*	0.26*

HAUS augmin-like complex subunit 3	HAUS3	0.35*	0.39*	0.40*	0.45*	0.66	0.62	0.59
Molybdenum cofactor sulfurase	MOCOS	0.36*	0.52*	0.58*	0.29*	0.24*	0.26*	0.25*
Nuclear receptor corepressor 1	NCOR1	0.36*	0.52*	0.51*	0.38*	0.32*	0.33*	0.51*
C-X-C motif chemokine 14	CXCL14	0.36*	0.37*	0.42*	0.28*	0.28*	0.42*	0.39*
Keratin, type II cytoskeletal 71	KRT71	0.36*	0.52*	0.41*	0.94	1.15	0.39*	0.51*
E3 ubiquitin-protein transferase MAEA	MAEA	0.36*	0.45*	0.47*	0.45*	0.72	0.67	0.61*
Beclin-1	BECN1	0.36*	0.54*	0.52*	0.41*	0.67	0.66	0.61*
WD repeat-containing protein 3	WDR3	0.37*	0.34*	0.36*	0.43*	0.82	0.73	0.70*
AP-4 complex subunit beta-1	AP4B1	0.37*	0.55*	0.63*	0.50*	0.67	0.68	0.55
TBC1 domain family member 2B	TBC1D2B	0.37*	0.52*	0.55*	0.35*	0.89	0.73	0.60
Nucleolar MIF4G domain-containing protein 1	NOM1	0.38*	0.40*	0.40*	0.60*	0.37*	0.27*	0.32*
Small subunit processome component 20 homolog	UTP20	0.39*	0.36*	0.37*	0.42*	0.86	0.84	1.24
Serine/threonine-protein kinase tousled-like 2	TLK2	0.39*	0.59*	0.61*	0.53*	0.32*	0.30*	0.34*
Liprin-alpha-4	PPFIA4	0.39*	0.45*	0.66*	1.37	0.17*	0.20*	0.32*
Polyamine-modulated factor 1-binding protein 1	PMFBP1	0.39*	0.15*	0.13*	0.14*	0.15*	0.25*	0.08*
Myotubularin-related protein 13	SBF2	0.39*	0.59*	0.60*	0.32*	0.67	0.42*	0.40*
Anaphase-promoting complex subunit 5	ANAPC5	0.39*	0.38*	0.39*	0.53*	1.56*	1.01	1.02
Integrator complex subunit 4	INTS4	0.39*	0.43*	0.46*	0.61*	1.10	1.22	1.14
Transcription factor Sp1	SP1	0.39*	0.49*	0.53*	0.26*	0.40*	0.44*	0.46*
Integrator complex subunit 5	INTS5	0.39*	0.34*	0.33*	0.35*	0.86	0.81	0.84
Deoxycytidine kinase	DCK	0.40*	0.45*	0.56*	0.34*	0.63	0.57*	0.54*
PCNA-interacting partner	PARPBP	0.40*	0.11*	0.08*	0.13*	0.12*	0.16*	0.06*
Probable protein phosphatase 1N	PPM1N	0.40*	0.19*	0.23*	0.27*	0.19*	0.42*	0.17*
Baculoviral IAP repeat-containing protein 2	BIRC2	0.41*	0.58*	0.49*	0.57*	1.37	1.08	1.31
Keratin, type II cytoskeletal 1	KRT1	0.42*	0.49*	0.57*	1.10	1.59*	0.44*	1.02
Uncharacterized protein C2orf42	C2orf42	0.42*	0.67*	0.53*	0.45*	0.41*	0.45*	0.28*
Mitotic deacetylase-associated SANT domain protein	MIDEAS	0.42*	0.58*	0.54*	0.52*	0.31*	0.47*	0.43*
General transcription factor 3C polypeptide 1	GTF3C1	0.42*	0.44*	0.37*	0.48*	0.68	0.68	0.43*
E3 ubiquitin-protein ligase NRDP1	RNF41	0.43*	0.55*	0.45*	0.33*	0.81	0.61	0.52
Circadian locomoter output cycles protein kaput	CLOCK	0.43*	0.48*	0.56*	0.34*	0.60	0.53*	0.46*
Transducin beta-like protein 3	TBL3	0.43*	0.52*	0.55*	0.64*	0.60	0.68	0.74
Bromodomain adjacent to zinc finger domain protein 1A	BAZ1A	0.43*	0.62*	0.56*	0.43*	0.58	0.43*	0.43*
Zinc finger protein 701	ZNF701	0.43*	0.34*	0.36*	0.45*	0.46*	0.42*	0.48*
Protein Wiz	WIZ	0.43*	0.61*	0.61*	0.58*	0.15*	0.17*	0.32*
Mitogen-activated protein kinase kinase kinase 20	MAP3K20	0.44*	0.46*	0.61*	0.40*	0.79	0.65	0.61*
Translation machinery-associated protein 7	TMA7	0.44*	0.46*	0.65*	0.80	0.44*	0.40*	0.71
WD repeat-containing protein 37	WDR37	0.44*	0.58*	0.50*	0.48*	0.72	0.69	0.64

Putative ATP-dependent RNA helicase DHX57	DHX57	0.45*	0.47*	0.46*	0.30*	0.71	0.63	0.46*
Sin3 histone deacetylase corepressor complex component SDS3	SUDS3	0.45*	0.57*	0.52*	0.61*	0.92	0.93	0.83
Methylosome subunit pICln	CLNS1A	0.45*	0.50*	0.51*	0.84	0.84	0.80	1.06
Transcription initiation factor TFIID subunit 5	TAF5	0.45*	0.59*	0.49*	0.70	1.60	1.45	1.68*
Caspase-14	CASP14	0.45*	0.59*	0.53*	1.31*	0.79	0.78	1.12
Serine/threonine-protein phosphatase 6 regulatory ankyrin repeat subunit A	ANKRD28	0.46*	0.44*	0.46*	0.44*	0.67	0.55	0.51*
Inactive ubiquitin carboxyl-terminal hydrolase 53	USP53	0.46*	0.63*	0.51*	0.31*	0.32*	0.36*	0.22*
Keratin, type I cytoskeletal 9	KRT9	0.46*	0.44*	0.46*	1.11	1.99*	0.51*	0.89
Structural maintenance of chromosomes protein 4	SMC4	0.46*	0.45*	0.55*	0.46*	1.22	1.09	0.96
Integrator complex subunit 7	INTS7	0.46*	0.58*	0.57*	0.49*	1.12	0.88	0.83
Ubiquitin-associated and SH3 domain-containing protein B	UBASH3B	0.46*	0.57*	0.63*	0.43*	0.52*	0.51*	0.44*
NFX1-type zinc finger-containing protein 1	ZNFX1	0.46*	0.56*	0.59*	0.49*	0.55*	0.63	0.56*
Zinc finger protein 22	ZNF22	0.46*	0.37*	0.55*	0.77	0.49*	0.35*	0.36*
E3 ubiquitin-protein ligase MSL2	MSL2	0.47*	0.40*	0.51*	2.74*	11.31*	10.04*	11.22*
ATPase family gene 2 protein homolog B	AFG2B	0.47*	0.50*	0.62*	0.54*	0.54	0.54	0.60
RRP12-like protein	RRP12	0.47*	0.46*	0.54*	0.47*	0.45*	0.46*	0.49*
Lysine-specific demethylase 9	RSBN1	0.47*	0.57*	0.46*	0.83	0.08*	0.42*	0.30*
Neurabin-1	PPP1R9A	0.47*	0.58*	0.64*	0.46*	0.49*	0.39*	0.47*
Alpha-endosulfine	ENSA	0.47*	0.44*	0.55*	0.53*	0.36*	0.35*	0.55*
Mismatch repair endonuclease PMS2	PMS2	0.47*	0.47*	0.58*	0.54*	1.06	0.65	0.77
Mitogen-activated protein kinase 7	MAPK7	0.47*	0.62*	0.67*	0.45*	0.68	0.63	0.63
Probable JmjC domain-containing histone demethylation protein 2C	JMJD1C	0.47*	0.42*	0.33*	0.50*	0.72	0.42*	0.29*
Calcium-binding and coiled-coil domain-containing protein 2	CALCOCO2	0.47*	0.60*	0.63*	0.55*	0.31*	0.29*	0.33*
DNA (cytosine-5)-methyltransferase 1	DNMT1	0.48*	0.32*	0.41*	0.70	3.00*	2.01*	2.38*
Loricrin	LORICRIN	0.48*	0.44*	0.64*	1.79*	1.63*	0.30*	1.10
RING finger and CHY zinc finger domain-containing protein 1	RCHY1	0.48*	0.63*	0.65*	0.35*	0.05*	0.12*	0.05*
Probable ATP-dependent RNA helicase DDX56	DDX56	0.48*	0.51*	0.57*	0.42*	0.34*	0.34*	0.30*
Cytosolic iron-sulfur assembly component 3	CIAO3	0.48*	0.47*	0.55*	0.58*	0.69	0.48*	0.58*
Keratin, type I cytoskeletal 27	KRT27	0.48*	0.64	0.52*	1.06	0.68	0.48*	1.03
Zinc finger CCCH domain-containing protein 8	ZC3H8	0.48*	0.52*	0.58*	0.44*	0.51*	0.43*	0.45*
DNA-directed RNA polymerase III subunit RPC1	POLR3A	0.49*	0.56*	0.66	0.97	1.00	0.97	1.20
DmX-like protein 2	DMXL2	0.50*	0.46*	0.41*	0.44*	0.53*	0.52*	0.44*
Vam6/Vps39-like protein	VPS39	0.50*	0.46*	0.46*	0.37*	0.63	0.54*	0.58*

TATA-binding protein-associated factor 172	BTAF1	0.50*	0.62*	0.58*	0.51*	0.58	0.54	0.52*
NF-X1-type zinc finger protein NFXL1	NFXL1	0.50*	0.58*	0.58*	0.40*	0.39*	0.35*	0.40*
Macrophage mannose receptor 1	MRC1	0.51*	0.40*	0.41*	1.72*	6.02*	7.32*	7.08*
Replication factor C subunit 5	RFC5	0.51*	0.49*	0.62*	0.29*	0.55*	0.70	0.43*
Ribonuclease P protein subunit p29	POP4	0.51*	0.61*	0.62*	0.63*	1.31	1.21	1.20
E3 ubiquitin-protein ligase RNF113A	RNF113A	0.51*	0.59*	0.63*	0.48*	0.18*	0.21*	0.31*
Ephrin type-B receptor 3	EPHB3	0.51*	0.62*	0.60*	1.22	4.74*	3.26*	4.09*
Keratin, type I cytoskeletal 14	KRT14	0.51*	0.49*	0.57*	1.84*	0.71*	0.52*	0.61*
TNF receptor-associated factor 2	TRAF2	0.52*	0.60*	0.58*	0.57*	0.95	0.93	0.89
AN1-type zinc finger protein 1	ZFAND1	0.53*	0.50*	0.58*	0.40*	0.41*	0.34*	0.71
Kinetochore-associated protein NSL1 homolog	NSL1	0.53*	0.63*	0.65*	0.56*	0.45*	0.26*	0.50*
SUN domain-containing protein 1	SUN1	0.53*	0.64*	0.58*	0.57*	0.65	0.69	0.49*
BTB/POZ domain-containing protein KCTD3	KCTD3	0.53*	0.58*	0.51*	0.54*	0.97	1.07	0.69
DNA replication licensing factor MCM5	MCM5	0.54*	0.55*	0.65*	0.72	0.60	0.67	1.03
Remodeling and spacing factor 1	RSF1	0.54*	0.63*	0.57*	0.64*	0.40*	0.41*	0.53*
E3 ubiquitin-protein ligase TRIM32	TRIM32	0.54*	0.56*	0.56*	0.48*	0.95	0.87	0.72
Desmocollin-1	DSC1	0.54*	0.35*	0.53*	0.79	1.12	0.55*	0.56*
Guanine nucleotide-binding protein subunit beta-like protein 1	GNB1L	0.54*	0.61*	0.64*	0.48*	0.50*	0.58	0.57
Structural maintenance of chromosomes protein 2	SMC2	0.55*	0.44*	0.54*	0.42*	0.65	0.74	0.48*
tRNA (uracil-5-)-methyltransferase homolog A	TRMT2A	0.55*	0.50*	0.52*	0.55*	0.58	0.55*	0.62
Thrombomodulin	THBD	0.55*	0.62*	0.60*	1.70*	7.47*	6.68*	7.22*
Serine/threonine-protein kinase Chk2	CHEK2	0.56*	0.63*	0.61*	0.38*	0.31*	0.25*	0.25*
ATP-dependent RNA helicase DDX24	DDX24	0.56*	0.55*	0.62*	0.81	0.51*	0.51*	0.67*
U3 small nucleolar RNA-associated protein 6 homolog	UTP6	0.56*	0.49*	0.59*	0.67	0.68	0.68	0.75
Non-structural maintenance of chromosomes element 3 homolog	NSMCE3	0.56*	0.62*	0.61*	0.60*	0.87	0.88	0.88
Vacuolar protein sorting-associated protein 18 homolog	VPS18	0.56*	0.56*	0.55*	0.49*	0.67*	0.70	0.59*
Zinc finger CCHC domain-containing protein 8	ZCCHC8	0.56*	0.55*	0.56*	0.60*	0.26*	0.25*	0.39*
Peroxisomal ATPase PEX1	PEX1	0.56*	0.64*	0.64*	0.39*	0.43*	0.48*	0.31*
Ribosomal RNA-processing protein 7 homolog A	RRP7A	0.57*	0.49*	0.52*	0.47*	0.39*	0.41*	0.46*
Zinc finger and BTB domain-containing protein 7A	ZBTB7A	0.57*	0.54*	0.56*	0.56*	0.52*	0.45*	0.56*
Serine/threonine-protein kinase MRCK alpha	CDC42BPA	0.58*	0.63*	0.57*	0.52*	0.64	0.58*	0.40*
Probable ribosome biogenesis protein RLP24	RSL24D1	0.58*	0.47*	0.54*	0.50*	0.22*	0.21*	0.27*
Condensin complex subunit 3	NCAPG	0.58*	0.53*	0.54*	0.45*	0.97	0.80	0.67
Epithelial splicing regulatory protein 2	ESRP2	0.58*	0.58*	0.66*	0.42*	0.50*	0.38*	0.45*
E3 ubiquitin-protein ligase makorin-2	MKRN2	0.59*	0.67	0.65*	0.42*	0.60	0.52*	0.45*

Dynamin-binding protein	DNMBP	0.59*	0.48*	0.50*	0.52*	0.66	0.52	0.46*
Replication factor C subunit 2	RFC2	0.59*	0.62*	0.63*	0.34*	0.52*	0.54*	0.44*
Transmembrane channel-like protein 6	TMC6	0.59*	0.47*	0.51*	0.62*	0.63	0.64	0.61
Tetratricopeptide repeat protein 7A	TTC7A	0.60*	0.57*	0.61*	0.46*	0.66	0.77	0.73
DNA mismatch repair protein Msh2	MSH2	0.60*	0.57*	0.67*	0.70	0.66	0.64	0.75
S1 RNA-binding domain-containing protein 1	SRBD1	0.60*	0.53*	0.64*	0.43*	0.58	0.40*	0.23*
WD repeat-containing protein 36	WDR36	0.60*	0.52*	0.56*	0.76	0.69	0.62	0.71
U3 small nucleolar RNA-associated protein 4 homolog	UTP4	0.61*	0.58*	0.61*	0.80	1.19	1.21	1.14
Phosphoinositide 3-kinase regulatory subunit 4	PIK3R4	0.61*	0.65*	0.59*	0.64*	0.72	0.62	0.58
3'-5' RNA helicase YTHDC2	YTHDC2	0.61*	0.59*	0.64*	0.82	0.72	0.60*	0.78
Protein AATF	AATF	0.61*	0.52*	0.65*	0.82	0.54*	0.54*	0.65
TRAF family member-associated NF-kappa-B activator	TANK	0.61*	0.64	0.66*	0.91	0.50*	0.50*	0.55
Transcriptional adapter 1	TADA1	0.62*	0.65*	0.58*	0.95	1.19	1.06	1.29
E3 ubiquitin-protein ligase MIB1	MIB1	0.62*	0.64*	0.55*	0.69	0.71	0.56	0.72
Gap junction beta-1 protein	GJB1	0.62*	0.56*	0.56*	0.75	0.66	0.53*	0.58*
Translation initiation factor eIF-2B subunit delta	EIF2B4	0.62*	0.59*	0.55*	0.64*	0.57*	0.57*	0.61*
DNA polymerase delta catalytic subunit	POLD1	0.62*	0.55*	0.53*	0.73	0.61	0.62	0.85
E3 ubiquitin-protein ligase BRE1B	RNF40	0.62*	0.66*	0.61*	0.62*	0.57*	0.49*	0.64
ATPase WRNIP1	WRNIP1	0.62*	0.60*	0.63*	0.35*	0.39*	0.35*	0.38*
A-kinase anchor protein 8	AKAP8	0.63*	0.53*	0.59*	0.50*	0.42*	0.47*	0.67
Tetratricopeptide repeat protein 12	TTC12	0.64*	0.64*	0.64*	0.57*	0.54*	0.51*	0.44*
Syndecan-1	SDC1	0.64*	0.52*	0.65*	0.81	0.67	0.59	0.73
Activity-dependent neuroprotector homeobox protein	ADNP	0.65*	0.65*	0.67*	0.68*	0.77	0.72*	0.86
Probable dimethyladenosine transferase	DIMT1	0.65*	0.59*	0.64*	0.70	0.59	0.75	0.90
Tubulin alpha chain-like 3	TUBAL3	0.66*	0.67*	0.56*	0.39*	0.33*	0.45*	0.17*
Nucleolar protein 6	NOL6	0.67*	0.57*	0.61*	0.81	0.75	0.64	0.74

B. Down-regulated proteins unique to Aquamin (AQ) [243 proteins]

		Interventions						
		Control			*With LPS & Cytokines*			
Proteins	Genes	**AQ**	AQ+MES	MES	*LPS-Cyto*	*AQ*	*AQ+MES*	*MES*
Reticulocalbin-3	RCN3	0.17*	0.72	0.78	1.01	3.31*	4.57*	5.68*
Carboxypeptidase B2	CPB2	0.23*	1.02	1.24	1.76*	5.38*	5.30*	10.11*
Collectin-11	COLEC11	0.28*	0.79	1.14	1.30	2.59*	3.59*	5.27*
Mimecan	OGN	0.29*	0.85	0.94	1.34	3.21*	4.58*	5.97*
HLA class II histocompatibility antigen, DP beta 1 chain	HLA-DPB1	0.31*	2.33*	1.80*	15.14*	13.55*	15.68*	15.01*
Dihydropyrimidinase	DPYS	0.33*	0.71	0.75	1.13	2.82*	3.76*	4.94*

Protein S100-A7	S100A7	0.33*	0.70	0.94	1.71*	0.60	1.01	1.30
Rho guanine nucleotide exchange factor 40	ARHGEF40	0.35*	0.74	1.05	0.31*	0.49*	0.88	0.97
Fibrinogen gamma chain	FGG	0.35*	1.28	2.38*	2.58*	3.47*	3.42*	6.21*
Olfactomedin-like protein 3	OLFML3	0.35*	1.30	0.87	1.59*	3.28*	5.46*	7.10*
Ankyrin repeat domain-containing protein SOWAHB	SOWAHB	0.35*	0.73	0.67	0.39*	0.53	0.48*	0.46*
Mucin-like protein 1	MUCL1	0.37*	0.77	0.72	1.23	0.90	0.72	1.47
Vasorin	VASN	0.39*	0.96	0.86	1.44	2.45*	3.55*	5.06*
Zinc finger protein 536	ZNF536	0.40*	1.44	1.10	1.81*	2.08*	2.70*	5.62*
ABC-type oligopeptide transporter ABCB9	ABCB9	0.40*	0.67	1.05	1.77*	0.59	0.93	2.42*
Suprabasin	SBSN	0.41*	0.70*	0.75*	1.09	0.96	0.80	1.12
Cerebellin-4	CBLN4	0.41*	1.58	2.52*	0.57*	0.78	1.73	1.95*
Coagulation factor X	F10	0.41*	0.77	0.81	1.13	3.39*	5.07*	5.60*
RAB6A-GEF complex partner protein 2	RGP1	0.41*	0.67	0.93	0.65	0.92	1.04	0.97
Coiled-coil domain-containing protein 39	CCDC39	0.41*	0.77	1.31	2.33*	2.48*	2.44*	2.90*
Microtubule-associated protein 1B	MAP1B	0.42*	0.77	0.85	1.20	2.79*	2.86*	3.79*
Protein BCAP	ODF2L	0.43*	1.24	1.79*	2.23*	2.03*	2.99*	3.70*
Programmed cell death 1 ligand 1	CD274	0.43*	1.53	1.44	16.94*	16.35*	19.58*	15.89*
Cdc42 effector protein 5	CDC42EP5	0.44*	0.80	0.94	1.05	0.88	0.85	0.47*
Ribosomal protein eL22-like	RPL22L1	0.45*	0.89	1.44	0.86	0.54	1.05	1.90*
Coiled-coil domain-containing protein 91	CCDC91	0.46*	0.97	0.95	0.49*	0.54*	0.49*	0.49*
Neural proliferation differentiation and control protein 1	NPDC1	0.46*	0.76	0.88	0.47*	0.26*	0.24*	0.25*
Carbamoyl-phosphate synthase [ammonia], mitochondrial	CPS1	0.47*	1.17	0.96	1.52*	4.01*	4.48*	5.28*
Protocadherin-12	PCDH12	0.47*	1.35	1.06	1.79*	2.87*	4.80*	6.92*
Kininogen-1	KNG1	0.47*	2.27*	2.73*	1.71*	0.68	2.29*	2.32*
Protein FAM117B	FAM117B	0.47*	0.87	0.70	0.60*	0.12*	0.28*	0.71
Ral GTPase-activating protein subunit alpha-2	RALGAPA2	0.47*	0.70	0.69*	0.52*	0.48*	0.38*	0.37*
Protein S100-A8	S100A8	0.48*	1.04	0.90	1.09	1.06	1.27	1.27
Nucleus accumbens-associated protein 1	NACC1	0.48*	0.68*	0.69*	0.50*	0.31*	0.32*	0.34*
Inter-alpha-trypsin inhibitor heavy chain H1	ITIH1	0.48*	0.96	1.54*	0.48*	0.48*	1.13	1.53
Integrin alpha-7	ITGA7	0.48*	0.78	0.74	5.21*	0.84	0.49*	1.76
Fermitin family homolog 3	FERMT3	0.48*	1.02	0.99	1.11	2.94*	4.31*	4.74*
C4b-binding protein alpha chain	C4BPA	0.48*	1.00	1.41	1.50*	1.06	1.25	1.68
Rootletin	CROCC	0.49*	1.51*	0.96	0.74	0.50*	0.53*	0.43*
Adenylyl cyclase-associated protein 2	CAP2	0.49*	1.23	1.16	1.51*	3.15*	3.71*	4.96*
Lysine-specific demethylase 2A	KDM2A	0.49*	0.82	0.77	0.73	0.44*	0.45*	0.70
Keratin, type II cytoskeletal 6A	KRT6A	0.49*	1.00	1.23	1.61*	0.93	1.36	1.29
Smad nuclear-interacting protein 1	SNIP1	0.49*	0.69	0.71	0.53*	0.69	0.66	0.48*

Protein Wnt-3a	WNT3A	0.49*	0.92	0.90	1.17	3.24*	4.10*	5.41*
Protein bassoon	BSN	0.50*	2.53*	1.96*	5.37*	19.19*	17.24*	29.63*
Sex hormone-binding globulin	SHBG	0.50*	2.02*	2.50*	0.70	0.75	1.95*	2.89*
Collagen alpha-1(IV) chain	COL4A1	0.50*	1.17	1.70*	1.82*	2.23*	2.22*	3.55*
Beta-enolase	ENO3	0.50*	0.75	0.76*	1.95*	5.84*	4.93*	4.93*
Apolipoprotein M	APOM	0.51*	0.86	1.24*	1.56*	2.35*	3.87*	5.61*
C-type lectin domain family 11 member A	CLEC11A	0.51*	1.23	1.22	1.20	2.68*	3.66*	4.12*
DNA-binding protein SATB2	SATB2	0.51*	0.92	0.75*	0.48*	0.45*	0.42*	0.63*
Protein unc-93 homolog A	UNC93A	0.51*	1.04	1.49	2.62*	1.95*	2.34*	3.58*
Dynein axonemal heavy chain 1	DNAH1	0.51*	1.54*	2.15*	1.10	0.62*	2.04*	2.51*
Afamin	AFM	0.52*	2.16*	2.43*	0.71*	0.67*	2.00*	2.67*
Myomegalin	PDE4DIP	0.52*	0.90	0.94	0.87	0.30*	0.31*	0.60*
Ferritin light chain	FTL	0.52*	2.72*	3.90*	1.95*	0.58	4.12*	9.36*
Claudin-1	CLDN1	0.52*	0.82	1.07	0.92	0.85	0.65	1.06
Collagen alpha-1(XI) chain	COL11A1	0.53*	2.32*	2.75*	0.87	0.94	2.20*	3.10*
CDK-activating kinase assembly factor MAT1	MNAT1	0.53*	0.93	1.05	0.79	0.52	0.89	1.51
Tensin-3	TNS3	0.53*	0.80	0.74*	0.43*	0.72	0.61	0.47*
Collagen alpha-2(I) chain	COL1A2	0.53*	2.15*	2.47*	0.72	0.65	1.88*	2.71*
Coiled-coil domain-containing protein 9B	CCDC9B	0.53*	0.96	1.03	0.55*	0.41*	0.73	0.79
AN1-type zinc finger protein 6	ZFAND6	0.53*	0.75*	0.85	0.35*	0.06*	0.11*	0.22*
Probable phosphoglycerate mutase 4	PGAM4	0.53*	1.15	2.54*	0.51*	0.63	1.94*	2.77*
Vitronectin	VTN	0.53*	1.33	1.51*	1.58*	1.55	1.93*	2.76*
DEP domain-containing mTOR-interacting protein	DEPTOR	0.53*	0.74	0.75*	0.26*	0.46*	0.39*	0.27*
Ubiquitin-associated protein 2	UBAP2	0.54*	0.90	1.00	0.59*	0.07*	0.16*	0.10*
Vascular cell adhesion protein 1	VCAM1	0.54*	1.13	1.26	2.04*	3.97*	5.56*	6.73*
Protocadherin gamma-C3	PCDHGC3	0.54*	1.39	1.37*	1.48*	2.77*	3.66*	5.21*
FERM and PDZ domain-containing protein 1	FRMPD1	0.54*	2.61*	2.58*	0.47*	0.94	1.85*	2.22*
Laminin subunit beta-1	LAMB1	0.54*	2.04*	1.65*	1.58*	2.39*	2.83*	3.78*
BAH and coiled-coil domain-containing protein 1	BAHCC1	0.54*	1.51*	0.94	0.43*	0.49*	0.40*	0.22*
Retinoic acid receptor responder protein 2	RARRES2	0.54*	1.61*	2.51*	0.66	0.68	1.46	2.08*
SPARC	SPARC	0.54*	1.40*	1.85*	1.86*	1.88*	3.08*	3.36*
Alpha-fetoprotein	AFP	0.54*	1.16	1.75*	1.41*	1.41*	2.14*	3.27*
Adenosine 3'-phospho 5'-phosphosulfate transporter 2	SLC35B3	0.55*	0.96	0.84	0.96	1.05	0.94	1.05
Rho GTPase-activating protein 42	ARHGAP42	0.55*	0.74	0.71	0.78	0.67	0.97	0.45*
Nuclear factor 1 C-type	NFIC	0.55*	0.74	0.78	1.06	1.84*	1.42	1.30
Nucleolar complex protein 3 homolog	NOC3L	0.55*	0.80	0.98	1.23	0.41*	0.42*	0.82
Laminin subunit alpha-1	LAMA1	0.55*	2.01*	1.66*	1.60*	2.30*	2.88*	3.96*

Thymosin beta-4	TMSB4X	0.55*	0.94	1.71*	1.20	1.01	1.17	1.93*
Guanidinoacetate N-methyltransferase	GAMT	0.55*	1.98*	1.89*	0.67	0.57	1.54	1.94*
Thymosin beta-10	TMSB10	0.55*	0.69	1.12	1.08	0.57	0.67	0.93
Alpha-1B-glycoprotein	A1BG	0.55*	1.99*	2.73*	0.77	0.76	2.05*	3.14*
Gamma-taxilin	TXLNG	0.56*	0.82	0.80	0.73	0.81	0.70	0.86
Myosin light chain 6B	MYL6B	0.56*	0.71*	0.75	0.80	1.66*	1.68*	1.15
Fibrinogen beta chain	FGB	0.56*	2.04*	1.67*	1.92*	3.53*	3.54*	6.17*
ATPase family gene 2 protein homolog A	AFG2A	0.56*	0.72	0.72	0.67	0.78	0.76	0.77
Carboxypeptidase N catalytic chain	CPN1	0.56*	0.92	0.92	1.44*	3.11*	4.54*	5.08*
Endoribonuclease ZC3H12A	ZC3H12A	0.56*	0.69*	0.73*	0.46*	0.80	0.84	0.70
Inter-alpha-trypsin inhibitor heavy chain H4	ITIH4	0.56*	1.42	1.59*	2.09*	1.62*	2.06*	3.18*
Non-histone chromosomal protein HMG-17	HMGN2	0.56*	1.04	1.61*	0.87	0.80	1.21	1.52
Phospholipid scramblase 1	PLSCR1	0.56*	1.04	1.09	1.60*	0.94	1.55	1.76*
Protein piccolo	PCLO	0.56*	1.75*	2.05*	0.55*	0.72	2.03*	2.36*
Sphingomyelin phosphodiesterase 2	SMPD2	0.56*	1.01	1.40	0.73	0.70	1.09	0.90
Tripartite motif-containing protein 26	TRIM26	0.56*	0.73*	0.71*	0.63*	0.53*	0.56*	0.62*
Dynamin-1	DNM1	0.57*	1.02	0.79	1.22	2.61*	1.81*	2.14*
Pigment epithelium-derived factor	SERPINF1	0.57*	1.24	1.90*	1.24	0.96	2.19*	3.38*
Pregnancy zone protein	PZP	0.57*	1.21	1.00	1.42	0.69	1.38	1.86
Keratin, type I cytoskeletal 16	KRT16	0.57*	0.74	0.81	1.57*	0.80	0.85	1.03
Protein polybromo-1	PBRM1	0.57*	0.81	0.74*	0.47*	0.32*	0.33*	0.27*
Deoxynucleotidyltransferase terminal-interacting protein 2	DNTTIP2	0.57*	0.77	0.76	0.51*	0.67	0.67	0.56*
Phosphoinositide 3-kinase adapter protein 1	PIK3AP1	0.57*	0.81	0.75	0.97	1.01	0.90	0.93
Cartilage oligomeric matrix protein	COMP	0.57*	0.84	1.22	1.73*	2.91*	4.29*	6.18*
Abl interactor 2	ABI2	0.57*	0.79	0.83	0.91	1.11	0.97	1.21
Retinoblastoma-associated protein	RB1	0.57*	0.73	0.82	0.71	1.02	1.15	0.84
AMP deaminase 3	AMPD3	0.58*	0.83	0.81	0.65	0.64	0.73	0.77
Cerebellar degeneration-related protein 2	CDR2	0.58*	0.93	0.86	0.81	1.26	0.91	1.09
Cytosolic carboxypeptidase 1	AGTPBP1	0.58*	0.73	0.86	0.64*	0.62	0.35*	0.67
Splicing regulator ARVCF	ARVCF	0.58*	0.91	0.98	0.40*	0.46*	0.37*	0.29*
BRCA1-associated protein	BRAP	0.58*	0.73	0.74	0.59*	0.33*	0.42*	0.44*
DNA-binding protein SMUBP-2	IGHMBP2	0.58*	1.53*	1.72*	2.47*	3.42*	3.38*	5.07*
E3 ubiquitin-protein ligase TRIM15	TRIM15	0.58*	0.81	0.82	0.72	0.74	0.86	0.75
Endoribonuclease Dicer	DICER1	0.58*	0.75	0.84	0.53*	0.94	0.86	0.85
Inactive tyrosine-protein kinase 7	PTK7	0.59*	0.75	0.86	0.69	1.12	0.98	0.88
Ribosomal protein eL42-like	RPL36AL	0.59*	0.84	0.84	0.70	0.61	0.72	0.65
Plasma kallikrein	KLKB1	0.59*	2.06*	2.86*	0.72	0.82	1.89*	2.72*

Gamma-tubulin complex component 2	TUBGCP2	0.59*	0.71*	0.77*	0.71*	0.92	0.94	0.96
Complement factor D	CFD	0.59*	1.43	3.72*	0.81	0.58	1.54	2.05*
Extracellular sulfatase Sulf-2	SULF2	0.59*	0.93	1.25*	0.86	0.35*	0.37*	0.73*
Replication factor C subunit 4	RFC4	0.59*	0.80	0.92	0.63*	0.81	0.77	0.95
Regucalcin	RGN	0.59*	1.19	1.30	1.08	1.30	1.94*	2.66*
Calmodulin-regulated spectrin-associated protein 1	CAMSAP1	0.59*	0.92	1.06	0.77	0.86	0.61	0.70
Poly(A)-specific ribonuclease PARN	PARN	0.59*	0.68*	0.67*	0.62*	0.66*	0.71	0.71
Alpha-amylase 1B	AMY1B	0.60*	1.01	1.13	1.52*	3.33*	3.94*	5.43*
Protein S100-A9	S100A9	0.60*	1.51*	1.41*	1.53*	1.35	1.39	2.46*
Low-density lipoprotein receptor-related protein 2	LRP2	0.60*	1.46	1.02	1.45	4.10*	5.03*	6.63*
Elongator complex protein 4	ELP4	0.60*	0.73	0.75	0.66	0.60	0.53	0.49*
OTU domain-containing protein 3	OTUD3	0.60*	0.71	0.81	0.39*	0.22*	0.16*	0.43*
Interferon-induced protein 44-like	IFI44L	0.60*	1.47	1.36	1.22	1.34	1.82	3.21*
NADPH oxidase organizer 1	NOXO1	0.60*	0.72	0.81	0.36*	0.61	0.52	0.31*
Protein pelota homolog	PELO	0.60*	0.72*	0.69*	0.59*	0.70	0.70	0.65*
Filaggrin	FLG	0.60*	0.72*	0.83	1.47*	0.97	0.88	1.06
Complement component C8 alpha chain	C8A	0.60*	1.16	1.38*	1.68*	3.50*	4.58*	6.06*
Glucocorticoid modulatory element-binding protein 2	GMEB2	0.60*	0.73	0.76	0.54*	0.68	0.74	0.57
Sex-determining region Y protein	SRY	0.61*	1.19	1.25	1.77*	1.72	1.71	2.48*
Coiled-coil domain-containing protein 85C	CCDC85C	0.61*	0.72*	0.70*	0.39*	0.15*	0.22*	0.24*
Immediate early response 3-interacting protein 1	IER3IP1	0.61*	1.26	1.09	1.05	1.64*	1.16	0.89
von Willebrand factor A domain-containing protein 1	VWA1	0.61*	1.07	1.14	1.04	2.29*	2.51*	3.15*
E3 ubiquitin-protein ligase TRIM68	TRIM68	0.61*	0.90	0.91	0.76	1.24	1.19	0.78
Laminin subunit gamma-1	LAMC1	0.61*	1.97*	1.62*	1.54*	2.28*	2.68*	3.65*
Synaptojanin-2	SYNJ2	0.61*	0.68*	0.76	0.49*	0.41*	0.32*	0.33*
ADP-ribosylation factor-binding protein GGA2	GGA2	0.61*	0.80	0.81	0.40*	0.69	0.44*	0.48*
Interleukin-1 receptor accessory protein	IL1RAP	0.61*	2.35*	2.99*	0.79	0.92	2.31*	3.58*
Coagulation factor V	F5	0.61*	1.22	1.26	1.74*	1.61	1.89*	2.43*
Metallothionein-1H	MT1H	0.61*	0.90	0.77	0.97	0.42*	0.55	0.62
Probable helicase with zinc finger domain	HELZ	0.61*	0.70	0.67*	0.49*	1.11	1.01	1.01
Regulatory-associated protein of mTOR	RPTOR	0.61*	0.70	0.73*	0.69	0.90	0.81	0.91
Histone-lysine N-methyltransferase SETD1A	SETD1A	0.61*	0.70	0.79	0.76	0.60	0.55	0.68
Helicase with zinc finger domain 2	HELZ2	0.61*	0.88	0.77	0.53*	0.36*	0.97	0.57
Alpha-2-macroglobulin	A2M	0.61*	1.48*	1.87*	1.88*	1.77*	2.51*	3.48*
Protein TANC1	TANC1	0.62*	0.71	0.76	0.69	0.87	0.59	0.63
UPF0538 protein C2orf76	C2orf76	0.62*	0.76	0.83	0.77	0.71	0.58	0.73
RalBP1-associated Eps domain-containing protein 2	REPS2	0.62*	0.72	0.76	0.43*	0.43*	0.24*	0.25*

Kinesin-like protein KIF13A	KIF13A	0.62*	0.77	0.74*	0.69	0.66	0.63	0.66
Vacuolar protein sorting-associated protein 72 homolog	VPS72	0.62*	0.73	0.82	0.28*	0.92	0.17*	0.70
EGF-containing fibulin-like extracellular matrix protein 1	EFEMP1	0.62*	2.26*	2.51*	0.81	0.53*	1.88*	2.37*
Insulin-like growth factor II	IGF2	0.62*	2.07*	2.04*	0.74	0.48*	1.65*	2.22*
CCHC-type zinc finger nucleic acid binding protein	CNBP	0.62*	0.99	0.95	1.03	0.55*	0.93	1.04
Cyclin-dependent kinase 1	CDK1	0.62*	0.67*	0.68*	0.40*	1.51	1.34	1.54*
Collectin-10	COLEC10	0.62*	1.16	1.14	1.23	2.63*	3.78*	4.85*
Complement factor H	CFH	0.62*	2.03*	27.05*	0.74	0.62	1.68*	1.72*
Low-density lipoprotein receptor-related protein 5	LRP5	0.62*	1.06	0.81	0.61*	0.55	0.72	0.76
WD repeat-containing protein 26	WDR26	0.62*	0.73	0.89	1.06	1.12	1.18	1.38
Plasminogen	PLG	0.62	1.31	1.39	1.44	1.62	1.48	1.84
Hepatocyte growth factor activator	HGFAC	0.62*	2.13*	2.48*	0.77*	0.77	2.27*	3.01*
CCR4-NOT transcription complex subunit 6	CNOT6	0.63*	0.77	0.73	1.15	2.38*	1.85*	2.10*
UDP-N-acetylglucosamine transferase subunit ALG14 homolog	ALG14	0.63	0.81	1.10	1.15	1.16	0.88	0.64
Spectrin beta chain, non-erythrocytic 2	SPTBN2	0.63*	0.94	1.28	0.86	0.84	1.25	1.43
Ceruloplasmin	CP	0.63*	1.04	1.10	1.39*	3.14*	3.98*	4.43*
Immunoglobulin kappa variable 4-1	IGKV4-1	0.63*	2.29*	37.17*	0.69	0.85	1.24	0.95
Serum amyloid A-4 protein	SAA4	0.63*	2.09*	2.47*	3.32*	9.01*	8.67*	16.73*
Vinexin	SORBS3	0.63*	1.02	0.97	0.84	0.76	0.96	1.01
Amphiregulin	AREG	0.63	1.46	1.11	1.32	1.51	1.25	3.02*
Mitochondrial ribosome-associated GTPase 2	MTG2	0.63*	0.83	0.94	0.26*	0.29*	0.28*	0.25*
Albumin	ALB	0.63*	1.73*	2.02*	2.42*	1.96*	2.70*	2.09*
Ubiquitin carboxyl-terminal hydrolase 3	USP3	0.63*	0.71	0.70	0.52*	0.67	0.41*	0.28*
Proto-oncogene c-Rel	REL	0.63*	0.88	0.79	1.17	1.50	0.99	1.70*
Phosphatidylinositol 3,4,5-trisphosphate 5-phosphatase 2	INPPL1	0.63*	0.89	0.77	0.77	0.97	0.98	1.27
RNA polymerase II subunit A C-terminal domain phosphatase	CTDP1	0.63*	0.67*	0.70*	0.62*	0.63	0.55*	0.62
Fibrinogen alpha chain	FGA	0.63*	0.68	2.55*	2.12*	0.76	1.05	2.43*
Immunoglobulin heavy constant gamma 4	IGHG4	0.64*	1.95*	32.94*	0.66*	0.72	1.67*	0.96
E3 ubiquitin-protein ligase RING2	RNF2	0.64*	0.81	0.86	0.85	0.15*	0.24*	0.38*
Metastasis-associated protein MTA3	MTA3	0.64*	0.69	0.82	0.71	0.94	0.80	1.02
Tetraspanin-9	TSPAN9	0.64	0.93	0.79	2.49*	7.29*	7.91*	7.67*
Collagen alpha-2(VI) chain	COL6A2	0.64*	1.24	1.33	1.16	1.60	2.85*	3.58*
Scrapie-responsive protein 1	SCRG1	0.64*	2.21*	2.25*	0.80	0.99	1.82*	2.12*
tRNA (guanine(26)-N(2))-dimethyltransferase	TRMT1	0.64*	0.82	0.82	0.50*	0.50*	0.39*	0.72
Transcriptional repressor p66-alpha	GATAD2A	0.64*	0.72	0.99	0.78	0.50*	0.69	0.85

DNA replication licensing factor MCM3	MCM3	0.64*	0.71	0.84	1.19	0.90	1.07	1.30
Fibromodulin	FMOD	0.64*	2.45*	2.75*	1.06	0.95	2.23*	3.19*
Ral GTPase-activating protein subunit alpha-1	RALGAPA1	0.64*	0.71	0.75	0.76	0.62	0.55	0.61
Chromogranin-A	CHGA	0.64*	5.56*	5.04*	0.86	1.34	4.03*	6.57*
Histone H3-7	H3-7	0.64*	0.78	0.93	0.99	1.01	1.06	1.31
Dermcidin	DCD	0.64*	0.68*	0.85	0.99	1.23	0.95	1.65*
Spectrin beta chain, erythrocytic	SPTB	0.65	0.70	1.24	1.53	3.31*	3.32*	4.60*
Single-stranded DNA-binding protein 4	SSBP4	0.65*	0.96	0.80	0.58*	1.08	1.15	1.07
Overexpressed in colon carcinoma 1 protein	OCC1	0.65*	0.70	0.87	0.63*	0.55*	0.62	0.87
Contactin-1	CNTN1	0.65*	2.25*	2.79*	0.80	0.88	2.29*	3.42*
TBC1 domain family member 8B	TBC1D8B	0.65*	0.82	0.74*	0.61*	0.63	0.67	0.63
Protein SDA1 homolog	SDAD1	0.65*	0.70	0.69*	0.69	0.35*	0.42*	0.47*
Beta-2-syntrophin	SNTB2	0.65*	0.94	0.98	0.71	1.15	1.29	1.33
Deoxynucleoside triphosphate triphosphohydrolase SAMHD1	SAMHD1	0.65	1.18	1.07	1.16	2.07*	1.70	1.86
Pleckstrin homology-like domain family B member 2	PHLDB2	0.65*	0.72	0.85	0.71	0.85	0.74	0.70
Hemopexin	HPX	0.65*	1.28	1.58*	1.68*	1.54	1.99*	3.50*
Synergin gamma	SYNRG	0.65*	0.69	0.84	0.56*	0.42*	0.37*	0.55
BMP-binding endothelial regulator protein	BMPER	0.65	1.20	1.13	1.57*	3.53*	3.94*	4.84*
Cell adhesion molecule 1	CADM1	0.65*	1.89*	2.04*	0.63	0.89	1.76*	2.25*
Coiled-coil domain-containing protein 50	CCDC50	0.65*	0.81	0.99	1.02	0.62	0.67	1.29
Zinc finger CCCH domain-containing protein 15	ZC3H15	0.65*	0.74*	0.80*	0.74*	0.67*	0.67	0.80
Homologous recombination OB-fold protein	HROB	0.65*	1.35*	1.48*	1.73*	1.51*	2.16*	1.96*
Pro-glucagon	GCG	0.65*	1.31*	1.64*	0.78	0.99	0.72	0.86
Phosphatidylcholine-sterol acyltransferase	LCAT	0.65*	2.10*	2.67*	0.76	0.94	2.06*	2.94*
TBC domain-containing protein kinase-like protein	TBCK	0.65*	0.69	0.71	0.71	0.87	0.74	0.79
Gap junction beta-2 protein	GJB2	0.66	0.71	0.82	0.91	1.23	1.20	1.26
DNA mismatch repair protein Msh3	MSH3	0.66*	0.67*	0.70*	0.60*	0.66	0.65	0.69
CD5 antigen-like	CD5L	0.66	1.53	23.17*	0.77	0.69	0.82	0.29*
Serine/arginine repetitive matrix protein 2	SRRM2	0.66*	0.89	1.08	0.93	0.71	0.87	1.25
Midasin	MDN1	0.66*	1.29	1.56*	1.48*	1.53*	2.03*	2.59*
BRCA2 and CDKN1A-interacting protein	BCCIP	0.66*	0.96	0.88	1.09	0.82	1.09	1.09
Transcription factor 20	TCF20	0.66*	0.99	1.00	2.22*	0.80	0.51	1.59
Guanine nucleotide-binding protein-like 3	GNL3	0.66*	0.68	0.79	0.55*	0.56	0.46*	0.47*
MAP kinase-activated protein kinase 5	MAPKAPK5	0.66*	0.99	0.88	0.49*	0.63	0.44*	0.45*
LIM and cysteine-rich domains protein 1	LMCD1	0.66	0.81	0.92	1.00	0.74	0.76	0.91
Immunoglobulin lambda constant 2	IGLC2	0.66*	1.33	4.95*	1.42	3.25*	2.14*	1.55

E3 ubiquitin-protein ligase SH3RF1	SH3RF1	0.66	0.85	0.79	0.70	0.69	0.55	0.45*
Actin filament-associated protein 1	AFAP1	0.66	1.00	0.86	0.61*	0.41*	0.40*	0.55
Immortalization up-regulated protein	IMUP	0.66*	1.26	1.81*	1.60*	1.16	1.10	1.75*
Mixed lineage kinase domain-like protein	MLKL	0.66	1.00	0.98	1.10	0.76	1.01	1.45
Calmodulin-like protein 5	CALML5	0.66	0.73	1.08	1.49	0.89	0.91	1.44
Periostin	POSTN	0.66*	1.63*	1.51*	1.02	2.43*	2.54*	3.38*
Dual specificity tyrosine-phosphorylation-regulated kinase 1A	DYRK1A	0.66*	0.71*	0.76*	0.82	1.55*	1.35	1.36
Protein MAK16 homolog	MAK16	0.66*	0.74	0.78	0.81	1.18	1.12	1.31
EF-hand domain-containing protein D1	EFHD1	0.66	43.85*	1.91*	0.69	0.69	0.84	3.11*
Keratin, type II cuticular Hb5	KRT85	0.66*	8.93*	1.06	0.69*	0.55*	0.40*	1.22
Histone H2B type 1-B	H2BC3	0.66*	0.83	0.98	1.05	0.58	0.63	0.97
Inter-alpha-trypsin inhibitor heavy chain H3	ITIH3	0.66*	1.15	1.11	1.68*	3.30*	4.88*	6.20*
Diacylglycerol kinase theta	DGKQ	0.66	1.19	0.97	0.63*	0.46*	0.64	0.53*
Thrombospondin-4	THBS4	0.67*	1.01	1.08	1.82*	3.14*	4.76*	6.66*
Desmoglein-1	DSG1	0.67	0.82	0.96	1.11	1.13	0.69	1.18
Muskelin	MKLN1	0.67*	0.77	0.73	0.79	1.11	1.17	1.24
Steroid hormone receptor ERR1	ESRRA	0.67*	0.77	0.76	0.66	0.67	0.61	0.61
Retinol-binding protein 4	RBP4	0.67*	2.60*	2.77*	0.92	0.90	2.14*	2.97*

C. Down-regulated proteins unique to Aquamin plus Mesalamine (AQ+MES) [65 proteins]

		Interventions						
		Control			*With LPS & Cytokines*			
Proteins	Genes	AQ	**AQ+MES**	MES	*LPS-Cyto*	*AQ*	*AQ+MES*	*MES*
HEAT repeat-containing protein 3	HEATR3	0.74	0.44*	0.85	0.72	0.52	0.37*	0.80
Leucine-rich alpha-2-glycoprotein	LRG1	1.03	0.46*	0.83	0.52*	0.97	0.66	0.54*
Mitogen-activated protein kinase kinase kinase kinase 1	MAP4K1	0.76*	0.46*	1.00	0.70	0.76	0.67	0.69
POTE ankyrin domain family member E	POTEE	1.02	0.49*	0.95	0.59*	0.50*	0.51*	0.90
Phosphatidate phosphatase LPIN3	LPIN3	0.77	0.49*	0.79	0.70	0.65	0.66	1.02
Protein PALS2	PALS2	0.98	0.52*	0.71	0.52*	1.81*	1.54	1.29
Dysferlin	DYSF	0.78*	0.53*	0.82	0.51*	0.36*	0.42*	0.35*
Keratin, type I cytoskeletal 13	KRT13	0.75*	0.53*	0.92	0.69*	0.87	0.64	1.46*
High mobility group protein HMGI-C	HMGA2	0.68	0.54*	0.81	0.74	0.54	0.64	0.71
Junctophilin-1	JPH1	1.14	0.54*	1.41	1.04	0.48*	0.49*	1.10
Monocyte differentiation antigen CD14	CD14	1.13	0.54*	1.02	0.95	1.09	0.63	0.72
Ribosomal protein uL30-like	RPL7L1	0.71*	0.56*	0.76	1.00	1.14	0.94	0.98

Nuclear ubiquitous casein and cyclin-dependent kinase substrate 1	NUCKS1	0.78	0.56*	0.78	0.82	0.70	0.71	0.91
Polyamine deacetylase HDAC10	HDAC10	1.11	0.56*	0.92	0.67	1.07	0.85	0.88
Histone PARylation factor 1	HPF1	1.26	0.56*	0.96	0.94	1.43	1.17	1.03
Regenerating islet-derived protein 4	REG4	0.73*	0.56*	0.98	0.66*	0.45*	0.49*	0.52*
Methyl-CpG-binding domain protein 2	MBD2	0.74	0.56*	0.76	0.69	0.63	0.60	0.68
General transcription factor IIF subunit 1	GTF2F1	0.78	0.57*	0.97	1.27	0.92	0.88	1.24
Exportin-6	XPO6	0.67	0.58*	0.69	0.91	0.66	0.82	0.82
WD repeat-containing protein 74	WDR74	0.74	0.58*	0.71	0.94	1.54	1.63	1.63
Thioredoxin-related transmembrane protein 4	TMX4	0.79	0.58*	0.73	0.70	0.65	0.46*	0.43*
DNA replication licensing factor MCM2	MCM2	0.73	0.58*	0.88	0.81	0.87	1.01	1.49
Nik-related protein kinase	NRK	0.87	0.59*	0.80	1.03	0.57	0.92	1.26
Breakpoint cluster region protein	BCR	0.83	0.60*	0.73	0.72	0.82	0.81	0.66
WD repeat-containing protein 55	WDR55	0.88	0.60*	0.71	0.76	0.50*	0.65	0.79
Non-histone chromosomal protein HMG-14	HMGN1	1.16	0.60*	0.78	3.08*	1.07	1.10	2.25*
Cystathionine beta-synthase	CBS	1.00	0.60*	0.69*	0.83	0.89	0.84	0.84
Integrator complex subunit 1	INTS1	0.96	0.61	0.78	1.00	1.30	1.20	1.14
Nuclear prelamin A recognition factor	NARF	0.85	0.61*	0.79	0.81	0.66	0.61	0.60
WW domain-containing transcription regulator protein 1	WWTR1	0.98	0.61	1.09	0.85	0.74	1.17	0.81
Arf-GAP domain and FG repeat-containing protein 2	AGFG2	0.81	0.62*	0.76	0.61*	0.61	0.45*	0.54
Myelin basic protein	MBP	1.02	0.63	1.44	1.00	0.75	0.72	0.88
Glucose-induced degradation protein 8 homolog	GID8	1.00	0.63*	1.00	1.03	1.02	1.07	1.26
E3 ubiquitin-protein ligase UHRF2	UHRF2	0.79	0.63	1.05	1.05	0.61	0.74	0.85
Probable ATP-dependent RNA helicase DHX37	DHX37	0.71	0.63	0.72	0.77	0.91	1.05	0.96
Sarcoplasmic/endoplasmic reticulum calcium ATPase 3	ATP2A3	1.17	0.63*	0.89	0.69	0.72	0.68	0.62
Histone deacetylase complex subunit SAP30	SAP30	0.72	0.63*	0.71	0.57*	0.95	0.62	0.72
Kinesin-like protein KIF6	KIF6	1.14	0.64	0.99	1.63*	1.91*	2.10*	1.79
Rab-like protein 2A	RABL2A	0.67*	0.64*	0.77	0.32*	0.45*	0.43*	0.28*
GTP-binding protein 4	GTPBP4	0.72*	0.64*	0.86	0.85	0.76	0.74	1.03
Kinesin-like protein KIF1B	KIF1B	0.75	0.64*	0.78	0.67	0.60	0.64	0.56
Multivesicular body subunit 12A	MVB12A	0.94	0.64*	0.98	0.89	0.49*	0.65	0.61
Acid sphingomyelinase-like phosphodiesterase 3b	SMPDL3B	0.89	0.64*	0.68*	0.86	1.05	0.69*	0.68*
Kinesin-like protein KIF23	KIF23	0.95	0.64*	0.70*	0.85	0.70*	0.59*	0.66*
Ras-related protein Rab-13	RAB13	1.05	0.65*	0.98	0.48*	0.46*	0.39*	0.43*
Ribonucleases P/MRP protein subunit POP1	POP1	0.83	0.65*	0.73	0.99	0.71	0.80	1.04
Microtubule-associated protein RP/EB family member 2	MAPRE2	0.71	0.65*	0.80	0.83	0.63	0.66	0.75
Ribosome biogenesis protein BMS1 homolog	BMS1	0.71	0.65	0.98	0.73	0.51*	0.57	0.79

DCC-interacting protein 13-alpha	APPL1	0.87	0.65*	0.89	0.72*	0.67	0.48*	0.61*
Caspase-5	CASP5	1.06	0.65*	0.95	1.37	0.19*	0.37*	0.45*
PEST proteolytic signal-containing nuclear protein	PCNP	0.88	0.65*	0.93	0.78	0.53*	0.64	0.76
Prothymosin alpha	PTMA	0.77	0.65*	1.07	0.59*	0.45*	0.66	1.38*
Serine/threonine-protein kinase A-Raf	ARAF	0.87	0.65*	0.81	0.60*	0.66	0.73	0.87
E3 ubiquitin-protein ligase TRIM22	TRIM22	0.69	0.65	0.73	0.93	0.97	0.99	0.93
Ceramide kinase	CERK	1.07	0.65	0.83	0.99	0.92	0.79	0.69
Rac GTPase-activating protein 1	RACGAP1	0.98	0.66*	0.69*	0.80	0.64*	0.52*	0.57*
IQCJ-SCHIP1 readthrough transcript protein	IQCJ-SCHIP1	0.72	0.66	1.10	0.93	0.89	0.80	1.02
E3 ubiquitin-protein ligase NEDD4	NEDD4	0.78	0.66*	0.85	0.76	1.18	1.35	0.97
Uncharacterized protein C11orf98	C11orf98	1.07	0.66*	0.84	0.91	0.21*	0.21*	0.25*
ATP-dependent RNA helicase DDX3Y	DDX3Y	0.68	0.66	0.87	0.61*	0.51*	0.52*	0.55*
Protein mono-ADP-ribosyltransferase PARP12	PARP12	0.90	0.66	1.04	1.49	1.32	1.35	1.43
Tyrosine-protein kinase BAZ1B	BAZ1B	0.67*	0.66*	0.69*	0.85	0.68	0.63	0.72
Glutamate-rich WD repeat-containing protein 1	GRWD1	0.78	0.67	0.82	0.95	0.75	0.73	0.90
Keratin, type II cytoskeletal 78	KRT78	0.71*	0.67*	1.26*	1.50*	1.92*	0.51*	1.49*
Translation factor GUF1, mitochondrial	GUF1	0.81	0.67*	0.68*	0.67*	0.68	0.81	0.86

D. Down-regulated proteins unique to Mesalamine (MES) [52 proteins]

		Interventions						
		Control			*With LPS & Cytokines*			
Proteins	Genes	AQ	AQ+MES	**MES**	*LPS-Cyto*	*AQ*	*AQ+MES*	*MES*
Zinc transporter ZIP5	SLC39A5	1.47*	0.68	0.34*	1.24	1.57	0.61	0.44*
Transmembrane 4 L6 family member 5	TM4SF5	1.24	1.38	0.43*	0.94	0.47*	0.88	0.52
Intelectin-2	ITLN2	1.04	1.01	0.45*	0.56*	1.13	0.38*	0.42*
Ectopic P granules protein 5 homolog	EPG5	1.07	0.91	0.47*	0.87	0.85	0.69	0.75
Growth arrest-specific protein 6	GAS6	1.31*	0.74	0.52*	1.10	1.23	0.47*	0.54*
Tuberin	TSC2	1.04	1.00	0.53*	1.07	1.27	1.06	1.00
AT-rich interactive domain-containing protein 4A	ARID4A	0.88	0.73	0.53*	1.09	0.88	0.72	0.87
UDP-glucuronosyltransferase 2B15	UGT2B15	0.87	0.69*	0.53*	0.73	0.92	0.62	0.61*
Protein Tob2	TOB2	0.73	0.84	0.54*	0.66	0.35*	0.37*	0.95
Protein angel homolog 2	ANGEL2	0.98	0.89	0.54*	0.99	0.91	0.95	0.63
Dimethylaniline monooxygenase [N-oxide-forming] 4	FMO4	1.07	0.86	0.56*	1.06	0.72	0.70	0.48*
Myelin regulatory factor-like protein	MYRFL	0.96	0.80	0.57*	1.19	0.89	0.77	0.61
Serine incorporator 2	SERINC2	0.90	0.86	0.58*	0.50*	0.89	0.86	0.55*
WD repeat domain phosphoinositide-interacting protein 4	WDR45	0.99	0.70	0.58*	0.84	0.62	0.94	0.79
Zinc finger FYVE domain-containing protein 1	ZFYVE1	1.06	0.74	0.59*	0.76	0.82	0.87	0.67

Ly6/PLAUR domain-containing protein 8	LYPD8	1.39	0.75	0.60*	0.39*	0.50*	0.41*	0.34*
Kallikrein-11	KLK11	1.02	0.71	0.60*	1.03	1.14	0.69	0.68
ADP-ribosylation factor 3	ARF3	0.75	1.05	0.60*	1.25	1.12	1.03	1.12
Deubiquitinating protein VCPIP1	VCPIP1	0.74	0.83	0.60*	0.92	0.99	0.96	1.02
Cytochrome P450 2C19	CYP2C19	2.12*	1.15	0.61*	1.64*	1.98*	0.88	0.62
Transmembrane 7 superfamily member 3	TM7SF3	0.93	0.97	0.61*	1.41*	1.17	1.51	1.23
FAS-associated death domain protein	FADD	0.90	0.79	0.61*	0.96	0.80	0.76	1.03
GTP-binding protein 10	GTPBP10	1.01	0.90	0.62*	0.52*	0.78	0.66*	0.52*
Phosphorylase b kinase gamma catalytic chain, liver/testis isoform	PHKG2	0.73	0.76	0.62*	0.58*	0.34*	0.52	0.30*
Transmembrane 4 L6 family member 4	TM4SF4	0.69	0.68	0.62*	0.67	0.36*	0.58	0.59
Keratin, type II cytoskeletal 1b	KRT77	0.89	0.80	0.63*	1.23	2.31*	0.70	1.26
Kynurenine formamidase	AFMID	0.96	0.74	0.63*	0.86	0.60*	0.58*	0.56*
Cancer-related nucleoside-triphosphatase	NTPCR	0.70*	0.79	0.64*	0.86	0.55*	0.65	0.85
Neutrophil gelatinase-associated lipocalin	LCN2	1.07	0.81	0.64*	1.17	1.23	1.09	0.94
Calcium-activated chloride channel regulator 1	CLCA1	0.89	1.05	0.64*	0.88	1.13	0.71	1.17
TBC1 domain family member 17	TBC1D17	0.73	0.69	0.65*	0.65*	0.98	0.78	0.76
Interferon-induced protein with tetratricopeptide repeats 1	IFIT1	0.75	1.08	0.65*	2.44*	3.27*	3.50*	2.42*
Inositol 1,4,5-trisphosphate receptor type 2	ITPR2	0.94	0.85	0.65*	0.64	0.58	0.66	0.55
Transmembrane 4 L6 family member 20	TM4SF20	1.61*	0.93	0.65*	0.63*	1.51*	0.97	0.50*
Equilibrative nucleoside transporter 3	SLC29A3	1.00	0.90	0.65*	0.97	1.15	0.74	0.89
Pericentriolar material 1 protein	PCM1	0.68*	0.68*	0.65*	0.61*	0.75	0.76	0.69
Mast/stem cell growth factor receptor Kit	KIT	0.70	1.13	0.66*	0.79	3.01*	2.43*	2.74*
5'-AMP-activated protein kinase subunit beta-1	PRKAB1	0.87	0.87	0.66*	0.70	0.87	0.80	0.83
High affinity cGMP-specific 3',5'-cyclic phosphodiesterase 9A	PDE9A	1.41*	1.01	0.66*	1.16	1.37	1.05	0.74
tRNA endonuclease ANKZF1	ANKZF1	0.80	0.74	0.66*	0.81	0.76	0.67	0.70
Protein RUFY3	RUFY3	0.98	0.84	0.66	0.88	0.87	0.76	1.21
Sorting nexin-30	SNX30	1.10	0.73	0.66*	0.80	0.73	0.69	0.56*
Selenoprotein W	SELENOW	1.07	0.68	0.66*	0.86	0.51*	0.45*	0.40*
AP-5 complex subunit beta-1	AP5B1	0.82	0.77	0.66*	0.61*	0.56	0.72	0.69
Serine/threonine-protein kinase Sgk2	SGK2	0.83	0.88	0.66*	0.55*	0.63	0.60*	0.50*
Uncharacterized protein C2orf72	C2orf72	1.13	0.75	0.66*	0.64*	0.05*	0.39*	0.08*
Metabotropic glycine receptor	GPR158	0.90	0.76	0.66*	0.84	0.89	0.81	0.82
Ribonuclease 7	RNASE7	0.79	0.80	0.67*	0.64*	1.10	0.43*	1.35
Carbonic anhydrase 1	CA1	0.97	0.86	0.67*	0.66*	0.70	0.66	0.66*
Cleavage and polyadenylation specificity factor subunit 3	CPSF3	0.70*	0.67*	0.67*	0.70*	0.73	0.74	0.72

Protein FAM107B	FAM107B	0.81	0.92	0.67*	0.64	0.61	0.69	0.69
KICSTOR complex protein ITFG2	ITFG2	0.90	0.77	0.67*	0.92	0.59	0.61	0.81

E. Common down-regulated proteins between Aquamin and Aquamin plus Mesalamine [55 proteins]

		Interventions						
		Control			*With LPS & Cytokines*			
Proteins	Genes	**AQ**	**AQ+MES**	MES	*LPS-Cyto*	*AQ*	*AQ+MES*	*MES*
Protein S100-A7A	S100A7A	0.27*	0.40*	4.36*	0.20*	0.48*	0.17*	0.46*
Keratin, type I cytoskeletal 10	KRT10	0.29*	0.34*	0.79*	0.85	0.85	0.37*	0.57*
Collagen alpha-3(VI) chain	COL6A3	0.29*	0.50*	1.58*	1.31	0.81	1.25	1.92*
Actin filament-associated protein 1-like 2	AFAP1L2	0.38*	0.66	0.77	0.37*	0.13*	0.36*	0.30*
Ubiquitin carboxyl-terminal hydrolase 27	USP27X	0.39*	0.49*	0.71*	0.43*	0.82	0.81	0.63
Transmembrane protein 225B	TMEM225B	0.44*	0.39*	0.80	0.51*	0.70	1.23	0.59
Cytosolic iron-sulfur assembly component 2B	CIAO2B	0.45*	0.60*	1.29	0.47*	0.38*	1.57	1.87
Arginase-1	ARG1	0.45*	0.50*	0.84	0.93	0.87	0.60	0.98
Neurogenic locus notch homolog protein 1	NOTCH1	0.46*	0.54*	0.85	0.49*	0.63	0.65	0.50*
Helicase SRCAP	SRCAP	0.47*	0.38*	0.76	0.62	0.78	0.59	0.85
CDK5 and ABL1 enzyme substrate 1	CABLES1	0.48*	0.56*	0.68*	0.48*	1.04	0.89	0.90
KICSTOR subunit 2	KICS2	0.48*	0.38*	1.09	1.29	0.39*	0.38*	0.78
Coiled-coil domain-containing protein 97	CCDC97	0.49*	0.65	1.06	0.56*	0.43*	0.38*	0.55
Chromobox protein homolog 8	CBX8	0.49*	0.66*	0.71*	0.63*	0.26*	0.20*	0.51*
Uncharacterized protein C9orf85	C9orf85	0.49*	0.66	0.86	0.54*	0.21*	0.17*	0.36*
Small proline-rich protein 2D	SPRR2D	0.49*	0.44*	1.18	2.59*	0.70	0.50*	0.58*
High mobility group protein HMG-I/HMG-Y	HMGA1	0.50*	0.61*	1.13	1.38*	1.08	1.34	1.87*
Nuclear factor 1 A-type	NFIA	0.51*	0.65*	0.67*	0.57*	0.73	0.68	0.60*
Paladin	PALD1	0.51*	0.59*	0.69*	0.60*	0.46*	0.56*	0.54*
eIF-2-alpha kinase GCN2	EIF2AK4	0.51*	0.63	0.70	0.65	0.82	0.77	0.79
Neuroguidin	NGDN	0.52*	0.50*	0.71*	0.85	0.74	0.82	0.74
Inositol polyphosphate-4-phosphatase type I A	INPP4A	0.52*	0.62	0.73	0.89	1.91*	1.37	1.56
Receptor-type tyrosine-protein phosphatase F	PTPRF	0.52*	0.48*	0.95	0.70*	0.47*	0.47*	0.64*
Nuclear receptor subfamily 2 group F member 6	NR2F6	0.53*	0.55*	0.85	1.03	0.36*	0.53	0.69
Proliferation marker protein Ki-67	MKI67	0.53*	0.44*	0.69	0.89	0.49*	0.62	1.02
Protein arginine N-methyltransferase 3	PRMT3	0.53*	0.56*	0.71*	0.61*	0.69	0.66	0.73
Serine/threonine-protein phosphatase 6 regulatory ankyrin repeat subunit C	ANKRD52	0.54*	0.63	0.71	0.49*	0.75	0.59	0.49*
Condensin complex subunit 1	NCAPD2	0.55*	0.51*	0.67*	0.68*	0.71	0.72	0.95

Mucosa-associated lymphoid tissue lymphoma translocation protein 1	MALT1	0.55*	0.63	0.79	0.62	0.67	0.61	0.58
Integrator complex subunit 11	INTS11	0.56*	0.66*	0.68*	0.59*	0.68	0.79	0.96
Protein FAM110B	FAM110B	0.57*	0.56*	0.69*	0.47*	0.56*	0.52*	0.45*
Coiled-coil domain-containing protein 124	CCDC124	0.57*	0.56*	0.69	0.84	0.69	0.60	0.56
Nephronectin	NPNT	0.57*	0.57*	0.73	0.71	1.35	1.21	1.73
SWI/SNF-related matrix-associated actin-dependent regulator of chromatin subfamily A-like protein 1	SMARCAL1	0.57*	0.55*	0.67*	0.31*	0.77	0.53*	0.44*
Ribosome biogenesis regulatory protein homolog	RRS1	0.57*	0.64*	0.98	1.07	0.46*	0.53*	0.78
Fos-related antigen 1	FOSL1	0.58*	0.55*	0.85	0.79	0.21*	0.42*	0.78
Target of EGR1 protein 1	TOE1	0.59*	0.60*	0.69*	0.57*	0.94	0.84	0.79
RNA-binding protein NOB1	NOB1	0.59*	0.55*	0.71*	0.61*	0.48*	0.50*	0.54*
Periodic tryptophan protein 2 homolog	PWP2	0.60*	0.58*	0.67*	0.55*	0.61*	0.61	0.60*
Ephrin type-A receptor 2	EPHA2	0.60*	0.62*	1.01	1.03	0.74*	0.77	0.99
DNA replication licensing factor MCM6	MCM6	0.61*	0.66*	0.70*	0.91	0.69	0.76	1.02
General transcription and DNA repair factor IIH helicase subunit XPD	ERCC2	0.62*	0.64*	0.68*	0.80	0.94	0.90	1.08
Jupiter microtubule associated homolog 1	JPT1	0.63*	0.58*	0.86	0.78	0.58	0.49*	0.65
Zinc finger C2HC domain-containing protein 1A	ZC2HC1A	0.63*	0.65*	0.81	0.62*	0.45*	0.51*	0.54*
Serine/threonine-protein kinase 11-interacting protein	STK11IP	0.63*	0.66	0.79	0.32*	0.11*	0.24*	0.36*
ATP-dependent RNA helicase DDX51	DDX51	0.64*	0.66*	0.72*	0.66*	0.55*	0.60	0.70
ATP-dependent RNA helicase DDX54	DDX54	0.64*	0.60*	0.72	0.75	0.24*	0.34*	0.56
Cyclin-dependent kinase 7	CDK7	0.65	0.58*	0.98	0.68	0.73	0.84	0.93
Telomerase-binding protein EST1A	SMG6	0.65*	0.61*	0.87	0.65	0.60	0.67	0.66
Keratin, type II cytoskeletal 5	KRT5	0.65*	0.63*	0.87	1.64*	1.09	0.72*	0.86
Ubiquitin carboxyl-terminal hydrolase 48	USP48	0.66*	0.45*	0.72	0.53*	0.62	0.70	0.74
Ribosome biogenesis protein BOP1	BOP1	0.66*	0.58*	0.70*	0.77	0.53*	0.54*	0.56
Unconventional myosin-Vc	MYO5C	0.66	0.63*	0.70	0.62*	0.67	0.78	0.66
Acyl-CoA (8-3)-desaturase	FADS1	0.67*	0.65*	0.70*	0.36*	0.52*	0.44*	0.41*
Spondin-1	SPON1	0.67*	0.66*	1.05	0.73*	0.56*	0.49*	0.69*

F. Common down-regulated proteins between Aquamin and Mesalamine [35 proteins]

		Interventions						
		Control			*With LPS & Cytokines*			
Proteins	Genes	**AQ**	AQ+MES	**MES**	*LPS-Cyto*	*AQ*	*AQ+MES*	*MES*
Extracellular serine/threonine protein kinase FAM20C	FAM20C	0.34*	0.67	0.26*	2.60*	9.33*	8.03*	9.49*
Protein Shroom1	SHROOM1	0.34*	0.73	0.64*	0.26*	0.49*	0.40*	0.24*

Serine/threonine-protein kinase WNK2	WNK2	0.36*	0.74	0.58*	0.39*	0.72	0.77	0.79
Coilin	COIL	0.37*	0.67	0.64*	0.77	1.39	1.58	1.54
Zinc finger FYVE domain-containing protein 26	ZFYVE26	0.41*	0.71	0.58*	0.36*	0.55	0.65	0.44*
Keratin, type I cytoskeletal 25	KRT25	0.44*	1.22	0.25*	0.41*	1.70*	0.23*	1.17
Protein DENND6B	DENND6B	0.44*	1.30	0.61*	0.48*	0.07*	0.15*	0.24*
Dermokine	DMKN	0.47*	0.70	0.58*	0.64*	1.58	0.79	1.33
Progesterone-induced-blocking factor 1	PIBF1	0.51*	0.67*	0.65*	0.46*	0.97	1.00	0.67
Rapamycin-insensitive companion of mTOR	RICTOR	0.52*	0.69	0.57*	0.47*	0.57	0.72	0.69
Zinc finger FYVE domain-containing protein 16	ZFYVE16	0.52*	0.76	0.62*	0.59*	0.40*	0.71	0.60
Rho GTPase-activating protein 32	ARHGAP32	0.53*	0.71	0.65*	0.42*	0.52*	0.63	0.46*
Chromosome alignment-maintaining phosphoprotein 1	CHAMP1	0.53*	0.76	0.62*	0.74	0.17*	0.25*	0.60*
Mediator of RNA polymerase II transcription subunit 22	MED22	0.54*	0.67	0.61*	0.62*	1.13	1.42	1.22
Polyhomeotic-like protein 2	PHC2	0.54*	0.75	0.61*	0.74	0.79	0.56	0.55
Microtubule-associated tumor suppressor 1	MTUS1	0.55*	0.71	0.59*	0.56*	0.91	0.83	0.68
Transmembrane protein 201	TMEM201	0.55*	0.68*	0.64*	0.55*	0.22*	0.38*	0.44*
Myosin-2	MYH2	0.56*	0.80	0.45*	1.12	5.57*	4.93*	4.82*
Pleckstrin	PLEK	0.56*	1.21	0.63*	1.27	4.09*	3.74*	4.36*
Single-stranded DNA-binding protein 3	SSBP3	0.56*	0.77	0.66*	0.58*	1.24	1.30	1.34
E3 ubiquitin-protein ligase TRIM36	TRIM36	0.58*	0.75	0.66*	0.49*	0.45*	0.50*	0.27*
Cleavage and polyadenylation specificity factor subunit 4	CPSF4	0.61*	0.69*	0.66*	0.55*	0.69	0.65	0.61*
Serine/threonine-protein phosphatase 6 regulatory subunit 2	PPP6R2	0.61*	0.71*	0.58*	0.47*	0.49*	0.54*	0.32*
Secreted Ly-6/uPAR domain-containing protein 2	SLURP2	0.62*	0.79	0.42*	2.19*	4.45*	1.19	3.77*
Rho GTPase-activating protein 21	ARHGAP21	0.62*	0.85	0.64*	0.69	1.05	0.85	0.73
Aftiphilin	AFTPH	0.62*	0.68	0.63*	0.72	0.96	0.84	0.86
3-hydroxy-3-methylglutaryl-coenzyme A reductase	HMGCR	0.62*	0.67	0.59*	0.55*	0.62	0.57	0.53*
Intermembrane lipid transfer protein VPS13D	VPS13D	0.63*	0.69	0.51*	0.69	0.82	0.95	0.49*
A-kinase anchor protein 8-like	AKAP8L	0.64*	0.71	0.65*	0.46*	0.25*	0.36*	0.45*
Dynein axonemal heavy chain 8	DNAH8	0.65*	0.96	0.58*	0.19*	0.36*	2.06*	0.37*
Tripartite motif-containing protein 5	TRIM5	0.65*	0.68	0.64*	0.55*	0.75	0.87	0.57
Uridine-cytidine kinase 2	UCK2	0.65*	0.69	0.67*	0.44*	0.39*	0.36*	0.34*
Kinase D-interacting substrate of 220 kDa	KIDINS220	0.66	0.81	0.67	0.81	0.76	0.85	0.57
Pogo transposable element with ZNF domain	POGZ	0.66*	0.71	0.59*	0.72	1.02	0.89	0.89
Lipid droplet assembly factor 1	LDAF1	0.67*	0.75	0.66	0.50*	0.54	0.57	0.41*

G. Common down-regulated proteins between Mesalamine and Aquamin plus Mesalamine [53 proteins]

		Interventions						

Proteins	Genes	Control			*With LPS & Cytokines*			
		AQ	**AQ+MES**	**MES**	*LPS-Cyto*	*AQ*	*AQ+MES*	*MES*
Glutathione S-transferase A2	GSTA2	1.36*	0.40*	0.10*	2.33*	1.56*	0.19*	0.50*
Calcium/calmodulin-dependent protein kinase type 1B	PNCK	1.12	0.39*	0.14*	1.00	1.60	0.82	0.13*
3 beta-hydroxysteroid dehydrogenase/Delta 5-->4-isomerase type 2	HSD3B2	1.43*	0.44*	0.24*	0.88	1.24	0.30*	0.21*
Meprin A subunit beta	MEP1B	1.18	0.37*	0.25*	1.11	1.11	0.36*	0.29*
UPF0235 protein C15orf40	C15orf40	0.86	0.40*	0.33*	1.30	0.91	0.45*	0.67
Carboxypeptidase O	CPO	1.36*	0.54*	0.34*	1.41*	1.27	0.41*	0.39*
Beta-1,3-N-acetylglucosaminyltransferase lunatic fringe	LFNG	0.67*	0.50*	0.41*	0.90	3.55*	2.99*	3.10*
Ornithine transcarbamylase, mitochondrial	OTC	1.13	0.62*	0.42*	0.86	1.03	0.41*	0.48*
Gastrotropin	FABP6	1.05	0.52*	0.43*	0.66*	0.55*	0.35*	0.48*
Tripartite motif-containing protein 3	TRIM3	0.85	0.53*	0.45*	0.64*	0.79	0.92	0.52
Arylamine N-acetyltransferase 1	NAT1	0.88	0.42*	0.46*	0.46*	0.77	0.60	0.62
Iodotyrosine deiodinase 1	IYD	0.94	0.57*	0.46*	0.68*	1.05	0.57*	0.51*
Glutathione S-transferase A1	GSTA1	0.72*	0.38*	0.47*	0.45*	0.49*	0.64	0.41*
Mitochondrial inner membrane protease ATP23 homolog	ATP23	1.03	0.58*	0.47*	0.82	0.73	0.58	0.46*
Profilin-3	PFN3	2.06*	0.44*	0.48*	0.82	0.81	0.84	0.37*
Mediator of RNA polymerase II transcription subunit 8	MED8	3.06*	0.51*	0.49*	22.20*	45.95*	32.32*	21.41*
Solute carrier family 13 member 2	SLC13A2	0.79	0.59*	0.49*	0.99	0.97	0.46*	0.81
Integrin alpha-1	ITGA1	0.90	0.54*	0.51*	0.80	0.98	0.51*	0.52*
ADP-ribosylation factor-like protein 14	ARL14	0.93	0.60*	0.53*	0.92	0.82	0.60	0.63
Pleckstrin homology-like domain family B member 1	PHLDB1	0.73*	0.61*	0.53*	0.70*	0.41*	0.32*	0.43*
Death-associated protein 1	DAP	0.68	0.53*	0.55*	0.77	0.45*	0.43*	0.48*
Fatty acyl-CoA reductase 2	FAR2	0.82	0.49*	0.55*	0.52*	0.11*	0.25*	0.27*
Mitochondrial tRNA methylthiotransferase CDK5RAP1	CDK5RAP1	0.68	0.62*	0.56*	0.69	0.60	0.56	0.58
DNA-directed RNA polymerase I subunit RPA1	POLR1A	0.74*	0.60*	0.57*	0.61*	0.73	0.64	0.71
DNA replication licensing factor MCM7	MCM7	0.82	0.61*	0.58*	0.83	0.93	0.94	1.16
Bridge-like lipid transfer protein family member 1	BLTP1	0.98	0.65	0.59*	0.88	1.01	0.80	0.58
Trefoil factor 2	TFF2	1.26	0.61*	0.60*	0.42*	0.59	0.34*	0.33*
Alpha-2A adrenergic receptor	ADRA2A	0.76	0.55*	0.60*	0.82	0.88	0.76	0.72
DNA mismatch repair protein Msh6	MSH6	0.85	0.62*	0.61*	0.60*	0.81	0.76	0.63
SH2 domain-containing protein 3A	SH2D3A	0.73*	0.66*	0.61*	0.66*	0.45*	0.51*	0.50*
Keratin, type I cytoskeletal 17	KRT17	0.71*	0.64*	0.61*	1.72*	0.68	0.55*	0.77
Inhibitor of growth protein 1	ING1	1.02	0.47*	0.62*	0.86	1.16	0.73	0.59
DNA methyltransferase 1-associated protein 1	DMAP1	0.69	0.59*	0.62*	0.73	0.93	0.89	0.84
Beta-chimaerin	CHN2	0.86	0.57*	0.62*	0.94	0.85	0.56*	0.58*

Mediator of DNA damage checkpoint protein 1	MDC1	0.91	0.65	0.62*	0.70	0.35*	0.50*	1.13
Target of rapamycin complex subunit LST8	MLST8	0.68*	0.67*	0.63*	0.78	1.17	1.14	0.85
Trafficking protein particle complex subunit 10	TRAPPC10	0.69*	0.66*	0.63*	0.55*	0.71	0.72	0.54*
Ribosomal protein S6 kinase alpha-4	RPS6KA4	0.69*	0.47*	0.63*	0.61*	0.49*	0.63	0.55*
ATPase MORC2	MORC2	0.67*	0.63*	0.64*	0.76	1.04	0.99	1.02
L-fucose kinase	FCSK	0.87	0.67*	0.64*	0.79	0.87	0.77	0.88
Pre-mRNA-splicing factor ATP-dependent RNA helicase PRP16	DHX38	0.84	0.66*	0.64*	0.68*	0.65	0.66	0.68*
F-box/LRR-repeat protein 18	FBXL18	0.74*	0.59*	0.64*	0.70	0.60	0.61	0.67
Protein TASOR	TASOR	0.74	0.65	0.64*	0.68	0.54	0.68	0.76
Ephrin-A2	EFNA2	0.88	0.60*	0.64*	0.88	0.70	0.70	0.55*
TGF-beta receptor type-2	TGFBR2	0.83	0.58*	0.65*	0.69	0.94	0.64	0.60
Alanyl-tRNA editing protein Aarsd1	AARSD1	0.76	0.66*	0.65*	0.82	0.74	0.73	0.80
Transforming acidic coiled-coil-containing protein 1	TACC1	0.94	0.62*	0.65*	0.84	0.56*	0.47*	0.61
Eukaryotic translation initiation factor 2D	EIF2D	0.75	0.61*	0.65*	0.73	0.40*	0.77	0.65
IQ motif and SEC7 domain-containing protein 2	IQSEC2	0.74	0.64	0.66*	0.69	0.89	0.72	0.85
Alcohol dehydrogenase 6	ADH6	1.07	0.66*	0.66*	0.79	0.97	0.65*	0.74
Large subunit GTPase 1 homolog	LSG1	0.76*	0.64*	0.66*	0.65*	0.54*	0.52*	0.52*
Atypical kinase COQ8B, mitochondrial	COQ8B	0.71	0.60*	0.66*	0.60*	0.50*	0.61	0.72
DNA-directed RNA polymerase II subunit RPB3	POLR2C	0.78	0.60*	0.66*	1.00	0.69	0.70	0.92

Values represent the abundance ratio from organoids (n=4 subjects) compared to the control. These proteins were down-regulated at a 1.5-fold change (<2% FDR). Corresponding abundance ratios from the other treatment groups are provided for comparison. Proteins common among groups and unique to individual groups under control conditions are presented. *Indicates significance compared to the control (at p<0.05).
