# Supplementary material for: Proteomic profile of human colon organoids: effects of a multi-mineral intervention alone and in the presence of pro-inflammatory and anti-inflammatory treatments
Source: Front Gastroenterol (Lausanne). 2025 Jul 2;4:1592669. doi: 10.3389/fgstr.2025.1592669 (PMC12952359; doi:10.3389/fgstr.2025.1592669)
Supplement: Supplementary Table S1 — Mineral composition of Aquamin. [file DataSheet1.zip › Table S5.pdf]

Supplement Table 5. Up-regulated proteins influenced by LPS-Cytokines alone and with Aquamin and Mesalamine (with 1.5-fold)

A. Common among four groups: LPS-Cytokines, +Aquamin, +Mesalamine and +Aquamin plus Mesalamine [296 proteins]

		Interventions						
		Control			*With LPS & Cytokines*			
Proteins	Genes	AQ	AQ+MES	MES	***LPS-Cyto***	***AQ***	***AQ+MES***	***MES***
HLA class II histocompatibility antigen, DRB1 beta chain	HLA-DRB1	0.99	1.11	1.30	36.37*	27.95*	34.64*	33.75*
Guanylate-binding protein 7	GBP7	1.02	2.02*	0.88	35.72*	32.72*	30.59*	33.79*
HLA class II histocompatibility antigen, DR alpha chain	HLA-DRA	1.05	1.75*	1.05	28.26*	24.40*	28.76*	24.96*
Bromodomain-containing protein 8	BRD8	1.65*	0.96	0.74	26.61*	35.18*	48.74*	52.93*
Mediator of RNA polymerase II transcription subunit 8	MED8	3.06*	0.51*	0.49*	22.20*	45.95*	32.32*	21.41*
Guanylate-binding protein 4	GBP4	1.91*	0.78	0.94	22.11*	26.71*	25.20*	24.08*
Programmed cell death 1 ligand 1	CD274	0.43*	1.53	1.44	16.94*	16.35*	19.58*	15.89*
HLA class II histocompatibility antigen, DP beta 1 chain	HLA-DPB1	0.31*	2.33*	1.80*	15.14*	13.55*	15.68*	15.01*
Indoleamine 2,3-dioxygenase 1	IDO1	0.88	0.96	0.89	15.01*	11.04*	10.46*	12.89*
Secreted and transmembrane protein 1	SECTM1	1.01	1.76*	1.78*	14.70*	8.17*	7.82*	13.21*
HLA class II histocompatibility antigen, DR beta 3 chain	HLA-DRB3	0.90	1.00	1.64*	12.42*	9.92*	10.03*	11.55*
Tryptophan--tRNA ligase, cytoplasmic	WARS1	0.89	1.08	1.05	11.26*	9.10*	9.83*	10.44*
Putative histone H2B type 2-C	H2BC20P	2.73*	0.76	0.85	10.59*	34.44*	25.80*	21.48*
Apolipoprotein C-II	APOC2	2.38*	0.68	0.99	9.94*	21.64*	14.48*	17.84*
HLA class II histocompatibility antigen gamma chain	CD74	1.27	1.28	1.16	9.37*	9.26*	9.53*	7.79*
Heat shock 70 kDa protein 1-like	HSPA1L	2.61*	1.48	1.29	8.97*	15.03*	9.11*	10.55*
HLA class II histocompatibility antigen, DP alpha 1 chain	HLA-DPA1	0.99	0.88	1.17	8.78*	8.23*	8.36*	9.34*
Vesicle-associated membrane protein 5	VAMP5	1.13	1.43	1.57	7.65*	6.90*	6.61*	6.29*
HLA class I histocompatibility antigen, B alpha chain	HLA-B	1.47*	2.37*	1.52*	7.62*	5.91*	7.94*	8.75*
Protein-glutamine gamma-glutamyltransferase 2	TGM2	1.11	1.32*	1.16	7.41*	7.72*	8.15*	7.52*
Guanylate-binding protein 1	GBP1	1.00	1.01	1.08	5.93*	5.84*	6.03*	6.15*
Ubiquitin/ISG15-conjugating enzyme E2 L6	UBE2L6	1.34	1.56*	1.29	5.90*	5.70*	6.47*	6.58*
Protein bassoon	BSN	0.50*	2.53*	1.96*	5.37*	19.19*	17.24*	29.63*
NADH dehydrogenase [ubiquinone] 1 beta subcomplex subunit 2, mitochondrial	NDUFB2	2.06*	1.92*	3.85*	5.33*	9.24*	8.41*	5.38*
HLA class I histocompatibility antigen, alpha chain F	HLA-F	1.23	1.49*	1.32	5.25*	4.42*	5.26*	5.03*
Sorting nexin-24	SNX24	3.83*	4.44*	3.16*	5.18*	5.74*	3.20*	4.30*
Thymidine phosphorylase	TYMP	1.24	1.52*	1.28	4.52*	3.92*	4.88*	4.23*
Methionine-R-sulfoxide reductase B2, mitochondrial	MSRB2	1.00	1.10	0.78	4.44*	13.63*	14.13*	14.06*

Tapasin	TAPBP	1.23*	1.27*	1.26*	4.39*	4.05*	4.83*	3.93*
Nitric oxide synthase, inducible	NOS2	1.07	1.14	1.22	4.35*	3.78*	5.27*	3.94*
Signal transducer and activator of transcription 1-alpha/beta	STAT1	1.04	1.05	1.01	4.34*	4.13*	4.36*	4.41*
Cytosol aminopeptidase	LAP3	0.96	1.09	0.97	4.32*	4.45*	4.43*	3.93*
Caspase-1	CASP1	1.07	1.17	1.45	4.31*	3.38*	3.74*	4.07*
Antigen peptide transporter 1	TAP1	1.06	1.23	1.08	4.28*	4.37*	4.87*	4.06*
Equilibrative nucleoside transporter 1	SLC29A1	2.51*	2.80*	2.70*	4.14*	4.67*	4.44*	3.78*
HLA class I histocompatibility antigen, A alpha chain	HLA-A	1.14	1.36*	1.18*	4.09*	4.31*	4.64*	3.93*
Tumor necrosis factor receptor superfamily member 5	CD40	0.84	1.23	1.53	3.99*	4.56*	4.44*	4.22*
Antigen peptide transporter 2	TAP2	0.97	1.03	1.03	3.89*	4.20*	4.32*	3.62*
Beta-2-microglobulin	B2M	1.10	1.32*	1.10	3.77*	4.13*	4.31*	4.05*
S-adenosylmethionine-dependent nucleotide dehydratase RSAD2	RSAD2	0.94	1.33	1.23	3.73*	6.25*	5.47*	2.08*
Interferon-induced GTP-binding protein Mx1	MX1	1.07	1.34	1.50*	3.61*	4.42*	5.17*	4.06*
Apolipoprotein L2	APOL2	0.92	1.01	0.97	3.60*	3.29*	3.98*	3.24*
Hepatocyte growth factor	HGF	1.29*	0.68*	0.82	3.59*	11.24*	9.91*	8.32*
Proteasome subunit beta type-10	PSMB10	1.06	1.34	1.22	3.51*	2.90*	4.61*	3.18*
HLA class I histocompatibility antigen, C alpha chain	HLA-C	1.10	1.34*	1.24*	3.50*	3.60*	4.35*	3.49*
Serum amyloid A-4 protein	SAA4	0.63*	2.09*	2.47*	3.32*	9.01*	8.67*	16.73*
Microfibril-associated glycoprotein 4	MFAP4	0.79	0.80	1.01	3.31*	11.58*	10.17*	10.31*
Interferon-induced protein with tetratricopeptide repeats 2	IFIT2	1.14	0.95	1.00	3.31*	4.23*	5.12*	2.98*
Lipocalin-1	LCN1	1.05	0.73	0.87	3.28*	5.55*	3.52*	5.52*
Gamma-interferon-inducible lysosomal thiol reductase	IFI30	1.04	1.13	1.17	3.26*	3.09*	3.31*	2.96*
Protein FAM234B	FAM234B	1.38	1.35	1.46*	3.26*	5.10*	4.90*	2.41*
Retinoic acid receptor responder protein 1	RARRES1	1.60*	2.72*	3.25*	3.23*	3.43*	6.05*	6.66*
Cytochrome b	MT-CYB	2.22*	1.67*	1.38	3.21*	5.40*	5.01*	3.12*
Phospholipase A2, membrane associated	PLA2G2A	1.31	4.25*	3.95*	3.18*	8.37*	15.64*	17.81*
Transmembrane and immunoglobulin domain-containing protein 1	TMIGD1	2.53*	7.38*	3.86*	3.17*	3.35*	4.41*	2.89*
HLA class I histocompatibility antigen, alpha chain E	HLA-E	0.98	1.36	1.22	3.08*	2.16*	2.05*	2.19*
Group IID secretory phospholipase A2	PLA2G2D	1.94*	0.98	0.85	3.07*	7.57*	9.66*	8.40*
Solute carrier family 40 member 1	SLC40A1	1.66*	1.70*	1.81*	3.07*	4.44*	3.81*	3.78*
Homeobox protein DBX1	DBX1	1.57*	1.54*	1.49*	3.07*	6.91*	6.60*	5.35*
Endoplasmic reticulum aminopeptidase 2	ERAP2	1.30*	1.34*	1.20*	3.05*	2.93*	2.65*	2.76*
Sodium-coupled neutral amino acid transporter 5	SLC38A5	0.85	1.24	1.29	3.03*	4.78*	3.88*	4.40*
Uncharacterized protein KIAA0040	KIAA0040	1.32	1.26	1.38	2.96*	3.16*	2.75*	3.21*

Putative HLA class I histocompatibility antigen, alpha chain H	HLA-H	1.19	1.64*	1.17	2.96*	2.63*	2.82*	2.42*
All-trans-retinol dehydrogenase [NAD(+)] ADH4	ADH4	1.07	2.55*	2.71*	2.95*	2.62*	2.01*	1.81*
Butyrophilin subfamily 3 member A3	BTN3A3	0.89	1.45	1.31	2.95*	2.00*	2.40*	2.94*
Complement factor I	CFI	0.68	2.00*	2.08*	2.94*	6.10*	6.03*	11.20*
Alpha-1-antitrypsin	SERPINA1	1.26*	1.76*	1.41*	2.93*	2.81*	2.44*	2.34*
Cathepsin O	CTSO	0.99	1.06	0.77	2.92*	3.74*	3.50*	2.61*
Filamin-C	FLNC	1.23	0.84	1.90*	2.91*	3.79*	4.00*	2.41*
Myeloid leukemia factor 2	MLF2	2.19*	1.97*	3.37*	2.90*	2.56*	1.98*	2.36*
Zinc finger protein 45	ZNF45	0.85	0.94	0.79	2.89*	7.63*	8.24*	8.00*
Neutral amino acid transporter A	SLC1A4	1.27	1.32	1.48*	2.87*	5.21*	5.15*	4.25*
Calcium uniporter regulatory subunit MCUb, mitochondrial	MCUB	1.24*	1.28*	1.27*	2.86*	3.39*	3.50*	3.08*
Sodium-coupled neutral amino acid symporter 2	SLC38A2	1.13	1.31	1.44*	2.86*	2.53*	3.21*	3.48*
Xylosyl- and glucuronyltransferase LARGE1	LARGE1	1.38	1.47	1.40	2.86*	5.42*	4.76*	3.33*
Ubiquitin D	UBD	1.03	4.25*	3.84*	2.84*	3.54*	12.39*	12.06*
Complement C3	C3	0.68*	2.23*	3.76*	2.82*	3.40*	4.41*	3.80*
Keratinocyte-associated transmembrane protein 2	KCT2	1.12	1.69*	1.51*	2.82*	2.19*	2.57*	2.90*
2'-5'-oligoadenylate synthase 3	OAS3	1.09	1.29	1.03	2.80*	3.02*	3.38*	3.00*
Cystine/glutamate transporter	SLC7A11	1.28	0.94	1.01	2.78*	5.27*	4.98*	2.84*
Neuronal calcium sensor 1	NCS1	0.67	1.11	1.15	2.78*	3.34*	3.17*	2.49*
Guanylate-binding protein 2	GBP2	1.05	1.05	1.09	2.75*	2.72*	2.89*	2.95*
E3 ubiquitin-protein ligase MSL2	MSL2	0.47*	0.40*	0.51*	2.74*	11.31*	10.04*	11.22*
Pro-opiomelanocortin	POMC	0.96	2.08*	2.21*	2.71*	4.33*	6.13*	10.26*
PDZ and LIM domain protein 4	PDLIM4	1.23	1.00	1.37	2.70*	2.24*	2.23*	2.43*
Apolipoprotein L1	APOL1	1.46	1.73*	1.82*	2.68*	3.15*	3.75*	2.64*
Protein unc-93 homolog A	UNC93A	0.51*	1.04	1.49	2.62*	1.95*	2.34*	3.58*
Plexin domain-containing protein 2	PLXDC2	1.16	1.24	1.67*	2.61*	5.06*	7.61*	8.74*
Extracellular serine/threonine protein kinase FAM20C	FAM20C	0.34*	0.67	0.26*	2.60*	9.33*	8.03*	9.49*
Fibrinogen gamma chain	FGG	0.35*	1.28	2.38*	2.58*	3.47*	3.42*	6.21*
Intercellular adhesion molecule 1	ICAM1	0.83	0.89	1.02	2.57*	2.62*	2.53*	2.58*
Major facilitator superfamily domain-containing protein 8	MFSD8	1.61*	1.27	1.09	2.56*	4.01*	3.96*	3.35*
Transmembrane protein 143	TMEM143	1.27*	1.40	1.43*	2.56*	4.25*	3.90*	3.12*
Keratinocyte-associated protein 2	KRTCAP2	1.54*	1.29	1.18	2.55*	4.86*	4.63*	2.87*
WD repeat domain phosphoinositide-interacting protein 1	WIPI1	1.14	1.11	1.19	2.55*	6.55*	2.34*	3.16*
2-aminomuconic semialdehyde dehydrogenase	ALDH8A1	1.90*	1.21	1.32	2.50*	13.33*	30.36*	5.64*
Tetraspanin-9	TSPAN9	0.64	0.93	0.79	2.49*	7.29*	7.91*	7.67*

Zinc transporter ZIP9	SLC39A9	1.28	1.40	1.31	2.48*	4.55*	4.80*	3.72*
DNA-binding protein SMUBP-2	IGHMBP2	0.58*	1.53*	1.72*	2.47*	3.42*	3.38*	5.07*
Secretogranin-3	SCG3	1.21	1.79*	1.95*	2.47*	2.79*	3.40*	4.48*
Guanylate-binding protein 5	GBP5	0.75	0.94	0.97	2.46*	2.69*	2.45*	2.83*
Sodium/myo-inositol cotransporter	SLC5A3	1.68*	1.70*	1.41	2.45*	3.59*	4.91*	2.00*
Betaine--homocysteine S-methyltransferase 1	BHMT	0.31*	0.41*	0.31*	2.44*	13.21*	10.27*	6.92*
Interferon-induced protein with tetratricopeptide repeats 1	IFIT1	0.75	1.08	0.65*	2.44*	3.27*	3.50*	2.42*
cAMP-dependent protein kinase inhibitor beta	PKIB	0.88	6.48*	9.41*	2.44*	2.06*	11.12*	18.67*
Proteasome activator complex subunit 2	PSME2	1.08	1.16	1.02	2.43*	2.30*	2.48*	2.36*
Beta-2-glycoprotein 1	APOH	1.26	2.33*	2.52*	2.43*	2.14*	2.35*	3.00*
Albumin	ALB	0.63*	1.73*	2.02*	2.42*	1.96*	2.70*	2.09*
Lactotransferrin	LTF	0.98	1.77*	2.37*	2.39*	2.02*	2.78*	3.12*
Tetraspanin-33	TSPAN33	1.76*	1.53	1.49	2.39*	3.91*	2.87*	2.46*
High affinity cationic amino acid transporter 1	SLC7A1	1.76*	1.42*	1.56*	2.39*	3.41*	3.56*	2.53*
Insulin-like growth factor-binding protein complex acid labile subunit	IGFALS	0.23*	0.37*	0.33*	2.38*	12.16*	9.41*	11.08*
Heme transporter FLVCR1	FLVCR1	1.18	1.20	1.29	2.36*	4.38*	4.25*	3.94*
Ubiquitin-like protein ISG15	ISG15	1.08	1.13	1.07	2.34*	1.92*	2.65*	2.14*
Intercellular adhesion molecule 2	ICAM2	1.34*	1.14	1.49*	2.34*	2.32*	1.74*	2.27*
Coiled-coil domain-containing protein 39	CCDC39	0.41*	0.77	1.31	2.33*	2.48*	2.44*	2.90*
Endoplasmic reticulum membrane adapter protein XK	XK	1.59*	1.81*	1.36	2.33*	3.37*	4.41*	2.84*
Peroxisome proliferator-activated receptor delta	PPARD	1.01	1.05	1.11	2.31*	3.67*	4.20*	6.07*
V-type immunoglobulin domain-containing suppressor of T-cell activation	VSIR	1.60*	1.43	1.49*	2.30*	5.02*	3.78*	3.72*
Inositol-3-phosphate synthase 1	ISYNA1	1.33*	1.61*	1.53*	2.28*	3.96*	2.92*	3.27*
DnaJ homolog subfamily C member 15	DNAJC15	1.21	1.83*	1.61*	2.28*	3.11*	2.09*	2.92*
Tropomodulin-2	TMOD2	0.97	2.08*	1.93*	2.25*	1.95*	2.91*	4.70*
Signal transducer and activator of transcription 2	STAT2	1.09	1.06	1.13	2.24*	2.52*	2.72*	2.89*
FXYD domain-containing ion transport regulator 5	FXYD5	3.37*	3.09*	2.13*	2.24*	5.59*	4.94*	4.29*
TPA-induced transmembrane protein	TTMP	1.81*	1.96*	1.42	2.23*	4.60*	4.29*	3.70*
Vang-like protein 1	VANGL1	1.17	1.03	1.13	2.23*	4.05*	4.10*	3.12*
Protein BCAP	ODF2L	0.43*	1.24	1.79*	2.23*	2.03*	2.99*	3.70*
All-trans-retinol dehydrogenase [NAD(+)] ADH7	ADH7	0.29*	0.43*	0.31*	2.22*	11.41*	8.22*	10.30*
Cytochrome c oxidase assembly factor 1 homolog	COA1	1.14	1.44	1.12	2.21*	4.42*	3.51*	3.77*
Protein YIF1A	YIF1A	1.55*	1.60	1.42	2.21*	3.72*	3.42*	2.33*
Sulfhydryl oxidase 2	QSOX2	1.47*	1.88*	1.74*	2.20*	3.05*	3.18*	3.04*
Liprin-alpha-3	PPFIA3	1.23	1.43	1.34*	2.20*	3.75*	3.49*	2.77*

HLA class I histocompatibility antigen, alpha chain G	HLA-G	1.14	1.45*	1.31	2.20*	2.70*	2.49*	1.93*
Nuclease EXOG, mitochondrial	EXOG	1.57*	1.36	1.45	2.19*	3.66*	3.66*	3.13*
Transmembrane protein 186	TMEM186	1.20	1.41	1.16	2.18*	2.89*	2.98*	2.50*
Peptidyl-prolyl cis-trans isomerase FKBP11	FKBP11	0.99	0.87	1.33*	2.18*	5.12*	4.89*	4.49*
Complement factor B	CFB	1.12	1.46	1.67*	2.18*	3.23*	2.38*	2.44*
Hemoglobin subunit beta	HBB	1.03	2.23*	1.89*	2.18*	2.36*	3.19*	4.34*
Glycerophosphodiester phosphodiesterase 1	GDE1	1.65*	1.63*	1.36*	2.17*	3.02*	3.04*	1.72*
EF-hand calcium-binding domain-containing protein 14	EFCAB14	1.26	1.38	1.36	2.16*	2.55*	2.99*	2.12*
Mitochondrial glutamate carrier 1	SLC25A22	1.07	1.35*	1.12	2.16*	2.36*	2.38*	2.04*
E3 ubiquitin-protein ligase TRIM21	TRIM21	1.24	1.08	1.31*	2.13*	1.75*	2.06*	1.95*
Collagen alpha-1(XII) chain	COL12A1	0.71	1.58	1.57*	2.12*	3.69*	6.05*	6.67*
Aldehyde oxidase	AOX1	0.14*	0.14*	0.27*	2.11*	11.69*	8.47*	9.08*
Lymphocyte antigen 6D	LY6D	0.89	1.56	2.25*	2.10*	2.22*	1.53	1.51
Interleukin-32	IL32	1.22	1.36	1.26	2.10*	2.35*	2.49*	2.12*
Inter-alpha-trypsin inhibitor heavy chain H4	ITIH4	0.56*	1.42	1.59*	2.09*	1.62*	2.06*	3.18*
Tetranectin	CLEC3B	0.72	1.19	2.37*	2.09*	1.76*	2.13*	2.78*
Proteasome activator complex subunit 1	PSME1	0.99	1.28*	1.17*	2.09*	1.89*	2.22*	1.95*
Apolipoprotein C-III	APOC3	0.91	2.22*	1.66*	2.09*	2.28*	2.65*	3.96*
Vitamin K-dependent protein Z	PROZ	0.80	0.77	0.91	2.08*	5.00*	4.05*	4.90*
Thrombospondin type-1 domain-containing protein 4	THSD4	1.04	0.98	1.23	2.06*	12.07*	6.18*	3.67*
Fermitin family homolog 2	FERMT2	0.05*	0.35*	0.25*	2.05*	9.27*	9.50*	10.36*
Tapasin-related protein	TAPBPL	1.19	1.24	1.12	2.05*	1.86*	2.18*	1.93*
HLA class II histocompatibility antigen, DR beta 5 chain	HLA-DRB5	0.87	1.01	0.77	2.05*	2.43*	1.98*	2.20*
Proteasome subunit beta type-9	PSMB9	1.05	1.16	1.14	2.04*	1.77*	2.18*	1.90*
Vascular cell adhesion protein 1	VCAM1	0.54*	1.13	1.26	2.04*	3.97*	5.56*	6.73*
Laminin subunit alpha-4	LAMA4	1.09	0.93	0.89	2.04*	2.27*	2.54*	2.81*
Heparin cofactor 2	SERPIND1	0.75	1.73*	2.75*	2.04*	3.83*	4.33*	7.38*
Zinc finger CCCH-type with G patch domain-containing protein	ZGPAT	1.03	0.97	0.95	2.00*	1.95*	2.17*	2.19*
Proteasome subunit beta type-8	PSMB8	1.37*	1.51*	1.39*	2.00*	1.62*	2.82*	1.92*
Beta-1,4-galactosyltransferase 5	B4GALT5	1.06	1.31	1.42	2.00*	3.85*	3.02*	3.50*
Probable ATP-dependent RNA helicase DDX60	DDX60	1.09	1.18	1.04	2.00*	2.38*	2.40*	2.03*
Shiftless antiviral inhibitor of ribosomal frameshifting protein	SHFL	1.10	1.04	1.06	1.99*	2.01*	1.82*	1.79
Solute carrier family 66 member 2	SLC66A2	1.55*	1.44*	1.44*	1.99*	4.16*	3.88*	3.56*
Interferon-induced protein with tetratricopeptide repeats 3	IFIT3	1.24	2.65*	2.64*	1.98*	2.49*	4.45*	4.39*
Tubulin beta-1 chain	TUBB1	0.21*	0.28*	0.24*	1.96*	9.06*	7.77*	9.62*

Short transmembrane mitochondrial protein 1	STMP1	1.49*	1.43	1.46	1.95*	5.09*	4.29*	4.19*
Beta-enolase	ENO3	0.50*	0.75	0.76*	1.95*	5.84*	4.93*	4.93*
Battenin	CLN3	1.57*	2.13*	1.44*	1.94*	1.87*	2.41*	2.39*
Mitochondrial import receptor subunit TOM40B	TOMM40L	1.43*	1.59*	1.46*	1.94*	2.74*	2.90*	2.29*
Pyridine nucleotide-disulfide oxidoreductase domain-containing protein 2	PYROXD2	1.28	1.08	1.19	1.94*	2.66*	2.32*	2.26*
Glycolipid transfer protein domain-containing protein 2	GLTPD2	1.47*	2.85*	2.85*	1.93*	2.81*	2.12*	3.41*
Electrogenic aspartate/glutamate antiporter SLC25A12, mitochondrial	SLC25A12	1.34*	1.20	1.21	1.93*	2.84*	2.60*	2.64*
Protein mono-ADP-ribosyltransferase PARP9	PARP9	0.96	0.91	0.90	1.92*	1.60*	1.98*	2.10*
Fibrinogen beta chain	FGB	0.56*	2.04*	1.67*	1.92*	3.53*	3.54*	6.17*
Cathepsin S	CTSS	1.09	1.16	1.03	1.92*	1.57*	1.81*	1.57*
LHFPL tetraspan subfamily member 2 protein	LHFPL2	1.51*	1.61*	1.23	1.92*	2.46*	1.60	1.57
Trophoblast glycoprotein	TPBG	1.42*	1.68*	1.61*	1.90*	2.67*	2.69*	1.69
Visinin-like protein 1	VSNL1	1.20*	1.18	1.08	1.89*	1.79*	1.73*	1.95*
Alpha-2-macroglobulin	A2M	0.61*	1.48*	1.87*	1.88*	1.77*	2.51*	3.48*
Triggering receptor expressed on myeloid cells 1	TREM1	2.25*	2.93*	1.88*	1.88*	4.12*	1.94*	3.89*
Divergent protein kinase domain 2A	DIPK2A	1.22	1.08	1.01	1.88*	2.86*	3.03*	2.68*
Neprilysin	MME	1.22	1.33	1.17	1.86*	3.35*	3.31*	2.68*
SPARC	SPARC	0.54*	1.40*	1.85*	1.86*	1.88*	3.08*	3.36*
Legumain	LGMN	1.14	1.28	1.09	1.85*	2.08*	2.39*	1.53*
Coiled-coil domain-containing protein 127	CCDC127	1.41	1.65*	1.53	1.85*	2.71*	2.49*	2.29*
Stromal interaction molecule 2	STIM2	0.77	1.30	1.75*	1.84*	1.54	2.36*	3.21*
PAT complex subunit Asterix	WDR83OS	1.51*	1.46	1.31	1.84*	5.64*	4.74*	4.39*
Alpha-2-antiplasmin	SERPINF2	0.86	1.62*	1.67*	1.84*	2.47*	5.09*	6.18*
Probable U3 small nucleolar RNA-associated protein 11	UTP11	0.88	0.77	0.91	1.84*	2.62*	2.45*	1.93*
Leukocyte surface antigen CD47	CD47	1.08	1.22	1.19*	1.84*	2.10*	2.04*	1.74*
Immunoglobulin lambda-1 light chain		0.85	1.19	2.66*	1.84*	3.41*	4.29*	5.54*
Complement C5	C5	1.02	1.33	1.48*	1.83*	3.14*	4.28*	5.49*
Laminin subunit alpha-5	LAMA5	1.04	1.44	1.45*	1.83*	1.58	1.98*	2.57*
Complement C4-A	C4A	0.73*	1.29*	2.17*	1.82*	3.29*	3.86*	4.65*
Thrombospondin-4	THBS4	0.67*	1.01	1.08	1.82*	3.14*	4.76*	6.66*
Collagen alpha-1(IV) chain	COL4A1	0.50*	1.17	1.70*	1.82*	2.23*	2.22*	3.55*
Zinc finger protein 536	ZNF536	0.40*	1.44	1.10	1.81*	2.08*	2.70*	5.62*
Mucin-1	MUC1	1.22*	1.12	1.05	1.81*	1.92*	1.94*	1.52*
F-box only protein 6	FBXO6	1.11	1.10	1.29	1.81*	1.73*	1.77*	2.13*
Hemoglobin subunit epsilon	HBE1	0.77	1.36	1.95*	1.80*	2.27*	2.55*	3.81*
Protocadherin-12	PCDH12	0.47*	1.35	1.06	1.79*	2.87*	4.80*	6.92*

Tubulin alpha-3C chain	TUBA3C	0.07*	0.27*	0.11*	1.78*	10.04*	7.83*	8.39*
Sex-determining region Y protein	SRY	0.61*	1.19	1.25	1.77*	1.72	1.71	2.48*
MIT domain-containing protein 1	MITD1	1.24	0.74	0.95	1.77*	2.40*	2.21*	2.51*
Signal peptide peptidase-like 2B	SPPL2B	1.26	1.61*	1.48*	1.77*	2.29*	2.26*	2.37*
Protransforming growth factor alpha	TGFA	1.48*	1.58*	1.65*	1.77*	3.24*	3.42*	3.06*
Metallophosphoesterase 1	MPPE1	1.08	1.37	1.03	1.77*	3.07*	3.52*	3.24*
Carboxypeptidase B2	CPB2	0.23*	1.02	1.24	1.76*	5.38*	5.30*	10.11*
Lymphocyte function-associated antigen 3	CD58	1.37*	1.92*	1.66*	1.76*	2.20*	2.42*	2.22*
Deoxyhypusine synthase	DHPS	0.95	1.73*	1.01	1.75*	1.58*	1.88*	1.77*
Bcl-2 homologous antagonist/killer	BAK1	1.21	1.16	1.24	1.75*	1.83*	1.80*	1.75*
Heat shock 70 kDa protein 6	HSPA6	1.26*	1.15	1.29*	1.75*	2.82*	3.06*	2.61*
Transmembrane reductase CYB561D2	CYB561D2	1.24	1.23	1.32	1.74*	2.33*	1.99*	1.98*
Magnesium transporter MRS2 homolog, mitochondrial	MRS2	1.30	1.34	1.19	1.74*	1.90*	2.64*	2.09*
Thyroxine-binding globulin	SERPINA7	0.70*	1.20	1.31*	1.74*	2.91*	3.91*	4.50*
Coagulation factor V	F5	0.61*	1.22	1.26	1.74*	1.61	1.89*	2.43*
Argininosuccinate synthase	ASS1	1.01	1.12	1.04	1.74*	1.50*	1.78*	1.82*
Superoxide dismutase [Mn], mitochondrial	SOD2	1.20*	1.69*	1.12	1.74*	1.96*	2.13*	2.03*
Arylsulfatase L	ARSL	1.47*	1.34*	1.26	1.74*	2.76*	3.07*	2.11*
Cartilage oligomeric matrix protein	COMP	0.57*	0.84	1.22	1.73*	2.91*	4.29*	6.18*
Osteomodulin	OMD	0.71	1.32	1.18	1.73*	2.97*	4.38*	5.35*
Homologous recombination OB-fold protein	HROB	0.65*	1.35*	1.48*	1.73*	1.51*	2.16*	1.96*
Guided entry of tail-anchored proteins factor 1	GET1	1.03	1.19	1.10	1.73*	2.26*	2.28*	1.64
Macrophage mannose receptor 1	MRC1	0.51*	0.40*	0.41*	1.72*	6.02*	7.32*	7.08*
CCR4-NOT transcription complex subunit 7	CNOT7	1.10	1.02	1.01	1.72*	1.86*	2.13*	2.12*
Xyloside xylosyltransferase 1	XXYLT1	1.34	1.32	1.20	1.72*	1.72*	1.59	1.53
Adenosine deaminase	ADA	0.90	1.07	1.23	1.71*	1.51	1.66*	1.73*
Proteoglycan 4	PRG4	0.74	1.15	1.30	1.71*	3.11*	5.08*	5.60*
2'-5'-oligoadenylate synthase 2	OAS2	0.80	3.38*	3.47*	1.71*	2.58*	9.01*	8.23*
Thrombomodulin	THBD	0.55*	0.62*	0.60*	1.70*	7.47*	6.68*	7.22*
Cell growth regulator with EF hand domain protein 1	CGREF1	1.45*	1.11	1.03	1.70*	3.02*	3.03*	2.29*
Vitamin D-binding protein	GC	0.71*	1.25	1.61*	1.69*	1.51*	2.10*	2.04*
Inter-alpha-trypsin inhibitor heavy chain H3	ITIH3	0.66*	1.15	1.11	1.68*	3.30*	4.88*	6.20*
Sideroflexin-2	SFXN2	1.15	1.13	1.21	1.68*	2.33*	2.13*	1.79*
Hemopexin	HPX	0.65*	1.28	1.58*	1.68*	1.54	1.99*	3.50*
Complement component C8 alpha chain	C8A	0.60*	1.16	1.38*	1.68*	3.50*	4.58*	6.06*
STARD3 N-terminal-like protein	STARD3NL	1.12	1.56*	1.24	1.68*	1.74	1.55	1.57
Antiviral innate immune response receptor RIG-I	RIGI	1.05	1.14	0.93	1.68*	1.84*	1.91*	1.56

E3 ubiquitin-protein ligase RNF213	RNF213	1.05	0.94	1.06	1.68*	1.91*	1.85*	1.84*
Sacsin	SACS	1.37*	1.56*	1.45*	1.67*	2.82*	2.91*	2.32*
Adhesion G-protein coupled receptor F1	ADGRF1	1.32*	1.87*	1.24	1.65*	1.98*	1.80*	1.81*
N-acyl-phosphatidylethanolamine-hydrolyzing phospholipase D	NAPEPLD	1.10	1.08	1.10	1.65*	1.70*	1.59	1.63
Mitochondrial calcium uniporter regulator 1	MCUR1	1.25*	1.48*	1.36*	1.64*	2.36*	2.42*	2.06*
Beta-1,3-galactosyl-O-glycosyl-glycoprotein beta-1,6-N-acetylglucosaminyltransferase	GCNT1	1.12	1.43	1.33	1.64*	2.69*	2.00*	1.56
Derlin-2	DERL2	0.83	1.02	1.18	1.64*	1.74*	1.70	2.16*
Apolipoprotein E	APOE	0.90	1.42*	1.50*	1.64*	1.69*	1.93*	2.28*
Kinesin-like protein KIF6	KIF6	1.14	0.64	0.99	1.63*	1.91*	2.10*	1.79
Mitochondrial import inner membrane translocase subunit Tim17-B	TIMM17B	1.21	1.12	1.22	1.63*	2.34*	2.51*	2.27*
Hepatocyte growth factor-like protein	MST1	1.03	1.45*	1.60*	1.62*	2.56*	3.35*	4.24*
Hemoglobin subunit alpha	HBA1	0.78*	1.53*	1.52*	1.62*	1.74*	2.44*	3.35*
Transgelin	TAGLN	0.82	2.04*	1.68*	1.62*	1.71	2.44*	2.91*
Inter-alpha-trypsin inhibitor heavy chain H2	ITIH2	0.86	1.71*	1.75*	1.61*	2.05*	2.42*	3.06*
Laminin subunit alpha-1	LAMA1	0.55*	2.01*	1.66*	1.60*	2.30*	2.88*	3.96*
Vesicle transport protein SFT2B	SFT2D2	1.27	1.27	1.21	1.60*	1.75*	1.59	1.81*
Parathyroid hormone/parathyroid hormone-related peptide receptor	PTH1R	0.35*	0.33*	0.47*	1.60*	8.24*	7.16*	7.08*
Interferon-induced transmembrane protein 3	IFITM3	0.94	2.48*	1.97*	1.60*	2.12*	4.68*	3.56*
Collagen alpha-1(XVIII) chain	COL18A1	0.84	1.26	1.26	1.60*	1.50*	1.92*	1.65*
Zinc transporter ZIP6	SLC39A6	1.48*	1.24	1.13	1.59*	1.66*	1.58*	1.82*
Olfactomedin-like protein 3	OLFML3	0.35*	1.30	0.87	1.59*	3.28*	5.46*	7.10*
Transmembrane protein 62	TMEM62	0.91	1.13	1.22	1.59*	1.71*	1.82*	1.91*
Lysophosphatidylserine lipase ABHD12	ABHD12	1.20	1.22	1.12	1.59*	1.53*	1.61*	1.77*
Mitochondrial adenyl nucleotide antiporter SLC25A25	SLC25A25	1.30	1.53	1.24	1.58*	2.23*	2.58*	1.80*
Equilibrative nucleoside transporter 2	SLC29A2	1.07	0.93	0.99	1.58*	1.89*	1.84*	1.84*
Mitochondrial pyruvate carrier 1	MPC1	0.99	1.83*	1.33	1.58	2.75*	2.41*	2.70*
Vitronectin	VTN	0.53*	1.33	1.51*	1.58*	1.55	1.93*	2.76*
Beta-galactoside alpha-2,6-sialyltransferase 1	ST6GAL1	1.01	1.00	1.17	1.58*	3.45*	2.72*	2.43*
Laminin subunit beta-1	LAMB1	0.54*	2.04*	1.65*	1.58*	2.39*	2.83*	3.78*
Gasdermin-D	GSDMD	1.07	1.24	0.96	1.57*	1.64*	1.81*	1.59*
BMP-binding endothelial regulator protein	BMPER	0.65	1.20	1.13	1.57*	3.53*	3.94*	4.84*
Cathepsin L2	CTSV	0.87	0.99	1.14	1.57*	1.53	1.52	1.54
Apolipoprotein M	APOM	0.51*	0.86	1.24*	1.56*	2.35*	3.87*	5.61*
Plasminogen activator inhibitor 2	SERPINB2	1.06	1.31	1.15	1.56*	1.64	1.67	1.70

Regulator of microtubule dynamics protein 3	RMDN3	1.19*	1.27*	1.16	1.56*	1.76*	1.81*	1.56*
3-ketodihydrosphingosine reductase	KDSR	1.14	1.50	1.32	1.56*	1.73	1.65	1.50
Ribonuclease P protein subunit p25	RPP25	1.02	1.07	1.07	1.55	1.87*	1.58	1.90*
Interferon-stimulated 20 kDa exonuclease-like 2	ISG20L2	1.32	1.12	1.33	1.55	2.06*	2.39*	2.47*
Vacuolar ATPase assembly integral membrane protein VMA21	VMA21	1.29	1.16	1.02	1.54	2.33*	2.16*	1.98
ER membrane protein complex subunit 7	EMC7	0.97	1.51*	1.43*	1.54*	1.50	1.59	1.99*
DNA polymerase subunit gamma-2, mitochondrial	POLG2	1.24	1.21	1.11	1.54*	1.53	1.63	1.57
Laminin subunit gamma-1	LAMC1	0.61*	1.97*	1.62*	1.54*	2.28*	2.68*	3.65*
Bone marrow stromal antigen 2	BST2	0.90	0.90	0.79	1.53	1.98*	2.11*	1.81
Spectrin beta chain, erythrocytic	SPTB	0.65	0.70	1.24	1.53	3.31*	3.32*	4.60*
Mediator of RNA polymerase II transcription subunit 18	MED18	0.93	0.97	1.07	1.53*	2.41*	1.91*	2.08*
UMP-CMP kinase 2, mitochondrial	CMPK2	0.96	1.64*	1.39	1.53	1.76*	2.93*	2.32*
Immunoglobulin superfamily member 8	IGSF8	1.11	1.61*	1.44*	1.53*	2.10*	1.93*	1.61*
CMP-N-acetylneuraminate-beta-galactosamide-alpha-2,3-sialyltransferase 1	ST3GAL1	0.97	1.05	1.11	1.53*	2.23*	2.22*	1.84*
Adhesion G-protein coupled receptor G7	ADGRG7	1.13	1.34	1.22	1.53	1.55	1.92*	1.56
Glutathione peroxidase 3	GPX3	0.72	1.00	1.35*	1.52*	2.96*	2.09*	2.86*
Sodium/hydrogen exchanger 8	SLC9A8	1.39*	1.34	1.33*	1.52*	2.76*	2.64*	2.00*
Carbamoyl-phosphate synthase [ammonia], mitochondrial	CPS1	0.47*	1.17	0.96	1.52*	4.01*	4.48*	5.28*
Alpha-amylase 1B	AMY1B	0.60*	1.01	1.13	1.52*	3.33*	3.94*	5.43*
Surfeit locus protein 1	SURF1	1.27	1.28	1.12	1.51*	2.16*	2.07*	1.84*
Adenylyl cyclase-associated protein 2	CAP2	0.49*	1.23	1.16	1.51*	3.15*	3.71*	4.96*
Prothrombin	F2	0.84	1.59	1.40	1.51*	2.10*	3.12*	3.81*

B. Up-regulated proteins unique to LPS-Cytokines (LPS-Cyto) [38 proteins]

		Interventions						
		Control			*With LPS & Cytokines*			
Proteins	Genes	AQ	AQ+MES	MES	***LPS-Cyto***	*AQ*	*AQ+MES*	*MES*
Small proline-rich protein 2D	SPRR2D	0.49*	0.44*	1.18	2.59*	0.70	0.50*	0.58*
Putative nucleoside diphosphate kinase	NME2P1	2.99*	1.90*	1.48*	2.39*	0.55*	0.75	0.60*
Nucleoplasmin-3	NPM3	1.99*	1.31	1.49	2.28*	0.37*	0.65	0.30*
Allograft inflammatory factor 1-like	AIF1L	1.33*	0.98	1.34*	2.00*	0.61*	0.64	1.17
Zinc-alpha-2-glycoprotein	AZGP1	0.88	1.21	1.26	2.00*	1.12	0.56*	1.08
Hepatoma-derived growth factor-related protein 3	HDGFL3	1.36*	1.48*	1.35*	1.86*	0.90	0.82	1.29
Protein max	MAX	1.28*	1.31	1.42*	1.85*	0.24*	0.39*	0.75
Putative heat shock protein HSP 90-alpha A4	HSP90AA4P	1.25	1.35	1.36	1.84*	0.78	0.94	1.30

Keratin, type I cytoskeletal 14	KRT14	0.51*	0.49*	0.57*	1.84*	0.71*	0.52*	0.61*
Keratin, type II cytoskeletal 6B	KRT6B	0.76*	0.70*	0.95	1.81*	1.04	0.91	1.11
DNA polymerase epsilon subunit 3	POLE3	1.42*	1.44	1.47	1.76*	1.10	0.98	0.64
Molybdopterin synthase sulfur carrier subunit	MOCS2	1.38	1.37	1.47*	1.73*	0.73	0.69	1.01
Keratin, type I cytoskeletal 17	KRT17	0.71*	0.64*	0.61*	1.72*	0.68	0.55*	0.77
Protein S100-A7	S100A7	0.33*	0.70	0.94	1.71*	0.60	1.01	1.30
N-acetyltransferase ESCO1	ESCO1	1.20	0.85	1.78*	1.70*	1.20	0.90	1.49
Actin-related protein 3B	ACTR3B	1.21*	1.28	1.27*	1.69*	1.17	1.10	1.14
Protein disulfide-isomerase A2	PDIA2	2.04*	1.32	1.24	1.68*	0.72	0.87	1.13
Transmembrane protein 115	TMEM115	1.15	1.37	1.11	1.67*	0.81	1.00	1.46
Keratin, type II cytoskeletal 5	KRT5	0.65*	0.63*	0.87	1.64*	1.09	0.72*	0.86
Keratin, type II cytoskeletal 6A	KRT6A	0.49*	1.00	1.23	1.61*	0.93	1.36	1.29
PC4 and SFRS1-interacting protein	PSIP1	1.51*	1.06	1.31*	1.61*	0.48*	0.47*	1.46*
Small nuclear ribonucleoprotein F	SNRPF	2.07*	1.62*	1.69*	1.59*	0.18*	0.89	0.54
Sodium-coupled monocarboxylate transporter 2	SLC5A12	1.14	1.13	0.87	1.59*	1.39	0.68	0.57
Keratin, type I cytoskeletal 16	KRT16	0.57*	0.74	0.81	1.57*	0.80	0.85	1.03
Arf-GAP with GTPase, ANK repeat and PH domain-containing protein 1	AGAP1	1.36	1.20	1.47	1.56	0.69	0.89	1.04
Nuclear transcription factor Y subunit alpha	NFYA	1.20	1.08	1.17	1.55*	0.52*	0.32*	0.82
tRNA-dihydrouridine(47) synthase [NAD(P)(+)]-like	DUS3L	1.03	1.35	1.33	1.55*	1.22	1.21	1.46
Protein TEX261	TEX261	1.42	1.46	1.20	1.55*	1.20	0.89	1.01
Syntaxin-10	STX10	1.17	1.05	1.20	1.54*	0.85	0.94	1.10
Tumor necrosis factor receptor superfamily member 6	FAS	1.10	1.01	1.19	1.54*	1.34	1.34	1.40*
Serum response factor-binding protein 1	SRFBP1	1.01	1.10	1.12	1.54*	0.80	0.63	1.07
Sodium- and chloride-dependent neutral and basic amino acid transporter B(0+)	SLC6A14	0.91	1.09	1.08	1.53*	1.41	1.38	1.44
C-C chemokine receptor-like 2	CCRL2	0.92	0.77	0.98	1.53	1.20	1.15	0.96
Cyclin-dependent kinase inhibitor 2A	CDKN2A	1.01	1.03	1.18	1.53*	0.32*	0.36*	0.49*
Small integral membrane protein 24	SMIM24	1.45	1.71*	0.90	1.51	1.28	1.22	0.90
Ubiquitin domain-containing protein 2	UBTD2	1.17	1.41	1.34	1.51	1.02	1.32	1.08
Lysosomal acid phosphatase	ACP2	0.92	1.08	1.06	1.51*	1.45*	1.41	1.35*
Solute carrier organic anion transporter family member 4A1	SLCO4A1	0.96	1.29	1.13	1.50*	1.27	1.02	1.32

C. Up-regulated proteins unique to Aquamin (AQ) with LPS-Cytokines [64 proteins]

		Interventions						
		Control			*With LPS & Cytokines*			
Proteins	Genes	AQ	AQ+MES	MES	*LPS-Cyto*	***AQ***	*AQ+MES*	*MES*

S-adenosyl-L-methionine-dependent tRNA 4-demethylwyosine synthase TYW1	TYW1	0.98	0.77	0.69*	1.30	4.94*	0.92	0.83
Haptoglobin	HP	0.70*	1.32*	3.72*	1.15	3.32*	1.47*	1.19
Peptidyl-tRNA hydrolase	PTRH1	1.02	1.23	1.02	1.25	2.58*	1.25	1.26
Arachidonate 12-lipoxygenase, 12R-type	ALOX12B	2.22*	1.37	7.85*	0.47*	2.58*	0.92	1.08
Keratin, type II cytoskeletal 1b	KRT77	0.89	0.80	0.63*	1.23	2.31*	0.70	1.26
Gasdermin-A	GSDMA	1.05	1.25	1.66*	1.00	2.19*	0.72	0.83
Membrane-spanning 4-domains subfamily A member 10	MS4A10	1.73*	2.21*	1.28	1.40	2.17*	1.20	1.03
Retinol-binding protein 2	RBP2	1.29*	2.84*	1.78*	1.20	2.02*	1.36*	1.25*
Keratin, type I cytoskeletal 9	KRT9	0.46*	0.44*	0.46*	1.11	1.99*	0.51*	0.89
Phosphatidylinositol 3-kinase regulatory subunit beta	PIK3R2	1.34	1.33	1.44	1.23	1.93*	1.27	1.19
Keratin, type II cytoskeletal 78	KRT78	0.71*	0.67*	1.26*	1.50*	1.92*	0.51*	1.49*
5'-nucleotidase domain-containing protein 3	NT5DC3	1.25*	1.00	0.86	1.42*	1.89*	1.46*	1.33
Sorbin and SH3 domain-containing protein 1	SORBS1	0.97	2.18*	1.43	1.40	1.86*	1.35	1.24
Transmembrane protein 125	TMEM125	1.53*	1.26	0.95	1.26	1.86*	0.98	1.15
Alpha-2-macroglobulin-like protein 1	A2ML1	1.27*	2.46*	5.56*	0.41*	1.85*	0.34*	1.36*
Nuclear factor 1 C-type	NFIC	0.55*	0.74	0.78	1.06	1.84*	1.42	1.30
Desmocollin-3	DSC3	1.72*	9.40*	34.18*	0.65	1.84*	0.93	0.01*
OTU domain-containing protein 4	OTUD4	0.76	0.99	0.99	1.05	1.83*	1.10	1.24
Aspartate dehydrogenase domain-containing protein	ASPDH	1.49*	2.23*	1.22	1.34	1.82*	1.07	1.07
Protein GPR108	GPR108	1.15	1.09	1.08	1.41*	1.81*	1.48*	1.38
Keratinocyte proline-rich protein	KPRP	0.80	0.87	1.38	1.41	1.80*	0.78	1.45
Signal peptide peptidase-like 3	SPPL3	1.03	0.85	1.23	0.96	1.79	1.17	1.29
F-box only protein 50	NCCRP1	1.73*	2.05*	3.97*	0.77	1.78*	0.88	1.38
Sphingosine-1-phosphate phosphatase 1	SGPP1	1.40	1.19	1.07	1.20	1.75*	1.45	1.05
Keratin, type II cytoskeletal 80	KRT80	2.20*	1.11	1.12	0.99	1.72*	0.99	1.07
NADH-ubiquinone oxidoreductase chain 2	MT-ND2	1.14	1.39	1.08	0.90	1.72*	1.19	0.90
Disheveled-associated activator of morphogenesis 1	DAAM1	0.91	0.92	0.98	1.23	1.71	1.47	1.34
Keratin, type I cytoskeletal 25	KRT25	0.44*	1.22	0.25*	0.41*	1.70*	0.23*	1.17
Cellular retinoic acid-binding protein 2	CRABP2	1.18	1.15	1.37*	1.37*	1.68*	1.45	1.34
Pseudouridylate synthase RPUSD4, mitochondrial	RPUSD4	0.89	0.95	0.90	0.83	1.66	0.63	0.78
Immediate early response 3-interacting protein 1	IER3IP1	0.61*	1.26	1.09	1.05	1.64*	1.16	0.89
Vitamin K-dependent gamma-carboxylase	GGCX	1.38*	1.37*	1.14	1.37*	1.64*	1.15	1.22
Maltase-glucoamylase	MGAM	1.32*	3.05*	1.31*	1.32*	1.62*	1.41*	0.94
Protein MMP24OS	MMP24OS	1.36	1.67*	1.29	1.11	1.61	1.43	1.33
Phosphatidylserine synthase 2	PTDSS2	1.40*	1.46*	1.39*	1.33	1.61	1.49	1.37
Protein OS-9	OS9	1.38*	1.38*	1.13	1.15	1.61*	1.43*	1.26
Nischarin	NISCH	0.87	1.04	0.87	1.24	1.60	1.39	1.49

Calcium/calmodulin-dependent protein kinase type 1B	PNCK	1.12	0.39*	0.14*	1.00	1.60	0.82	0.13*
Mediator of RNA polymerase II transcription subunit 24	MED24	0.92	0.82	0.82	1.25	1.60	1.43	1.45
Keratin, type II cytoskeletal 1	KRT1	0.42*	0.49*	0.57*	1.10	1.59*	0.44*	1.02
Leucine-rich repeat-containing G-protein coupled receptor 4	LGR4	0.77	0.86	0.82	0.89	1.59*	1.25	1.33
Dermokine	DMKN	0.47*	0.70	0.58*	0.64*	1.58	0.79	1.33
Alpha-1,3-mannosyl-glycoprotein 4-beta-N-acetylglucosaminyltransferase B	MGAT4B	1.01	1.01	0.99	1.09	1.58*	1.47	1.37
Ribonuclease P protein subunit p14	RPP14	1.25	1.10	1.01	1.35	1.57	1.38	1.26
Zinc transporter ZIP5	SLC39A5	1.47*	0.68	0.34*	1.24	1.57	0.61	0.44*
Myelin expression factor 2	MYEF2	0.70*	1.05	1.05	0.66*	1.57	1.32	1.36
Anaphase-promoting complex subunit 5	ANAPC5	0.39*	0.38*	0.39*	0.53*	1.56*	1.01	1.02
Golgi SNAP receptor complex member 2	GOSR2	1.03	1.20	1.10	1.29	1.55	1.38	1.34
Dual specificity tyrosine-phosphorylation-regulated kinase 1A	DYRK1A	0.66*	0.71*	0.76*	0.82	1.55*	1.35	1.36
3 beta-hydroxysteroid dehydrogenase type 7	HSD3B7	1.04	1.19	0.89	1.24	1.55	1.49	1.07
Rhomboid-related protein 4	RHBDD1	0.68	1.08	1.44	1.07	1.54	1.38	1.19
Sucrase-isomaltase, intestinal	SI	1.39*	1.36*	0.98	1.15	1.53*	1.07	0.72*
Transcobalamin-2	TCN2	1.28*	1.12	1.03	1.08	1.53*	1.20	0.84
Prolyl 4-hydroxylase subunit alpha-1	P4HA1	1.24*	1.28*	0.82*	1.11	1.53*	1.44*	0.97
Aminopeptidase RNPEPL1	RNPEPL1	1.36	0.89	0.84	1.26	1.52	0.89	0.98
Beta-1,4-N-acetylgalactosaminyltransferase 3	B4GALNT3	1.15	1.32	1.14	1.37	1.52	1.32	1.27
Transmembrane 4 L6 family member 20	TM4SF20	1.61*	0.93	0.65*	0.63*	1.51*	0.97	0.50*
SPARC-related modular calcium-binding protein 1	SMOC1	0.90	1.19	1.34*	1.42*	1.51	1.47	1.46
Galectin-3-binding protein	LGALS3BP	1.19	1.21	0.91	1.29*	1.51*	1.16	1.08
Dipeptidyl peptidase 4	DPP4	1.02	1.06	0.91	1.22*	1.51*	1.32*	1.17
Proline-rich protein 9	PRR9	1.25	15.97*	11.14*	0.74	1.51	0.43*	0.33*
SCAN domain-containing protein 3	SCAND3	1.06	0.93	1.12	1.20	1.51	1.44	1.37
Pumilio homolog 2	PUM2	1.00	1.01	0.78	1.00	1.51	1.05	1.00
CSC1-like protein 2	TMEM63B	1.10	1.23	1.04	1.14	1.50	1.23	1.21

D. Up-regulated proteins unique to Aquamin plus Mesalamine (AQ+MES) with LPS-Cytokines [69 proteins]

		Interventions						
		Control			*With LPS & Cytokines*			
Proteins	Genes	AQ	AQ+MES	MES	*LPS-Cyto*	*AQ*	***AQ+MES***	*MES*
Dynein axonemal heavy chain 8	DNAH8	0.65*	0.96	0.58*	0.19*	0.36*	2.06*	0.37*
Proteasome subunit beta type-3	PSMB3	1.04	1.36*	0.83	1.48*	1.15	2.03*	1.45*
Immunoglobulin heavy variable 3-49	IGHV3-49	0.94	2.96*	25.72*	0.91	1.41	2.01*	0.78

NEDD4 family-interacting protein 1	NDFIP1	0.94	1.39	0.98	1.05	1.44	1.98*	0.90
Proteasome subunit alpha type-7	PSMA7	1.12	1.30*	1.38*	1.33*	1.25	1.96*	1.35*
Dixin	DIXDC1	1.10	1.59*	1.27	1.43	0.87	1.95*	1.47
Alpha-tocopherol transfer protein	TTPA	0.89	1.02	1.13	1.20	1.39	1.93*	1.21
55 kDa erythrocyte membrane protein	MPP1	1.16	2.80*	1.47*	1.10	1.36	1.92*	1.21
Proteasome subunit alpha type-4	PSMA4	1.07	1.25*	1.27*	1.30*	1.14	1.92*	1.32*
Proteasome subunit alpha type-2	PSMA2	1.10	1.16	1.12	1.40*	1.18	1.92*	1.37*
Proteasome subunit beta type-2	PSMB2	1.36*	1.44*	1.27*	1.28	1.23	1.90*	1.42*
V-type proton ATPase 116 kDa subunit a 2	ATP6V0A2	0.97	1.32	1.43	1.11	1.37	1.88*	1.45
Trehalase	TREH	1.60*	2.35*	1.52*	0.90	1.42*	1.88*	1.13
Phosphatidylethanolamine N-methyltransferase	PEMT	1.16	1.56	1.27	1.00	1.29	1.88*	1.29
Inositol-trisphosphate 3-kinase C	ITPKC	1.17	2.04*	1.95*	1.21	1.37	1.86*	1.46
Acyl-CoA:lysophosphatidylglycerol acyltransferase 1	LPGAT1	1.10	1.50*	1.18	1.37*	1.49*	1.82*	1.33
Proteasome subunit beta type-4	PSMB4	1.07	1.17	1.33*	1.33*	1.24	1.80*	1.33*
Enhancer of filamentation 1	NEDD9	1.46*	1.77*	1.30	0.93	1.23	1.75*	0.91
7-methylguanosine phosphate-specific 5'-nucleotidase	NT5C3B	1.45*	1.37	1.15	1.06	0.83	1.75	0.44*
WD repeat and SOCS box-containing protein 2	WSB2	1.15	1.43	1.24	1.40	1.21	1.73	1.48
Proteasome subunit alpha type-3	PSMA3	1.10	1.20	1.15*	1.20	1.04	1.72*	1.22
E3 ubiquitin-protein ligase TRIM31	TRIM31	0.95	1.22	1.36	1.26	0.88	1.72	1.39
Proteasome subunit alpha type-1	PSMA1	1.10	1.22	1.30*	1.35*	1.16	1.72*	1.30*
Proteasome subunit alpha type-6	PSMA6	1.03	1.12	1.33*	1.41*	1.09	1.70*	1.26*
Tigger transposable element-derived protein 3	TIGD3	0.93	1.74*	0.94	1.15	1.30	1.69	1.19
Mediator of RNA polymerase II transcription subunit 11	MED11	1.14	1.04	1.11	0.99	1.25	1.69	0.78
Multiple coagulation factor deficiency protein 2	MCFD2	1.48*	1.46*	1.12	0.99	1.45	1.68*	1.23
Interferon regulatory factor 1	IRF1	0.85	1.36	1.21	0.90	0.93	1.68	1.24
Reticulophagy regulator 3	RETREG3	0.98	0.97	1.05	1.04	1.35	1.68*	1.18
Immunoglobulin heavy constant gamma 4	IGHG4	0.64*	1.95*	32.94*	0.66*	0.72	1.67*	0.96
Ceramide glucosyltransferase	UGCG	1.26	1.37	1.32	1.24	1.11	1.67	1.22
Hyaluronan-binding protein 2	HABP2	0.99	1.28	1.36	0.93	1.22	1.66	1.45
Lactadherin	MFGE8	1.13	1.35*	1.35*	1.26	1.45*	1.66*	1.32
Dual oxidase maturation factor 2	DUOXA2	1.45*	1.75*	1.60*	1.11	1.43	1.63*	1.04
Proteasome subunit beta type-1	PSMB1	1.20	1.23	1.18	1.22	1.03	1.63*	1.14
Solute carrier family 66 member 3	SLC66A3	1.36	2.04*	1.50*	0.98	1.35	1.63	1.02
E3 ubiquitin ligase RNF121	RNF121	1.22	1.65*	1.28	1.18	1.43	1.63	1.17
Lysophospholipid acyltransferase 1	MBOAT1	1.35	1.09	1.16	1.16	1.22	1.62	1.21
Prenylated Rab acceptor protein 1	RABAC1	0.98	1.19	1.32	1.22	1.17	1.62	1.41
Alkaline phosphatase, placental type	ALPP	0.98	0.67*	0.93	0.51*	1.19	1.61	1.36

Rap guanine nucleotide exchange factor 2	RAPGEF2	1.37	1.49	0.93	1.01	1.40	1.61	0.84
Interferon-stimulated gene 20 kDa protein	ISG20	1.14	1.25	1.35*	1.12	1.39	1.60*	1.46*
Toll-like receptor 3	TLR3	1.17	1.23	0.89	1.46*	1.46*	1.60*	1.24
Proteasome subunit alpha type-5	PSMA5	0.97	1.09	1.21*	1.23*	1.09	1.60*	1.16
High affinity copper uptake protein 1	SLC31A1	1.36*	1.51*	1.17	1.17	1.11	1.59*	1.16
Protein RFT1 homolog	RFT1	1.24	1.24	1.18	1.31	1.43	1.58	1.48
Peroxisomal membrane protein PEX13	PEX13	1.03	1.23	1.21	1.15	1.27	1.57	1.42
2'-5'-oligoadenylate synthase 1	OAS1	1.13	1.17	0.95	1.43*	1.33	1.57*	1.27
Transmembrane channel-like protein 5	TMC5	1.15	1.31	1.32	1.32	1.47	1.57	1.31
Nicotinamide phosphoribosyltransferase	NAMPT	1.04	1.10	1.09	1.39*	1.41*	1.56*	1.39*
Ectonucleotide pyrophosphatase/phosphodiesterase family member 1	ENPP1	0.97	1.08	1.27	1.06	1.35	1.56	1.27
Nuclear pore complex protein Nup160	NUP160	0.94	1.01	0.89	1.13	1.30	1.56	1.27
Alanine aminotransferase 2	GPT2	1.19	1.45	1.18	1.43	1.40	1.56	1.21
Alsin	ALS2	1.51*	1.57	1.67*	1.30	1.37	1.56	1.50
Elongation of very long chain fatty acids protein 5	ELOVL5	1.28	1.30	1.17	1.23	1.30	1.56	1.28
Cytokine receptor common subunit gamma	IL2RG	1.27	1.67*	1.27	1.28	1.27	1.55	0.97
Lipase maturation factor 2	LMF2	1.07	1.27	1.17	1.31	1.26	1.55	1.33
Intestinal-type alkaline phosphatase	ALPI	1.25	1.60*	1.15	1.01	1.45	1.54	1.09
Electrogenic sodium bicarbonate cotransporter 1	SLC4A4	1.22	1.21	1.08	1.27	1.30	1.54	1.28
ORM1-like protein 3	ORMDL3	1.30*	1.34	1.28	1.35	1.36	1.54	1.44
H(+)/Cl(-) exchange transporter 7	CLCN7	1.13	1.48*	1.11	1.41	1.49	1.54	1.17
Immunoglobulin heavy variable 3-7	IGHV3-7	0.93	2.12*	23.53*	0.83	1.48	1.53	0.58
Mucin-4	MUC4	1.19	0.88	0.94	1.35	1.50	1.53	1.29
Anion exchange protein 2	SLC4A2	0.96	1.19	0.95	1.45*	1.49	1.52*	1.10
Transmembrane protein 184B	TMEM184B	0.97	0.97	1.02	1.10	1.29	1.52	1.19
Torsin-1B	TOR1B	1.03	1.29	1.11	1.18	1.41	1.51*	1.42
Translocating chain-associated membrane protein 1	TRAM1	1.18	1.17	1.29	1.23	1.36	1.51*	1.34
Transmembrane 7 superfamily member 3	TM7SF3	0.93	0.97	0.61*	1.41*	1.17	1.51	1.23
Protein unc-93 homolog B1	UNC93B1	1.21	1.29	1.14	1.29	1.42	1.50*	1.22

E. Up-regulated proteins unique to Mesalamine (MES) with LPS-Cytokines [84 proteins]

		Interventions						
		Control			*With LPS & Cytokines*			
Proteins	Genes	AQ	AQ+MES	MES	*LPS-Cyto*	*AQ*	*AQ+MES*	***MES***
Keratin, type II cuticular Hb4	KRT84	3.41*	1.07	1.03	1.40	0.95	1.04	3.69*
Keratin, type I cuticular Ha4	KRT34	0.84	63.49*	2.24*	0.74	1.05	0.91	3.16*

EF-hand domain-containing protein D1	EFHD1	0.66	43.85*	1.91*	0.69	0.69	0.84	3.11*
Eukaryotic peptide chain release factor GTP-binding subunit ERF3B	GSPT2	0.82	0.93	1.07	1.39	0.95	0.85	3.07*
Keratin, type I cuticular Ha1	KRT31	0.68*	49.23*	2.04*	0.65*	0.92	0.80	2.96*
Keratin, type I cuticular Ha3-I	KRT33A	0.87	24.84*	1.71*	0.94	0.91	1.29	2.44*
Dual specificity protein phosphatase 12	DUSP12	0.92	0.94	0.94	1.02	1.45	1.43	2.31*
Thrombospondin-1	THBS1	0.92	0.96	1.17	1.23	1.07	1.45*	2.29*
Mitotic-spindle organizing protein 1	MZT1	1.14	1.09	2.73*	0.92	0.63	0.71	2.13*
tRNA methyltransferase 10 homolog A	TRMT10A	0.87	1.03	1.66*	0.99	1.01	1.30	2.12*
Spliceosome-associated protein CWC27 homolog	CWC27	2.02*	1.81*	1.94*	1.33	0.51	0.99	2.11*
Retrotransposon-derived protein PEG10	PEG10	0.68	0.94	1.32	1.06	1.31	1.39	2.08*
Retinoic acid receptor responder protein 2	RARRES2	0.54*	1.61*	2.51*	0.66	0.68	1.46	2.08*
Cell migration-inducing and hyaluronan-binding protein	CEMIP	0.69*	0.80	0.90	1.04	1.29	1.30	2.08*
Nucleoporin Nup43	NUP43	1.06	1.34	1.38*	1.45*	1.26	1.43	2.05*
Fibronectin	FN1	0.81	1.09	1.21	1.17	0.99	1.39	1.99*
Serine protease HTRA1	HTRA1	0.84	1.16	1.55*	0.94	1.32	1.42	1.99*
Lysozyme g-like protein 2	LYG2	0.84	31.25*	1.48	0.85	1.13	1.33	1.98*
Vimentin	VIM	0.73	1.24	1.17	1.24	1.45	1.29	1.96*
Thymosin beta-4	TMSB4X	0.55*	0.94	1.71*	1.20	1.01	1.17	1.93*
Collagen alpha-3(VI) chain	COL6A3	0.29*	0.50*	1.58*	1.31	0.81	1.25	1.92*
Ferroxidase HEPHL1	HEPHL1	0.77	61.07*	3.28*	0.93	1.02	0.31*	1.91
Ribosomal protein eL22-like	RPL22L1	0.45*	0.89	1.44	0.86	0.54	1.05	1.90*
Histone H2B type 3-B	H2BC26	0.72	1.01	1.20	1.26	1.42	1.21	1.87*
High mobility group protein HMG-I/HMG-Y	HMGA1	0.50*	0.61*	1.13	1.38*	1.08	1.34	1.87*
Pregnancy zone protein	PZP	0.57*	1.21	1.00	1.42	0.69	1.38	1.86
Exostosin-2	EXT2	1.09	1.51*	1.42*	1.25	1.38	1.43	1.85*
Beta-1,4-glucuronyltransferase 1	B4GAT1	0.84	1.35	1.30	1.08	1.38	1.22	1.79
Syntaxin-binding protein 1	STXBP1	0.71	1.08	1.38	0.90	1.16	1.49	1.77*
Tripartite motif-containing protein 14	TRIM14	0.85	1.01	0.96	1.21	1.16	1.07	1.77*
Kynureninase	KYNU	0.82	1.00	1.61*	0.52*	0.92	1.21	1.74*
Testis-expressed protein 10	TEX10	0.89	1.21	0.82	1.30	1.49	1.41	1.74
Nephronectin	NPNT	0.57*	0.57*	0.73	0.71	1.35	1.21	1.73
Ribonucleoprotein PTB-binding 2	RAVER2	0.86	1.47	1.25	0.59*	0.57	0.57	1.71
ICOS ligand	ICOSLG	1.00	1.25	1.37	1.26	1.24	1.50	1.70
Iron-sulfur cluster assembly 1 homolog, mitochondrial	ISCA1	0.81	1.20	1.48*	0.84	0.99	1.35	1.68*
Palmitoyltransferase ZDHHC20	ZDHHC20	1.12	1.18	1.15	1.29	1.42	1.35	1.68*
4-hydroxyphenylpyruvate dioxygenase-like protein	HPDL	1.20	1.03	0.99	1.34	1.30	1.29	1.68
C4b-binding protein alpha chain	C4BPA	0.48*	1.00	1.41	1.50*	1.06	1.25	1.68

Cohesin subunit SA-1	STAG1	0.98	1.01	0.97	1.44	1.28	1.35	1.67
Arylacetamide deacetylase	AADAC	1.04	1.62	1.20	1.20	1.10	1.11	1.66
Dermcidin	DCD	0.64*	0.68*	0.85	0.99	1.23	0.95	1.65*
Peroxidasin homolog	PXDN	1.04	1.13	1.31	1.18	1.11	1.39	1.65*
Neurogenic locus notch homolog protein 2	NOTCH2	0.99	1.26	1.38	1.49	1.28	1.28	1.65
Transcription elongation factor SPT4	SUPT4H1	1.14	1.63*	0.98	1.44	1.15	1.33	1.64
Proprotein convertase subtilisin/kexin type 5	PCSK5	0.95	1.00	0.81	1.15	1.02	0.84	1.63
Serine/threonine-protein kinase 3	STK3	0.70	0.78	1.01	1.18	1.09	0.86	1.62
Nurim	NRM	1.27	0.90	1.16	1.15	1.00	1.40	1.62
FACT complex subunit SSRP1	SSRP1	0.93	0.95	1.07	1.26	1.01	1.03	1.59*
Caspase-7	CASP7	1.04	1.26	1.14	1.46*	1.44	1.46	1.59*
Sideroflexin-4	SFXN4	0.93	1.26	1.05	1.30*	1.46*	1.48*	1.59*
Urokinase-type plasminogen activator	PLAU	0.96	1.36	1.17	1.36	1.32	1.42	1.58
N-acetylglucosamine-1-phosphodiester alpha-N-acetylglucosaminidase	NAGPA	0.82	1.28	1.80*	0.75	0.93	1.45	1.58
Myosin-10	MYH10	0.88	0.74*	0.95	1.01	0.91	1.19	1.58*
AT-rich interactive domain-containing protein 1A	ARID1A	0.99	1.20	1.04	1.28	1.26	1.36	1.58
Vesicle transport protein SEC20	BNIP1	1.14	1.27	1.23	1.08	1.14	1.18	1.58
Trafficking protein particle complex subunit 2-like protein	TRAPPC2L	0.96	1.06	1.01	0.93	1.05	1.32	1.57
Sodium-dependent lysophosphatidylcholine symporter 1	MFSD2A	1.38	1.71*	1.13	1.34	0.87	1.25	1.57
Mitochondrial import inner membrane translocase subunit Tim10 B	TIMM10B	1.17	1.25	0.96	1.50*	1.33	1.43	1.56
Insulin-like growth factor-binding protein 4	IGFBP4	0.88	1.45*	1.57*	0.83	0.89	1.38	1.56
Glutathione peroxidase 2	GPX2	1.00	1.03	1.06	1.29	1.25	1.30	1.55*
ADP-ribosylation factor GTPase-activating protein 1	ARFGAP1	0.99	1.39*	1.38*	1.49*	1.20	1.21	1.55*
Golgin-45	BLZF1	0.88	1.13	1.10	1.05	1.08	0.98	1.54
5'-nucleotidase domain-containing protein 2	NT5DC2	0.93	1.08	1.18	0.94	1.22	1.20	1.54*
Ribosome production factor 1	RPF1	0.83	0.67*	0.93	1.42*	1.40	1.26	1.54
Inter-alpha-trypsin inhibitor heavy chain H1	ITIH1	0.48*	0.96	1.54*	0.48*	0.48*	1.13	1.53
Alpha-methylacyl-CoA racemase	AMACR	1.35	1.27	1.07	1.26	1.21	1.18	1.53
Histone H1.2	H1-2	0.96	0.74	1.15	0.79	0.98	0.88	1.53*
Tumor necrosis factor receptor superfamily member 12A	TNFRSF12A	0.88	1.46	1.75*	1.47	1.35	1.30	1.52
Multiple inositol polyphosphate phosphatase 1	MINPP1	1.08	1.23	1.32*	1.49*	1.06	1.23	1.52
Homeobox protein CDX-2	CDX2	1.29	1.40	1.36	1.43	0.92	1.24	1.52
ATP-binding cassette sub-family B member 10, mitochondrial	ABCB10	1.07	1.30	1.13	1.34	1.48	1.23	1.52
Syndecan-4	SDC4	1.04	0.89	1.12	1.48*	1.49*	1.32	1.52*
Non-histone chromosomal protein HMG-17	HMGN2	0.56*	1.04	1.61*	0.87	0.80	1.21	1.52

mRNA-decapping enzyme 1B	DCP1B	1.26	2.01*	1.92*	0.58*	1.35	0.76	1.52
ATP-dependent DNA/RNA helicase DHX36	DHX36	0.77*	0.80	0.81	0.93	1.17	1.34	1.52*
Tsukushi	TSKU	0.99	1.13	1.15	1.08	1.43	1.47	1.51
Apolipoprotein B-100	APOB	0.77	1.20	1.37	1.26	1.18	1.26	1.51
CDK-activating kinase assembly factor MAT1	MNAT1	0.53*	0.93	1.05	0.79	0.52	0.89	1.51
UDP-glucuronic acid decarboxylase 1	UXS1	1.12	1.22	1.09	1.06	1.42	1.45	1.51
GRIP1-associated protein 1	GRIPAP1	1.01	0.97	1.28	1.16	1.12	1.26	1.51
Serpin H1	SERPINH1	1.08	1.21	1.03	1.15	1.29	1.30*	1.51*
Sodium-dependent multivitamin transporter	SLC5A6	1.09	1.02	1.02	1.41*	1.39	1.46	1.50*
Cell division cycle protein 27 homolog	CDC27	1.11	0.99	1.00	0.98	0.96	1.19	1.50

F. Common up-regulated proteins between LPS-Cytokines alone and with Aquamin [17 proteins]

		Interventions						
		Control			*With LPS & Cytokines*			
Proteins	Genes	AQ	AQ+MES	MES	***LPS-Cyto***	***AQ***	*AQ+MES*	*MES*
Insulin-like growth factor-binding protein 1	IGFBP1	1.38	0.82	0.97	3.49*	1.56	1.39	1.48
Interferon-related developmental regulator 1	IFRD1	2.60*	0.80	1.96*	3.12*	1.60	1.01	0.77
Glutathione S-transferase A2	GSTA2	1.36*	0.40*	0.10*	2.33*	1.56*	0.19*	0.50*
Proline-rich acidic protein 1	PRAP1	1.21	1.06	0.84	2.23*	2.24*	1.21	1.08
Bromodomain-containing protein 3	BRD3	1.18	1.44	1.36	1.92*	1.62	1.09	1.42
Formin-binding protein 4	FNBP4	0.67	1.03	0.79	1.91*	1.61	1.38	1.42
Male-enhanced antigen 1	MEA1	1.28	1.17	1.37	1.81*	1.59	1.28	1.50
Loricrin	LORICRIN	0.48*	0.44*	0.64*	1.79*	1.63*	0.30*	1.10
Sodium-dependent neutral amino acid transporter B(0)AT1	SLC6A19	1.63*	2.12*	0.93	1.71*	1.74*	1.12	0.47*
Dynein axonemal light chain 1	DNAL1	0.94	1.27	1.25	1.69*	2.16*	1.29	1.48
Myeloid-associated differentiation marker	MYADM	1.30	1.03	1.17	1.67*	1.60	1.06	1.35
Deleted in malignant brain tumors 1 protein	DMBT1	2.24*	0.89	1.16*	1.67*	1.57*	0.60*	0.73*
Matrilysin	MMP7	1.52*	1.02	1.05	1.66*	1.74*	1.24	1.42*
Cytochrome P450 2C19	CYP2C19	2.12*	1.15	0.61*	1.64*	1.98*	0.88	0.62
EKC/KEOPS complex subunit TPRKB	TPRKB	1.13	1.46	1.16	1.53*	1.62	1.15	1.49
F-box only protein 28	FBXO28	0.94	1.19	1.24	1.52	1.68	1.38	1.44
2-amino-3-carboxymuconate-6-semialdehyde decarboxylase	ACMSD	1.49*	1.40	0.84	1.51*	1.53	1.09	1.10

G. Common up-regulated proteins between LPS-Cytokines alone and with Aquamin plus Mesalamine [2 proteins]

Interventions

		Control			*With LPS & Cytokines*			
Proteins	Genes	AQ	AQ+MES	MES	***LPS-Cyto***	*AQ*	***AQ+MES***	*MES*
Threonylcarbamoyl-AMP synthase	YRDC	1.20	1.69*	1.25	1.89*	0.94	1.91*	1.10
Zinc transporter ZIP10	SLC39A10	1.20	1.29	1.24	1.67*	1.02	1.68	1.37

H. Common up-regulated proteins between LPS-Cytokines alone and with Mesalamine [18 proteins]

		Interventions						
		Control			*With LPS & Cytokines*			
Proteins	Genes	AQ	AQ+MES	MES	***LPS-Cyto***	*AQ*	*AQ+MES*	***MES***
Integrin alpha-7	ITGA7	0.48*	0.78	0.74	5.21*	0.84	0.49*	1.76
Non-histone chromosomal protein HMG-14	HMGN1	1.16	0.60*	0.78	3.08*	1.07	1.10	2.25*
Protein-glutamine gamma-glutamyltransferase E	TGM3	1.03	1.11	0.97	2.43*	1.47*	1.08	1.56*
Transcription factor 20	TCF20	0.66*	0.99	1.00	2.22*	0.80	0.51	1.59
Fibrinogen alpha chain	FGA	0.63*	0.68	2.55*	2.12*	0.76	1.05	2.43*
Arrestin-C	ARR3	0.73	1.04	1.01	1.99*	1.12	0.86	1.82
Bleomycin hydrolase	BLMH	1.70*	1.51*	1.70*	1.98*	1.46	1.45	1.59
Zinc finger protein 284	ZNF284	1.90*	1.45	1.70*	1.88*	0.68	0.99	2.29*
Protein FAM193A	FAM193A	0.75	0.87	1.40	1.83*	0.96	1.32	1.98*
Transmembrane protein 179B	TMEM179B	1.32	1.24	1.18	1.82*	1.44	1.48	1.67*
Ankyrin repeat domain-containing protein 27	ANKRD27	1.10	1.61*	1.51*	1.81*	1.46*	1.06	1.92*
ABC-type oligopeptide transporter ABCB9	ABCB9	0.40*	0.67	1.05	1.77*	0.59	0.93	2.42*
Ferritin heavy chain	FTH1	0.99	0.81	1.35*	1.72*	1.29	1.24	1.97*
tRNA (guanine-N(7)-)-methyltransferase	METTL1	1.03	1.27	1.33	1.70*	1.32	1.25	1.80*
Immortalization up-regulated protein	IMUP	0.66*	1.26	1.81*	1.60*	1.16	1.10	1.75*
Serine/threonine-protein kinase 4	STK4	0.98	1.00	1.13	1.56*	1.42	1.45	1.60*
Protein S100-A9	S100A9	0.60*	1.51*	1.41*	1.53*	1.35	1.39	2.46*
Epsin-2	EPN2	1.54*	1.34*	1.44*	1.52*	1.37	1.38	1.54*

I. Common up-regulated proteins among LPS-Cytokines alone, with Aquamin and with Aquamin plus Mesalamine [9 proteins]

		Interventions						
		Control			*With LPS & Cytokines*			
Proteins	Genes	AQ	AQ+MES	MES	***LPS-Cyto***	***AQ***	***AQ+MES***	*MES*
Peroxisome assembly protein 12	PEX12	1.11	1.29	1.00	2.05*	2.02*	1.96*	1.41
Complement component C8 gamma chain	C8G	1.96*	2.13*	4.51*	1.81*	2.79*	1.96*	1.26
Apolipoprotein L6	APOL6	1.33	1.18	0.80	1.70*	2.00*	2.15*	1.29
Gamma-interferon-inducible protein 16	IFI16	1.10	1.19	1.04	1.62*	1.62	1.69*	1.36
Ferredoxin-2, mitochondrial	FDX2	1.78*	1.45	1.55*	1.59*	2.48*	1.91*	1.27

NK-tumor recognition protein	NKTR	1.53*	1.80*	1.57	1.59	1.50	1.67	1.19
Serine protease inhibitor Kazal-type 1	SPINK1	0.67	1.40	1.08	1.56*	1.71*	1.57	1.39
Armadillo repeat-containing protein 2	ARMC2	1.29	1.30	1.14	1.55*	1.88*	1.86*	1.46
Ileal sodium/bile acid cotransporter	SLC10A2	1.33*	2.98*	1.64*	1.53*	1.67*	2.16*	1.42

J. Common up-regulated proteins among LPS-Cytokines alone, with Aquamin and with Mesalamine [6 proteins]

		Interventions						
		Control			*With LPS & Cytokines*			
Proteins	Genes	AQ	AQ+MES	MES	***LPS-Cyto***	***AQ***	*AQ+MES*	***MES***
Apolipoprotein D	APOD	1.10	2.13*	1.32	4.26*	2.98*	1.00	2.56*
Prolactin-inducible protein	PIP	0.90	1.33*	1.20	3.19*	2.97*	0.93	2.67*
Secreted Ly-6/uPAR domain-containing protein 2	SLURP2	0.62*	0.79	0.42*	2.19*	4.45*	1.19	3.77*
Electron transfer flavoprotein regulatory factor 1	ETFRF1	1.41*	1.71*	1.48*	1.68*	1.53	1.47	1.73*
U3 small nucleolar RNA-associated protein 15 homolog	UTP15	0.85	0.85	0.86	1.57*	1.57*	1.37	1.90*
Prostatic acid phosphatase	ACP3	1.83*	8.61*	2.97*	1.52*	2.09*	1.08	1.62

K. Common up-regulated proteins among LPS-Cytokines alone, with Aquamin plus Mesalamine and with Mesalamine [29 proteins]

		Interventions						
		Control			*With LPS & Cytokines*			
Proteins	Genes	AQ	AQ+MES	MES	***LPS-Cyto***	*AQ*	***AQ+MES***	***MES***
Collagen alpha-6(IV) chain	COL4A6	0.76	1.01	1.42	2.54*	0.98	1.86*	2.68*
Nuclear receptor coactivator 6	NCOA6	2.02*	3.40*	3.87*	2.35*	0.89	2.98*	4.19*
CD166 antigen	ALCAM	1.12	1.95*	1.74*	2.10*	1.22	2.14*	1.78*
Interferon-induced protein with tetratricopeptide repeats 5	IFIT5	1.04	1.34	1.60*	2.00*	1.09	1.96*	2.05*
Midkine	MDK	0.80	1.07	1.17	1.97*	1.39	1.68*	1.69*
Ferritin light chain	FTL	0.52*	2.72*	3.90*	1.95*	0.58	4.12*	9.36*
Antithrombin-III	SERPINC1	0.69*	1.19	1.55*	1.84*	1.44*	1.87*	2.27*
Alpha-2-HS-glycoprotein	AHSG	0.70*	1.45*	1.73*	1.82*	1.47*	2.35*	2.40*
Bis(5'-adenosyl)-triphosphatase	FHIT	1.09	1.58*	1.32	1.78*	1.32	1.52	1.77*
Mitoferrin-2	SLC25A28	1.83*	3.25*	3.19*	1.78*	1.29	3.96*	2.50*
Apolipoprotein L5	APOL5	1.57*	0.98	1.90*	1.78*	1.11	1.62	1.76
N-myc-interactor	NMI	1.08	1.13	1.26*	1.73*	1.34	1.52*	1.63*
MARVEL domain-containing protein 3	MARVELD3	1.31	1.82*	1.63*	1.72*	0.97	1.89*	1.56
Kininogen-1	KNG1	0.47*	2.27*	2.73*	1.71*	0.68	2.29*	2.32*
Opioid growth factor receptor	OGFR	1.32*	2.03*	1.72*	1.66*	1.39	1.91*	2.03*
NEDD8 ultimate buster 1	NUB1	0.87	1.39*	1.34*	1.66*	0.92	1.60*	1.55*

Cilia- and flagella-associated protein 100	CFAP100	0.89	1.31	1.78*	1.65*	1.48	2.37*	2.52*
Gasdermin-B	GSDMB	0.99	1.13	1.35*	1.64*	0.98	1.59	1.64*
Stomatin	STOM	1.08	1.18	1.06	1.62*	1.50	1.71*	1.65*
Phospholipid scramblase 1	PLSCR1	0.56*	1.04	1.09	1.60*	0.94	1.55	1.76*
Collagen alpha-1(II) chain	COL2A1	1.18	4.35*	5.26*	1.58*	1.09	3.94*	5.38*
Collagen alpha-1(XV) chain	COL15A1	0.83	1.81*	1.43	1.57	1.31	1.62	2.31*
G patch domain-containing protein 8	GPATCH8	0.78	0.72	1.11	1.55*	1.25	1.53	1.63
Putative sodium-coupled neutral amino acid transporter 10	SLC38A10	1.01	1.36	1.38	1.55*	1.41	1.86*	1.55
Protein C19orf12	C19orf12	1.61*	2.28*	1.77*	1.55	1.49	1.94*	1.86
Transcription factor ETV6	ETV6	1.90*	1.88*	2.40*	1.54*	1.45	2.02*	1.81
Apolipoprotein A-IV	APOA4	0.95	3.94*	8.02*	1.53*	1.25	2.60*	3.78*
ATP-binding cassette sub-family C member 2	ABCC2	1.50*	2.42*	1.43*	1.53*	1.48*	1.89*	1.60*
GATOR complex protein MIOS	MIOS	1.18	1.06	1.20	1.50*	1.47	1.61	2.16*

L. Common up-regulated proteins between LPS-Cytokines with Aquamin and with Aquamin+Mesalamine [50 proteins]

		Interventions						
		Control			*With LPS & Cytokines*			
Proteins	Genes	AQ	AQ+MES	MES	*LPS-Cyto*	***AQ***	***AQ+MES***	*MES*
Cadherin-17	CDH17	3.59*	3.47*	1.05	0.91	2.69*	2.67*	0.73*
Phosphatidylinositol 4-phosphate 5-kinase type-1 beta	PIP5K1B	1.11	1.19	0.97	0.79	2.66*	2.81*	1.27
Copine-8	CPNE8	1.21*	1.04	0.87	1.35*	2.62*	2.32*	1.37*
Fibronectin type III and SPRY domain-containing protein 1	FSD1	3.62*	3.96*	1.02	0.70	2.52*	1.94*	0.49*
Poly(ADP-ribose) glycohydrolase	PARG	1.16	1.02	0.74	1.29	2.51*	1.58	0.89
Lipase member H	LIPH	1.16	1.37	1.18	1.37	2.38*	2.40*	1.48
Calcium/manganese antiporter SLC30A10	SLC30A10	3.21*	2.20*	1.22	1.36	2.24*	2.25*	0.82
Sodium/hydrogen exchanger 2	SLC9A2	1.36*	1.35	1.38*	1.10	2.18*	1.67	1.28
Desmoglein-2	DSG2	2.17*	2.21*	0.93	1.08	2.03*	2.03*	1.45*
Protein FAM3A	FAM3A	1.18	1.25	1.22	1.39*	2.02*	1.88*	1.48*
Protocadherin-1	PCDH1	2.10*	2.09*	1.14	0.88	1.93*	1.95*	1.13
Very low-density lipoprotein receptor	VLDLR	1.58*	1.36	1.44*	1.24	1.93*	1.56	1.43
Sphingolipid delta(4)-desaturase DES1	DEGS1	1.29	1.47	1.22	1.39	1.87*	1.96*	1.45
Olfactory receptor 1M1	OR1M1	1.48*	1.95*	1.32	1.06	1.87*	2.10*	1.19
Copine-2	CPNE2	1.17	1.35	1.18	1.26	1.86*	1.71	1.26
Xaa-Pro aminopeptidase 2	XPNPEP2	2.00*	2.97*	1.79*	1.17	1.85*	1.67*	1.38
Synaptotagmin-7	SYT7	0.99	1.23	1.00	0.99	1.84*	1.91*	0.91
H(+)/Cl(-) exchange transporter 5	CLCN5	1.32	1.34	1.07	1.43	1.83*	1.83*	1.19

Glutamine synthetase	GLUL	1.31*	1.26	1.08	1.40*	1.83*	1.78*	1.38*
Pterin-4-alpha-carbinolamine dehydratase 2	PCBD2	1.71*	1.26	1.07	1.40	1.81*	1.88*	1.22
Protein PALS2	PALS2	0.98	0.52*	0.71	0.52*	1.81*	1.54	1.29
Ankyrin-2	ANK2	1.33	1.30	1.15	1.25	1.79*	1.60	1.09
Melanotransferrin	MELTF	1.46*	1.86*	1.26	1.24	1.78	1.93*	1.35
Integrin alpha-5	ITGA5	1.11	1.46*	1.46*	1.08	1.78*	1.74*	1.18
Galactosylgalactosylxylosylprotein 3-beta-glucuronosyltransferase 3	B3GAT3	1.46*	1.65*	1.29	1.47*	1.77	2.12*	1.07
Cytochrome c oxidase assembly protein COX18, mitochondrial	COX18	1.09	0.91	0.92	1.27	1.76*	1.54	1.47
Protein SERAC1	SERAC1	1.48*	1.37	1.03	1.09	1.72	1.53	1.18
Solute carrier family 52, riboflavin transporter, member 3	SLC52A3	1.21	1.54	1.45	1.29	1.72	1.52	1.49
Neuropilin-2	NRP2	1.63*	1.43	1.45*	1.26	1.69*	1.56	1.33
Heme transporter HRG1	SLC48A1	1.02	1.24	0.93	1.23	1.68	1.75*	1.35
Elongation factor 1-alpha 2	EEF1A2	0.84	1.52*	1.19	1.02	1.66*	1.71*	1.43
Protein CLN8	CLN8	1.36	1.43	1.29	1.34	1.66	1.67	1.33
Myosin light chain 6B	MYL6B	0.56*	0.71*	0.75	0.80	1.66*	1.68*	1.15
Signal peptide peptidase-like 2A	SPPL2A	1.09	1.25	0.96	1.40	1.65*	1.53	1.19
CD82 antigen	CD82	0.79	0.90	1.01	1.06	1.64	1.59	1.20
Osteopetrosis-associated transmembrane protein 1	OSTM1	1.28	1.27	1.19	1.35	1.64	1.67	1.34
Lathosterol oxidase	SC5D	1.20	1.17	1.09	1.35	1.63	1.68	1.44
Tetratricopeptide repeat protein 22	TTC22	1.20	1.09	1.32	1.46	1.62	1.58	1.21
Solute carrier family 53 member 1	XPR1	1.58*	1.51	1.38	1.17	1.61	1.70*	1.32
Sushi domain-containing protein 2	SUSD2	1.30	2.95*	1.70*	1.09	1.57	1.60	0.92
Glutaminyl-peptide cyclotransferase-like protein	QPCTL	1.26	1.86*	1.14	1.24	1.57	1.58	1.49
UDP-GlcNAc:betaGal beta-1,3-N-acetylglucosaminyltransferase 7	B3GNT7	1.09	1.78*	1.50*	0.94	1.56	1.64*	1.41
Proprotein convertase subtilisin/kexin type 9	PCSK9	1.03	1.13	1.18	1.09	1.55	1.55	1.26
Protein O-linked-mannose beta-1,4-N-acetylglucosaminyltransferase 2	POMGNT2	1.04	1.53	1.41	1.30	1.55	1.88	1.04
Cytochrome P450 4F11	CYP4F11	0.97	1.71*	1.13	1.31	1.55	1.55	1.32
Arginase-2, mitochondrial	ARG2	1.67*	1.74*	1.25	1.44	1.55	1.51	0.93
Integral membrane protein 2B	ITM2B	1.06	1.19	1.07	1.35	1.53	1.61*	1.40
tRNA modification GTPase GTPBP3, mitochondrial	GTPBP3	0.71	1.16	1.14	1.12	1.53	1.55	0.65
GPI inositol-deacylase	PGAP1	1.24	1.51*	1.27	1.13	1.52	1.91*	1.21
p53 apoptosis effector related to PMP-22	PERP	1.05	1.22	1.25	0.82	1.51	2.65*	1.46

M. Common up-regulated proteins between LPS-Cytokines with Aquamin and with Mesalamine [50 proteins]

		Interventions						
		Control			*With LPS & Cytokines*			
Proteins	Genes	AQ	AQ+MES	MES	*LPS-Cyto*	***AQ***	*AQ+MES*	***MES***
Keratin, type II cytoskeletal 4	KRT4	3.27*	1.56*	9.03*	0.70	10.27*	1.13	6.70*
Cornifin-B	SPRR1B	0.79	1.62*	1.68*	1.14	4.30*	0.46*	3.71*
Cystatin-A	CSTA	1.20*	1.45*	1.11	1.25	3.86*	0.98	3.06*
Cornulin	CRNN	1.73*	2.38*	5.70*	0.44*	3.63*	0.61	2.57*
Protein KPLCE	KPLCE	1.16	0.88	0.98	0.61*	3.48*	0.40*	1.62*
Secretoglobin family 1D member 2	SCGB1D2	0.91	1.39	1.53*	1.04	3.37*	0.82	2.35*
Neutrophil elastase	ELANE	0.97	1.85*	0.83	1.30	3.28*	0.92	2.27*
Serpin B12	SERPINB12	0.96	1.21	2.32*	1.07	3.22*	0.93	2.21*
Protein-glutamine gamma-glutamyltransferase K	TGM1	1.13	1.39	1.71*	0.66	3.12*	0.49*	2.44*
Serpin B3	SERPINB3	0.72*	1.14	1.82*	1.19	2.93*	0.78	1.96*
Plakophilin-1	PKP1	0.84	3.19*	2.07*	0.84	2.73*	0.56*	2.07*
Cystatin-S	CST4	0.83	1.14	1.53*	1.06	2.72*	1.49	2.96*
Immunoglobulin heavy constant alpha 1	IGHA1	0.68*	1.29*	3.28*	1.11	2.69*	1.30	2.32*
Keratin, type I cytoskeletal 23	KRT23	2.31*	1.81*	6.31*	0.44*	2.65*	0.70	2.04*
Semenogelin-1	SEMG1	1.74*	4.10*	2.91*	0.71	2.56*	0.55*	2.67*
Histidine ammonia-lyase	HAL	1.60*	2.11*	5.94*	0.35*	2.50*	0.56	2.12*
BPI fold-containing family A member 1	BPIFA1	1.11	2.30*	1.94*	1.49*	2.48*	1.40	3.23*
Zymogen granule protein 16 homolog B	EECP	1.16	1.50*	1.36*	1.18	2.47*	1.02	2.66*
Galectin-7	LGALS7	0.90	4.40*	4.72*	1.18	2.20*	0.97	1.65
Repetin	RPTN	1.60*	1.00	4.84*	0.42*	2.18*	0.67	3.40*
Carboxypeptidase A4	CPA4	0.89	1.16	1.45*	0.83	2.09*	0.70	1.86*
Hornerin	HRNR	0.99	0.89	1.09	1.23	2.06*	1.28	1.68*
Serine/threonine-protein kinase 31	STK31	2.24*	1.32	4.57*	0.59*	2.06*	0.73	1.99*
Cystatin-M	CST6	1.81*	3.67*	3.47*	0.54*	1.96*	0.58	1.84*
Myeloblastin	PRTN3	0.84	3.59*	2.95*	0.55*	1.93*	0.45*	1.61
Inositol polyphosphate-4-phosphatase type I A	INPP4A	0.52*	0.62	0.73	0.89	1.91*	1.37	1.56
Immunoglobulin heavy constant alpha 2	IGHA2	1.13	3.35*	14.54*	0.74	1.90*	1.16	4.45*
Corneodesmosin	CDSN	1.08	0.72*	0.79	1.44*	1.86*	0.87	1.75*
RING1 and YY1-binding protein	RYBP	1.77*	1.81*	2.11*	1.28	1.80	0.92	2.13*
Mammaglobin-B	SCGB2A1	1.32	5.33*	2.76*	0.64	1.80*	0.64	1.75
Mitotic spindle assembly checkpoint protein MAD2A	MAD2L1	1.28	1.24	1.18	1.44	1.73*	1.40	1.91*
tRNA N6-adenosine threonylcarbamoyltransferase, mitochondrial	OSGEPL1	1.13	0.88	0.97	1.11	1.71	1.38	1.61
SRSF protein kinase 2	SRPK2	0.77	0.95	0.96	1.06	1.64*	1.38	1.84*

DDB1- and CUL4-associated factor 1	DCAF1	0.85	0.86	0.90	1.20	1.64*	1.44	1.69*
Kallikrein-10	KLK10	1.24*	0.99	1.30*	1.49*	1.63*	1.37	1.51*
Plasminogen	PLG	0.62	1.31	1.39	1.44	1.62	1.48	1.84
Cell division cycle protein 16 homolog	CDC16	1.12	1.46	1.40	1.32	1.61*	1.11	1.58
Lysophosphatidic acid receptor 2	LPAR2	1.31	0.98	1.18	0.93	1.60	1.36	1.54
Transcription initiation factor TFIID subunit 5	TAF5	0.45*	0.59*	0.49*	0.70	1.60	1.45	1.68*
YTH domain-containing family protein 1	YTHDF1	0.95	1.17	1.09	1.02	1.58*	1.43	1.57*
BTB/POZ domain-containing protein KCTD14	KCTD14	0.89	1.16	1.10	1.23	1.57	1.37	1.61
Pyruvate kinase PKLR	PKLR	0.74*	1.35	1.19	1.31	1.57	1.24	1.64*
Retroviral-like aspartic protease 1	ASPRV1	0.92	3.16*	10.29*	0.39*	1.54*	0.49*	1.97*
Serine protease 1	PRSS1	0.83	1.15	1.10	1.33	1.54*	1.50	1.98*
Synaptojanin-1	SYNJ1	0.74	0.96	0.94	1.01	1.53	1.49	1.61
Transport and Golgi organization protein 2 homolog	TANGO2	1.01	1.34	1.26	1.11	1.52	1.26	1.79
Amphiregulin	AREG	0.63	1.46	1.11	1.32	1.51	1.25	3.02*
Cyclin-dependent kinase 1	CDK1	0.62*	0.67*	0.68*	0.40*	1.51	1.34	1.54*
Putative hydroxypyruvate isomerase	HYI	1.07	1.19	1.13	1.22	1.50	1.34	1.51
Proto-oncogene c-Rel	REL	0.63*	0.88	0.79	1.17	1.50	0.99	1.70*

N. Common up-regulated proteins between LPS-Cytokines with Aquamin plus Mesalamine and with Mesalamine [105 proteins]

		Interventions						
		Control			*With LPS & Cytokines*			
Proteins	Genes	AQ	AQ+MES	MES	*LPS-Cyto*	*AQ*	***AQ+MES***	***MES***
C-X-C motif chemokine 10	CXCL10	0.72	34.05*	43.44*	0.97	0.83	30.10*	22.62*
HLA class II histocompatibility antigen, DM beta chain	HLA-DMB	1.59*	13.66*	8.99*	1.13	1.05	10.01*	8.97*
HLA class II histocompatibility antigen, DM alpha chain	HLA-DMA	1.43	5.47*	4.95*	1.02	1.13	5.22*	4.36*
HLA class II histocompatibility antigen, DR beta 4 chain	HLA-DRB4	0.71	3.84*	4.17*	0.84	1.08	4.68*	3.60*
Chromogranin-A	CHGA	0.64*	5.56*	5.04*	0.86	1.34	4.03*	6.57*
Fibroleukin	FGL2	0.77	3.23*	3.07*	0.79	0.93	3.82*	3.15*
Complement C2	C2	0.70	2.82*	4.91*	0.68	0.79	3.36*	3.92*
Secretogranin-2	SCG2	0.74	3.08*	4.35*	0.99	1.09	3.27*	5.45*
Plasmalemma vesicle-associated protein	PLVAP	0.75	3.13*	3.90*	1.02	1.13	3.00*	3.92*
Centrosomal protein of 85 kDa	CEP85	0.93	4.39*	4.17*	1.04	1.23	2.87*	4.16*
T-complex protein 10A homolog 1	TCP10L	0.85	1.26	2.12*	0.87	0.83	2.79*	2.40*
Synaptic vesicle membrane protein VAT-1 homolog-like	VAT1L	0.71	3.05*	4.20*	0.93	1.19	2.75*	4.58*
Tenascin-X	TNXB	0.89	2.86*	3.62*	0.93	1.02	2.74*	3.87*
Transmembrane protein 236	TMEM236	1.79*	4.00*	2.65*	1.34	1.43	2.59*	1.66
Phospholipid transfer protein	PLTP	0.70	2.51*	3.16*	0.97	0.87	2.48*	3.00*

Cadherin-13	CDH13	0.84	2.41*	3.15*	0.88	1.01	2.44*	3.83*
Vasopressin-neurophysin 2-copeptin	AVP	0.94	3.13*	3.08*	1.27	1.08	2.41*	3.99*
EH domain-containing protein 3	EHD3	0.76	3.83*	3.66*	1.19	1.06	2.38*	3.59*
Receptor-type tyrosine-protein phosphatase zeta	PTPRZ1	0.81	2.75*	4.03*	0.97	1.19	2.32*	3.43*
Interleukin-1 receptor accessory protein	IL1RAP	0.61*	2.35*	2.99*	0.79	0.92	2.31*	3.58*
Contactin-1	CNTN1	0.65*	2.25*	2.79*	0.80	0.88	2.29*	3.42*
A disintegrin and metalloproteinase with thrombospondin motifs 13	ADAMTS13	0.68	2.03*	2.82*	0.65	0.85	2.28*	2.94*
Hepatocyte growth factor activator	HGFAC	0.62*	2.13*	2.48*	0.77*	0.77	2.27*	3.01*
Oncoprotein-induced transcript 3 protein	OIT3	0.70*	2.44*	2.81*	0.81	0.94	2.23*	3.12*
Fibromodulin	FMOD	0.64*	2.45*	2.75*	1.06	0.95	2.23*	3.19*
Moesin	MSN	0.69*	1.72*	3.19*	1.45*	1.46*	2.21*	3.03*
Collagen alpha-1(XI) chain	COL11A1	0.53*	2.32*	2.75*	0.87	0.94	2.20*	3.10*
Beta-parvin	PARVB	0.82	2.48*	3.45*	0.96	1.17	2.19*	3.29*
Pigment epithelium-derived factor	SERPINF1	0.57*	1.24	1.90*	1.24	0.96	2.19*	3.38*
NEDD4 family-interacting protein 2	NDFIP2	1.07	2.34*	2.44*	0.95	1.12	2.19*	2.96*
Retinol-binding protein 4	RBP4	0.67*	2.60*	2.77*	0.92	0.90	2.14*	2.97*
Alpha-fetoprotein	AFP	0.54*	1.16	1.75*	1.41*	1.41*	2.14*	3.27*
Neural cell adhesion molecule 1	NCAM1	0.68*	2.18*	2.49*	0.81	0.77	2.12*	3.03*
Metalloproteinase inhibitor 3	TIMP3	0.80	3.49*	2.39*	0.78	0.88	2.09*	1.51
Tyrosine-protein kinase receptor Tie-1	TIE1	0.68	1.62*	2.43*	0.79	0.81	2.09*	2.76*
C-type mannose receptor 2	MRC2	0.88	2.19*	2.65*	0.96	1.06	2.07*	2.88*
Phosphatidylcholine-sterol acyltransferase	LCAT	0.65*	2.10*	2.67*	0.76	0.94	2.06*	2.94*
Alpha-1B-glycoprotein	A1BG	0.55*	1.99*	2.73*	0.77	0.76	2.05*	3.14*
Dynein axonemal heavy chain 1	DNAH1	0.51*	1.54*	2.15*	1.10	0.62*	2.04*	2.51*
Protein piccolo	PCLO	0.56*	1.75*	2.05*	0.55*	0.72	2.03*	2.36*
Afamin	AFM	0.52*	2.16*	2.43*	0.71*	0.67*	2.00*	2.67*
Retinal dehydrogenase 2	ALDH1A2	0.81	1.80*	1.94*	0.84	1.03	1.97*	2.31*
Sex hormone-binding globulin	SHBG	0.50*	2.02*	2.50*	0.70	0.75	1.95*	2.89*
Putative beta-actin-like protein 3	POTEKP	3.53*	4.54*	4.91*	1.20	1.16	1.95*	5.20*
Probable phosphoglycerate mutase 4	PGAM4	0.53*	1.15	2.54*	0.51*	0.63	1.94*	2.77*
Regucalcin	RGN	0.59*	1.19	1.30	1.08	1.30	1.94*	2.66*
Glutathione S-transferase A5	GSTA5	0.75	1.99*	2.22*	0.78	0.90	1.92*	2.62*
Plasma kallikrein	KLKB1	0.59*	2.06*	2.86*	0.72	0.82	1.89*	2.72*
Insulin	INS	0.69*	0.82	0.84	1.09	1.12	1.89*	2.68*
Apolipoprotein A-II	APOA2	0.90	2.24*	10.88*	0.84	1.17	1.89*	2.53*
EGF-containing fibulin-like extracellular matrix protein 1	EFEMP1	0.62*	2.26*	2.51*	0.81	0.53*	1.88*	2.37*
Collagen alpha-2(I) chain	COL1A2	0.53*	2.15*	2.47*	0.72	0.65	1.88*	2.71*

FERM and PDZ domain-containing protein 1	FRMPD1	0.54*	2.61*	2.58*	0.47*	0.94	1.85*	2.22*
Plastin-2	LCP1	0.68*	2.28*	7.32*	0.81	0.92	1.85*	2.39*
Interferon-induced protein 44-like	IFI44L	0.60*	1.47	1.36	1.22	1.34	1.82	3.21*
Scrapie-responsive protein 1	SCRG1	0.64*	2.21*	2.25*	0.80	0.99	1.82*	2.12*
Collagen alpha-1(XXI) chain	COL21A1	0.75	1.78*	2.36*	0.97	0.96	1.81*	2.34*
DENN domain-containing protein 3	DENND3	1.10	1.37	1.25	1.16	0.91	1.80	1.74
Steryl-sulfatase	STS	1.09	1.27	1.24	1.42*	1.36	1.79*	1.57
Cell adhesion molecule 1	CADM1	0.65*	1.89*	2.04*	0.63	0.89	1.76*	2.25*
ERC protein 2	ERC2	0.70	2.27*	2.27*	0.79	0.70	1.76	2.58*
Selenoprotein P	SELENOP	0.80	1.87*	2.83*	0.81	1.24	1.76*	1.95*
DnaJ homolog subfamily B member 9	DNAJB9	1.26	1.81*	1.41	1.32	1.26	1.76	1.72
Mucin-3A	MUC3A	1.00	2.47*	2.01*	1.05	1.21	1.75*	1.67*
Acyl-coenzyme A synthetase ACSM3, mitochondrial	ACSM3	1.15	1.53	1.29	1.43	1.41	1.74*	1.81*
Cerebellin-4	CBLN4	0.41*	1.58	2.52*	0.57*	0.78	1.73	1.95*
Ubiquitin carboxyl-terminal hydrolase isozyme L1	UCHL1	0.88	0.91	1.36	1.44	1.28	1.72	1.75
Exocyst complex component 3-like protein 4	EXOC3L4	0.82	1.70*	1.67*	0.79	0.71	1.71*	1.62
2'-5'-oligoadenylate synthase-like protein	OASL	1.25	1.70*	1.54*	1.09	1.26	1.71*	1.54
TRPM8 channel-associated factor 2	TCAF2	1.14	1.76*	2.34*	1.03	1.43	1.71	1.71
Carboxypeptidase E	CPE	0.79	2.20*	2.47*	0.75	0.99	1.70	2.53*
Tissue factor	F3	0.97	1.60*	1.48*	1.34*	1.23	1.69*	1.58*
Transforming growth factor-beta-induced protein ig-h3	TGFBI	0.93	1.95*	1.72*	1.09	1.36	1.69*	2.50*
Complement factor H	CFH	0.62*	2.03*	27.05*	0.74	0.62	1.68*	1.72*
Membrane primary amine oxidase	AOC3	0.85	2.15*	3.07*	0.93	1.18	1.67	2.86*
Cytochrome P450 2J2	CYP2J2	0.90	1.14	1.05	1.03	1.36	1.67	1.54
Collagen alpha-1(III) chain	COL3A1	0.82	1.61*	2.22*	0.72	0.87	1.66*	2.04*
Peptidyl-prolyl cis-trans isomerase E	PPIE	0.92	1.14	1.34*	1.12	1.27	1.66*	2.21*
Insulin-like growth factor II	IGF2	0.62*	2.07*	2.04*	0.74	0.48*	1.65*	2.22*
Protein GUCD1	GUCD1	1.02	1.64*	1.44	0.74	1.48	1.65	1.75
Probable serine carboxypeptidase CPVL	CPVL	0.97	1.32	1.59*	1.34	1.15	1.65*	2.03*
Desumoylating isopeptidase 1	DESI1	0.95	1.72*	1.76*	1.06	1.21	1.64	2.27*
Coagulation factor XIII A chain	F13A1	0.79	1.13	1.36	1.44	1.37	1.62	2.10*
Latent-transforming growth factor beta-binding protein 4	LTBP4	0.82	2.90*	2.39*	1.01	1.40	1.61	3.16*
Microtubule-associated proteins 1A/1B light chain 3 beta 2	MAP1LC3B2	1.26	1.67*	1.73*	1.34	1.32	1.61	1.83*
DNA ligase 1	LIG1	0.88	0.85	0.81	0.95	1.47	1.60	1.53
Tropomyosin beta chain	TPM2	0.68*	1.91*	1.88*	0.86	0.83	1.59	2.29*
Coilin	COIL	0.37*	0.67	0.64*	0.77	1.39	1.58	1.54
Forkhead box protein G1	FOXG1	0.98	1.32	1.34	1.04	1.17	1.58	1.75*

Cytosolic iron-sulfur assembly component 2B	CIAO2B	0.45*	0.60*	1.29	0.47*	0.38*	1.57	1.87
Desmoglein-3	DSG3	0.68	3.24*	4.23*	0.59	0.94	1.57	2.39*
ER degradation-enhancing alpha-mannosidase-like protein 2	EDEM2	1.23	1.67*	1.68*	1.01	1.30	1.56	1.89*
Tubulointerstitial nephritis antigen-like	TINAGL1	0.67*	0.93	1.35*	1.10	1.17	1.55*	2.61*
Sorting nexin-18	SNX18	1.13	1.23	1.20	1.12	1.42	1.55	1.51
Complement factor D	CFD	0.59*	1.43	3.72*	0.81	0.58	1.54	2.05*
Guanidinoacetate N-methyltransferase	GAMT	0.55*	1.98*	1.89*	0.67	0.57	1.54	1.94*
Laminin subunit alpha-2	LAMA2	1.06	1.20	1.71*	1.33	1.36	1.54	1.76
E3 ubiquitin-protein ligase DTX3L	DTX3L	0.93	0.99	0.95	1.42*	1.45	1.53*	1.62*
Glycosylphosphatidylinositol anchor attachment 1 protein	GPAA1	1.14	1.22	1.08	1.18	1.33	1.52*	1.88*
Septin-6	SEPTIN6	0.72	0.95	0.96	1.16	1.22	1.52	1.82*
Desmoglein-4	DSG4	1.01	65.35*	2.23*	0.77	1.18	1.51	2.16*
Angiopoietin-related protein 4	ANGPTL4	1.12	0.96	1.32	1.31	1.32	1.51	1.62
Lysyl oxidase homolog 3	LOXL3	0.95	1.24	1.68*	0.97	1.28	1.51	1.85*
Agrin	AGRN	0.86	1.17	1.13	1.28*	1.30	1.51*	2.14*
Protein PML	PML	0.89	0.87	0.96	1.21	0.95	1.51	1.75

O. Common up-regulated proteins among LPS-Cytokines with Aquamin, with Aquamin plus Mesalamine and with Measlamine [137 proteins]

		Interventions						
		Control			*With LPS & Cytokines*			
Proteins	Genes	AQ	AQ+MES	MES	*LPS-Cyto*	***AQ***	***AQ+MES***	***MES***
Ligand of Numb protein X 2	LNX2	1.06	1.01	0.96	1.48	6.81*	4.23*	4.01*
Myosin-2	MYH2	0.56*	0.80	0.45*	1.12	5.57*	4.93*	4.82*
Endoplasmic reticulum protein SC65	P3H4	0.73	0.82	0.75	1.40	5.17*	4.16*	5.01*
Ephrin type-B receptor 3	EPHB3	0.51*	0.62*	0.60*	1.22	4.74*	3.26*	4.09*
Heparan sulfate glucosamine 3-O-sulfotransferase 1	HS3ST1	0.19*	0.35*	0.29*	1.17	4.51*	3.55*	5.50*
Low-density lipoprotein receptor-related protein 2	LRP2	0.60*	1.46	1.02	1.45	4.10*	5.03*	6.63*
Mammaglobin-A	SCGB2A2	1.06	1.93*	1.71*	1.32	4.09*	1.54	3.31*
Pleckstrin	PLEK	0.56*	1.21	0.63*	1.27	4.09*	3.74*	4.36*
Endothelial lipase	LIPG	0.35*	0.46*	0.43*	1.12	4.07*	3.10*	3.50*
Alpha-1-antichymotrypsin	SERPINA3	1.08	1.90*	2.88*	1.28	3.87*	1.63	2.12*
Beta-1,3-N-acetylglucosaminyltransferase lunatic fringe	LFNG	0.67*	0.50*	0.41*	0.90	3.55*	2.99*	3.10*
Bone morphogenetic protein 1	BMP1	0.22*	0.37*	0.31*	0.91	3.52*	2.50*	3.63*
DNA topoisomerase 2-alpha	TOP2A	0.73*	0.82	0.69*	0.95	3.45*	3.10*	3.18*
Coagulation factor X	F10	0.41*	0.77	0.81	1.13	3.39*	5.07*	5.60*
Reticulocalbin-3	RCN3	0.17*	0.72	0.78	1.01	3.31*	4.57*	5.68*

Immunoglobulin lambda constant 2	IGLC2	0.66*	1.33	4.95*	1.42	3.25*	2.14*	1.55
Protein Wnt-3a	WNT3A	0.49*	0.92	0.90	1.17	3.24*	4.10*	5.41*
Mimecan	OGN	0.29*	0.85	0.94	1.34	3.21*	4.58*	5.97*
Serum paraoxonase/arylesterase 1	PON1	0.69	1.33	1.69*	1.47	3.15*	4.26*	4.58*
Ceruloplasmin	CP	0.63*	1.04	1.10	1.39*	3.14*	3.98*	4.43*
Glutathione S-transferase Mu 2	GSTM2	0.74	1.19	1.67*	1.22	3.12*	3.94*	3.58*
Carboxypeptidase N catalytic chain	CPN1	0.56*	0.92	0.92	1.44*	3.11*	4.54*	5.08*
Cilia- and flagella-associated protein 45	CFAP45	1.69*	1.98*	2.43*	1.19	3.11*	2.25*	2.51*
Apolipoprotein C-I	APOC1	0.88	1.73*	2.48*	1.47*	3.07*	4.84*	3.83*
Matrix metalloproteinase-28	MMP28	0.33*	0.49*	0.56*	0.80	3.03*	2.32*	2.79*
Mast/stem cell growth factor receptor Kit	KIT	0.70	1.13	0.66*	0.79	3.01*	2.43*	2.74*
DNA (cytosine-5)-methyltransferase 1	DNMT1	0.48*	0.32*	0.41*	0.70	3.00*	2.01*	2.38*
Fermitin family homolog 3	FERMT3	0.48*	1.02	0.99	1.11	2.94*	4.31*	4.74*
Immunoglobulin kappa constant	IGKC	0.71*	1.56*	4.90*	1.34*	2.92*	1.88*	2.00*
FK506-binding protein-like	FKBPL	0.89	0.94	0.99	1.11	2.90*	2.69*	1.57
Tumor necrosis factor ligand superfamily member 9	TNFSF9	0.73	0.93	0.78	1.36	2.86*	2.06*	2.33*
Serine incorporator 5	SERINC5	0.80	0.94	0.92	1.49*	2.82*	2.63*	2.45*
Dihydropyrimidinase	DPYS	0.33*	0.71	0.75	1.13	2.82*	3.76*	4.94*
Microtubule-associated protein 1B	MAP1B	0.42*	0.77	0.85	1.20	2.79*	2.86*	3.79*
Protocadherin gamma-C3	PCDHGC3	0.54*	1.39	1.37*	1.48*	2.77*	3.66*	5.21*
Complement component C7	C7	0.72	1.09	1.33	1.47	2.77*	4.26*	4.88*
C-type lectin domain family 11 member A	CLEC11A	0.51*	1.23	1.22	1.20	2.68*	3.66*	4.12*
Transmembrane protein 223	TMEM223	1.04	0.97	0.93	1.24	2.66*	1.67	1.61
RAF proto-oncogene serine/threonine-protein kinase	RAF1	0.83	0.87	0.84	1.08	2.65*	2.68*	2.02*
Collectin-10	COLEC10	0.62*	1.16	1.14	1.23	2.63*	3.78*	4.85*
Dynamin-1	DNM1	0.57*	1.02	0.79	1.22	2.61*	1.81*	2.14*
Transmembrane gamma-carboxyglutamic acid protein 1	PRRG1	0.88	1.02	1.15	1.38	2.61*	2.51*	1.80*
Collectin-11	COLEC11	0.28*	0.79	1.14	1.30	2.59*	3.59*	5.27*
Solute carrier family 35 member B1	SLC35B1	2.33*	2.96*	3.17*	1.47	2.58*	2.91*	2.01*
Condensin complex subunit 2	NCAPH	1.51*	1.22	1.07	1.36	2.49*	2.98*	1.72*
Vasorin	VASN	0.39*	0.96	0.86	1.44	2.45*	3.55*	5.06*
Periostin	POSTN	0.66*	1.63*	1.51*	1.02	2.43*	2.54*	3.38*
CCR4-NOT transcription complex subunit 6	CNOT6	0.63*	0.77	0.73	1.15	2.38*	1.85*	2.10*
Anaphase-promoting complex subunit 1	ANAPC1	0.33*	0.29*	0.32*	0.60*	2.35*	1.97*	2.10*
Protein spinster homolog 1	SPNS1	1.08	1.18	1.22	1.45	2.35*	2.03*	2.00*
Cartilage-associated protein	CRTAP	0.97	1.09	1.04	1.21	2.34*	2.51*	2.31*
MANSC domain-containing protein 1	MANSC1	0.94	1.34	1.50*	1.10	2.34*	2.35*	1.72*

Procollagen C-endopeptidase enhancer 1	PCOLCE	0.69	1.78*	2.20*	0.96	2.34*	2.41*	2.65*
Alpha-N-acetylgalactosaminide alpha-2,6-sialyltransferase 6	ST6GALNAC6	1.10	1.02	1.14	1.14	2.33*	2.20*	1.93*
Plasmolipin	PLLP	1.13	1.06	1.05	1.50*	2.31*	1.82*	1.64*
von Willebrand factor A domain-containing protein 1	VWA1	0.61*	1.07	1.14	1.04	2.29*	2.51*	3.15*
N-acetyllactosaminide beta-1,3-N-acetylglucosaminyltransferase 2	B3GNT2	1.29	1.91*	1.58*	1.36	2.26*	2.13*	2.17*
Proton myo-inositol cotransporter	SLC2A13	1.34	1.49	1.29	1.28	2.22*	2.37*	1.80*
Complement component C9	C9	0.83	1.47*	1.50*	1.29	2.22*	3.31*	3.45*
Sushi repeat-containing protein SRPX2	SRPX2	0.71*	1.06	1.21	0.82	2.21*	1.83*	2.35*
Noggin	NOG	0.90	1.20	1.12	1.37	2.18*	3.03*	2.78*
Protein SYS1 homolog	SYS1	1.18	1.11	1.13	1.38	2.13*	2.08*	1.69
Anaphase-promoting complex subunit CDC26	CDC26	0.99	1.15	1.18	1.36	2.10*	2.14*	1.97
Serpin B7	SERPINB7	1.00	1.84*	1.21	1.19	2.08*	2.49*	1.71*
BolA-like protein 1	BOLA1	1.13	0.84	1.21	1.37	2.08*	1.51	1.58
Deoxynucleoside triphosphate triphosphohydrolase SAMHD1	SAMHD1	0.65	1.18	1.07	1.16	2.07*	1.70	1.86
Glucose-6-phosphate exchanger SLC37A4	SLC37A4	1.22	1.37	1.24	1.36	2.07*	2.39*	1.67
Bromodomain-containing protein 9	BRD9	0.70*	1.71*	1.15	1.38*	2.07*	3.25*	5.06*
Ectonucleoside triphosphate diphosphohydrolase 6	ENTPD6	1.31	1.39	1.18	1.44	2.06*	2.00*	1.81*
Proteasomal ATPase-associated factor 1	PAAF1	0.79	1.33	1.03	1.34	2.05*	1.58	2.35*
SPARC-related modular calcium-binding protein 2	SMOC2	0.84	1.62*	1.69*	1.38	2.05*	1.83*	1.95*
Cytochrome c oxidase assembly protein COX11, mitochondrial	COX11	1.28	1.96*	2.23*	1.39	2.04*	2.52*	3.10*
Trafficking protein particle complex subunit 2	TRAPPC2	1.13	1.07	1.03	1.43*	2.03*	1.85*	1.83*
Nidogen-2	NID2	0.73	1.89*	1.61*	1.45*	2.02*	2.55*	3.43*
4-galactosyl-N-acetylglucosaminide 3-alpha-L-fucosyltransferase FUT6	FUT6	1.30*	1.36	1.24	1.39	2.02*	1.83*	1.84*
Galactose-3-O-sulfotransferase 2	GAL3ST2	0.84	0.77	0.80	0.85	2.02*	1.95*	1.90*
Sterile alpha motif domain-containing protein 9-like	SAMD9L	1.07	1.96*	1.88*	1.41	2.01*	3.01*	3.00*
Centrin-3	CETN3	1.05	1.10	0.89	1.21	2.00*	2.02*	1.57
Bcl-2-like protein 1	BCL2L1	1.01	1.01	0.95	1.48*	2.00*	1.88*	1.78*
Collagen alpha-1(I) chain	COL1A1	0.89	1.53*	1.40*	1.45*	1.99*	2.46*	3.30*
RRP15-like protein	RRP15	1.11	0.98	0.88	1.49*	1.99*	1.63*	2.82*
Sodium/hydrogen exchanger 6	SLC9A6	1.17	1.09	1.20	1.35	1.96*	2.05*	1.54
Fumarylacetoacetase	FAH	0.97	0.83	0.99	1.31	1.96*	1.77*	1.88*
Growth factor receptor-bound protein 7	GRB7	0.74	0.68	0.71	1.04	1.95*	1.91*	1.61
Acyl-coenzyme A thioesterase MBLAC2	MBLAC2	1.00	1.09	1.30	1.48*	1.92*	1.83*	1.85*
Sulfotransferase 2A1	SULT2A1	1.49*	5.11*	2.72*	1.25	1.90*	2.73*	1.62

UNC93-like protein MFSD11	MFSD11	1.13	1.15	1.06	1.30	1.88*	1.93*	1.52
Nidogen-1	NID1	0.75*	1.56*	1.44*	1.41*	1.86*	2.21*	2.89*
CD177 antigen	CD177	1.04	1.45*	1.41*	1.26	1.85*	2.23*	1.55*
Laminin subunit beta-2	LAMB2	0.67*	1.91*	1.30*	1.38*	1.83*	2.20*	3.05*
HIG1 domain family member 1A, mitochondrial	HIGD1A	1.46	1.58*	1.10	1.43	1.83*	2.31*	1.94*
cAMP-specific 3',5'-cyclic phosphodiesterase 4C	PDE4C	0.89	2.07*	2.99*	0.79	1.82*	2.80*	3.99*
Solute carrier organic anion transporter family member 2B1	SLCO2B1	1.05	1.32	1.49*	1.04	1.81	1.86	1.72
Stromal cell-derived factor 2	SDF2	0.95	1.08	1.16	1.25	1.80*	1.78*	1.55
Alpha-1,3-mannosyl-glycoprotein 2-beta-N-acetylglucosaminyltransferase	MGAT1	1.12	1.44*	1.50*	1.39*	1.79*	1.89*	1.90*
Fibulin-1	FBLN1	0.88	1.47*	1.43*	1.41*	1.79*	1.99*	1.99*
Cytochrome c oxidase assembly protein COX20, mitochondrial	COX20	1.21*	1.14	1.03	1.46*	1.79*	1.59*	1.64*
UPF0606 protein KIAA1549L	KIAA1549L	1.36	0.96	1.23	1.26	1.77*	1.54	1.62
Retinol-binding protein 1	RBP1	1.18	1.15	1.11	1.21	1.75*	2.10*	1.53
Carboxypeptidase A2	CPA2	1.24	3.77*	2.77*	0.76	1.74	4.32*	2.79*
F-box only protein 2	FBXO2	1.08	2.13*	1.67*	0.90	1.73*	2.19*	1.63
Molybdate-anion transporter	MFSD5	1.26	1.19	1.13	1.22	1.73*	1.70*	1.52
Vesicle transport protein GOT1B	GOLT1B	1.02	1.29	1.37*	1.27	1.73*	2.35*	1.58
Chloride intracellular channel protein 5	CLIC5	1.22*	1.32	1.26*	1.41*	1.73*	1.54*	1.52*
Out at first protein homolog	OAF	0.98	1.36	0.99	1.38	1.71*	1.51	1.61
Testis-expressed protein 9	TEX9	1.68*	1.53*	0.95	1.42*	1.70*	2.57*	2.77*
Acetylcholinesterase	ACHE	1.68*	3.16*	2.26*	1.09	1.69	2.30*	1.62
Lysyl oxidase homolog 4	LOXL4	0.90	1.02	1.10	1.34	1.69	1.66	1.90*
Nuclear autoantigen Sp-100	SP100	1.01	1.18	0.89	1.33	1.67	2.13*	2.28*
Uncharacterized protein MISP3	MISP3	1.18	1.36	1.56*	1.30	1.66	1.70	2.06*
Collagen alpha-1(V) chain	COL5A1	1.02	1.90*	1.77*	1.10	1.65*	2.12*	2.62*
Mitochondrial thiamine pyrophosphate carrier	SLC25A19	1.15	1.00	0.97	1.28	1.65	1.54	1.51
Bisphosphoglycerate mutase	BPGM	0.83	1.30	1.11	1.07	1.64	1.94*	2.03*
WD repeat-containing protein 70	WDR70	0.91	0.86	0.97	1.03	1.63*	1.55*	1.56*
Proteinase-activated receptor 2	F2RL1	1.12	1.22	1.20	1.40*	1.63*	1.78*	1.69*
Clusterin	CLU	0.83	0.92	1.32	1.31	1.61	1.50	2.08*
Heat shock 70 kDa protein 13	HSPA13	0.96	1.49*	1.51*	1.27	1.61*	1.99*	2.46*
Mitochondrial fission process protein 1	MTFP1	1.06	1.51*	1.46*	1.44	1.61*	1.62*	1.80*
Follistatin-related protein 1	FSTL1	0.95	4.04*	4.57*	1.47*	1.61	3.52*	5.73*
Protein S100-A3	S100A3	1.12	26.05*	2.82*	0.58*	1.60	1.77	3.12*
Collagen alpha-2(VI) chain	COL6A2	0.64*	1.24	1.33	1.16	1.60	2.85*	3.58*

eEF1A lysine and N-terminal methyltransferase	METTL13	0.70	0.78	0.82	0.87	1.59	1.63	1.60
Mitochondrial glutamate carrier 2	SLC25A18	0.86	1.95*	2.62*	0.94	1.58	2.10*	3.70*
Collagen alpha-2(IV) chain	COL4A2	0.86	1.37	1.05	1.41*	1.58*	2.07*	3.23*
Ras-related protein Rab-8B	RAB8B	0.94	1.25	1.10	1.33	1.58	1.67*	1.51
CD70 antigen	CD70	0.94	1.26	1.25	1.30	1.58	1.63	1.60
Type-1 angiotensin II receptor-associated protein	AGTRAP	1.15	1.18	1.22	1.25	1.58	1.64	1.65
Mediator of RNA polymerase II transcription subunit 1	MED1	0.91	1.28	1.27	1.27	1.57	1.76*	1.95*
Solute carrier family 2, facilitated glucose transporter member 5	SLC2A5	1.25*	6.90*	5.49*	1.35*	1.55*	6.09*	4.52*
Lumican	LUM	0.67*	1.73*	1.81*	1.41*	1.54*	2.50*	3.78*
WD repeat-containing protein 74	WDR74	0.74	0.58*	0.71	0.94	1.54	1.63	1.63
Ubiquitin-like modifier-activating enzyme 7	UBA7	1.21	1.29	1.14	1.39*	1.53*	1.66*	1.57*
Midasin	MDN1	0.66*	1.29	1.56*	1.48*	1.53*	2.03*	2.59*
Dynein axonemal assembly factor 10	DNAAF10	1.08	1.30	1.22	1.36	1.52	1.55	1.65
Collagen alpha-1(VI) chain	COL6A1	0.88	1.56*	1.49*	1.06	1.51*	2.05*	2.64*
Prolyl 3-hydroxylase 1	P3H1	1.03	1.04	1.15	1.16	1.51	1.55	1.79*
Basement membrane-specific heparan sulfate proteoglycan core protein	HSPG2	0.86*	1.45*	1.21*	1.18	1.51*	1.85*	2.07*

Values represent the abundance ratio from organoids (n=4 subjects) compared to the control. These proteins were up-regulated at a 1.5-fold change (<2% FDR). Corresponding abundance ratios from the other treatment groups are provided for comparison. Proteins common among groups and unique to individual groups under proinflammatory conditions (LPS-Cytokines) are presented. *Indicates significance compared to the control (at $p<0.05$).
