# Supplementary material for: Proteomic profile of human colon organoids: effects of a multi-mineral intervention alone and in the presence of pro-inflammatory and anti-inflammatory treatments
Source: Front Gastroenterol (Lausanne). 2025 Jul 2;4:1592669. doi: 10.3389/fgstr.2025.1592669 (PMC12952359; doi:10.3389/fgstr.2025.1592669)
Supplement: Supplementary Table S1 — Mineral composition of Aquamin. [file DataSheet1.zip › Table S6.pdf]

Supplement Table 6. Down-regulated proteins influenced by LPS-Cytokines alone and with Aquamin and Mesalamine (with 1.5-fold)

A. Common among four groups: LPS-Cytokines, +Aquamin, +Mesalamine and +Aquamin plus Mesalamine (261 proteins)

		Interventions						
		Control			*With LPS & Cytokines*			
Proteins	Genes	AQ	AQ+MES	MES	***LPS-Cyto***	***AQ***	***AQ+MES***	***MES***
WD repeat and FYVE domain-containing protein 3	WDFY3	0.23*	0.09*	0.12*	0.09*	0.07*	0.18*	0.06*
TBC1 domain family member 25	TBC1D25	0.21*	0.09*	0.08*	0.10*	0.11*	0.15*	0.07*
Rho guanine nucleotide exchange factor 3	ARHGEF3	0.18*	0.44*	0.36*	0.12*	0.48*	0.17*	0.24*
Integrin alpha-E	ITGAE	0.27*	0.09*	0.14*	0.13*	0.10*	0.22*	0.09*
PCNA-interacting partner	PARPBP	0.40*	0.11*	0.08*	0.13*	0.12*	0.16*	0.06*
Guanine nucleotide-binding protein-like 3-like protein	GNL3L	0.23*	0.38*	0.37*	0.14*	0.22*	0.31*	0.20*
Polyamine-modulated factor 1-binding protein 1	PMFBP1	0.39*	0.15*	0.13*	0.14*	0.15*	0.25*	0.08*
Zinc finger protein 654	ZNF654	0.19*	0.24*	0.21*	0.15*	0.36*	0.33*	0.29*
Zinc finger protein with KRAB and SCAN domains 4	ZKSCAN4	0.27*	0.19*	0.22*	0.15*	0.37*	0.19*	0.22*
Protein kinase C theta type	PRKCQ	0.26*	0.08*	0.11*	0.16*	0.14*	0.22*	0.11*
Kelch-like ECH-associated protein 1	KEAP1	0.19*	0.33*	0.37*	0.18*	0.39*	0.32*	0.29*
E3 SUMO-protein ligase ZNF451	ZNF451	0.32*	0.37*	0.35*	0.19*	0.43*	0.44*	0.36*
NEDD4-binding protein 3	N4BP3	0.35*	0.47*	0.43*	0.20*	0.36*	0.29*	0.26*
Zinc finger protein 136	ZNF136	0.20*	0.18*	0.20*	0.20*	0.26*	0.23*	0.17*
Protein S100-A7A	S100A7A	0.27*	0.40*	4.36*	0.20*	0.48*	0.17*	0.46*
Oxytocin-neurophysin 1	OXT	0.28*	0.52*	0.47*	0.20*	0.20*	0.50*	0.37*
Centrosomal protein of 131 kDa	CEP131	0.35*	0.36*	0.35*	0.21*	0.33*	0.09*	0.09*
Protein ECT2	ECT2	0.28*	0.38*	0.40*	0.25*	0.64	0.62	0.55*
Protein Shroom1	SHROOM1	0.34*	0.73	0.64*	0.26*	0.49*	0.40*	0.24*
Sorting nexin-11	SNX11	0.89	1.08	1.16	0.26*	0.06*	0.16*	0.28*
DEP domain-containing mTOR-interacting protein	DEPTOR	0.53*	0.74	0.75*	0.26*	0.46*	0.39*	0.27*
Transcription factor Sp1	SP1	0.39*	0.49*	0.53*	0.26*	0.40*	0.44*	0.46*
Telomere-associated protein RIF1	RIF1	0.11*	0.18*	0.18*	0.26*	0.35*	0.29*	0.28*
Mitochondrial ribosome-associated GTPase 2	MTG2	0.63*	0.83	0.94	0.26*	0.29*	0.28*	0.25*
Calcium/calmodulin-dependent protein kinase type II subunit beta	CAMK2B	0.12*	0.10*	0.17*	0.26*	0.64*	0.53*	0.47*
Probable protein phosphatase 1N	PPM1N	0.40*	0.19*	0.23*	0.27*	0.19*	0.42*	0.17*
Folylpolyglutamate synthase, mitochondrial	FPGS	0.67*	0.98	0.96	0.28*	0.32*	0.24*	0.06*
C-X-C motif chemokine 14	CXCL14	0.36*	0.37*	0.42*	0.28*	0.28*	0.42*	0.39*
SURP and G-patch domain-containing protein 2	SUGP2	0.29*	0.33*	0.39*	0.28*	0.24*	0.23*	0.19*
Helicase-like transcription factor	HLTF	0.34*	0.41*	0.37*	0.28*	0.52	0.63	0.51

Ribosomal biogenesis protein LAS1L	LAS1L	0.22*	0.26*	0.25*	0.28*	0.62*	0.51*	0.40*
Dihydrofolate reductase	DHFR	1.10	1.27	1.41	0.29*	0.02*	0.21*	0.06*
Molybdenum cofactor sulfurase	MOCOS	0.36*	0.52*	0.58*	0.29*	0.24*	0.26*	0.25*
Ras association domain-containing protein 6	RASSF6	0.72*	1.04	0.98	0.29*	0.22*	0.19*	0.08*
AT-rich interactive domain-containing protein 2	ARID2	0.24*	0.38*	0.30*	0.30*	0.48*	0.35*	0.48*
DNA topoisomerase 2-binding protein 1	TOPBP1	0.34*	0.50*	0.60*	0.31*	0.44*	0.44*	0.51*
Inactive ubiquitin carboxyl-terminal hydrolase 53	USP53	0.46*	0.63*	0.51*	0.31*	0.32*	0.36*	0.22*
Rab-like protein 2A	RABL2A	0.67*	0.64*	0.77	0.32*	0.45*	0.43*	0.28*
Serine/threonine-protein kinase 11-interacting protein	STK11IP	0.63*	0.66	0.79	0.32*	0.11*	0.24*	0.36*
Zinc finger protein 592	ZNF592	0.25*	0.28*	0.27*	0.33*	0.59	0.55	0.49*
Keratin, type II cytoskeletal 2 epidermal	KRT2	0.21*	0.26*	0.47*	0.33*	0.50*	0.23*	0.35*
Rho GTPase-activating protein 29	ARHGAP29	0.19*	0.35*	0.41*	0.33*	0.58	0.50*	0.35*
Circadian locomoter output cycles protein kaput	CLOCK	0.43*	0.48*	0.56*	0.34*	0.60	0.53*	0.46*
Structural maintenance of chromosomes protein 5	SMC5	0.30*	0.36*	0.38*	0.34*	0.60	0.46*	0.61*
Replication factor C subunit 2	RFC2	0.59*	0.62*	0.63*	0.34*	0.52*	0.54*	0.44*
Gametogenetin-binding protein 2	GGNBP2	0.34*	0.37*	0.50*	0.34*	0.51*	0.46*	0.40*
Deoxycytidine kinase	DCK	0.40*	0.45*	0.56*	0.34*	0.63	0.57*	0.54*
ATPase WRNIP1	WRNIP1	0.62*	0.60*	0.63*	0.35*	0.39*	0.35*	0.38*
RING finger and CHY zinc finger domain-containing protein 1	RCHY1	0.48*	0.63*	0.65*	0.35*	0.05*	0.12*	0.05*
AN1-type zinc finger protein 6	ZFAND6	0.53*	0.75*	0.85	0.35*	0.06*	0.11*	0.22*
Trafficking kinesin-binding protein 1	TRAK1	0.29*	0.38*	0.48*	0.36*	0.46*	0.61	0.58
Acyl-CoA (8-3)-desaturase	FADS1	0.67*	0.65*	0.70*	0.36*	0.52*	0.44*	0.41*
NADPH oxidase organizer 1	NOXO1	0.60*	0.72	0.81	0.36*	0.61	0.52	0.31*
Zinc finger FYVE domain-containing protein 26	ZFYVE26	0.41*	0.71	0.58*	0.36*	0.55	0.65	0.44*
Actin filament-associated protein 1-like 2	AFAP1L2	0.38*	0.66	0.77	0.37*	0.13*	0.36*	0.30*
Vam6/Vps39-like protein	VPS39	0.50*	0.46*	0.46*	0.37*	0.63	0.54*	0.58*
Dephospho-CoA kinase domain-containing protein	DCAKD	0.87	0.98	0.96	0.38*	0.35*	0.42*	0.20*
Serine/threonine-protein kinase Chk2	CHEK2	0.56*	0.63*	0.61*	0.38*	0.31*	0.25*	0.25*
Nuclear receptor corepressor 1	NCOR1	0.36*	0.52*	0.51*	0.38*	0.32*	0.33*	0.51*
Tubulin alpha chain-like 3	TUBAL3	0.66*	0.67*	0.56*	0.39*	0.33*	0.45*	0.17*
Ankyrin repeat domain-containing protein SOWAHB	SOWAHB	0.35*	0.73	0.67	0.39*	0.53	0.48*	0.46*
Ly6/PLAUR domain-containing protein 8	LYPD8	1.39	0.75	0.60*	0.39*	0.50*	0.41*	0.34*
Peroxisomal ATPase PEX1	PEX1	0.56*	0.64*	0.64*	0.39*	0.43*	0.48*	0.31*
OTU domain-containing protein 3	OTUD3	0.60*	0.71	0.81	0.39*	0.22*	0.16*	0.43*
Coiled-coil domain-containing protein 85C	CCDC85C	0.61*	0.72*	0.70*	0.39*	0.15*	0.22*	0.24*
NF-X1-type zinc finger protein NFXL1	NFXL1	0.50*	0.58*	0.58*	0.40*	0.39*	0.35*	0.40*
Survival motor neuron protein	SMN1	0.18*	0.25*	0.29*	0.40*	0.38*	0.38*	0.43*

Lysine-specific demethylase 4B	KDM4B	0.84	0.89	0.79	0.40*	0.30*	0.24*	0.20*
Splicing regulator ARVCF	ARVCF	0.58*	0.91	0.98	0.40*	0.46*	0.37*	0.29*
Dual specificity mitogen-activated protein kinase kinase 5	MAP2K5	0.80	1.13	0.88	0.40*	0.51*	0.46*	0.42*
E3 ubiquitin-protein ligase Midline-1	MID1	0.32*	0.37*	0.40*	0.41*	0.38*	0.40*	0.35*
Beclin-1	BECN1	0.36*	0.54*	0.52*	0.41*	0.67	0.66	0.61*
Trefoil factor 2	TFF2	1.26	0.61*	0.60*	0.42*	0.59	0.34*	0.33*
Eukaryotic translation initiation factor 4E transporter	EIF4ENIF1	0.69*	0.95	0.98	0.42*	0.18*	0.18*	0.11*
E3 ubiquitin-protein ligase makorin-2	MKRN2	0.59*	0.67	0.65*	0.42*	0.60	0.52*	0.45*
High affinity cAMP-specific and IBMX-insensitive 3',5'-cyclic phosphodiesterase 8A	PDE8A	0.67	1.00	1.06	0.42*	0.23*	0.44*	0.20*
Rho GTPase-activating protein 32	ARHGAP32	0.53*	0.71	0.65*	0.42*	0.52*	0.63	0.46*
Epithelial splicing regulatory protein 2	ESRP2	0.58*	0.58*	0.66*	0.42*	0.50*	0.38*	0.45*
Probable ATP-dependent RNA helicase DDX56	DDX56	0.48*	0.51*	0.57*	0.42*	0.34*	0.34*	0.30*
S1 RNA-binding domain-containing protein 1	SRBD1	0.60*	0.53*	0.64*	0.43*	0.58	0.40*	0.23*
Ubiquitin-associated and SH3 domain-containing protein B	UBASH3B	0.46*	0.57*	0.63*	0.43*	0.52*	0.51*	0.44*
Protein SSXT	SS18	0.94	0.98	0.94	0.43*	0.17*	0.37*	0.57*
Bromodomain adjacent to zinc finger domain protein 1A	BAZ1A	0.43*	0.62*	0.56*	0.43*	0.58	0.43*	0.43*
RalBP1-associated Eps domain-containing protein 2	REPS2	0.62*	0.72	0.76	0.43*	0.43*	0.24*	0.25*
BAH and coiled-coil domain-containing protein 1	BAHCC1	0.54*	1.51*	0.94	0.43*	0.49*	0.40*	0.22*
GTPase IMAP family member 2	GIMAP2	0.71	0.78	0.85	0.43*	0.47*	0.58	0.26*
Leydig cell tumor 10 kDa protein homolog	C19orf53	0.21*	0.19*	0.20*	0.43*	0.37*	0.36*	0.45*
Protein Churchill	CHURC1	0.97	1.48*	1.21	0.43*	0.23*	0.14*	0.19*
Death domain-associated protein 6	DAXX	0.67*	0.81	0.80	0.44*	0.47*	0.33*	0.30*
DNA-directed RNA polymerases I and III subunit RPAC2	POLR1D	0.68*	0.68	0.74	0.44*	0.44*	0.49*	0.46*
DmX-like protein 2	DMXL2	0.50*	0.46*	0.41*	0.44*	0.53*	0.52*	0.44*
Zinc finger CCCH domain-containing protein 8	ZC3H8	0.48*	0.52*	0.58*	0.44*	0.51*	0.43*	0.45*
Uridine-cytidine kinase 2	UCK2	0.65*	0.69	0.67*	0.44*	0.39*	0.36*	0.34*
Putative GTP-binding protein 6	GTPBP6	0.69	0.83	0.91	0.44*	0.38*	0.36*	0.12*
HAUS augmin-like complex subunit 3	HAUS3	0.35*	0.39*	0.40*	0.45*	0.66	0.62	0.59
Pre-mRNA-splicing factor SLU7	SLU7	0.69*	0.95	0.95	0.45*	0.61	0.60	0.51*
Uncharacterized protein C2orf42	C2orf42	0.42*	0.67*	0.53*	0.45*	0.41*	0.45*	0.28*
Inositol hexakisphosphate kinase 1	IP6K1	0.78	0.89	0.90	0.45*	0.22*	0.26*	0.26*
Glutathione S-transferase A1	GSTA1	0.72*	0.38*	0.47*	0.45*	0.49*	0.64	0.41*
Zinc finger protein 701	ZNF701	0.43*	0.34*	0.36*	0.45*	0.46*	0.42*	0.48*
Eukaryotic translation initiation factor 2-alpha kinase 3	EIF2AK3	0.79	0.80	0.95	0.46*	0.42*	0.54	0.19*

Neurabin-1	PPP1R9A	0.47*	0.58*	0.64*	0.46*	0.49*	0.39*	0.47*
A-kinase anchor protein 8-like	AKAP8L	0.64*	0.71	0.65*	0.46*	0.25*	0.36*	0.45*
SOSS complex subunit C	INIP	0.86	0.86	0.75*	0.46*	0.17*	0.19*	0.05*
Pre-mRNA-splicing factor CWC22 homolog	CWC22	0.81	1.00	0.97	0.46*	0.27*	0.32*	0.40*
Activating transcription factor 7-interacting protein 1	ATF7IP	0.27*	0.43*	0.38*	0.47*	0.46*	0.50*	0.46*
RRP12-like protein	RRP12	0.47*	0.46*	0.54*	0.47*	0.45*	0.46*	0.49*
Zinc finger and BTB domain-containing protein 11	ZBTB11	0.25*	0.37*	0.31*	0.47*	0.42*	0.29*	0.49*
Caspase-2	CASP2	0.87	1.00	1.01	0.47*	0.40*	0.49*	0.29*
Interferon regulatory factor 2-binding protein 1	IRF2BP1	1.00	0.92	0.82	0.47*	0.50*	0.43*	0.40*
Echinoderm microtubule-associated protein-like 3	EML3	0.32*	0.50*	0.48*	0.47*	0.21*	0.10*	0.30*
Protein FAM110B	FAM110B	0.57*	0.56*	0.69*	0.47*	0.56*	0.52*	0.45*
Serine/threonine-protein phosphatase 6 regulatory subunit 2	PPP6R2	0.61*	0.71*	0.58*	0.47*	0.49*	0.54*	0.32*
Ribosomal RNA-processing protein 7 homolog A	RRP7A	0.57*	0.49*	0.52*	0.47*	0.39*	0.41*	0.46*
Neural proliferation differentiation and control protein 1	NPDC1	0.46*	0.76	0.88	0.47*	0.26*	0.24*	0.25*
Protein polybromo-1	PBRM1	0.57*	0.81	0.74*	0.47*	0.32*	0.33*	0.27*
Dynein regulatory complex protein 10	IQCD	0.69	2.52*	1.32	0.48*	0.17*	0.13*	0.09*
DNA-binding protein SATB2	SATB2	0.51*	0.92	0.75*	0.48*	0.45*	0.42*	0.63*
Ras-related protein Rab-13	RAB13	1.05	0.65*	0.98	0.48*	0.46*	0.39*	0.43*
Guanine nucleotide-binding protein subunit beta-like protein 1	GNB1L	0.54*	0.61*	0.64*	0.48*	0.50*	0.58	0.57
Protein DENND6B	DENND6B	0.44*	1.30	0.61*	0.48*	0.07*	0.15*	0.24*
E3 ubiquitin-protein ligase RNF113A	RNF113A	0.51*	0.59*	0.63*	0.48*	0.18*	0.21*	0.31*
Neurogenic locus notch homolog protein 1	NOTCH1	0.46*	0.54*	0.85	0.49*	0.63	0.65	0.50*
Biogenesis of lysosome-related organelles complex 1 subunit 3	BLOC1S3	0.30*	0.42*	0.36*	0.49*	0.50*	0.39*	0.62
MAP kinase-activated protein kinase 5	MAPKAPK5	0.66*	0.99	0.88	0.49*	0.63	0.44*	0.45*
E3 ubiquitin-protein ligase TRIM36	TRIM36	0.58*	0.75	0.66*	0.49*	0.45*	0.50*	0.27*
Bifunctional polynucleotide phosphatase/kinase	PNKP	0.87	1.01	0.99	0.49*	0.66	0.45*	0.56*
NFX1-type zinc finger-containing protein 1	ZNFX1	0.46*	0.56*	0.59*	0.49*	0.55*	0.63	0.56*
Synaptojanin-2	SYNJ2	0.61*	0.68*	0.76	0.49*	0.41*	0.32*	0.33*
Coiled-coil domain-containing protein 91	CCDC91	0.46*	0.97	0.95	0.49*	0.54*	0.49*	0.49*
Breast cancer anti-estrogen resistance protein 3	BCAR3	0.89	1.15	1.03	0.49*	0.47*	0.51	0.21*
NCK-interacting protein with SH3 domain	NCKIPSD	0.79	0.71	0.75	0.50*	0.50*	0.44*	0.28*
Probable ribosome biogenesis protein RLP24	RSL24D1	0.58*	0.47*	0.54*	0.50*	0.22*	0.21*	0.27*
Lipid droplet assembly factor 1	LDAF1	0.67*	0.75	0.66	0.50*	0.54	0.57	0.41*
A-kinase anchor protein 8	AKAP8	0.63*	0.53*	0.59*	0.50*	0.42*	0.47*	0.67
Aldo-keto reductase family 1 member C2	AKR1C2	1.21	1.04	0.87	0.50*	0.61	0.49*	0.66

Nucleus accumbens-associated protein 1	NACC1	0.48*	0.68*	0.69*	0.50*	0.31*	0.32*	0.34*
TATA-binding protein-associated factor 172	BTAF1	0.50*	0.62*	0.58*	0.51*	0.58	0.54	0.52*
Transmembrane and coiled-coil domain-containing protein 3	TMCO3	0.76	1.05	1.00	0.51*	0.54	0.66	0.42*
Proteasome subunit beta type-6	PSMB6	0.97	1.02	0.79	0.51*	0.44*	0.52*	0.40*
F-box only protein 44	FBXO44	1.23	1.11	0.97	0.51*	0.38*	0.61	0.44*
Activator of basal transcription 1	ABT1	0.75*	0.79	0.72*	0.51*	0.42*	0.47*	0.48*
Dysferlin	DYSF	0.78*	0.53*	0.82	0.51*	0.36*	0.42*	0.35*
Na(+)/H(+) exchange regulatory cofactor NHE-RF4	NHERF4	0.90	0.93	0.78	0.51*	0.46*	0.43*	0.33*
BLOC-1-related complex subunit 6	BORCS6	1.11	0.98	1.00	0.51*	0.06*	0.30*	0.27*
Probable ATP-dependent RNA helicase DDX52	DDX52	0.71*	0.73	0.79	0.51*	0.59*	0.56*	0.41*
Serine/threonine-protein kinase MRCK alpha	CDC42BPA	0.58*	0.63*	0.57*	0.52*	0.64	0.58*	0.40*
Mitotic deacetylase-associated SANT domain protein	MIDEAS	0.42*	0.58*	0.54*	0.52*	0.31*	0.47*	0.43*
Methionyl-tRNA formyltransferase, mitochondrial	MTFMT	0.82	0.84	0.84	0.52*	0.18*	0.34*	0.16*
Dynamin-binding protein	DNMBP	0.59*	0.48*	0.50*	0.52*	0.66	0.52	0.46*
Protein CDV3 homolog	CDV3	0.77	0.80	0.88	0.52*	0.43*	0.46*	0.52*
ADP-ribosylation factor-like protein 6	ARL6	0.78	0.84	0.91	0.52*	0.36*	0.42*	0.43*
Ral GTPase-activating protein subunit alpha-2	RALGAPA2	0.47*	0.70	0.69*	0.52*	0.48*	0.38*	0.37*
Fatty acyl-CoA reductase 2	FAR2	0.82	0.49*	0.55*	0.52*	0.11*	0.25*	0.27*
RNA 3'-terminal phosphate cyclase-like protein	RCL1	0.68*	0.71*	0.73*	0.52*	0.56*	0.56*	0.58*
Ubiquitin-conjugating enzyme E2 Q1	UBE2Q1	0.72*	0.69*	0.69*	0.53*	0.52*	0.54*	0.38*
Alpha-endosulfine	ENSA	0.47*	0.44*	0.55*	0.53*	0.36*	0.35*	0.55*
Interferon regulatory factor 2	IRF2	0.72*	1.02	1.01	0.53*	0.22*	0.21*	0.29*
Serine/threonine-protein kinase tousled-like 2	TLK2	0.39*	0.59*	0.61*	0.53*	0.32*	0.30*	0.34*
ATPase family gene 2 protein homolog B	AFG2B	0.47*	0.50*	0.62*	0.54*	0.54	0.54	0.60
Coiled-coil domain-containing protein 28A	CCDC28A	0.96	0.91	1.00	0.54*	0.58	0.53	0.53*
APOBEC1 complementation factor	A1CF	1.14	1.10	1.03	0.54*	0.28*	0.54	0.03*
Uncharacterized protein C9orf85	C9orf85	0.49*	0.66	0.86	0.54*	0.21*	0.17*	0.36*
Serine/threonine-protein kinase Sgk2	SGK2	0.83	0.88	0.66*	0.55*	0.63	0.60*	0.50*
Aldo-keto reductase family 1 member B10	AKR1B10	1.02	0.84	0.74*	0.55*	0.62*	0.50*	0.50*
Transmembrane protein 201	TMEM201	0.55*	0.68*	0.64*	0.55*	0.22*	0.38*	0.44*
Calcium-binding and coiled-coil domain-containing protein 2	CALCOCO2	0.47*	0.60*	0.63*	0.55*	0.31*	0.29*	0.33*
Guanine nucleotide-binding protein-like 3	GNL3	0.66*	0.68	0.79	0.55*	0.56	0.46*	0.47*
Periodic tryptophan protein 2 homolog	PWP2	0.60*	0.58*	0.67*	0.55*	0.61*	0.61	0.60*
Proteasome subunit beta type-7	PSMB7	0.97	0.93	0.85	0.55*	0.51*	0.55*	0.47*
tRNA (uracil-5-)-methyltransferase homolog A	TRMT2A	0.55*	0.50*	0.52*	0.55*	0.58	0.55*	0.62
3-hydroxy-3-methylglutaryl-coenzyme A reductase	HMGCR	0.62*	0.67	0.59*	0.55*	0.62	0.57	0.53*

SH3 and PX domain-containing protein 2B	SH3PXD2B	0.86	1.06	1.09	0.55*	0.37*	0.41*	0.59*
Kinetochore-associated protein NSL1 homolog	NSL1	0.53*	0.63*	0.65*	0.56*	0.45*	0.26*	0.50*
Dysbindin	DTNBP1	0.83	0.78	0.88	0.56*	0.64	0.53	0.55
Inactive ubiquitin thioesterase OTULINL	OTULINL	0.97	0.94	0.77	0.56*	0.55*	0.65	0.35*
Trypsin-3	PRSS3	1.35	0.78	1.47	0.56*	0.60	0.49*	0.62
Synergin gamma	SYNRG	0.65*	0.69	0.84	0.56*	0.42*	0.37*	0.55
Zinc finger and BTB domain-containing protein 7A	ZBTB7A	0.57*	0.54*	0.56*	0.56*	0.52*	0.45*	0.56*
tRNA-dihydrouridine(20) synthase [NAD(P)+]-like	DUS2	0.80*	0.85	0.93	0.56*	0.41*	0.35*	0.37*
Phosphatase and actin regulator 2	PHACTR2	0.89	0.87	1.18	0.56*	0.04*	0.47*	0.22*
Coiled-coil domain-containing protein 97	CCDC97	0.49*	0.65	1.06	0.56*	0.43*	0.38*	0.55
NFATC2-interacting protein	NFATC2IP	0.85	0.85	1.09	0.56*	0.21*	0.20*	0.18*
Tetratricopeptide repeat protein 12	TTC12	0.64*	0.64*	0.64*	0.57*	0.54*	0.51*	0.44*
Adipogenesis regulatory factor	ADIRF	1.03	0.94	1.13*	0.57*	0.51*	0.53*	0.62*
Transcription factor Sp3	SP3	0.82	0.80	0.85	0.57*	0.39*	0.63	0.63
Phosphorylase b kinase gamma catalytic chain, liver/testis isoform	PHKG2	0.73	0.76	0.62*	0.58*	0.34*	0.52	0.30*
Mitochondrial mRNA pseudouridine synthase RPUSD3	RPUSD3	0.99	1.04	1.05	0.58*	0.24*	0.31*	0.18*
Zinc finger protein 703	ZNF703	0.68	0.86	0.91	0.58*	0.54	0.33*	0.54
Ribosome quality control complex subunit TCF25	TCF25	0.76*	0.96	0.83	0.58*	0.49*	0.59*	0.41*
Constitutive coactivator of peroxisome proliferator-activated receptor gamma	FAM120B	0.69	0.77	0.74	0.58*	0.59	0.59	0.64
Protein Wiz	WIZ	0.43*	0.61*	0.61*	0.58*	0.15*	0.17*	0.32*
Transcriptional repressor protein YY1	YY1	0.68*	0.92	0.97	0.59*	0.40*	0.51*	0.57*
BRCA1-associated protein	BRAP	0.58*	0.73	0.74	0.59*	0.33*	0.42*	0.44*
Mitochondrial amidoxime-reducing component 1	MTARC1	1.04	0.96	1.13	0.59*	0.21*	0.41*	0.49*
Ubiquitin-associated protein 2	UBAP2	0.54*	0.90	1.00	0.59*	0.07*	0.16*	0.10*
Signal-induced proliferation-associated 1-like protein 3	SIPA1L3	0.73*	0.82	0.70*	0.59*	0.51*	0.48*	0.44*
Ubiquitin-associated protein 1	UBAP1	0.75	0.91	0.87	0.59*	0.25*	0.55	0.49*
Exopolyphosphatase PRUNE1	PRUNE1	0.77	0.86	0.83	0.59*	0.58	0.58	0.49*
tRNA (guanine(6)-N2)-methyltransferase THUMP3	THUMPD3	0.84	0.71*	0.79	0.59*	0.36*	0.41*	0.40*
Zinc finger CCHC domain-containing protein 8	ZCCHC8	0.56*	0.55*	0.56*	0.60*	0.26*	0.25*	0.39*
Cell division cycle protein 123 homolog	CDC123	0.72*	0.81	0.88	0.60*	0.49*	0.57*	0.44*
DNA ligase 3	LIG3	0.85	0.86	0.93	0.60*	0.48*	0.62*	0.56*
Nucleolar MIF4G domain-containing protein 1	NOM1	0.38*	0.40*	0.40*	0.60*	0.37*	0.27*	0.32*
Pyrin	MEFV	0.88	0.88	1.30	0.60*	0.60	0.51*	0.60*
Paladin	PALD1	0.51*	0.59*	0.69*	0.60*	0.46*	0.56*	0.54*
Acyl-CoA-binding domain-containing protein 4	ACBD4	1.11	1.04	0.85	0.61*	0.56*	0.54*	0.39*
Actin filament-associated protein 1	AFAP1	0.66	1.00	0.86	0.61*	0.41*	0.40*	0.55

ATP-dependent RNA helicase DDX3Y	DDX3Y	0.68	0.66	0.87	0.61*	0.51*	0.52*	0.55*
Trefoil factor 1	TFF1	1.54*	1.08	0.91	0.61*	0.63*	0.48*	0.37*
Butyrophilin-like protein 8	BTNL8	0.98	1.50*	1.43*	0.61*	0.47*	0.65*	0.47*
Arf-GAP domain and FG repeat-containing protein 2	AGFG2	0.81	0.62*	0.76	0.61*	0.61	0.45*	0.54
Kelch repeat and BTB domain-containing protein 11	KBTBD11	0.89	0.77	0.94	0.61*	0.58	0.54	0.63
FLYWCH family member 2	FLYWCH2	1.10	1.08	1.06	0.61*	0.19*	0.22*	0.18*
Ribosomal protein S6 kinase alpha-4	RPS6KA4	0.69*	0.47*	0.63*	0.61*	0.49*	0.63	0.55*
RNA-binding protein NOB1	NOB1	0.59*	0.55*	0.71*	0.61*	0.48*	0.50*	0.54*
Transmembrane channel-like protein 6	TMC6	0.59*	0.47*	0.51*	0.62*	0.63	0.64	0.61
Protein FAM83F	FAM83F	0.88	0.91	0.86	0.62*	0.54*	0.54*	0.48*
E3 ubiquitin-protein ligase BRE1B	RNF40	0.62*	0.66*	0.61*	0.62*	0.57*	0.49*	0.64
DNA/RNA-binding protein KIN17	KIN	0.94	1.01	1.03	0.62*	0.12*	0.36*	0.39*
RNA polymerase II subunit A C-terminal domain phosphatase	CTDP1	0.63*	0.67*	0.70*	0.62*	0.63	0.55*	0.62
EKC/KEOPS complex subunit LAGE3	LAGE3	0.82	1.02	1.07	0.62*	0.23*	0.19*	0.31*
Peptidyl-prolyl cis-trans isomerase-like 4	PPIL4	1.02	0.84	0.74	0.62*	0.23*	0.22*	0.30*
Zinc finger C2HC domain-containing protein 1A	ZC2HC1A	0.63*	0.65*	0.81	0.62*	0.45*	0.51*	0.54*
Neurabin-2	PPP1R9B	0.74	0.80	0.85	0.62*	0.62	0.60	0.57
Dematin	DMTN	0.78	1.09	1.13	0.62*	0.06*	0.12*	0.23*
Protein FAM13A	FAM13A	0.92	1.01	0.96	0.63*	0.33*	0.35*	0.40*
Docking protein 1	DOK1	0.79	0.84	0.98	0.63*	0.38*	0.46*	0.44*
Diacylglycerol kinase theta	DGKQ	0.66	1.19	0.97	0.63*	0.46*	0.64	0.53*
Tripartite motif-containing protein 26	TRIM26	0.56*	0.73*	0.71*	0.63*	0.53*	0.56*	0.62*
Chromobox protein homolog 8	CBX8	0.49*	0.66*	0.71*	0.63*	0.26*	0.20*	0.51*
Ras GTPase-activating-like protein IQGAP2	IQGAP2	0.95	0.78*	0.90	0.63*	0.59*	0.59*	0.59*
5'-AMP-activated protein kinase subunit beta-2	PRKAB2	0.76*	1.08	1.05	0.63*	0.41*	0.60	0.58*
Thiol S-methyltransferase TMT1B	TMT1B	0.95	0.99	0.81	0.63*	0.66	0.63	0.56*
Inositol 1,4,5-trisphosphate receptor type 2	ITPR2	0.94	0.85	0.65*	0.64	0.58	0.66	0.55
Zinc finger protein 768	ZNF768	0.73	0.67*	0.83	0.64*	0.19*	0.28*	0.47*
Breast carcinoma-amplified sequence 1	BCAS1	0.92	0.79	1.02	0.64*	0.57*	0.51*	0.59*
Translation initiation factor eIF-2B subunit delta	EIF2B4	0.62*	0.59*	0.55*	0.64*	0.57*	0.57*	0.61*
Uncharacterized protein C2orf72	C2orf72	1.13	0.75	0.66*	0.64*	0.05*	0.39*	0.08*
Remodeling and spacing factor 1	RSF1	0.54*	0.63*	0.57*	0.64*	0.40*	0.41*	0.53*
La-related protein 1B	LARP1B	0.95	0.73	0.88	0.64	0.59	0.59	0.59
Motile sperm domain-containing protein 1	MOSPD1	1.07	1.64*	1.30	0.65	0.27*	0.58	0.13*
Large subunit GTPase 1 homolog	LSG1	0.76*	0.64*	0.66*	0.65*	0.54*	0.52*	0.52*
Proline and serine-rich protein 2	PROSER2	0.90	0.94	1.01	0.65	0.32*	0.42*	0.44*
Transcription termination factor 3, mitochondrial	MTERF3	1.00	1.19	1.30*	0.65*	0.24*	0.33*	0.40*

Nonsense-mediated mRNA decay factor SMG9	SMG9	0.69*	0.82	0.91	0.65*	0.33*	0.55*	0.61
Protein furry homolog	FRY	0.34*	0.64*	0.59*	0.65	0.30*	0.36*	0.55
Zinc finger protein 346	ZNF346	0.71	0.71	0.77	0.65	0.14*	0.30*	0.51*
Coiled-coil domain-containing protein 43	CCDC43	1.16	1.24	1.32	0.65	0.08*	0.31*	0.22*
Rab5 GDP/GTP exchange factor	RABGEF1	0.67*	0.92	0.93	0.65	0.50*	0.62	0.53
tRNA N(3)-methylcytidine methyltransferase METTL2B	METTL2B	0.79*	0.90	0.86	0.65*	0.30*	0.36*	0.31*
Elongator complex protein 4	ELP4	0.60*	0.73	0.75	0.66	0.60	0.53	0.49*
Glutathione S-transferase C-terminal domain-containing protein	GSTCD	0.71*	0.72*	0.72*	0.66*	0.61*	0.63	0.58*
Transcription elongation factor A protein 3	TCEA3	0.93	1.00	0.97	0.66	0.33*	0.51*	0.60
DNA-directed RNA polymerase I subunit RPA49	POLR1E	0.80	0.77	0.76	0.66	0.64	0.64	0.55
SH2 domain-containing protein 3A	SH2D3A	0.73*	0.66*	0.61*	0.66*	0.45*	0.51*	0.50*
Gastrotropin	FABP6	1.05	0.52*	0.43*	0.66*	0.55*	0.35*	0.48*
Valine--tRNA ligase, mitochondrial	VARS2	1.06	0.83	0.74*	0.66*	0.41*	0.44*	0.30*
Regenerating islet-derived protein 4	REG4	0.73*	0.56*	0.98	0.66*	0.45*	0.49*	0.52*
RNA polymerase-associated protein LEO1	LEO1	0.98	1.02	0.90	0.67	0.22*	0.41*	0.30*
Dynein axonemal heavy chain 17	DNAH17	1.74*	2.19*	1.86*	0.67	0.06*	0.20*	0.23*

B. Down-regulated proteins unique to LPS-Cytokines (LPS-Cyto) [96 proteins]

		Interventions						
		Control			*With LPS & Cytokines*			
Proteins	Genes	AQ	AQ+MES	MES	***LPS-Cyto***	*AQ*	*AQ+MES*	*MES*
Gamma-tubulin complex component 3	TUBGCP3	0.19*	0.27*	0.29*	0.31*	0.86	0.80	0.82
Mediator of RNA polymerase II transcription subunit 23	MED23	0.28*	0.39*	0.32*	0.32*	0.85	0.69	0.70
Integrator complex subunit 5	INTS5	0.39*	0.34*	0.33*	0.35*	0.86	0.81	0.84
Serine/threonine-protein kinase WNK2	WNK2	0.36*	0.74	0.58*	0.39*	0.72	0.77	0.79
Histone-lysine N-methyltransferase EHMT2	EHMT2	0.25*	0.28*	0.29*	0.40*	1.50	1.10	1.16
Cyclin-dependent kinase 1	CDK1	0.62*	0.67*	0.68*	0.40*	1.51	1.34	1.54*
Structural maintenance of chromosomes protein 6	SMC6	0.28*	0.28*	0.27*	0.41*	1.10	0.92	1.02
Repetin	RPTN	1.60*	1.00	4.84*	0.42*	2.18*	0.67	3.40*
Mediator of RNA polymerase II transcription subunit 12	MED12	0.25*	0.27*	0.31*	0.42*	0.97	0.81	0.84
Small subunit processome component 20 homolog	UTP20	0.39*	0.36*	0.37*	0.42*	0.86	0.84	1.24
Transmembrane protein 209	TMEM209	0.34*	0.43*	0.44*	0.42*	1.00	0.80	0.87
WD repeat-containing protein 3	WDR3	0.37*	0.34*	0.36*	0.43*	0.82	0.73	0.70*
Keratin, type I cytoskeletal 23	KRT23	2.31*	1.81*	6.31*	0.44*	2.65*	0.70	2.04*
Pre-mRNA-processing factor 39	PRPF39	0.97	1.06	0.98	0.46*	0.82	0.98	0.80
Endoribonuclease ZC3H12A	ZC3H12A	0.56*	0.69*	0.73*	0.46*	0.80	0.84	0.70

Progesterone-induced-blocking factor 1	PIBF1	0.51*	0.67*	0.65*	0.46*	0.97	1.00	0.67
Structural maintenance of chromosomes protein 4	SMC4	0.46*	0.45*	0.55*	0.46*	1.22	1.09	0.96
FERM and PDZ domain-containing protein 1	FRMPD1	0.54*	2.61*	2.58*	0.47*	0.94	1.85*	2.22*
Arachidonate 12-lipoxygenase, 12R-type	ALOX12B	2.22*	1.37	7.85*	0.47*	2.58*	0.92	1.08
CDK5 and ABL1 enzyme substrate 1	CABLES1	0.48*	0.56*	0.68*	0.48*	1.04	0.89	0.90
Calpain-8	CAPN8	0.70	0.94	0.81	0.48*	0.91	0.83	0.88
E3 ubiquitin-protein ligase TRIM32	TRIM32	0.54*	0.56*	0.56*	0.48*	0.95	0.87	0.72
Probable helicase with zinc finger domain	HELZ	0.61*	0.70	0.67*	0.49*	1.11	1.01	1.01
Integrator complex subunit 7	INTS7	0.46*	0.58*	0.57*	0.49*	1.12	0.88	0.83
Alkaline phosphatase, placental type	ALPP	0.98	0.67*	0.93	0.51*	1.19	1.61	1.36
Probable ATP-dependent RNA helicase DDX20	DDX20	0.33*	0.54*	0.49*	0.51*	0.75	0.70	0.73
Kynureninase	KYNU	0.82	1.00	1.61*	0.52*	0.92	1.21	1.74*
Protein PALS2	PALS2	0.98	0.52*	0.71	0.52*	1.81*	1.54	1.29
Endoribonuclease Dicer	DICER1	0.58*	0.75	0.84	0.53*	0.94	0.86	0.85
Retrotransposon Gag-like protein 8C	RTL8C	0.91	1.18	1.12	0.53*	0.73	0.85	0.85
Anaphase-promoting complex subunit 5	ANAPC5	0.39*	0.38*	0.39*	0.53*	1.56*	1.01	1.02
Probable ATP-dependent RNA helicase DDX10	DDX10	0.89	0.78	0.82	0.53*	0.89	0.84	0.75
MYND-type zinc finger-containing chromatin reader ZMYND8	ZMYND8	0.68	0.70	0.69	0.54*	0.69	0.82	1.45
BTB/POZ domain-containing protein KCTD3	KCTD3	0.53*	0.58*	0.51*	0.54*	0.97	1.07	0.69
PWWP domain-containing DNA repair factor 4	PWWP4	1.54*	1.13	1.24	0.54*	1.27	1.13	0.67
Transformation/transcription domain-associated protein	TRRAP	0.30*	0.35*	0.40*	0.54*	1.21	1.16	1.04
Protein piccolo	PCLO	0.56*	1.75*	2.05*	0.55*	0.72	2.03*	2.36*
Nitric oxide-associated protein 1	NOA1	0.95	1.02	1.02	0.55*	0.82	0.71	0.87
UDP-N-acetylhexosamine pyrophosphorylase-like protein 1	UAP1L1	0.69*	0.81	0.74*	0.55*	1.08	0.98	0.90
Microtubule-associated tumor suppressor 1	MTUS1	0.55*	0.71	0.59*	0.56*	0.91	0.83	0.68
PH-interacting protein	PHIP	0.81	0.72	0.81	0.56*	0.82	0.81	0.68
Baculoviral IAP repeat-containing protein 2	BIRC2	0.41*	0.58*	0.49*	0.57*	1.37	1.08	1.31
Target of EGR1 protein 1	TOE1	0.59*	0.60*	0.69*	0.57*	0.94	0.84	0.79
Cerebellin-4	CBLN4	0.41*	1.58	2.52*	0.57*	0.78	1.73	1.95*
TNF receptor-associated factor 2	TRAF2	0.52*	0.60*	0.58*	0.57*	0.95	0.93	0.89
Protein S100-A3	S100A3	1.12	26.05*	2.82*	0.58*	1.60	1.77	3.12*
Serine/threonine-protein kinase RIO3	RIOK3	0.81	0.76	0.69	0.58*	0.85	0.71	0.80
Single-stranded DNA-binding protein 3	SSBP3	0.56*	0.77	0.66*	0.58*	1.24	1.30	1.34
DNA mismatch repair protein Mlh3	MLH3	1.28	0.99	1.18	0.58*	1.07	0.82	0.87
mRNA-decapping enzyme 1B	DCP1B	1.26	2.01*	1.92*	0.58*	1.35	0.76	1.52

Single-stranded DNA-binding protein 4	SSBP4	0.65*	0.96	0.80	0.58*	1.08	1.15	1.07
Desmoglein-3	DSG3	0.68	3.24*	4.23*	0.59	0.94	1.57	2.39*
Serine/threonine-protein kinase 31	STK31	2.24*	1.32	4.57*	0.59*	2.06*	0.73	1.99*
Ras-related protein Rab-6B	RAB6B	1.14	0.98	0.99	0.59*	0.74	0.70	0.73
Integrator complex subunit 11	INTS11	0.56*	0.66*	0.68*	0.59*	0.68	0.79	0.96
Non-structural maintenance of chromosomes element 3 homolog	NSMCE3	0.56*	0.62*	0.61*	0.60*	0.87	0.88	0.88
Anaphase-promoting complex subunit 1	ANAPC1	0.33*	0.29*	0.32*	0.60*	2.35*	1.97*	2.10*
DNA-directed RNA polymerase II subunit RPB2	POLR2B	0.86	0.81	0.74	0.60*	0.80	0.76	0.85
ATP-dependent RNA helicase DDX19B	DDX19B	1.02	1.19	1.08	0.60*	0.92	1.22	0.87
Sin3 histone deacetylase corepressor complex component SDS3	SUDS3	0.45*	0.57*	0.52*	0.61*	0.92	0.93	0.83
Histidine-rich glycoprotein	HRG	0.76	1.27	4.14*	0.61	0.90	1.03	1.39
Unconventional myosin-IXb	MYO9B	0.70*	0.80	0.75*	0.61*	0.88	0.89	0.78
Integrator complex subunit 4	INTS4	0.39*	0.43*	0.46*	0.61*	1.10	1.22	1.14
Pericentriolar material 1 protein	PCM1	0.68*	0.68*	0.65*	0.61*	0.75	0.76	0.69
ER lumen protein-retaining receptor 1	KDELR1	0.93	0.91	1.01	0.61*	0.99	1.19	0.93
Mediator of RNA polymerase II transcription subunit 22	MED22	0.54*	0.67	0.61*	0.62*	1.13	1.42	1.22
Glycerol-3-phosphate acyltransferase 4	GPAT4	1.16	1.21	1.18	0.63*	0.96	0.87	0.70
Serine/threonine-protein kinase PAK 5	PAK5	1.18	0.85	0.91	0.63	0.94	1.28	0.83
Ribonuclease P protein subunit p29	POP4	0.51*	0.61*	0.62*	0.63*	1.31	1.21	1.20
Cell adhesion molecule 1	CADM1	0.65*	1.89*	2.04*	0.63	0.89	1.76*	2.25*
Replication factor C subunit 4	RFC4	0.59*	0.80	0.92	0.63*	0.81	0.77	0.95
Guanine nucleotide-binding protein subunit alpha-15	GNA15	0.90	2.12*	3.41*	0.64	1.28	1.26	1.03
DNA topoisomerase 3-beta-1	TOP3B	0.68	0.69	0.76	0.64*	0.82	0.74	0.68
Tetratricopeptide repeat protein 13	TTC13	0.85	1.07	1.31	0.64	1.18	1.21	1.20
Heat shock-related 70 kDa protein 2	HSPA2	0.97	1.11	1.15	0.64*	0.76	0.82	0.72
Dermokine	DMKN	0.47*	0.70	0.58*	0.64*	1.58	0.79	1.33
GEM-interacting protein	GMIP	0.91	0.79	0.86	0.64	0.71	0.75	0.88
Protein phosphatase 1 regulatory subunit 14C	PPP1R14C	1.14	0.99	1.29	0.65	0.96	0.84	0.85
RAB6A-GEF complex partner protein 2	RGP1	0.41*	0.67	0.93	0.65	0.92	1.04	0.97
eIF-2-alpha kinase GCN2	EIF2AK4	0.51*	0.63	0.70	0.65	0.82	0.77	0.79
A disintegrin and metalloproteinase with thrombospondin motifs 13	ADAMTS13	0.68	2.03*	2.82*	0.65	0.85	2.28*	2.94*
Mitochondrial antiviral-signaling protein	MAVS	0.91	1.11	1.16	0.65*	0.70	0.89	0.89
Death-associated protein kinase 2	DAPK2	0.99	0.95	0.94	0.65	1.31	0.93	0.96
TBC1 domain family member 17	TBC1D17	0.73	0.69	0.65*	0.65*	0.98	0.78	0.76
B-cell lymphoma/leukemia 11B	BCL11B	0.76	0.76	0.86	0.65	0.86	0.79	0.99

Keratin, type I cuticular Ha1	KRT31	0.68*	49.23*	2.04*	0.65*	0.92	0.80	2.96*
Renin	REN	1.34	1.56*	1.57*	0.66	0.96	0.87	0.83
Myelin expression factor 2	MYEF2	0.70*	1.05	1.05	0.66*	1.57	1.32	1.36
DDB1- and CUL4-associated factor 6	DCAF6	0.75	0.86	1.02	0.66	0.91	1.16	0.82
Immunoglobulin heavy constant gamma 4	IGHG4	0.64*	1.95*	32.94*	0.66*	0.72	1.67*	0.96
Leucine-rich repeat-containing protein 75A	LRRC75A	0.79	1.07	1.06	0.66	0.68	0.75	0.72
Retinoic acid receptor responder protein 2	RARRES2	0.54*	1.61*	2.51*	0.66	0.68	1.46	2.08*
15-hydroxyprostaglandin dehydrogenase [NAD(+)]	HPGD	1.05	1.10	1.06	0.67*	0.71	0.85	0.80
Translation factor GUF1, mitochondrial	GUF1	0.81	0.67*	0.68*	0.67*	0.68	0.81	0.86
Protein Daple	CCDC88C	0.91	0.72*	0.75*	0.67*	0.86	0.69	0.69
U3 small nucleolar RNA-associated protein 6 homolog	UTP6	0.56*	0.49*	0.59*	0.67	0.68	0.68	0.75

C. Down-regulated proteins unique to Aquamin (AQ) with LPS-Cytokines [137 proteins]

		Interventions						
		Control			*With LPS & Cytokines*			
Proteins	Genes	AQ	AQ+MES	MES	*LPS-Cyto*	***AQ***	*AQ+MES*	*MES*
Nuclear envelope pore membrane protein POM 121	POM121	0.75	1.26	1.56*	0.87	0.22*	1.01	1.38
Polyglutamine-binding protein 1	PQBP1	1.06	1.73*	1.85*	1.25	0.26*	0.69	1.16
Protein Aster-B	GRAMD1B	0.77	0.85	0.73	0.83	0.35*	0.83	0.90
Chromatin modification-related protein MEAF6	MEAF6	0.73	0.77	0.68*	1.03	0.46*	0.77	0.86
Protein C10	C12orf57	0.85	0.95	1.28	1.09	0.46*	0.72	0.88
TNFAIP3-interacting protein 1	TNIP1	0.92	1.07	1.14	0.73	0.47*	0.83	0.97
Insulin-like growth factor II	IGF2	0.62*	2.07*	2.04*	0.74	0.48*	1.65*	2.22*
Pre-mRNA 3'-end-processing factor FIP1	FIP1L1	0.92	0.96	0.98	0.75	0.48*	0.67	1.03
Protein O-glucosyltransferase 3	POGLUT3	1.62*	1.67*	1.49*	0.96	0.48*	0.78	1.14
Upstream stimulatory factor 1	USF1	0.92	0.94	1.04	0.77	0.49*	0.71	0.86
Peptidyl-prolyl cis-trans isomerase G	PPIG	0.85	0.81	0.98	0.96	0.49*	0.82	1.10
Neurotensin/neuromedin N	NTS	0.74	1.54*	1.30	1.01	0.50*	1.03	0.72
Large ribosomal subunit protein bL32m	MRPL32	1.35*	1.33	1.23	1.38	0.50*	0.88	1.01
Transcriptional repressor p66-alpha	GATAD2A	0.64*	0.72	0.99	0.78	0.50*	0.69	0.85
Regulator of G-protein signaling 10	RGS10	1.14	1.18	1.22	1.43	0.50*	0.92	0.86
Spliceosome-associated protein CWC27 homolog	CWC27	2.02*	1.81*	1.94*	1.33	0.51	0.99	2.11*
CD2 antigen cytoplasmic tail-binding protein 2	CD2BP2	1.03	1.11	1.35	1.15	0.52*	0.93	1.03
CDK-activating kinase assembly factor MAT1	MNAT1	0.53*	0.93	1.05	0.79	0.52	0.89	1.51
Biogenesis of lysosome-related organelles complex 1 subunit 5	BLOC1S5	0.92	0.82	1.14	0.96	0.52	0.73	0.94
SAP domain-containing ribonucleoprotein	SARNP	1.10	1.28	1.51*	0.98	0.53*	0.92	0.93

Protein LSM14 homolog A	LSM14A	0.79	0.85	1.16	0.92	0.53	0.74	1.02
EGF-containing fibulin-like extracellular matrix protein 1	EFEMP1	0.62*	2.26*	2.51*	0.81	0.53*	1.88*	2.37*
DNA polymerase beta	POLB	0.99	1.03	1.08	1.13	0.53*	0.70	0.85
Serine/Arginine-related protein 53	RSRC1	0.97	1.19	1.35	1.16	0.53*	0.75	1.14
Ribosomal protein eL22-like	RPL22L1	0.45*	0.89	1.44	0.86	0.54	1.05	1.90*
Ubiquitin carboxyl-terminal hydrolase 17-like protein 15	USP17L15	1.01	0.98	0.76*	0.89	0.54*	0.79	0.99
Uncharacterized protein C7orf50	C7orf50	0.69	1.04	1.15	1.07	0.54	0.84	1.23
Protein TASOR	TASOR	0.74	0.65	0.64*	0.68	0.54	0.68	0.76
CCHC-type zinc finger nucleic acid binding protein	CNBP	0.62*	0.99	0.95	1.03	0.55*	0.93	1.04
E3 ubiquitin-protein ligase TRIM47	TRIM47	1.07	0.74	0.89	0.70	0.55	0.67	0.72
MAPK regulated corepressor interacting protein 2	MCRIP2	0.94	1.03	1.06	0.69	0.55	0.78	1.01
Calpain-15	CAPN15	0.79	0.74	0.87	0.77	0.55	0.69	0.82
Methylated-DNA--protein-cysteine methyltransferase	MGMT	0.91	1.07	1.06	0.80	0.55*	0.67	0.68
Interferon regulatory factor 2-binding protein 2	IRF2BP2	0.90	1.10	0.99	0.83	0.56	0.74	0.86
LYR motif-containing protein 1	LYRM1	0.95	1.23	1.16	0.90	0.56	0.72	0.85
Elongin-A	ELOA	0.98	1.04	1.25*	1.07	0.56*	0.68	1.05
Caveolae-associated protein 3	CAVIN3	1.26	1.02	1.14	0.88	0.56	0.67	0.82
PHD and RING finger domain-containing protein 1	PHRF1	0.89	0.83	0.97	0.76	0.56	0.70	0.83
Pre-mRNA-splicing factor ISY1 homolog	ISY1	0.93	1.03	0.99	1.04	0.56*	0.70	0.90
Nik-related protein kinase	NRK	0.87	0.59*	0.80	1.03	0.57	0.92	1.26
NudC domain-containing protein 3	NUDCD3	0.96	0.69	1.21	1.09	0.57	0.80	1.01
Thymosin beta-10	TMSB10	0.55*	0.69	1.12	1.08	0.57	0.67	0.93
Nucleolar protein 4-like	NOL4L	0.74	0.81	0.72	0.74	0.58	0.73	0.71
Transforming acidic coiled-coil-containing protein 2	TACC2	1.05	0.98	1.01	0.83	0.58	0.69	0.78
Ferritin light chain	FTL	0.52*	2.72*	3.90*	1.95*	0.58	4.12*	9.36*
Complement factor D	CFD	0.59*	1.43	3.72*	0.81	0.58	1.54	2.05*
Rab GTPase-activating protein 1-like, isoform 10	RABGAP1L	0.99	0.79	1.10	0.99	0.58	0.68	0.68
[Pyruvate dehydrogenase [acetyl-transferring]]-phosphatase 2, mitochondrial	PDP2	1.70*	1.56*	1.29	1.09	0.59	0.86	0.90
MRG/MORF4L-binding protein	MRGBP	1.05	0.96	1.20	1.19	0.59	0.95	0.98
Protein IWS1 homolog	IWS1	1.07	0.99	1.21	0.95	0.59*	0.80	0.92
Probable dimethyladenosine transferase	DIMT1	0.65*	0.59*	0.64*	0.70	0.59	0.75	0.90
ABC-type oligopeptide transporter ABCB9	ABCB9	0.40*	0.67	1.05	1.77*	0.59	0.93	2.42*
Transcription initiation factor IIB	GTF2B	0.93	0.87	0.79	0.79	0.59	0.80	0.83
Signal transducing adapter molecule 2	STAM2	1.02	0.88	1.07	0.83	0.59	0.70	0.90
Axin interactor, dorsalization-associated protein	AIDA	0.80	0.86	0.90	1.01	0.60	0.75	0.84
Exosome component 10	EXOSC10	0.98	0.95	0.99	1.16	0.60	0.67	0.93

Probable glutamate--tRNA ligase, mitochondrial	EARS2	0.88	0.94	0.94	0.76	0.60	0.80	0.73
Cyclin-dependent kinase 12	CDK12	0.76	0.75	0.79	0.78	0.60	0.71	0.72
Keratin, type II cytoskeletal 7	KRT7	1.02	1.08	1.12*	0.93	0.60*	0.84	0.89
DNA replication licensing factor MCM5	MCM5	0.54*	0.55*	0.65*	0.72	0.60	0.67	1.03
Protein phosphatase 1 regulatory subunit 14B	PPP1R14B	0.87	1.14	0.95	1.07	0.60	1.03	0.93
Protein S100-A7	S100A7	0.33*	0.70	0.94	1.71*	0.60	1.01	1.30
Microtubule-associated serine/threonine-protein kinase 4	MAST4	1.07	0.86	1.26	0.73	0.60	0.72	0.94
Telomerase RNA component interacting RNase	TRIR	0.74	0.98	1.04	0.84	0.61	0.85	0.85
E3 ubiquitin-protein ligase UHRF2	UHRF2	0.79	0.63	1.05	1.05	0.61	0.74	0.85
N-alpha-acetyltransferase 20	NAA20	0.97	1.21	1.16	1.11	0.61*	0.84	1.01
Segment polarity protein dishevelled homolog DVL-1	DVL1	0.77	0.80	0.68*	0.75	0.61	0.79	0.84
Negative elongation factor A	NELFA	1.04	1.11	1.26	0.88	0.61	0.69	0.85
Ubiquitin-associated protein 2-like	UBAP2L	0.83	0.94	1.39*	0.90	0.61*	0.76	1.09
Splicing factor 45	RBM17	0.93	0.98	0.93	1.00	0.62	0.83	0.97
Complement factor H	CFH	0.62*	2.03*	27.05*	0.74	0.62	1.68*	1.72*
START domain-containing protein 10	STARD10	0.87	0.93	0.72*	0.79	0.62	0.78	0.78
ADP-ribose glycohydrolase OARD1	OARD1	0.92	0.80	0.87	0.88	0.62	0.72	0.75
WD repeat domain phosphoinositide-interacting protein 4	WDR45	0.99	0.70	0.58*	0.84	0.62	0.94	0.79
RCC1-like G exchanging factor-like protein	RCC1L	1.23	1.34	1.22	1.17	0.62	0.70	0.69
Serine/threonine-protein kinase VRK1	VRK1	0.78*	0.82	0.76*	0.77*	0.62*	0.67*	0.79
Zinc finger protein 185	ZNF185	0.88	0.90	1.10	0.81	0.62	0.80	0.77
Rab proteins geranylgeranyltransferase component A 2	CHML	1.17	1.18	1.06	1.06	0.62	0.68	0.70
Dynein axonemal heavy chain 1	DNAH1	0.51*	1.54*	2.15*	1.10	0.62*	2.04*	2.51*
Importin subunit alpha-7	KPNA6	0.83	0.83	0.94	0.80	0.62	0.67	0.92
Mitotic-spindle organizing protein 1	MZT1	1.14	1.09	2.73*	0.92	0.63	0.71	2.13*
Prolyl endopeptidase-like	PREPL	0.82	0.84	0.74	0.74	0.63	0.80	0.67
Large ribosomal subunit protein eL24	RPL24	0.78*	0.79	0.91	0.75*	0.63*	0.74	0.97
AP2-associated protein kinase 1	AAK1	0.97	0.99	0.91	0.85	0.63*	0.80	0.90
Magnesium-dependent phosphatase 1	MDP1	1.09	1.23	1.15	0.96	0.63	0.75	0.84
Nucleolar GTP-binding protein 2	GNL2	0.79	1.10	0.81	1.06	0.63	0.75	1.19
Peptidyl-prolyl cis-trans isomerase NIMA-interacting 4	PIN4	0.96	1.03	1.04	0.95	0.63	0.92	0.96
Pre-mRNA-splicing factor 38A	PRPF38A	1.16	1.06	1.00	1.01	0.63	0.77	0.71
Inhibitor of Bruton tyrosine kinase	IBTK	0.71	0.73	0.70	0.74	0.63	0.70	0.77
Protein SCAF11	SCAF11	0.77	0.71	0.80	0.74	0.63	0.73	0.75
Nucleoprotein TPR	TPR	0.93	0.93	1.03	1.02	0.63*	0.77	0.91

Segment polarity protein dishevelled homolog DVL-2	DVL2	1.07	1.18	1.11	1.08	0.63	0.70	0.96
Uncharacterized protein C1orf21	C1orf21	1.00	1.07	1.02	1.18	0.63	0.80	0.89
Zinc finger protein castor homolog 1	CASZ1	0.92	0.95	0.79	0.78	0.63	0.78	0.70
Zinc finger CCCH domain-containing protein 18	ZC3H18	0.84	0.69	0.99	0.94	0.63	0.76	0.87
Ras GTPase-activating protein 2	RASA2	0.84	0.82	0.94	0.75	0.64	0.82	0.77
Negative elongation factor B	NELFB	0.86	0.85	0.85	0.86	0.64	0.78	0.69
PDZ and LIM domain protein 2	PDLIM2	0.86	1.11	1.11	0.84	0.64	0.78	0.90
Histone deacetylase 8	HDAC8	0.95	0.98	0.89	0.91	0.64	0.79	0.74
Lysophosphatidylcholine acyltransferase 2	LPCAT2	1.02	1.01	1.02	0.81	0.64	0.68	0.73
Serine/threonine-protein phosphatase 1 regulatory subunit 10	PPP1R10	1.08	0.91	1.17	1.31	0.64	0.75	0.99
SH3 domain-binding protein 5-like	SH3BP5L	1.15	1.08	1.06	1.07	0.64	0.88	0.89
DNA-directed RNA polymerase I subunit RPA2	POLR1B	0.75	0.69	0.76	0.76	0.64	0.81	0.84
FYVE and coiled-coil domain-containing protein 1	FYCO1	0.95	0.85	0.89	0.73*	0.64*	0.76	0.67*
AFG1-like ATPase	AFG1L	1.06	0.82	0.71	0.82	0.64	0.77	0.86
Nitric oxide synthase-interacting protein	NOSIP	0.68*	0.84	0.92	0.78	0.65	0.69	0.92
High mobility group protein 20A	HMG20A	0.84	0.90	0.92	0.94	0.65	0.69	0.99
Collagen alpha-2(I) chain	COL1A2	0.53*	2.15*	2.47*	0.72	0.65	1.88*	2.71*
mRNA cap guanine-N7 methyltransferase	RNMT	1.08	0.83	0.97	0.83	0.65	0.78	0.85
Sesquipedalian-1	PHETA1	0.89	1.00	0.85	0.89	0.65	0.76	0.70
Alcohol dehydrogenase 1C	ADH1C	0.94	0.94	0.70*	0.95	0.65*	0.72*	1.22*
Jupiter microtubule associated homolog 2	JPT2	0.73	0.98	0.90	0.77	0.65	0.70	0.83
Endothelial differentiation-related factor 1	EDF1	0.86	0.81	1.04	0.89	0.65	0.72	0.90
Islet cell autoantigen 1	ICA1	0.87	0.81	0.93	0.70*	0.65	0.76	0.74
Cell death regulator Aven	AVEN	0.76*	0.71*	0.76*	0.80	0.65	0.70	0.73
Zinc finger CCCH domain-containing protein 11A	ZC3H11A	0.80	1.03	1.07	0.74	0.65	0.67	0.71
Microtubule-associated protein 4	MAP4	1.03	0.96	1.19*	0.75*	0.65*	0.67*	0.84
Nuclear cap-binding protein subunit 2	NCBP2	0.94	1.01	1.11	1.06	0.65	0.75	1.18
UBAP1-MVB12-associated (UMA)-domain containing protein 1	UMAD1	0.77	0.75	0.90	0.85	0.65	0.80	0.72
SCY1-like protein 2	SCYL2	1.02	0.98	1.17	0.98	0.65	0.78	0.89
Thyrotroph embryonic factor	TEF	1.06	0.95	0.91	0.90	0.65	0.85	0.96
dCTP pyrophosphatase 1	DCTPP1	0.86	0.75	0.70*	0.86	0.66	0.77	0.84
Trafficking protein particle complex subunit 14	TRAPPC14	1.04	0.81	0.89	0.91	0.66	0.96	0.79
Small ribosomal subunit protein uS12m	MRPS12	0.94	0.74	1.12	0.90	0.66	0.71	1.01
Tripartite motif-containing protein 2	TRIM2	1.00	0.85	0.86	0.81	0.66	0.74	0.74
Sulfotransferase 2B1	SULT2B1	0.98	0.88	1.25	0.75	0.66	0.77	0.83
B-cell CLL/lymphoma 7 protein family member A	BCL7A	0.98	1.13	1.15	1.43	0.66	0.83	1.23

Nuclear factor NF-kappa-B p105 subunit	NFKB1	0.95	0.97	1.04	0.90	0.66	0.81	0.97
Exportin-6	XPO6	0.67	0.58*	0.69	0.91	0.66	0.82	0.82
Filamin-binding LIM protein 1	FBLIM1	0.77	0.92	0.79	0.93	0.66	0.87	0.74
Protein FAM83B	FAM83B	0.84	0.75	1.00	0.98	0.66	0.90	1.07
Protein PRRC2C	PRRC2C	0.87	0.87	1.02	0.88	0.67	0.80	0.98
Plectin	PLEC	1.17	0.85	0.67*	0.71*	0.67*	0.75	0.94
Mitotic interactor and substrate of PLK1	MISP	0.92	1.07	1.11	0.92	0.67*	0.89	1.06
Suppressor of SWI4 1 homolog	PPAN	1.18	0.98	1.15	1.26	0.67	0.74	1.07
Zinc finger CCCH domain-containing protein 15	ZC3H15	0.65*	0.74*	0.80*	0.74*	0.67*	0.67	0.80
Mitochondrial import inner membrane translocase subunit Tim8 A	TIMM8A	1.13	1.09	0.74*	1.13	0.67	0.86	0.84

D. Down-regulated proteins unique to Aquamin plus Mesalamine (AQ+MES) with LPS-Cytokines [90 proteins]

		Interventions						
		Control			*With LPS & Cytokines*			
Proteins	Genes	AQ	AQ+MES	MES	*LPS-Cyto*	*AQ*	***AQ+MES***	*MES*
Calmodulin-like protein 3	CALML3	1.02	52.36*	12.16*	0.75	1.15	0.24*	0.82
Loricrin	LORICRIN	0.48*	0.44*	0.64*	1.79*	1.63*	0.30*	1.10
Ferroxidase HEPHL1	HEPHL1	0.77	61.07*	3.28*	0.93	1.02	0.31*	1.91
Filaggrin-2	FLG2	0.24*	0.30*	0.44*	0.94	0.69*	0.34*	0.69*
Keratin, type II cuticular Hb2	KRT82	0.91	23.09*	1.49*	0.74	1.00	0.40*	1.04
Keratin, type II cytoskeletal 1	KRT1	0.42*	0.49*	0.57*	1.10	1.59*	0.44*	1.02
UPF0235 protein C15orf40	C15orf40	0.86	0.40*	0.33*	1.30	0.91	0.45*	0.67
Solute carrier family 13 member 2	SLC13A2	0.79	0.59*	0.49*	0.99	0.97	0.46*	0.81
Cornifin-B	SPRR1B	0.79	1.62*	1.68*	1.14	4.30*	0.46*	3.71*
Complement C1r subcomponent-like protein	C1RL	1.53*	1.26	1.35	1.05	1.08	0.47*	0.76
Keratin, type I cytoskeletal 27	KRT27	0.48*	0.64	0.52*	1.06	0.68	0.48*	1.03
Integrin alpha-7	ITGA7	0.48*	0.78	0.74	5.21*	0.84	0.49*	1.76
Keratin, type I cytoskeletal 9	KRT9	0.46*	0.44*	0.46*	1.11	1.99*	0.51*	0.89
Keratin, type II cytoskeletal 78	KRT78	0.71*	0.67*	1.26*	1.50*	1.92*	0.51*	1.49*
Kinesin-like protein KIF14	KIF14	1.08	1.04	1.02	1.08	1.33	0.51	0.80
Ubiquitin-conjugating enzyme E2 E1	UBE2E1	0.95	0.75	1.03	0.83	0.72	0.51	0.78
Transcription factor 20	TCF20	0.66*	0.99	1.00	2.22*	0.80	0.51	1.59
Early estrogen-induced gene 1 protein	EEIG1	0.91	0.93	1.05	0.79	0.78	0.52	0.90
RELT-like protein 1	RELL1	0.81	1.11	1.18	0.95	0.84	0.52	0.77
Trypsin-2	PRSS2	1.21	0.69	0.67*	1.28	0.72	0.53	0.68
Semenogelin-1	SEMG1	1.74*	4.10*	2.91*	0.71	2.56*	0.55*	2.67*

Keratin, type I cytoskeletal 17	KRT17	0.71*	0.64*	0.61*	1.72*	0.68	0.55*	0.77
Ubiquitin thioesterase OTU1	YOD1	1.22	1.23	2.20*	0.87	0.79	0.55	0.91
Zinc-alpha-2-glycoprotein	AZGP1	0.88	1.21	1.26	2.00*	1.12	0.56*	1.08
E3 ubiquitin-protein ligase MIB1	MIB1	0.62*	0.64*	0.55*	0.69	0.71	0.56	0.72
Lysosomal thioesterase PPT2	PPT2	0.78	1.58*	0.68	1.00	0.92	0.56	0.82
Plakophilin-1	PKP1	0.84	3.19*	2.07*	0.84	2.73*	0.56*	2.07*
Alpha-2-macroglobulin receptor-associated protein	LRPAP1	0.90	0.82	0.91	0.82	0.73	0.56*	0.67*
Peroxisomal ATPase PEX6	PEX6	0.87	0.82	0.92	0.98	0.72	0.57	0.76
UPF0538 protein C2orf76	C2orf76	0.62*	0.76	0.83	0.77	0.71	0.58	0.73
TBC1 domain family member 4	TBC1D4	0.72	0.82	0.99	0.92	0.82	0.58	0.85
Cytotoxic granule associated RNA binding protein TIA1	TIA1	0.87	0.80	0.88	0.99	0.75	0.60	0.74
3'-5' RNA helicase YTHDC2	YTHDC2	0.61*	0.59*	0.64*	0.82	0.72	0.60*	0.78
UPF0488 protein C8orf33	C8orf33	0.95	1.00	0.97	0.80	0.84	0.60*	0.68*
Deleted in malignant brain tumors 1 protein	DMBT1	2.24*	0.89	1.16*	1.67*	1.57*	0.60*	0.73*
Engulfment and cell motility protein 2	ELMO2	1.06	1.12	1.49	1.10	1.17	0.60	1.01
Arginase-1	ARG1	0.45*	0.50*	0.84	0.93	0.87	0.60	0.98
Large ribosomal subunit protein uL23m	MRPL23	0.93	0.93	1.09	0.89	0.76	0.61	0.73
SH3 domain-containing protein 21	SH3D21	1.28	1.49*	1.04	1.06	0.84	0.61	0.68
Elafin	PI3	1.00	0.70	1.30	0.83	0.80	0.61	0.77
Uridine-cytidine kinase-like 1	UCKL1	0.71	0.77	0.75	0.90	0.75	0.61	0.74
Probable rRNA-processing protein EBP2	EBNA1BP2	0.94	0.83	1.12	1.17	0.71	0.61*	0.96
Calmodulin-regulated spectrin-associated protein 1	CAMSAP1	0.59*	0.92	1.06	0.77	0.86	0.61	0.70
Synaptotagmin-like protein 2	SYTL2	0.92	0.83	0.86	0.89	0.71	0.62	0.79
Doublecortin domain-containing protein 1	DCDC1	0.92	0.96	0.81	0.79	0.75	0.62	1.03
WD repeat-containing protein 36	WDR36	0.60*	0.52*	0.56*	0.76	0.69	0.62	0.71
PRKC apoptosis WT1 regulator protein	PAWR	0.92	1.01	1.18	0.97	0.71	0.62	0.85
Ankyrin repeat and MYND domain-containing protein 2	ANKMY2	0.84	0.78	0.81	0.79	0.75	0.62	0.76
Methylthioribulose-1-phosphate dehydratase	APIP	1.24	0.94	1.17	0.99	0.71	0.62	0.97
Zinc finger CCCH domain-containing protein 13	ZC3H13	0.93	0.91	0.95	1.07	0.68	0.62	0.74
Pseudouridylate synthase RPUSD4, mitochondrial	RPUSD4	0.89	0.95	0.90	0.83	1.66	0.63	0.78
CD99 antigen	CD99	0.94	1.06	1.08	0.75	0.82	0.63	1.13
Tyrosine-protein kinase BAZ1B	BAZ1B	0.67*	0.66*	0.69*	0.85	0.68	0.63	0.72
Interleukin-18	IL18	0.96	0.82	0.87	0.80	0.75	0.63*	0.72*
Glia-derived nexin	SERPINE2	0.74*	0.75*	1.04	0.74*	0.67*	0.63*	0.68*
Monocyte differentiation antigen CD14	CD14	1.13	0.54*	1.02	0.95	1.09	0.63	0.72
Liprin-beta-2	PPFIBP2	0.82	0.79	0.86	0.80	0.74	0.63	0.73
Myosin light chain kinase, smooth muscle	MYLK	0.98	0.96	1.07	0.75	0.68	0.63	0.76

Serum response factor-binding protein 1	SRFBP1	1.01	1.10	1.12	1.54*	0.80	0.63	1.07
Xaa-Pro dipeptidase	PEPD	1.04	0.94	0.90	0.96	0.82	0.64	0.90
Phosphoribosylformylglycinamidine synthase	PFAS	0.86	0.97	0.85	1.16	0.82	0.64	1.06
Keratin, type I cytoskeletal 13	KRT13	0.75*	0.53*	0.92	0.69*	0.87	0.64	1.46*
Neuroblastoma suppressor of tumorigenicity 1	NBL1	1.17	0.99	0.92	0.90	0.90	0.64	0.87
Rho guanine nucleotide exchange factor 10-like protein	ARHGEF10L	0.96	0.79	0.85	0.76	0.73	0.64	0.79
Large ribosomal subunit protein eL39	RPL39	0.80	0.87	0.91	0.94	0.70	0.64	1.00
Nucleolar protein 16	NOP16	0.95	0.81	0.95	1.18	0.70	0.64	1.03
Nucleolar protein 6	NOL6	0.67*	0.57*	0.61*	0.81	0.75	0.64	0.74
cAMP-dependent protein kinase catalytic subunit beta	PRKACB	0.90	0.84	0.75	0.73	0.70	0.64	0.72
Syntaxin-binding protein 6	STXBP6	0.91	0.70	1.02	1.04	0.74	0.64	0.97
A-kinase anchor protein 7 isoform gamma	AKAP7	1.47	1.45	1.24	0.82	0.98	0.64	0.75
Tumor protein D53	TPD52L1	1.13	0.91	1.09	0.93	0.75	0.64	0.70
TSC22 domain family protein 4	TSC22D4	0.99	0.86	1.00	0.97	0.98	0.65	1.02
NAD-dependent protein deacetylase sirtuin-2	SIRT2	0.98	0.70	1.14	0.95	0.77	0.65	0.89
Claudin-1	CLDN1	0.52*	0.82	1.07	0.92	0.85	0.65	1.06
Ubiquitin-ribosomal protein eL40 fusion protein	UBA52	0.91	0.84	0.99	0.77	0.67	0.65	0.80
Ceramide-1-phosphate transfer protein	CPTP	0.90	0.78	0.78	0.83	0.81	0.65	0.83
Mesothelin	MSLN	0.98	0.86	1.13	0.86	0.77	0.65*	0.79
MutS protein homolog 5	MSH5	1.03	0.80	1.02	0.91	0.80	0.65	0.69
Nucleolar protein 14	NOP14	0.79*	0.81	1.18*	1.45*	0.75	0.65*	1.42*
Alcohol dehydrogenase 6	ADH6	1.07	0.66*	0.66*	0.79	0.97	0.65*	0.74
KxDL motif-containing protein 1	KXD1	0.83	0.78	0.80	0.89	0.92	0.66	0.85
Kalirin	KALRN	0.90	0.85	0.95	0.95	0.73	0.66	0.89
E3 ubiquitin-protein ligase RBBP6	RBBP6	1.01	0.94	1.17	0.97	0.70	0.66	1.02
Lysozyme C	LYZ	0.97	0.91	0.94	0.94	0.79	0.66*	0.92
Mitogen-activated protein kinase kinase kinase 4	MAP3K4	0.79	0.70	0.71	0.86	0.71	0.66	0.68
Coiled-coil domain-containing protein 9	CCDC9	1.01	1.07	0.94	1.18	0.77	0.66	0.69
DNA-directed RNA polymerase II subunit RPB9	POLR2I	0.70*	0.79	0.78	0.75	0.73	0.66	0.85
Cordon-bleu protein-like 1	COBLL1	0.84	0.83	0.95	0.76	0.73	0.66	0.86
Protein MANBAL	MANBAL	1.08	1.01	0.99	0.91	0.84	0.66	0.79
Xylulose kinase	XYLB	1.01	0.80	0.73*	0.98	0.76	0.66*	0.70*

E. Down-regulated proteins unique to Mesalamine (MES) with LPS-Cytokines [113 proteins]

		Interventions						
		Control			*With LPS & Cytokines*			

Proteins	Genes	AQ	AQ+MES	MES	*LPS-Cyto*	*AQ*	*AQ+MES*	***MES***
Calcium/calmodulin-dependent protein kinase type 1B	PNCK	1.12	0.39*	0.14*	1.00	1.60	0.82	0.13*
CD5 antigen-like	CD5L	0.66	1.53	23.17*	0.77	0.69	0.82	0.29*
StAR-related lipid transfer protein 7, mitochondrial	STARD7	0.92	1.44	1.26	0.95	0.85	1.26	0.30*
Profilin-3	PFN3	2.06*	0.44*	0.48*	0.82	0.81	0.84	0.37*
Ubiquinone biosynthesis protein COQ4 homolog, mitochondrial	COQ4	1.99*	1.66*	0.97	1.02	0.88	0.76	0.40*
Cytochrome P450 4F2	CYP4F2	1.28	1.00	0.89	0.76	0.96	0.81	0.43*
7-methylguanosine phosphate-specific 5'-nucleotidase	NT5C3B	1.45*	1.37	1.15	1.06	0.83	1.75	0.44*
Rho GTPase-activating protein 42	ARHGAP42	0.55*	0.74	0.71	0.78	0.67	0.97	0.45*
Cdc42 effector protein 5	CDC42EP5	0.44*	0.80	0.94	1.05	0.88	0.85	0.47*
Angiotensin-converting enzyme	ACE	1.37*	1.37*	0.72*	1.10	1.34*	0.70*	0.47*
Sodium-dependent neutral amino acid transporter B(0)AT1	SLC6A19	1.63*	2.12*	0.93	1.71*	1.74*	1.12	0.47*
Protein O-mannose kinase	POMK	1.35	1.28	1.31	0.78	0.83	0.81	0.48*
Dimethylaniline monooxygenase [N-oxide-forming] 4	FMO4	1.07	0.86	0.56*	1.06	0.72	0.70	0.48*
Serine/threonine-protein kinase 38-like	STK38L	1.41	0.92	0.85	1.13	0.74	1.02	0.49*
Intermembrane lipid transfer protein VPS13D	VPS13D	0.63*	0.69	0.51*	0.69	0.82	0.95	0.49*
Fibronectin type III and SPRY domain-containing protein 1	FSD1	3.62*	3.96*	1.02	0.70	2.52*	1.94*	0.49*
UDP-glucuronosyltransferase 2B7	UGT2B7	1.26	1.15	0.68*	0.75	1.37	0.97	0.50*
UDP-glucuronosyltransferase 2A3	UGT2A3	1.71*	1.27	0.93	0.94	1.03	0.73	0.51*
Tyrosine-protein kinase STYK1	STYK1	1.26	1.26	1.03	0.83	0.95	0.75	0.51*
DNA repair protein complementing XP-C cells	XPC	1.02	0.73	0.86	0.91	0.69	0.76	0.51*
Carcinoembryonic antigen-related cell adhesion molecule 7	CEACAM7	1.29*	0.98	0.79*	0.69*	0.82	0.91	0.52*
Syntabulin	SYBU	1.08	0.71	0.67	0.89	1.10	0.71	0.52
Bile salt export pump	ABCB11	1.44*	1.28	0.96	0.92	1.08	1.03	0.52
Serine/threonine-protein kinase ULK3	ULK3	0.96	1.00	1.00	0.78	0.74	0.67	0.52*
Protein AMN1 homolog	AMN1	0.83	0.93	0.95	0.67	0.92	0.82	0.53*
Phosphatidylserine decarboxylase proenzyme, mitochondrial	PISD	1.17	0.99	0.92	0.78	0.77	0.72	0.53*
GPI ethanolamine phosphate transferase 1	PIGN	1.17	1.12	1.08	0.80	0.72	0.74	0.53*
Sulfotransferase 1C2	SULT1C2	1.49*	0.71*	0.76	0.94	1.13	0.68	0.54*
NEDD4-like E3 ubiquitin-protein ligase WWP1	WWP1	0.82	0.76	0.69	0.90	0.98	0.69	0.54
Interleukin-1 receptor-associated kinase 4	IRAK4	0.94	0.92	0.91	0.80	0.74	0.75	0.54*
RAB6-interacting golgin	GORAB	0.98	0.96	1.26	0.80	0.99	1.05	0.55
Immunoglobulin lambda variable 1-51	IGLV1-51	0.85	1.31	8.76*	0.95	0.92	0.94	0.55*

Ephrin-A2	EFNA2	0.88	0.60*	0.64*	0.88	0.70	0.70	0.55*
Transmembrane 6 superfamily member 2	TM6SF2	1.03	0.80	0.84	0.74	0.89	1.02	0.56
Sorting nexin-30	SNX30	1.10	0.73	0.66*	0.80	0.73	0.69	0.56*
Kinase D-interacting substrate of 220 kDa	KIDINS220	0.66	0.81	0.67	0.81	0.76	0.85	0.57
Ribonuclease H2 subunit A	RNASEH2A	1.15	1.09	1.21	1.09	0.71	0.82	0.57
Sodium-coupled monocarboxylate transporter 2	SLC5A12	1.14	1.13	0.87	1.59*	1.39	0.68	0.57
Indian hedgehog protein	IHH	1.38*	1.29	0.84	0.97	1.24	0.92	0.57
Heat shock factor protein 1	HSF1	0.93	0.99	0.95	0.75	0.70	0.76	0.57*
Valacyclovir hydrolase	BPHL	1.04	1.03	0.86	0.69*	0.84	0.77	0.58*
Protein FAM98C	FAM98C	0.89	0.85	0.91	0.67	0.78	0.67	0.58
Immunoglobulin heavy variable 3-7	IGHV3-7	0.93	2.12*	23.53*	0.83	1.48	1.53	0.58
Oxysterol-binding protein-related protein 5	OSBPL5	0.87	0.71	0.76	0.68	0.98	1.08	0.58*
Calcium channel flower homolog	CACFD1	0.89	0.81	0.77	0.82	1.01	0.91	0.58
AKT-interacting protein	AKTIP	0.85	0.90	0.81	0.67*	0.75	0.73	0.58*
Creatine kinase B-type	CKB	1.86*	1.48*	1.47*	0.69*	0.99	1.05	0.58*
Bridge-like lipid transfer protein family member 1	BLTP1	0.98	0.65	0.59*	0.88	1.01	0.80	0.58
Peroxisomal bifunctional enzyme	EHHADH	1.10	1.00	0.90	0.70*	0.71	0.74	0.59*
Inhibitor of growth protein 1	ING1	1.02	0.47*	0.62*	0.86	1.16	0.73	0.59
Patatin-like phospholipase domain-containing protein 2	PNPLA2	1.07	1.23	0.89	0.71	0.90	0.92	0.59*
Transcription cofactor vestigial-like protein 4	VGLL4	0.92	1.59*	1.92*	0.98	0.94	0.98	0.60
Mitochondrial peptide methionine sulfoxide reductase	MSRA	1.01	0.90	0.78	0.73	0.88	0.69	0.60
Retinol dehydrogenase 10	RDH10	1.07	0.96	0.96	0.67	0.90	0.68	0.61
Guanylyl cyclase C	GUCY2C	1.25	1.13	1.00	0.75	0.80	0.74	0.61
Serine hydrolase RBBP9	RBBP9	1.10	0.93	0.99	1.02	0.85	0.76	0.61
Myelin regulatory factor-like protein	MYRFL	0.96	0.80	0.57*	1.19	0.89	0.77	0.61
Cadherin-related family member 2	CDHR2	1.07	1.07	0.89	0.79	0.96	0.68	0.61*
Keratinocyte differentiation factor 1	KDF1	0.96	1.14	1.14	0.92	0.82	0.74	0.61
Epidermal retinol dehydrogenase 2	SDR16C5	1.03	1.10	0.91	0.76	0.82	0.71	0.61*
Mitochondrial Rho GTPase 1	RHOT1	0.78	1.00	1.00	0.87	0.83	0.92	0.61
Alanine aminotransferase 1	GPT	1.14	1.16	0.80	0.68*	0.84	0.80	0.62*
Polyprenol reductase	SRD5A3	1.02	0.92	0.90	0.68	0.89	0.78	0.62
A disintegrin and metalloproteinase with thrombospondin motifs 2	ADAMTS2	1.04	0.96	0.98	0.90	0.79	0.82	0.62
Cytochrome P450 2C19	CYP2C19	2.12*	1.15	0.61*	1.64*	1.98*	0.88	0.62
Transmembrane protein 54	TMEM54	1.23	1.25	1.27	0.97	0.70	0.74	0.62
Protein asteroid homolog 1	ASTE1	0.94	1.10	1.01	0.75	1.22	1.40	0.62
Sarcoplasmic/endoplasmic reticulum calcium ATPase 3	ATP2A3	1.17	0.63*	0.89	0.69	0.72	0.68	0.62

Interleukin-1 receptor-associated kinase 1	IRAK1	0.88	0.82	0.84	0.75	0.74	0.78	0.62
SEC14 domain and spectrin repeat-containing protein 1	SESTD1	0.79	0.84	0.82	0.77	0.84	0.68	0.63
All-trans-retinol 13,14-reductase	RETSAT	1.09	0.92	0.82*	0.79*	0.98	0.76	0.63*
Tuftelin	TUFT1	0.84	0.99	1.01	0.78	0.97	0.67	0.63
Gap junction beta-3 protein	GJB3	0.93	0.70	0.74	0.68	1.01	0.70	0.63
Sorting nexin-13	SNX13	1.11	0.85	0.80	0.69	0.75	0.99	0.63
Protein angel homolog 2	ANGEL2	0.98	0.89	0.54*	0.99	0.91	0.95	0.63
UDP-N-acetylglucosamine transferase subunit ALG14 homolog	ALG14	0.63	0.81	1.10	1.15	1.16	0.88	0.64
DNA polymerase epsilon subunit 3	POLE3	1.42*	1.44	1.47	1.76*	1.10	0.98	0.64
Transmembrane protein 82	TMEM82	1.16	1.11	0.78	0.86	0.98	0.95	0.64
Rab GTPase-activating protein 1-like	HHL	0.91	0.86	1.11	0.77	0.78	0.89	0.64*
Chromobox protein homolog 1	CBX1	0.86	1.05	0.93	0.94	0.76	0.73	0.64*
Fatty acid-binding protein, liver	FABP1	1.10	0.92	0.87	0.78*	0.83	0.71*	0.65*
Acidic fibroblast growth factor intracellular-binding protein	FIBP	0.73*	0.79	0.73*	0.74	0.80	0.76	0.65*
Receptor tyrosine-protein kinase erbB-2	ERBB2	0.79	0.87	0.96	1.03	0.85	0.87	0.65
EGF domain-specific O-linked N-acetylglucosamine transferase	EOGT	0.96	0.86	0.72	0.85	0.81	1.00	0.65
Protein N-terminal asparagine amidohydrolase	NTAN1	1.23	0.94	1.05	0.94	0.70	0.82	0.65
Peroxiredoxin-6	PRDX6	1.15	1.16	1.01	0.75*	0.82	0.86	0.65*
Coenzyme Q-binding protein COQ10 homolog B, mitochondrial	COQ10B	0.88	1.05	0.84	0.80	0.74	0.78	0.65
Amiloride-sensitive sodium channel subunit alpha	SCNN1A	1.60*	1.38	0.85	0.99	0.86	1.44	0.65
tRNA modification GTPase GTPBP3, mitochondrial	GTPBP3	0.71	1.16	1.14	1.12	1.53	1.55	0.65
Fatty acid desaturase 6	FADS6	1.32*	1.26	0.88	0.94	1.21	1.03	0.65
SH2 domain-containing protein 4A	SH2D4A	0.91	0.83	0.99	0.97	0.87	0.86	0.65
Protein unc-13 homolog B	UNC13B	0.77	0.75	0.94	0.80	0.69	0.74	0.65
Phospholipid-transporting ATPase IA	ATP8A1	1.05	1.10	0.94	0.73*	0.82	0.84	0.65*
Long-chain fatty acid transport protein 1	SLC27A1	1.02	1.10	0.96	0.81	1.10	1.02	0.65
Transmembrane protein 263	TMEM263	0.67*	0.91	0.93	0.67*	0.71	0.72	0.65*
Acid sphingomyelinase-like phosphodiesterase 3a	SMPDL3A	0.99	0.75	0.81	0.81	0.76	0.76	0.66*
Group XIIB secretory phospholipase A2-like protein	PLA2G12B	1.01	1.25	0.97	0.86	0.74	1.13	0.66
Mediator of RNA polymerase II transcription subunit 25	MED25	0.80	0.95	0.70	0.87	0.75	0.84	0.66
ATP-dependent RNA helicase DQX1	DQX1	1.09	0.84	0.71	1.10	0.78	0.90	0.66
ATP-binding cassette sub-family D member 3	ABCD3	1.04	1.08	0.96	0.80*	0.84	0.93	0.66*
1-acylglycerol-3-phosphate O-acyltransferase ABHD5	ABHD5	0.95	0.84	0.89	0.94	1.04	0.76	0.66
Ribosomal protein S6 kinase beta-1	RPS6KB1	0.98	1.31	1.08	0.88	0.77	0.76	0.66

Cytoplasmic phosphatidylinositol transfer protein 1	PITPNC1	0.99	0.98	0.88	1.09	0.69	0.71	0.66
Aldehyde dehydrogenase 1A1	ALDH1A1	1.18	0.95	0.87	0.73*	0.91	0.68*	0.66*
Breakpoint cluster region protein	BCR	0.83	0.60*	0.73	0.72	0.82	0.81	0.66
Transmembrane protein 256	TMEM256	0.85	0.72	0.87	0.82	0.85	0.91	0.66
Zinc transporter ZIP4	SLC39A4	1.32*	1.15	0.98	0.85	0.94	1.15	0.66
Zinc finger FYVE domain-containing protein 1	ZFYVE1	1.06	0.74	0.59*	0.76	0.82	0.87	0.67
Eukaryotic translation initiation factor 4E type 3	EIF4E3	1.03	0.83	0.75	1.11	1.08	0.96	0.67
Dual specificity mitogen-activated protein kinase kinase 6	MAP2K6	1.09	1.18	0.99	0.76	0.72	0.69	0.67*
Long-chain-fatty-acid--CoA ligase 1	ACSL1	1.16	0.88	0.84	0.79	0.91	0.73	0.67*
NF-kappa-B inhibitor alpha	NFKBIA	1.02	1.07	1.14	0.89	0.91	1.27	0.67
Transmembrane protein 35B	TMEM35B	0.99	1.13	0.70	1.06	0.86	0.89	0.67

F. Common down-regulated proteins between LPS-Cytokines alone and with Aquamin [22 proteins]

		Interventions						
		Control			*With LPS & Cytokines*			
Proteins	Genes	AQ	AQ+MES	MES	***LPS-Cyto***	***AQ***	*AQ+MES*	*MES*
Rho guanine nucleotide exchange factor 40	ARHGEF40	0.35*	0.74	1.05	0.31*	0.49*	0.88	0.97
Tetratricopeptide repeat protein 7A	TTC7A	0.60*	0.57*	0.61*	0.46*	0.66	0.77	0.73
Cytosolic iron-sulfur assembly component 2B	CIAO2B	0.45*	0.60*	1.29	0.47*	0.38*	1.57	1.87
Rapamycin-insensitive companion of mTOR	RICTOR	0.52*	0.69	0.57*	0.47*	0.57	0.72	0.69
Inter-alpha-trypsin inhibitor heavy chain H1	ITIH1	0.48*	0.96	1.54*	0.48*	0.48*	1.13	1.53
Prolyl 3-hydroxylase 3	P3H3	1.07	1.08	1.14	0.50*	0.61	0.82	0.93
Probable phosphoglycerate mutase 4	PGAM4	0.53*	1.15	2.54*	0.51*	0.63	1.94*	2.77*
Ubiquitin carboxyl-terminal hydrolase 48	USP48	0.66*	0.45*	0.72	0.53*	0.62	0.70	0.74
Coiled-coil domain-containing protein 9B	CCDC9B	0.53*	0.96	1.03	0.55*	0.41*	0.73	0.79
Uncharacterized protein FLJ45252		0.82	0.79	1.02	0.58*	0.64	0.69	0.76
Serine/threonine-protein kinase A-Raf	ARAF	0.87	0.65*	0.81	0.60*	0.66	0.73	0.87
Chromodomain-helicase-DNA-binding protein 1-like	CHD1L	0.79	0.73	0.72	0.60*	0.67	0.84	0.76
AP-5 complex subunit beta-1	AP5B1	0.82	0.77	0.66*	0.61*	0.56	0.72	0.69
Low-density lipoprotein receptor-related protein 5	LRP5	0.62*	1.06	0.81	0.61*	0.55	0.72	0.76
Poly(A)-specific ribonuclease PARN	PARN	0.59*	0.68*	0.67*	0.62*	0.66*	0.71	0.71
Pachytene checkpoint protein 2 homolog	TRIP13	0.77*	0.84	0.92	0.64*	0.65	0.81	0.73
Protein FAM107B	FAM107B	0.81	0.92	0.67*	0.64	0.61	0.69	0.69
Transducin beta-like protein 3	TBL3	0.43*	0.52*	0.55*	0.64*	0.60	0.68	0.74
AMP deaminase 3	AMPD3	0.58*	0.83	0.81	0.65	0.64	0.73	0.77
ADP-ribosylation factor-like protein 2	ARL2	0.75	0.69	0.75	0.66	0.60	0.68	0.69

Guanidinoacetate N-methyltransferase	GAMT	0.55*	1.98*	1.89*	0.67	0.57	1.54	1.94*
GRB10-interacting GYF protein 2	GIGYF2	0.85	0.78	0.97	0.67*	0.55*	0.67	0.83

G. Common down-regulated proteins between LPS-Cytokines alone and with Aquamin plus Mesalamine [21 proteins]

		Interventions						
		Control			*With LPS & Cytokines*			
Proteins	Genes	AQ	AQ+MES	MES	***LPS-Cyto***	*AQ*	***AQ+MES***	*MES*
Cap-specific mRNA (nucleoside-2'-O-)-methyltransferase 2	CMTR2	0.25*	0.45*	0.57*	0.26*	0.82	0.62	0.87
Vacuolar protein sorting-associated protein 72 homolog	VPS72	0.62*	0.73	0.82	0.28*	0.92	0.17*	0.70
Histidine ammonia-lyase	HAL	1.60*	2.11*	5.94*	0.35*	2.50*	0.56	2.12*
Retroviral-like aspartic protease 1	ASPRV1	0.92	3.16*	10.29*	0.39*	1.54*	0.49*	1.97*
Keratin, type I cytoskeletal 25	KRT25	0.44*	1.22	0.25*	0.41*	1.70*	0.23*	1.17
Alpha-2-macroglobulin-like protein 1	A2ML1	1.27*	2.46*	5.56*	0.41*	1.85*	0.34*	1.36*
Cornulin	CRNN	1.73*	2.38*	5.70*	0.44*	3.63*	0.61	2.57*
Cystatin-M	CST6	1.81*	3.67*	3.47*	0.54*	1.96*	0.58	1.84*
Mismatch repair endonuclease PMS2	PMS2	0.47*	0.47*	0.58*	0.54*	1.06	0.65	0.77
Myeloblastin	PRTN3	0.84	3.59*	2.95*	0.55*	1.93*	0.45*	1.61
Histone deacetylase complex subunit SAP30	SAP30	0.72	0.63*	0.71	0.57*	0.95	0.62	0.72
Protein LRATD1	LRATD1	0.99	0.73	0.82	0.58*	0.76	0.67	0.70
DNA-directed RNA polymerase I subunit RPA1	POLR1A	0.74*	0.60*	0.57*	0.61*	0.73	0.64	0.71
Proline-rich protein 15	PRR15	0.69	0.84	1.11	0.61*	0.69	0.62	0.77
Protein KPLCE	KPLCE	1.16	0.88	0.98	0.61*	3.48*	0.40*	1.62*
Protein arginine N-methyltransferase 3	PRMT3	0.53*	0.56*	0.71*	0.61*	0.69	0.66	0.73
Helicase SRCAP	SRCAP	0.47*	0.38*	0.76	0.62	0.78	0.59	0.85
Ribonuclease 7	RNASE7	0.79	0.80	0.67*	0.64*	1.10	0.43*	1.35
Mammaglobin-B	SCGB2A1	1.32	5.33*	2.76*	0.64	1.80*	0.64	1.75
Uncharacterized protein KIAA1143	KIAA1143	1.23	0.88	1.10	0.64*	0.71	0.43*	0.72
Protein-glutamine gamma-glutamyltransferase K	TGM1	1.13	1.39	1.71*	0.66	3.12*	0.49*	2.44*

H. Common down-regulated proteins between LPS-Cytokines alone and with Mesalamine [32 proteins]

		Interventions						
		Control			*With LPS & Cytokines*			
Proteins	Genes	AQ	AQ+MES	MES	***LPS-Cyto***	*AQ*	*AQ+MES*	***MES***
TBC1 domain family member 2B	TBC1D2B	0.37*	0.52*	0.55*	0.35*	0.89	0.73	0.60
Ubiquitin carboxyl-terminal hydrolase 27	USP27X	0.39*	0.49*	0.71*	0.43*	0.82	0.81	0.63

E3 ubiquitin-protein transferase MAEA	MAEA	0.36*	0.45*	0.47*	0.45*	0.72	0.67	0.61*
Condensin complex subunit 3	NCAPG	0.58*	0.53*	0.54*	0.45*	0.97	0.80	0.67
WD repeat-containing protein 37	WDR37	0.44*	0.58*	0.50*	0.48*	0.72	0.69	0.64
General transcription factor 3C polypeptide 1	GTF3C1	0.42*	0.44*	0.37*	0.48*	0.68	0.68	0.43*
Vacuolar protein sorting-associated protein 18 homolog	VPS18	0.56*	0.56*	0.55*	0.49*	0.67*	0.70	0.59*
AP-4 complex subunit beta-1	AP4B1	0.37*	0.55*	0.63*	0.50*	0.67	0.68	0.55
Pleckstrin homology domain-containing family A member 2	PLEKHA2	0.73	0.77	0.79	0.50*	0.71	0.87	0.39*
Serine incorporator 2	SERINC2	0.90	0.86	0.58*	0.50*	0.89	0.86	0.55*
Prostate stem cell antigen	PSCA	1.17	0.85	1.13	0.51*	0.78	0.87	0.67
Transmembrane protein 225B	TMEM225B	0.44*	0.39*	0.80	0.51*	0.70	1.23	0.59
Pleckstrin homology domain-containing family S member 1	PLEKHS1	0.77	1.46	1.10	0.52*	0.69	0.70	0.17*
Glucocorticoid modulatory element-binding protein 2	GMEB2	0.60*	0.73	0.76	0.54*	0.68	0.74	0.57
Trafficking protein particle complex subunit 10	TRAPPC10	0.69*	0.66*	0.63*	0.55*	0.71	0.72	0.54*
Tripartite motif-containing protein 5	TRIM5	0.65*	0.68	0.64*	0.55*	0.75	0.87	0.57
CNK3/IPCEF1 fusion protein	CNK3/IPCEF1	1.00	1.11	0.82	0.56*	0.99	1.03	0.64
Nuclear factor 1 A-type	NFIA	0.51*	0.65*	0.67*	0.57*	0.73	0.68	0.60*
Flavin-containing monooxygenase 5	FMO5	1.01	0.96	0.80	0.57*	0.78	0.91	0.58*
Protein pelota homolog	PELO	0.60*	0.72*	0.69*	0.59*	0.70	0.70	0.65*
F-box only protein 38	FBXO38	0.77	0.74	0.67*	0.60*	0.72	0.72	0.56*
DNA mismatch repair protein Msh6	MSH6	0.85	0.62*	0.61*	0.60*	0.81	0.76	0.63
Serine/threonine-protein kinase 17B	STK17B	1.11	1.30	0.93	0.61*	0.76	0.73	0.48*
Peptide chain release factor 1, mitochondrial	MTRF1	1.00	1.14	1.03	0.62*	0.95	1.03	0.40*
Unconventional myosin-Vc	MYO5C	0.66	0.63*	0.70	0.62*	0.67	0.78	0.66
17-beta-hydroxysteroid dehydrogenase type 2	HSD17B2	1.40*	1.31*	0.98	0.63*	0.70*	0.67*	0.42*
Transmembrane 4 L6 family member 20	TM4SF20	1.61*	0.93	0.65*	0.63*	1.51*	0.97	0.50*
Tripartite motif-containing protein 3	TRIM3	0.85	0.53*	0.45*	0.64*	0.79	0.92	0.52
Ras-related protein Rab-3B	RAB3B	0.90	0.94	1.15	0.64*	0.81	0.78	0.61
Desmocollin-3	DSC3	1.72*	9.40*	34.18*	0.65	1.84*	0.93	0.01*
EEF1A lysine methyltransferase 1	EEF1AKMT1	0.97	1.13	1.04	0.65*	0.75	0.70	0.63
Calpain-7	CAPN7	0.75	0.70	0.69*	0.66*	0.76	0.71	0.61

I. Common down-regulated proteins among LPS-Cytokines alone, with Aquamin and with Aquamin plus Mesalamine [23 proteins]

		Interventions						
		Control			*With LPS & Cytokines*			
Proteins	Genes	AQ	AQ+MES	MES	***LPS-Cyto***	***AQ***	***AQ+MES***	*MES*

PHD finger protein 6	PHF6	0.32*	0.43*	0.46*	0.26*	0.65*	0.64*	0.69*
AN1-type zinc finger protein 1	ZFAND1	0.53*	0.50*	0.58*	0.40*	0.41*	0.34*	0.71
Splicing factor, suppressor of white-apricot homolog	SFSWAP	0.77	0.87	0.71*	0.42*	0.48*	0.56	0.78
ATP-dependent RNA helicase DDX39A	DDX39A	0.72	0.78	0.83	0.48*	0.52*	0.64	0.68
tRNA (guanine(26)-N(2))-dimethyltransferase	TRMT1	0.64*	0.82	0.82	0.50*	0.50*	0.39*	0.72
Synaptopodin	SYNPO	0.94	1.12	1.25	0.56*	0.48*	0.64	0.88
Ubiquitin carboxyl-terminal hydrolase 16	USP16	0.76*	0.88	0.87	0.56*	0.59*	0.54*	0.80
FAST kinase domain-containing protein 1, mitochondrial	FASTKD1	0.94	0.96	0.93	0.58*	0.31*	0.45*	0.82
Refilin-A	RFLNA	1.15	1.15	1.16	0.58*	0.61	0.65	0.94
Prothymosin alpha	PTMA	0.77	0.65*	1.07	0.59*	0.45*	0.66	1.38*
Ribonucleoprotein PTB-binding 2	RAVER2	0.86	1.47	1.25	0.59*	0.57	0.57	1.71
POTE ankyrin domain family member E	POTEE	1.02	0.49*	0.95	0.59*	0.50*	0.51*	0.90
Atypical kinase COQ8B, mitochondrial	COQ8B	0.71	0.60*	0.66*	0.60*	0.50*	0.61	0.72
Protein FAM117B	FAM117B	0.47*	0.87	0.70	0.60*	0.12*	0.28*	0.71
DNA mismatch repair protein Msh3	MSH3	0.66*	0.67*	0.70*	0.60*	0.66	0.65	0.69
Hyccin 2	HYCC2	0.90	0.89	0.69	0.60*	0.58	0.62	0.71
FH1/FH2 domain-containing protein 1	FHOD1	0.77	0.69	0.76	0.61*	0.59	0.63	0.67
Secretion-regulating guanine nucleotide exchange factor	SERGEF	0.98	1.22	1.06	0.62*	0.61	0.61	0.82
Overexpressed in colon carcinoma 1 protein	OCC1	0.65*	0.70	0.87	0.63*	0.55*	0.62	0.87
Cytosolic carboxypeptidase 1	AGTPBP1	0.58*	0.73	0.86	0.64*	0.62	0.35*	0.67
Serine/threonine-protein kinase LMTK2	LMTK2	0.80	0.95	0.98	0.65*	0.60	0.63	0.68
Protein Tob2	TOB2	0.73	0.84	0.54*	0.66	0.35*	0.37*	0.95
ATP-dependent RNA helicase DDX51	DDX51	0.64*	0.66*	0.72*	0.66*	0.55*	0.60	0.70

J. Common down-regulated proteins among LPS-Cytokines alone, with Aquamin and with Mesalamine [25 proteins]

		Interventions						
		Control			*With LPS & Cytokines*			
Proteins	Genes	AQ	AQ+MES	MES	***LPS-Cyto***	***AQ***	*AQ+MES*	***MES***
Dynein axonemal heavy chain 8	DNAH8	0.65*	0.96	0.58*	0.19*	0.36*	2.06*	0.37*
Replication factor C subunit 5	RFC5	0.51*	0.49*	0.62*	0.29*	0.55*	0.70	0.43*
Tumor necrosis factor receptor superfamily member 10D	TNFRSF10D	0.96	1.40*	1.27	0.41*	0.56*	1.02	0.45*
Structural maintenance of chromosomes protein 2	SMC2	0.55*	0.44*	0.54*	0.42*	0.65	0.74	0.48*
Heat shock protein beta-1	HSPB1	1.21*	1.07	0.98	0.50*	0.60*	0.67*	0.56*
Deoxynucleotidyltransferase terminal-interacting protein 2	DNTTIP2	0.57*	0.77	0.76	0.51*	0.67	0.67	0.56*

Helicase with zinc finger domain 2	HELZ2	0.61*	0.88	0.77	0.53*	0.36*	0.97	0.57
Conserved oligomeric Golgi complex subunit 6	COG6	0.87	1.08	0.80	0.55*	0.59	0.70	0.45*
Membrane-bound transcription factor site-1 protease	MBTPS1	0.93	0.92	1.00	0.56*	0.56	0.69	0.60
SUN domain-containing protein 1	SUN1	0.53*	0.64*	0.58*	0.57*	0.65	0.69	0.49*
NTF2-related export protein 2	NXT2	1.07	1.05	0.93	0.57*	0.44*	0.78	0.66
Trinucleotide repeat-containing gene 6B protein	TNRC6B	1.51*	1.07	0.90	0.59*	0.65	1.27	0.62
Zinc finger FYVE domain-containing protein 16	ZFYVE16	0.52*	0.76	0.62*	0.59*	0.40*	0.71	0.60
Phosphomannomutase 1	PMM1	0.91	1.20	1.12	0.61*	0.65	0.73	0.49*
TBC1 domain family member 8B	TBC1D8B	0.65*	0.82	0.74*	0.61*	0.63	0.67	0.63
Protein kish-A	TMEM167A	0.87	0.92	0.94	0.62*	0.60*	1.03	0.64
RNA-binding protein 12B	RBM12B	0.80	0.82	0.78	0.63	0.58	0.68	0.58
Complement C1r subcomponent	C1R	0.94	1.31	14.51*	0.63	0.54	1.27	0.15*
Threonine synthase-like 1	THNSL1	0.88	1.16	1.27	0.63*	0.62	0.71	0.66
Sulfate transporter	SLC26A2	2.55*	1.80*	0.90	0.65*	0.48*	0.77	0.44*
Proteasome subunit beta type-5	PSMB5	0.90	0.81	0.84	0.65*	0.60	0.71	0.57*
Probable proline--tRNA ligase, mitochondrial	PARS2	0.84	0.87	0.88	0.65*	0.55*	0.68	0.51*
Telomerase-binding protein EST1A	SMG6	0.65*	0.61*	0.87	0.65	0.60	0.67	0.66
Zinc finger protein 718	ZNF718	0.75	0.71	0.69*	0.65*	0.60	0.70	0.63
Ras and Rab interactor 2	RIN2	0.79	0.80	0.71	0.66	0.56	0.72	0.55

K. Common down-regulated proteins among LPS-Cytokines alone, with Aquamin plus Mesalamine and with Mesalamine [40 proteins]

		Interventions						
		Control			*With LPS & Cytokines*			
Proteins	Genes	AQ	AQ+MES	MES	***LPS-Cyto***	*AQ*	***AQ+MES***	***MES***
Primary cilium assembly protein FAM149B1	FAM149B1	0.29*	0.24*	0.24*	0.30*	0.85	0.39*	0.60
Putative ATP-dependent RNA helicase DHX57	DHX57	0.45*	0.47*	0.46*	0.30*	0.71	0.63	0.46*
SWI/SNF-related matrix-associated actin-dependent regulator of chromatin subfamily A-like protein 1	SMARCAL1	0.57*	0.55*	0.67*	0.31*	0.77	0.53*	0.44*
Myotubularin-related protein 13	SBF2	0.39*	0.59*	0.60*	0.32*	0.67	0.42*	0.40*
E3 ubiquitin-protein ligase NRDP1	RNF41	0.43*	0.55*	0.45*	0.33*	0.81	0.61	0.52
ADP-ribosylation factor-binding protein GGA2	GGA2	0.61*	0.80	0.81	0.40*	0.69	0.44*	0.48*
Mitogen-activated protein kinase kinase kinase 20	MAP3K20	0.44*	0.46*	0.61*	0.40*	0.79	0.65	0.61*
Monocarboxylate transporter 6	SLC16A5	0.77	0.92	0.78	0.43*	0.68	0.55	0.46*
Tensin-3	TNS3	0.53*	0.80	0.74*	0.43*	0.72	0.61	0.47*
Tensin-4	TNS4	0.30*	0.47*	0.46*	0.43*	0.67	0.52*	0.45*
Serine/threonine-protein phosphatase 6 regulatory ankyrin repeat subunit A	ANKRD28	0.46*	0.44*	0.46*	0.44*	0.67	0.55	0.51*
Mitogen-activated protein kinase 7	MAPK7	0.47*	0.62*	0.67*	0.45*	0.68	0.63	0.63

Arylamine N-acetyltransferase 1	NAT1	0.88	0.42*	0.46*	0.46*	0.77	0.60	0.62
Serine/threonine-protein phosphatase 6 regulatory ankyrin repeat subunit C	ANKRD52	0.54*	0.63	0.71	0.49*	0.75	0.59	0.49*
Probable JmjC domain-containing histone demethylation protein 2C	JMJD1C	0.47*	0.42*	0.33*	0.50*	0.72	0.42*	0.29*
Meprin A subunit alpha	MEP1A	1.35*	1.47*	0.85	0.50*	0.69*	0.57*	0.47*
Atypical kinase COQ8A, mitochondrial	COQ8A	0.82	1.19	1.04	0.52*	0.72	0.62	0.45*
Leucine-rich alpha-2-glycoprotein	LRG1	1.03	0.46*	0.83	0.52*	0.97	0.66	0.54*
GTP-binding protein 10	GTPBP10	1.01	0.90	0.62*	0.52*	0.78	0.66*	0.52*
Ubiquitin carboxyl-terminal hydrolase 3	USP3	0.63*	0.71	0.70	0.52*	0.67	0.41*	0.28*
Smad nuclear-interacting protein 1	SNIP1	0.49*	0.69	0.71	0.53*	0.69	0.66	0.48*
Cleavage and polyadenylation specificity factor subunit 4	CPSF4	0.61*	0.69*	0.66*	0.55*	0.69	0.65	0.61*
Intelectin-2	ITLN2	1.04	1.01	0.45*	0.56*	1.13	0.38*	0.42*
Protein-arginine deiminase type-2	PADI2	1.11	1.05	0.91	0.57*	0.73	0.64*	0.53*
DDB1- and CUL4-associated factor 13	DCAF13	0.82*	0.72*	0.75*	0.58*	0.70	0.63*	0.65*
RNA-binding protein 6	RBM6	0.84	0.77	0.79	0.58*	0.73	0.64	0.51*
Pleckstrin homology domain-containing family A member 4	PLEKHA4	1.12	0.99	1.45	0.58*	0.71	0.54	0.62
Cytosolic iron-sulfur assembly component 3	CIAO3	0.48*	0.47*	0.55*	0.58*	0.69	0.48*	0.58*
Cytochrome c oxidase assembly factor 4 homolog, mitochondrial	COA4	1.02	1.08	0.99	0.59*	0.81	0.61	0.63
Annexin A13	ANXA13	1.19*	0.98	0.76*	0.60*	0.83	0.55*	0.39*
Hydroxymethylglutaryl-CoA synthase, mitochondrial	HMGCS2	1.33*	1.13	0.88	0.61*	0.73*	0.66*	0.55*
Sulfotransferase 1B1	SULT1B1	1.02	0.90	0.84	0.61*	0.67	0.54*	0.47*
5'-AMP-activated protein kinase subunit gamma-2	PRKAG2	0.84	1.01	0.80	0.62*	0.81	0.57	0.48*
Mucosa-associated lymphoid tissue lymphoma translocation protein 1	MALT1	0.55*	0.63	0.79	0.62	0.67	0.61	0.58
Poly(rC)-binding protein 3	PCBP3	1.00	0.97	0.95	0.63	0.72	0.66	0.58
Transferrin receptor protein 1	TFRC	1.04	0.83	0.67*	0.63*	0.93	0.58*	0.52*
Phosphoinositide 3-kinase regulatory subunit 4	PIK3R4	0.61*	0.65*	0.59*	0.64*	0.72	0.62	0.58
Carbonic anhydrase 1	CA1	0.97	0.86	0.67*	0.66*	0.70	0.66	0.66*
Lactase/phlorizin hydrolase	LCT	0.79	0.74	1.16	0.66	0.69	0.48*	0.39*
Steroid hormone receptor ERR1	ESRRA	0.67*	0.77	0.76	0.66	0.67	0.61	0.61

L. Common down-regulated proteins between LPS-Cytokines with Aquamin and with Aquamin plus Mesalamine [123 proteins]

		Interventions						
		Control			*With LPS & Cytokines*			
Proteins	Genes	AQ	AQ+MES	MES	*LPS-Cyto*	***AQ***	***AQ+MES***	*MES*

Nuclear speckle splicing regulatory protein 1	NSRP1	1.69*	2.06*	2.33*	1.09	0.02*	0.40*	1.14
Nucleolar protein 7	NOL7	1.42*	1.20	1.38*	1.46*	0.21*	0.42*	0.68
Fos-related antigen 1	FOSL1	0.58*	0.55*	0.85	0.79	0.21*	0.42*	0.78
Protein max	MAX	1.28*	1.31	1.42*	1.85*	0.24*	0.39*	0.75
Vesicle transport protein SFT2C	SFT2D3	0.91	1.08	0.75*	1.19	0.25*	0.28*	0.85
Transmembrane protein 51	TMEM51	1.19	1.38	1.70*	1.29	0.27*	0.66	0.80
Phosphorylated adapter RNA export protein	PHAX	1.12	0.93	1.21	0.85	0.29*	0.50	0.80
Protein Dr1	DR1	1.21	1.20	1.30	1.10	0.29*	0.62	0.80
RNA exonuclease 4	REXO4	1.08	0.91	1.02	1.10	0.29*	0.36*	0.80
Zinc finger CCCH domain-containing protein 4	ZC3H4	1.14	1.02	1.15	1.18	0.29*	0.57	0.81
U6 snRNA-associated Sm-like protein LSm5	LSM5	1.17	1.00	1.01	1.07	0.30*	0.48*	0.68
Microfibrillar-associated protein 1	MFAP1	1.11	0.96	1.09	1.09	0.31*	0.38*	0.68*
Translation machinery-associated protein 16	TMA16	0.81	0.94	1.04	1.01	0.34*	0.54*	0.83
Protein ELYS	AHCTF1	0.73*	0.88	0.87	0.95	0.35*	0.29*	0.76
Extracellular sulfatase Sulf-2	SULF2	0.59*	0.93	1.25*	0.86	0.35*	0.37*	0.73*
Mediator of DNA damage checkpoint protein 1	MDC1	0.91	0.65	0.62*	0.70	0.35*	0.50*	1.13
Zinc finger protein ubi-d4	DPF2	0.75	0.98	0.71	0.73	0.35*	0.50*	0.81
KRR1 small subunit processome component homolog	KRR1	1.24*	1.05	1.20*	0.95	0.35*	0.43*	0.82
U3 small nucleolar RNA-associated protein 14 homolog A	UTP14A	0.74	0.96	1.11	1.00	0.35*	0.52*	0.74
Nuclear receptor subfamily 2 group F member 6	NR2F6	0.53*	0.55*	0.85	1.03	0.36*	0.53	0.69
KICSTOR subunit 2	KICS2	0.48*	0.38*	1.09	1.29	0.39*	0.38*	0.78
U4/U6.U5 small nuclear ribonucleoprotein 27 kDa protein	SNRNP27	1.30	0.82	1.06	1.32	0.39*	0.59	0.89
Nucleolar complex protein 3 homolog	NOC3L	0.55*	0.80	0.98	1.23	0.41*	0.42*	0.82
Mth938 domain-containing protein	AAMDC	0.97	1.03	0.99	0.89	0.42*	0.55*	0.67*
YLP motif-containing protein 1	YLPM1	0.84	0.74	0.93	0.85	0.42*	0.46*	0.75
Thioredoxin-like protein 4A	TXNL4A	1.23*	1.22	1.01	1.41*	0.42*	0.56*	0.75
Protein FRG1	FRG1	0.78	0.72	0.85	0.69	0.43*	0.44*	0.68
Lysine-specific demethylase 2A	KDM2A	0.49*	0.82	0.77	0.73	0.44*	0.45*	0.70
RWD domain-containing protein 1	RWDD1	1.11	1.03	1.05	1.13	0.44*	0.58*	0.71
Translation machinery-associated protein 7	TMA7	0.44*	0.46*	0.65*	0.80	0.44*	0.40*	0.71
Receptor-interacting serine/threonine-protein kinase 2	RIPK2	0.69	0.83	0.85	1.23	0.45*	0.63	0.68
ATP-dependent RNA helicase DHX8	DHX8	0.85	1.18	1.11	1.00	0.45*	0.65	0.87
Mitotic-spindle organizing protein 2B	MZT2B	0.98	0.92	1.03	0.95	0.45*	0.63	0.74
Ribosome biogenesis regulatory protein homolog	RRS1	0.57*	0.64*	0.98	1.07	0.46*	0.53*	0.78
Deoxyhypusine hydroxylase	DOHH	0.99	1.19	1.04	1.07	0.46*	0.59	0.97
Receptor-interacting serine/threonine-protein kinase 3	RIPK3	0.92	0.79	0.97	0.85	0.46*	0.58*	0.69*

Histone chaperone ASF1A	ASF1A	0.86	0.97	0.99	1.00	0.46*	0.54*	0.79
SERPINE1 mRNA-binding protein 1	SERBP1	0.81*	0.82	1.07	0.78	0.47*	0.51*	0.70*
B-cell linker protein	BLNK	0.97	1.17	1.10	1.18	0.47*	0.53*	0.86
Junctophilin-1	JPH1	1.14	0.54*	1.41	1.04	0.48*	0.49*	1.10
PC4 and SFRS1-interacting protein	PSIP1	1.51*	1.06	1.31*	1.61*	0.48*	0.47*	1.46*
Large ribosomal subunit protein mL55	MRPL55	1.09	1.07	1.04	0.96	0.48*	0.57*	0.69
Transcription initiation factor IIE subunit beta	GTF2E2	0.97	1.01	1.04	1.06	0.48*	0.58	0.75
Proliferation marker protein Ki-67	MKI67	0.53*	0.44*	0.69	0.89	0.49*	0.62	1.02
Oxidoreductase-like domain-containing protein 1	OXLD1	1.07	1.12	1.14	1.01	0.49*	0.52*	0.75
Ubiquitin-conjugating enzyme E2 R2	UBE2R2	1.01	1.25	1.22	1.37	0.50*	0.62	1.04
WD repeat-containing protein 55	WDR55	0.88	0.60*	0.71	0.76	0.50*	0.65	0.79
Death-inducer obliterator 1	DIDO1	0.93	1.03	1.03	0.98	0.50*	0.51	0.98
Ribosome biogenesis protein BMS1 homolog	BMS1	0.71	0.65	0.98	0.73	0.51*	0.57	0.79
ATP-dependent RNA helicase DDX24	DDX24	0.56*	0.55*	0.62*	0.81	0.51*	0.51*	0.67*
Envoplakin	EVPL	0.87	0.87	1.06	0.80	0.52*	0.56*	0.74
HEAT repeat-containing protein 3	HEATR3	0.74	0.44*	0.85	0.72	0.52	0.37*	0.80
Nuclear transcription factor Y subunit alpha	NFYA	1.20	1.08	1.17	1.55*	0.52*	0.32*	0.82
PEST proteolytic signal-containing nuclear protein	PCNP	0.88	0.65*	0.93	0.78	0.53*	0.64	0.76
Survival of motor neuron-related-splicing factor 30	SMNDC1	1.02	1.21	1.27	1.27	0.53*	0.65	0.91
Large ribosomal subunit protein eL29	RPL29	0.89	0.85	1.02	1.01	0.53*	0.65*	0.78
Phosphopantothenoylcysteine decarboxylase	PPCDC	0.91	0.83	0.80	1.01	0.53*	0.49*	0.69
Chromobox protein homolog 5	CBX5	1.01	0.95	1.04	0.82	0.53*	0.60*	0.72
Zinc finger protein 638	ZNF638	0.68*	0.84	0.84	0.71	0.53*	0.62	0.78
RNA polymerase II-associated factor 1 homolog	PAF1	0.83	0.80	0.85	0.92	0.53*	0.58	0.75
Protein LSM14 homolog B	LSM14B	0.75	0.77	1.15	0.88	0.54	0.42*	0.89
Insulin-like growth factor 2 mRNA-binding protein 3	IGF2BP3	1.18	0.90	0.98	0.88	0.54*	0.57*	0.72
High mobility group protein HMGI-C	HMGA2	0.68	0.54*	0.81	0.74	0.54	0.64	0.71
Nucleolar and spindle-associated protein 1	NUSAP1	1.44*	1.64*	1.78*	1.33	0.54	0.27*	0.86
7SK snRNA methylphosphate capping enzyme	MEPCE	0.76	0.81	0.96	0.78	0.55	0.62	0.74
Pumilio homolog 1	PUM1	0.85	0.82	0.93	0.70	0.55*	0.66	0.71
Probable RNA-binding protein 19	RBM19	0.69*	0.75	0.83	0.70	0.55*	0.54*	0.74
Signal transducing adapter molecule 1	STAM	0.89	0.89	1.02	0.83	0.55*	0.59*	0.92
Calcium homeostasis endoplasmic reticulum protein	CHERP	0.82	0.84	0.95	0.90	0.55*	0.65*	0.91
Ladinin-1	LAD1	0.97	0.78*	0.94	0.69*	0.55*	0.50*	0.71*
Cancer-related nucleoside-triphosphatase	NTPCR	0.70*	0.79	0.64*	0.86	0.55*	0.65	0.85
Keratin, type II cuticular Hb5	KRT85	0.66*	8.93*	1.06	0.69*	0.55*	0.40*	1.22
Spondin-1	SPON1	0.67*	0.66*	1.05	0.73*	0.56*	0.49*	0.69*

Protein AHNAK2	AHNAK2	0.92	0.86	0.88	0.74*	0.57*	0.49*	0.68*
Mortality factor 4-like protein 1	MORF4L1	0.86	1.13	1.08	0.98	0.57	0.59	0.88
Regulator of nonsense transcripts 3B	UPF3B	0.93	0.84	0.83	1.03	0.57	0.50*	0.75
Ribosome production factor 2 homolog	RPF2	0.73*	0.71*	0.77	0.81	0.57*	0.62	0.76
Neuroblast differentiation-associated protein AHNAK	AHNAK	0.93	0.93	1.07	0.69*	0.58*	0.60*	0.75
FAS-associated factor 1	FAF1	0.96	0.81	0.77	1.04	0.58	0.59	0.69
Protein PAXX	PAXX	1.12	0.97	1.07	1.00	0.58	0.64	0.77
Histone H2B type 1-B	H2BC3	0.66*	0.83	0.98	1.05	0.58	0.63	0.97
RNA-binding motif, single-stranded-interacting protein 2	RBMS2	1.06	0.91	1.23	0.90	0.58	0.65	0.83
Glia maturation factor gamma	GMFG	1.27	1.38	1.29	1.34	0.59	0.62	0.74
Negative elongation factor E	NELFE	0.80	0.86	1.02	0.81	0.59	0.61	0.71
Mediator of RNA polymerase II transcription subunit 15	MED15	0.90	0.70	0.92	0.76	0.59	0.56	0.82
KICSTOR complex protein ITFG2	ITFG2	0.90	0.77	0.67*	0.92	0.59	0.61	0.81
TOX high mobility group box family member 4	TOX4	0.76*	0.97	0.96	0.95	0.59*	0.60	0.67*
PWWP domain-containing protein 2A	PWWP2A	1.12	1.65*	1.22	1.07	0.59	0.61	0.74
TOM1-like protein 1	TOM1L1	0.99	0.93	0.92	1.00	0.59*	0.61	0.76
F-box/LRR-repeat protein 18	FBXL18	0.74*	0.59*	0.64*	0.70	0.60	0.61	0.67
Arginyl-tRNA--protein transferase 1	ATE1	0.90	1.01	1.07	0.68	0.60	0.60	0.67
Histone-lysine N-methyltransferase SETD1A	SETD1A	0.61*	0.70	0.79	0.76	0.60	0.55	0.68
Alpha-taxilin	TXLNA	0.77*	0.83	0.89	0.78*	0.60*	0.64*	0.84
[F-actin]-monooxygenase MICAL3	MICAL3	1.05	1.30	1.09	0.84	0.60	0.62	0.74
DNA polymerase delta catalytic subunit	POLD1	0.62*	0.55*	0.53*	0.73	0.61	0.62	0.85
Allograft inflammatory factor 1-like	AIF1L	1.33*	0.98	1.34*	2.00*	0.61*	0.64	1.17
Ubiquitin-conjugating enzyme E2 R1	CDC34	0.89	0.78	1.01	0.88	0.61	0.63	0.95
Krueppel-like factor 5	KLF5	0.83	0.76	0.82	0.67*	0.61*	0.63*	0.69*
Insulin-like growth factor-binding protein 2	IGFBP2	1.02	1.19	1.29	0.69	0.62	0.57	0.83
Coiled-coil domain-containing protein 50	CCDC50	0.65*	0.81	0.99	1.02	0.62	0.67	1.29
Eukaryotic translation initiation factor 4B	EIF4B	0.92	0.78	1.00	0.78*	0.62*	0.60*	0.77
Methyl-CpG-binding domain protein 2	MBD2	0.74	0.56*	0.76	0.69	0.63	0.60	0.68
Protein strawberry notch homolog 1	SBNO1	0.73	0.68	0.89	0.70	0.63	0.56	0.90
Nucleoredoxin	NXN	0.92	0.89	1.01	0.93	0.63	0.67	0.72
Centrosomal protein of 170 kDa	CEP170	0.89	1.03	1.10	1.06	0.63	0.57	0.98
Microtubule-associated protein RP/EB family member 2	MAPRE2	0.71	0.65*	0.80	0.83	0.63	0.66	0.75
Cytoplasmic polyadenylation element-binding protein 2	CPEB2	0.79	1.18	1.09	0.69	0.63	0.60	0.67
BAG family molecular chaperone regulator 3	BAG3	0.91	0.84	1.06	0.89	0.63	0.58*	0.89
NudC domain-containing protein 2	NUDCD2	0.81	0.88	0.94	0.71	0.64	0.63	0.68

Tuftelin-interacting protein 11	TFIP11	0.90	0.97	1.04	0.90	0.64	0.62	0.85
Histone-lysine N-methyltransferase SETD7	SETD7	0.89	0.70	1.06	0.91	0.64	0.63	0.70
Sciellin	SCEL	0.81*	0.77*	1.09	0.81	0.64*	0.64*	0.86
BMP and activin membrane-bound inhibitor homolog	BAMBI	1.00	1.31*	0.93	1.39*	0.64*	0.61*	1.07
Probable ATP-dependent RNA helicase DDX41	DDX41	0.76*	0.85	0.83	0.79	0.65	0.65	0.78
Sorbin and SH3 domain-containing protein 2	SORBS2	1.28	1.02	0.92	0.96	0.65	0.55	0.71
MKI67 FHA domain-interacting nucleolar phosphoprotein	NIFK	0.99	0.98	1.05	1.01	0.65	0.65	0.83
Macoilin	MACO1	1.00	0.99	1.06	0.97	0.65	0.66	0.69
Neugrin	NGRN	1.04	1.57*	1.71*	1.11	0.65	0.56*	0.90
Asparagine synthetase [glutamine-hydrolyzing]	ASNS	0.76	0.68*	0.97	0.95	0.65	0.66	0.99
Phosphatidate phosphatase LPIN3	LPIN3	0.77	0.49*	0.79	0.70	0.65	0.66	1.02
Pre-mRNA-splicing factor ATP-dependent RNA helicase PRP16	DHX38	0.84	0.66*	0.64*	0.68*	0.65	0.66	0.68*
DNA mismatch repair protein Msh2	MSH2	0.60*	0.57*	0.67*	0.70	0.66	0.64	0.75
Syndecan-1	SDC1	0.64*	0.52*	0.65*	0.81	0.67	0.59	0.73

M. Common down-regulated proteins between LPS-Cytokines with Aquamin and with Mesalamine [37 proteins]

		Interventions						
		Control			*With LPS & Cytokines*			
Proteins	Genes	AQ	AQ+MES	MES	*LPS-Cyto*	***AQ***	*AQ+MES*	***MES***
Group 10 secretory phospholipase A2	PLA2G10	1.13	1.83*	1.30	0.87	0.14*	0.82	0.58
Small nuclear ribonucleoprotein F	SNRPF	2.07*	1.62*	1.69*	1.59*	0.18*	0.89	0.54
Cyclic AMP-dependent transcription factor ATF-2	ATF2	0.70*	0.75	0.87	0.71*	0.29*	0.70	0.51*
Protein cordon-bleu	COBL	0.75	0.96	0.75	0.77	0.33*	0.72	0.60
Eukaryotic translation initiation factor 2D	EIF2D	0.75	0.61*	0.65*	0.73	0.40*	0.77	0.65
DNA dC->dU-editing enzyme APOBEC-3C	APOBEC3C	0.70	0.87	0.84	0.69	0.40*	0.73	0.37*
Pre-mRNA-splicing factor SYF2	SYF2	0.91	1.19	1.22	1.07	0.43*	0.74	0.51*
Mitochondrial assembly of ribosomal large subunit protein 1	MALSU1	0.92	0.82	0.88	1.04	0.44*	0.73	0.53
Receptor-type tyrosine-protein phosphatase gamma	PTPRG	1.17	1.42	1.35	1.14	0.45*	0.71	0.35*
Cyclin-D1-binding protein 1	CCNDBP1	0.91	0.91	0.82	0.69	0.46*	0.76	0.52*
Transmembrane 4 L6 family member 5	TM4SF5	1.24	1.38	0.43*	0.94	0.47*	0.88	0.52
Spermatogenesis-associated protein 20	SPATA20	0.96	0.88	0.77	0.75	0.50*	0.67	0.61*
Tetratricopeptide repeat protein 17	TTC17	0.85	1.15	0.96	0.76	0.52	0.70	0.55
Diacylglycerol kinase zeta	DGKZ	0.78	0.87	0.94	1.11	0.54	0.73	0.57
Sodium-dependent phosphate transporter 1	SLC20A1	1.47*	1.85*	1.42*	0.90	0.54	0.70	0.57
Protein spire homolog 1	SPIRE1	0.86	0.87	0.90	0.70	0.55	0.75	0.50*

Putative nucleoside diphosphate kinase	NME2P1	2.99*	1.90*	1.48*	2.39*	0.55*	0.75	0.60*
Elongation of very long chain fatty acids protein 7	ELOVL7	0.95	0.94	1.07	0.92	0.55*	0.74	0.58*
Mitogen-activated protein kinase kinase kinase 2	MAP3K2	0.81	0.83	0.67	0.76	0.57	0.70	0.44*
Negative elongation factor C/D	NELFCD	1.15	1.00	1.02	0.80	0.58*	0.72	0.62*
Mitochondrial fission regulator 1	MTFR1	0.73	1.14	1.06	0.72	0.59	0.69	0.63
Testis development-related protein	TDRP	1.05	1.04	1.14	0.78	0.60	0.70	0.44*
Natural cytotoxicity triggering receptor 3 ligand 1	NCR3LG1	1.33	0.74	1.17	0.69	0.61	0.77	0.55
Rab9 effector protein with kelch motifs	RABEPK	0.97	0.97	0.96	0.98	0.61	0.68	0.65
Ribosomal protein eL42-like	RPL36AL	0.59*	0.84	0.84	0.70	0.61	0.72	0.65
Metalloreductase STEAP3	STEAP3	1.43*	1.45*	1.12	0.90	0.61	0.82	0.59
Interferon regulatory factor 6	IRF6	1.13	1.02	1.10	0.84	0.63	0.71	0.63*
Caspase activity and apoptosis inhibitor 1	CAAP1	0.97	0.96	0.83	0.74	0.63	0.80	0.66
Kelch-like protein 3	KLHL3	1.27	0.99	0.89	0.86	0.63	1.17	0.61
DNA-directed RNA polymerase, mitochondrial	POLRMT	1.04	0.95	0.97	0.83	0.64	0.74	0.57
Mitofusin-1	MFN1	0.84	1.05	1.04	0.77	0.64	0.78	0.51*
E3 ubiquitin-protein ligase RNF14	RNF14	0.84	1.01	0.80	0.73	0.64	0.73	0.60
Keratin, type I cytoskeletal 20	KRT20	1.04	1.03	0.88	0.94	0.65*	0.84	0.64*
Proline-rich protein 15-like protein	PRR15L	0.92	0.94	0.94	0.69	0.65	0.80	0.57
Threonylcarbamoyladenosine tRNA methylthiotransferase	CDKAL1	0.82	0.96	1.01	0.68	0.65	0.69	0.57
Thioredoxin-like protein 4B	TXNL4B	1.26	1.11	1.35	1.29	0.66	1.11	0.52
Mucin-19	MUC19	1.02	0.89	0.72	0.69	0.67	0.73	0.41*

N. Common down-regulated proteins between LPS-Cytokines with Aquamin plus Mesalamine and with Mesalamine [44 proteins]

		Interventions						
		Control			*With LPS & Cytokines*			
Proteins	Genes	AQ	AQ+MES	MES	*LPS-Cyto*	*AQ*	***AQ+MES***	***MES***
Glutathione S-transferase A2	GSTA2	1.36*	0.40*	0.10*	2.33*	1.56*	0.19*	0.50*
3 beta-hydroxysteroid dehydrogenase/Delta 5-->4-isomerase type 2	HSD3B2	1.43*	0.44*	0.24*	0.88	1.24	0.30*	0.21*
Meprin A subunit beta	MEP1B	1.18	0.37*	0.25*	1.11	1.11	0.36*	0.29*
Keratin, type I cytoskeletal 10	KRT10	0.29*	0.34*	0.79*	0.85	0.85	0.37*	0.57*
Large ribosomal subunit protein mL54	MRPL54	1.08	0.82	0.81	0.94	0.84	0.38*	0.33*
Keratin, type II cytoskeletal 71	KRT71	0.36*	0.52*	0.41*	0.94	1.15	0.39*	0.51*
Carboxypeptidase O	CPO	1.36*	0.54*	0.34*	1.41*	1.27	0.41*	0.39*
Ornithine transcarbamylase, mitochondrial	OTC	1.13	0.62*	0.42*	0.86	1.03	0.41*	0.48*
Proline-rich protein 9	PRR9	1.25	15.97*	11.14*	0.74	1.51	0.43*	0.33*
Growth arrest-specific protein 6	GAS6	1.31*	0.74	0.52*	1.10	1.23	0.47*	0.54*

DCC-interacting protein 13-alpha	APPL1	0.87	0.65*	0.89	0.72*	0.67	0.48*	0.61*
Small proline-rich protein 2D	SPRR2D	0.49*	0.44*	1.18	2.59*	0.70	0.50*	0.58*
Integrin alpha-1	ITGA1	0.90	0.54*	0.51*	0.80	0.98	0.51*	0.52*
Keratin, type I cytoskeletal 14	KRT14	0.51*	0.49*	0.57*	1.84*	0.71*	0.52*	0.61*
Carboxymethylenebutenolidase homolog	CMBL	1.06	0.80	0.84*	0.74*	0.83	0.53*	0.63*
Methyltransferase-like protein 17, mitochondrial	METTL17	1.00	0.69	0.99	0.86	0.78	0.53	0.65
Rhomboid-related protein 2	RHBDL2	0.90	0.80	1.42	0.77	0.73	0.54	0.60
Desmocollin-1	DSC1	0.54*	0.35*	0.53*	0.79	1.12	0.55*	0.56*
E3 ubiquitin-protein ligase SH3RF1	SH3RF1	0.66	0.85	0.79	0.70	0.69	0.55	0.45*
Beta-chimaerin	CHN2	0.86	0.57*	0.62*	0.94	0.85	0.56*	0.58*
Polyhomeotic-like protein 2	PHC2	0.54*	0.75	0.61*	0.74	0.79	0.56	0.55
Iodotyrosine deiodinase 1	IYD	0.94	0.57*	0.46*	0.68*	1.05	0.57*	0.51*
Large ribosomal subunit protein bL34m	MRPL34	1.01	0.83	0.83	0.75	0.79	0.57	0.59
Mitochondrial inner membrane protease ATP23 homolog	ATP23	1.03	0.58*	0.47*	0.82	0.73	0.58	0.46*
Thiosulfate:glutathione sulfurtransferase	TSTD1	0.92	0.78	0.80	0.84	0.71	0.59	0.57
Protein TANC1	TANC1	0.62*	0.71	0.76	0.69	0.87	0.59	0.63
Kinesin-like protein KIF23	KIF23	0.95	0.64*	0.70*	0.85	0.70*	0.59*	0.66*
Coiled-coil domain-containing protein 124	CCDC124	0.57*	0.56*	0.69	0.84	0.69	0.60	0.56
AF4/FMR2 family member 4	AFF4	1.09	1.30	1.21	1.27	1.00	0.60	0.59
ADP-ribosylation factor-like protein 14	ARL14	0.93	0.60*	0.53*	0.92	0.82	0.60	0.63
Uncharacterized protein C1orf198	C1orf198	0.84	1.29	0.88	0.77	0.71	0.60	0.26*
Zinc transporter ZIP5	SLC39A5	1.47*	0.68	0.34*	1.24	1.57	0.61	0.44*
Rho guanine nucleotide exchange factor 12	ARHGEF12	0.78	0.82	0.82	0.68	0.76	0.61	0.66
UDP-glucuronosyltransferase 2B15	UGT2B15	0.87	0.69*	0.53*	0.73	0.92	0.62	0.61*
Putative bifunctional UDP-N-acetylglucosamine transferase and deubiquitinase ALG13	ALG13	1.06	0.87	0.79	0.89	0.68	0.63	0.59*
Dipeptidase 1	DPEP1	1.38*	0.85	0.70*	0.98	1.13	0.63*	0.58*
All trans-polyprenyl-diphosphate synthase PDSS2	PDSS2	1.18	0.97	0.91	0.90	0.77	0.64	0.52*
TGF-beta receptor type-2	TGFBR2	0.83	0.58*	0.65*	0.69	0.94	0.64	0.60
tRNA (cytosine(34)-C(5))-methyltransferase, mitochondrial	NSUN3	0.94	1.02	1.03	0.85	0.78	0.64	0.53
Alpha-galactosidase A	GLA	0.72*	1.44*	1.19*	0.98	0.69*	0.65*	0.65*
Membrane-associated guanylate kinase, WW and PDZ domain-containing protein 1	MAGI1	1.18	1.17	0.88	1.02	0.70	0.65	0.65
UDP-glucuronosyltransferase 2B17	UGT2B17	1.16	1.09	0.79*	0.69*	0.72*	0.66*	0.49*
Acireductone dioxygenase	ADI1	1.00	0.84	0.96	0.89	0.71	0.66	0.66
GTP cyclohydrolase 1 feedback regulatory protein	GCHFR	1.02	0.93	0.70*	0.96	1.12	0.67	0.67

O. Common down-regulated proteins among LPS-Cytokines with Aquamin, with Aquamin plus Mesalamine and with Mesalamine [184 proteins]

		Interventions						
		Control			*With LPS & Cytokines*			
Proteins	Genes	AQ	AQ+MES	MES	*LPS-Cyto*	***AQ***	***AQ+MES***	***MES***
Multiple PDZ domain protein	MPDZ	1.42*	1.28	1.27	1.35	0.05*	0.20*	0.42*
M-phase phosphoprotein 6	MPHOSPH6	1.02	0.90	1.03	0.68	0.05*	0.42*	0.48*
Pre-rRNA-processing protein TSR2 homolog	TSR2	1.13	1.05	1.08	1.05	0.06*	0.23*	0.35*
Zinc finger protein 595	ZNF595	1.36	1.35	1.34	1.19	0.07*	0.59	0.32*
Lysine-specific demethylase 9	RSBN1	0.47*	0.57*	0.46*	0.83	0.08*	0.42*	0.30*
Protein TMED8	TMED8	1.19	1.33	1.27	1.01	0.08*	0.53	0.22*
Surfeit locus protein 6	SURF6	0.69*	0.68*	0.69*	0.87	0.08*	0.19*	0.58*
PRKR-interacting protein 1	PRKRIP1	1.25	0.93	1.09	1.01	0.09*	0.20*	0.34*
Cleavage stimulation factor subunit 2 tau variant	CSTF2T	0.90	1.00	1.09	0.73	0.10*	0.38*	0.55
Transcription initiation factor IIA subunit 1	GTF2A1	1.15	1.08	1.24	1.08	0.12*	0.35*	0.48*
Cyclic AMP-responsive element-binding protein 1	CREB1	0.74*	0.72	0.81	0.71	0.13*	0.32*	0.43*
Glutamyl-tRNA(Gln) amidotransferase subunit A, mitochondrial	QRSL1	1.26*	1.24*	1.30*	0.75*	0.13*	0.24*	0.08*
Tudor domain-containing protein 3	TDRD3	1.39	1.39	2.19*	0.80	0.14*	0.30*	0.42*
Ribosome biogenesis protein NOP53	NOP53	0.97	0.95	1.15	1.14	0.14*	0.25*	0.62*
Signal-induced proliferation-associated 1-like protein 1	SIPA1L1	0.86	1.21	1.14	1.06	0.15*	0.17*	0.49*
E3 ubiquitin-protein ligase RING2	RNF2	0.64*	0.81	0.86	0.85	0.15*	0.24*	0.38*
PSME3-interacting protein	PSME3IP1	0.84	1.03	1.07	0.86	0.16*	0.12*	0.33*
Chromosome alignment-maintaining phosphoprotein 1	CHAMP1	0.53*	0.76	0.62*	0.74	0.17*	0.25*	0.60*
Liprin-alpha-4	PPFIA4	0.39*	0.45*	0.66*	1.37	0.17*	0.20*	0.32*
Proteasome assembly chaperone 4	PSMG4	1.35*	1.22	1.32*	1.11	0.18*	0.24*	0.43*
Ribosome biogenesis protein SLX9 homolog	SLX9	1.03	1.15	1.25	0.93	0.18*	0.26*	0.30*
Nibrin	NBN	0.82	1.02	1.01	1.25	0.18*	0.31*	0.55*
Caspase-5	CASP5	1.06	0.65*	0.95	1.37	0.19*	0.37*	0.45*
Small integral membrane protein 20	SMIM20	1.53*	1.65*	1.62*	1.27*	0.19*	0.38*	0.30*
Uncharacterized protein C1orf122	C1orf122	0.98	1.23	1.01	1.22	0.20*	0.46*	0.54
Acyl-CoA-binding domain-containing protein 6	ACBD6	0.78	0.77	0.90	0.72	0.21*	0.40*	0.35*
Endoribonuclease YbeY	YBEY	1.15	0.98	0.88	0.84	0.21*	0.49*	0.50*
Uncharacterized protein C11orf98	C11orf98	1.07	0.66*	0.84	0.91	0.21*	0.21*	0.25*
Leukocyte receptor cluster member 8	LENG8	1.48*	1.79*	1.57*	1.20	0.21*	0.51*	0.13*
Pseudouridylate synthase RPUSD2	RPUSD2	1.20	1.09	1.13	0.98	0.22*	0.29*	0.31*
GPALPP motifs-containing protein 1	GPALPP1	1.39*	1.10	1.30	1.27	0.24*	0.24*	0.58

ATP-dependent RNA helicase DDX54	DDX54	0.64*	0.60*	0.72	0.75	0.24*	0.34*	0.56
ASNSD1 upstream open reading frame protein	ASDURF	0.89	1.15	1.16*	0.89	0.24*	0.27*	0.37*
Selenoprotein H	SELENOH	1.24*	1.05	1.06	1.15	0.25*	0.35*	0.53*
Coiled-coil-helix-coiled-coil-helix domain-containing protein 5	CHCHD5	1.36*	1.07	0.87	0.88	0.26*	0.40*	0.29*
SH2 domain-containing adapter protein B	SHB	0.86	0.96	0.92	0.74	0.26*	0.37*	0.55*
DNA-directed RNA polymerase I subunit RPA43	POLR1F	0.99	1.01	1.03	0.77	0.26*	0.46*	0.40*
Coiled-coil domain-containing protein 86	CCDC86	0.97	0.88	0.95	1.04	0.27*	0.30*	0.61
DNA excision repair protein ERCC-1	ERCC1	0.84	0.80	0.90	0.89	0.27*	0.36*	0.41*
Transcription factor E2F4	E2F4	0.76	0.86	0.77	0.73	0.27*	0.51*	0.31*
Peptide deformylase, mitochondrial	PDF	1.14	1.27	1.13	1.04	0.28*	0.48*	0.58
Ribosomal RNA processing protein 36 homolog	RRP36	1.13	0.92	1.27	1.22	0.28*	0.30*	0.43*
Myomegalin	PDE4DIP	0.52*	0.90	0.94	0.87	0.30*	0.31*	0.60*
Formin-binding protein 1	FNBP1	0.90	1.21	1.14	0.80	0.30*	0.34*	0.43*
Kynurenine--oxoglutarate transaminase 1	KYAT1	0.87	0.84	0.98	0.85	0.30*	0.48*	0.51*
Y-box-binding protein 3	YBX3	1.16	1.01	1.30	0.77	0.31*	0.47*	0.65
WASH complex subunit 2C	WASHC2C	0.85	0.84	0.96	1.02	0.31*	0.42*	0.58*
Protein FAN	NSMAF	0.85	0.90	1.07	0.78	0.31*	0.36*	0.36*
Ribosomal RNA processing protein 1 homolog B	RRP1B	0.84	0.85	0.81*	0.71*	0.31*	0.39*	0.45*
Cdc42 effector protein 4	CDC42EP4	1.18	0.89	1.25	0.79	0.32*	0.51*	0.40*
Cyclin-dependent kinase inhibitor 2A	CDKN2A	1.01	1.03	1.18	1.53*	0.32*	0.36*	0.49*
KAT8 regulatory NSL complex subunit 3	KANSL3	1.13	0.85	0.80	0.99	0.33*	0.35*	0.33*
Regulation of nuclear pre-mRNA domain-containing protein 1A	RPRD1A	0.79*	0.77	0.81*	0.78	0.33*	0.53*	0.50*
Myocyte-specific enhancer factor 2D	MEF2D	0.78	1.08	1.11	0.83	0.33*	0.41*	0.65
Max-like protein X	MLX	0.96	1.07	0.98	0.86	0.33*	0.38*	0.47*
Tubulin-specific chaperone C	TBCC	0.92	0.86	0.97	1.05	0.34*	0.47*	0.60*
Splicing factor Cactin	CACTIN	1.09	1.00	1.12	1.04	0.35*	0.40*	0.56*
Protein SDA1 homolog	SDAD1	0.65*	0.70	0.69*	0.69	0.35*	0.42*	0.47*
Ubiquitin-like domain-containing CTD phosphatase 1	UBLCP1	0.93	1.03	1.08	0.98	0.35*	0.51*	0.63*
N6-adenosine-methyltransferase catalytic subunit	METTL3	1.04	1.24	1.19	0.71*	0.36*	0.32*	0.49*
Transmembrane 4 L6 family member 4	TM4SF4	0.69	0.68	0.62*	0.67	0.36*	0.58	0.59
A-kinase anchor protein 2	PALM2AKAP2	1.01	0.97	1.24	0.92	0.36*	0.58	0.67
Nucleoplasmin-3	NPM3	1.99*	1.31	1.49	2.28*	0.37*	0.65	0.30*
DNA repair protein XRCC4	XRCC4	1.08	1.04	1.20	0.99	0.37*	0.48*	0.49*
Ubiquinol-cytochrome-c reductase complex assembly factor 6	UQCC6	1.50*	1.63*	1.62*	1.17	0.37*	0.43*	0.39*
5-methylcytosine rRNA methyltransferase NSUN4	NSUN4	0.91	0.99	0.97	0.81	0.38*	0.56	0.35*

GrpE protein homolog 2, mitochondrial	GRPEL2	1.26*	1.12	1.07	0.79	0.38*	0.43*	0.38*
Bromodomain-containing protein 4	BRD4	1.20*	1.20	1.19*	0.96	0.38*	0.50*	0.58*
DnaJ homolog subfamily C member 21	DNAJC21	0.94	0.94	0.87	0.82	0.38*	0.41*	0.55*
Actin-related protein 5	ACTR5	0.96	0.83	1.04	0.80	0.39*	0.64	0.54
Large ribosomal subunit protein mL65	MRPS30	0.95	0.89	0.84	0.72*	0.39*	0.45*	0.34*
Endoplasmic reticulum resident protein 27	ERP27	1.40*	1.22	1.11	0.83	0.39*	0.57	0.37*
Sorting nexin-15	SNX15	1.13	1.03	0.91	0.76	0.40*	0.45*	0.36*
Fos-related antigen 2	FOSL2	0.85	0.88	1.00	0.74	0.40*	0.37*	0.41*
E3 ubiquitin-protein ligase ZNRF2	ZNRF2	0.99	1.08	1.00	0.81	0.40*	0.52*	0.51*
Pleckstrin homology-like domain family B member 1	PHLDB1	0.73*	0.61*	0.53*	0.70*	0.41*	0.32*	0.43*
AN1-type zinc finger protein 2B	ZFAND2B	0.91	0.99	0.88	0.76	0.42*	0.59	0.51*
SID1 transmembrane family member 2	SIDT2	1.03	1.02	0.97	0.88	0.42*	0.50	0.40*
DNA polymerase subunit gamma-1	POLG	0.77	0.98	0.89	0.71	0.42*	0.45*	0.42*
Zinc finger protein 330	ZNF330	0.83	0.85	0.81	0.70	0.42*	0.55	0.52*
Metallothionein-1H	MT1H	0.61*	0.90	0.77	0.97	0.42*	0.55	0.62
Shootin-1	SHTN1	0.79*	0.84	0.87	0.76*	0.43*	0.45*	0.54*
p53 and DNA damage-regulated protein 1	PDRG1	0.91	1.18	1.19	1.01	0.43*	0.49*	0.65*
Ribosome biogenesis protein NSA2 homolog	NSA2	0.71*	0.71*	0.76	0.88	0.44*	0.48*	0.62
Fatty acyl-CoA reductase 1	FAR1	0.73	0.75	0.99	0.67	0.44*	0.48*	0.36*
E3 ubiquitin-protein ligase RNF25	RNF25	0.77	0.99	0.84	0.79	0.44*	0.47*	0.62
Nucleolar protein 10	NOL10	0.72*	0.67*	0.82	0.68*	0.44*	0.49*	0.51*
TP53-regulated inhibitor of apoptosis 1	TRIAP1	1.35	1.33	1.20	1.16	0.45*	0.59	0.67
Death-associated protein 1	DAP	0.68	0.53*	0.55*	0.77	0.45*	0.43*	0.48*
SUN domain-containing ossification factor	SUCO	0.95	0.99	0.77	0.68	0.45*	0.39*	0.35*
Alpha-ketoglutarate-dependent dioxygenase alkB homolog 7, mitochondrial	ALKBH7	0.95	0.89	0.84	0.79	0.45*	0.45*	0.29*
2-(3-amino-3-carboxypropyl)histidine synthase subunit 2	DPH2	1.00	1.13	1.16	0.89	0.46*	0.30*	0.45*
Replication termination factor 2	RTF2	0.68*	0.83	0.82	0.68	0.46*	0.48*	0.49*
Kanadaptin	SLC4A1AP	0.79	0.88	0.82	0.89	0.46*	0.61	0.55
Protein FAM83G	FAM83G	1.12	1.58*	1.13	0.85	0.46*	0.56	0.54
28S rRNA (cytosine-C(5))-methyltransferase	NSUN5	0.76*	0.72*	0.80*	0.75*	0.47*	0.51*	0.53*
ADP-ribosylation factor-like protein 15	ARL15	0.91	0.97	0.78	0.84	0.47*	0.53*	0.53*
Serine/threonine-protein kinase N1	PKN1	0.71	0.71	0.84	0.82	0.47*	0.63	0.64
Receptor-type tyrosine-protein phosphatase F	PTPRF	0.52*	0.48*	0.95	0.70*	0.47*	0.47*	0.64*
DENN domain-containing protein 1A	DENND1A	0.80	0.86	1.18	0.78	0.48*	0.59	0.61
MAP kinase-interacting serine/threonine-protein kinase 1	MKNK1	0.76	0.68	0.83	0.71	0.48*	0.55*	0.61

Zinc finger FYVE domain-containing protein 21	ZFYVE21	0.95	0.83	0.83	0.77	0.48*	0.56*	0.57*
Katanin p60 ATPase-containing subunit A1	KATNA1	0.84	0.95	1.29	0.80	0.48*	0.51	0.43*
Putative monooxygenase p33MONOX	KIAA1191	1.29	1.25	1.40*	0.92	0.48*	0.44*	0.43*
CCAAT/enhancer-binding protein zeta	CEBPZ	0.71*	0.70*	0.69*	0.74	0.48*	0.58*	0.67
tRNA (guanine(37)-N1)-methyltransferase	TRMT5	1.09	0.88	0.98	0.94	0.48*	0.53*	0.40*
tRNA dimethylallyltransferase	TRIT1	0.93	0.94	0.77	0.89	0.49*	0.52*	0.31*
Zinc finger protein 22	ZNF22	0.46*	0.37*	0.55*	0.77	0.49*	0.35*	0.36*
UPF0462 protein C4orf33	C4orf33	1.07	0.98	0.89	0.77	0.49*	0.46*	0.43*
Glycylpeptide N-tetradecanoyltransferase 2	NMT2	0.89	1.06	1.00	0.70*	0.49*	0.54*	0.55*
Lariat debranching enzyme	DBR1	0.90	0.70	0.90	0.80	0.49*	0.60	0.58
Multivesicular body subunit 12A	MVB12A	0.94	0.64*	0.98	0.89	0.49*	0.65	0.61
TRAF family member-associated NF-kappa-B activator	TANK	0.61*	0.64	0.66*	0.91	0.50*	0.50*	0.55
Cysteine protease ATG4C	ATG4C	1.22	1.51	1.51*	1.04	0.50*	0.27*	0.39*
Rootletin	CROCC	0.49*	1.51*	0.96	0.74	0.50*	0.53*	0.43*
MOB kinase activator 3B	MOB3B	0.71	0.93	0.82	0.73	0.51*	0.65	0.56
Selenoprotein W	SELENOW	1.07	0.68	0.66*	0.86	0.51*	0.45*	0.40*
Prostaglandin reductase 3	PTGR3	1.10	1.09	1.08	0.75	0.51*	0.60*	0.37*
N-terminal Xaa-Pro-Lys N-methyltransferase 1	NTMT1	0.83	0.88	0.84	0.84	0.51	0.55	0.54*
Translational activator of cytochrome c oxidase 1	TACO1	0.98	1.04	0.92	0.89	0.51*	0.64*	0.60*
Small ribosomal subunit protein bS21m	MRPS21	0.94	0.91	0.82	0.79	0.52*	0.58*	0.56*
Pseudouridylate synthase TRUB1	TRUB1	1.04	1.11	1.03	0.73*	0.52*	0.57*	0.56*
Ribosome biogenesis protein BOP1	BOP1	0.66*	0.58*	0.70*	0.77	0.53*	0.54*	0.56
SNARE-associated protein Snapin	SNAPIN	0.84	0.89	1.04	0.78	0.53*	0.64	0.60
tRNA (guanine(10)-N2)-methyltransferase homolog	TRMT11	0.96	0.98	0.90	0.96	0.53	0.67	0.61
Protein N-terminal glutamine amidohydrolase	NTAQ1	0.79	1.04	0.94	0.70	0.53	0.64	0.47*
Peroxisomal acyl-coenzyme A oxidase 2	ACOX2	1.19	1.07	0.89	0.75*	0.53*	0.51*	0.41*
Transcription initiation factor TFIID subunit 7	TAF7	0.91	0.90	0.80	1.04	0.53*	0.52*	0.61
Protein KTI12 homolog	KTI12	1.00	1.10	1.20	0.98	0.54	0.47*	0.47*
Actin-binding LIM protein 2	ABLIM2	0.78	0.89	0.89	0.68*	0.54*	0.59	0.58*
IgGFc-binding protein	FCGBP	1.09	1.13	1.23	0.82	0.54	0.51*	0.57
Protein AATF	AATF	0.61*	0.52*	0.65*	0.82	0.54*	0.54*	0.65
DNA repair endonuclease XPF	ERCC4	0.69	0.78	0.78	0.75	0.54	0.57	0.50*
Phenylalanine--tRNA ligase, mitochondrial	FARS2	0.88	0.83	0.80	0.71*	0.54*	0.52*	0.50*
SURP and G-patch domain-containing protein 1	SUGP1	1.18	1.25	1.06	1.09	0.55	0.66	0.65
BAG family molecular chaperone regulator 4	BAG4	0.91	0.89	0.93	0.81	0.55	0.58	0.67
Probable N-acetyltransferase 14	NAT14	0.90	0.93	1.08	0.95	0.55*	0.50*	0.43*
Transforming acidic coiled-coil-containing protein 1	TACC1	0.94	0.62*	0.65*	0.84	0.56*	0.47*	0.61

Calcium/calmodulin-dependent protein kinase type 1D	CAMK1D	0.92	1.15	0.95	0.81	0.56*	0.61	0.39*
Ephrin type-A receptor 1	EPHA1	0.71*	0.85	0.88	0.73	0.56*	0.65	0.55*
Lon protease homolog 2, peroxisomal	LONP2	0.89	0.94	0.85	0.71	0.56	0.54*	0.48*
Aminomethyltransferase, mitochondrial	AMT	0.98	0.99	0.96	0.77	0.57*	0.63	0.52*
Importin subunit alpha-1	KPNA2	0.83	1.20	1.27*	0.89	0.58*	0.59*	0.63*
Jupiter microtubule associated homolog 1	JPT1	0.63*	0.58*	0.86	0.78	0.58	0.49*	0.65
Phosphoprotein associated with glycosphingolipid-enriched microdomains 1	PAG1	1.00	1.02	1.11	0.85	0.58	0.56*	0.61
Ligand-dependent corepressor	LCOR	0.88	0.85	0.89	0.72	0.58	0.62	0.58
StAR-related lipid transfer protein 5	STARD5	1.16	1.12	1.05	0.89	0.59	0.63	0.55*
Tetratricopeptide repeat protein 39A	TTC39A	1.06	0.89	0.75*	0.77	0.59*	0.63*	0.47*
Nucleolar protein 9	NOP9	0.70	0.71	0.85	0.84	0.59	0.55	0.65
Cytosolic iron-sulfur assembly component 2A	CIAO2A	1.03	0.91	0.80*	0.78	0.59*	0.62	0.58*
Transcription initiation factor IIA subunit 2	GTF2A2	1.36	1.35	1.54*	1.37	0.59	0.36*	0.48*
Kynurenine formamidase	AFMID	0.96	0.74	0.63*	0.86	0.60*	0.58*	0.56*
Kinesin-like protein KIF1B	KIF1B	0.75	0.64*	0.78	0.67	0.60	0.64	0.56
Mitochondrial tRNA methylthiotransferase CDK5RAP1	CDK5RAP1	0.68	0.62*	0.56*	0.69	0.60	0.56	0.58
Telomeric repeat-binding factor 2	TERF2	1.54*	1.23	1.29	1.43	0.60	0.56	0.66
Ethanolamine kinase 1	ETNK1	1.21	1.20	0.99	0.88	0.61	0.66	0.64
Frizzled-6	FZD6	0.99	1.22	1.31	0.81	0.61	0.51	0.65
Protein FAM118B	FAM118B	0.81*	0.72*	0.75*	0.89	0.61*	0.60*	0.62*
Mothers against decapentaplegic homolog 5	SMAD5	0.84	0.87	0.86	0.67	0.61	0.51*	0.53*
Biotinidase	BTD	1.46*	1.07	0.92	0.97	0.61*	0.62*	0.58*
Ral GTPase-activating protein subunit alpha-1	RALGAPA1	0.64*	0.71	0.75	0.76	0.62	0.55	0.61
Histone-lysine N-trimethyltransferase SMYD5	SMYD5	0.97	0.88	0.75	1.14	0.62	0.63	0.67
ATP-dependent RNA helicase DDX50	DDX50	0.81	0.80	0.84	0.81	0.62	0.52*	0.62*
Unconventional prefoldin RPB5 interactor 1	URI1	0.73	0.74	0.72	0.89	0.62	0.53	0.64
NAD kinase	NADK	0.84	0.85	0.77	0.89	0.63	0.66	0.65
8-oxo-dGDP phosphatase NUDT18	NUDT18	1.02	0.93	0.92	0.83	0.63	0.53	0.62
[F-actin]-monooxygenase MICAL2	MICAL2	0.79	0.67*	0.86	0.69	0.63	0.51*	0.60*
Inositol polyphosphate multikinase	IPMK	0.88	0.88	0.70*	0.70	0.63	0.64	0.55*
NEDD8-conjugating enzyme UBE2F	UBE2F	0.80	0.74	0.74	0.71	0.64	0.65	0.65
Rac GTPase-activating protein 1	RACGAP1	0.98	0.66*	0.69*	0.80	0.64*	0.52*	0.57*
COX assembly mitochondrial protein 2 homolog	CMC2	1.24*	1.12	1.05	0.96	0.64	0.56*	0.51*
Fatty acid-binding protein, intestinal	FABP2	1.03	0.91	0.93	0.72*	0.64*	0.59*	0.63*
Transcription factor Sp6	SP6	0.94	1.06	0.92	0.83	0.65	0.53	0.32*
5-phosphohydroxy-L-lysine phospho-lyase	PHYKPL	0.94	0.88	0.73*	0.80	0.65	0.64	0.58*
Solute carrier family 12 member 9	SLC12A9	1.02	0.83	0.88	0.80	0.65	0.57*	0.53*

Thioredoxin-related transmembrane protein 4	TMX4	0.79	0.58*	0.73	0.70	0.65	0.46*	0.43*
RalA-binding protein 1	RALBP1	0.81	0.83	0.73*	0.79	0.65	0.59	0.64
Origin recognition complex subunit 2	ORC2	0.72*	0.82	0.81	0.76	0.65	0.66	0.61*
Ras-related protein Rab-20	RAB20	0.88	0.81	0.76*	0.72*	0.66	0.60*	0.55*
Gap junction beta-1 protein	GJB1	0.62*	0.56*	0.56*	0.75	0.66	0.53*	0.58*
Protein phosphatase 1 regulatory subunit 1B	PPP1R1B	1.00	1.05	1.01	0.79	0.66	0.66	0.53*
Kinesin-like protein KIF13A	KIF13A	0.62*	0.77	0.74*	0.69	0.66	0.63	0.66
Nuclear prelamin A recognition factor	NARF	0.85	0.61*	0.79	0.81	0.66	0.61	0.60
Ribonucleoside-diphosphate reductase subunit M2	RRM2	1.74*	1.38	1.58*	0.79	0.67	0.60	0.55*

Values represent the abundance ratio from organoids (n=4 subjects) compared to the control. These proteins were down-regulated at a 1.5-fold change (<2% FDR). Corresponding abundance ratios from the other treatment groups are provided for comparison. Proteins common among groups and unique to individual groups under proinflammatory conditions (LPS-Cytokines) are presented. *Indicates significance compared to the control (at $p<0.05$).
