# Supplementary material for: Proteomic profile of human colon organoids: effects of a multi-mineral intervention alone and in the presence of pro-inflammatory and anti-inflammatory treatments
Source: Front Gastroenterol (Lausanne). 2025 Jul 2;4:1592669. doi: 10.3389/fgstr.2025.1592669 (PMC12952359; doi:10.3389/fgstr.2025.1592669)
Supplement: Supplementary Table S1 — Mineral composition of Aquamin. [file DataSheet1.zip › Table S7.pdf]

Supplement Table 7. Proteins influenced by Aquamin and Mesalamine under LPS-Cytokines conditions

**7A. Proteins presented in Figure 3**		Interventions						
		Control				*With LPS & Cytokines*		
Proteins	Genes	Control	AQ	AQ+MES	MES	***AQ***	***AQ+MES***	***MES***
**A. Cell-cell junction**								
Tight junction protein ZO-1	TJP1	1.02	1.03	1.07	1.16	0.71*	0.81	0.99
Tight junction protein ZO-2	TJP2	1.10	1.01	0.97	1.15	0.82*	0.89	0.99
Tight junction protein ZO-3	TJP3	1.09	1.12	1.09	1.19	0.97	1.04	1.04
Claudin-1	CLDN1	1.08	0.57*	0.89	1.15	0.92	0.70	1.15
Claudin-3	CLDN3	0.95	0.93	1.07	1.00	0.91	1.18	1.16
Claudin-4	CLDN4	1.17	1.27	1.48*	1.15	1.14	1.33	1.03
Claudin-7	CLDN7	0.89	0.93	0.91	0.85	1.13	1.06	0.88
Claudin-23	CLDN23	0.97	0.84	0.92	0.95	0.76	0.88	1.12
Occludin	OCLN	0.90	0.93	0.92	0.96	0.99	1.03	0.92
Junctional adhesion molecule A	JAM1	0.82	0.87	0.92	0.88	1.03	1.06	0.91
Cadherin-1	CDH1	0.95	0.93	0.94	0.93	0.89	0.81*	0.81*
Cadherin-3	CDH3	0.95	1.46*	1.50*	0.95	1.32	1.29	0.78
Cadherin-13	CDH13	1.13	0.95	2.73*	3.56*	1.14	2.76*	4.34*
Cadherin-17	CDH17	1.10	3.94*	3.81*	1.15	2.95*	2.92*	0.80*
Cadherin-related family member 2	CDHR2	1.27	1.35*	1.35*	1.13	1.22	0.86	0.78*
Cadherin-related family member 5	CDHR5	1.08	1.26	1.57*	0.95	1.30	1.18	0.90
Protocadherin-1	PCDH1	1.13	2.38*	2.37*	1.29	2.19*	2.21*	1.28
Protocadherin-12	PCDH12	0.56*	0.26*	0.76	0.59*	1.61	2.68*	3.87*
Protocadherin gamma-C3	PCDHGC3	0.68*	0.37*	0.94	0.93	1.88*	2.48*	3.53*
Desmocollin-2	DSC2	1.20	1.64*	1.57*	1.25	1.36*	1.38*	0.97
Desmoglein-1	DSG1	0.90	0.60*	0.74	0.87	1.01	0.62*	1.06
Desmoglein-2	DSG2	0.92	2.00*	2.04*	0.86	1.87*	1.88*	1.34
Desmoglein-3	DSG3	1.70	1.15	5.50*	7.19*	1.60	2.66*	4.06*
Desmoplakin	DSP	0.97	0.97	0.96	0.77*	1.02	0.97	0.86
Junction plakoglobin	JUP	1.06	1.24	1.29	1.03	1.27*	1.18	0.93
Plectin	PLEC	1.42*	1.66*	1.21	0.95	0.95	1.06	1.33
**B. Mucin-related**								
Mucin-1	MUC1	0.55*	0.67*	0.62*	0.58*	1.06	1.07	0.84
Mucin-2	MUC2	1.30*	1.16	1.47*	1.47*	1.37*	1.41*	1.38*
Mucin-3A	MUC3A	0.95	0.95	2.35*	1.92*	1.15	1.66*	1.59*

Mucin-3B	MUC3B	1.07	1.38	1.54*	1.50*	1.19	1.58*	1.23
Mucin-4	MUC4	0.74	0.88	0.65*	0.70*	1.11	1.13	0.95
Mucin-5AC	MUC5AC	1.21	1.15	0.91	1.32*	0.97	1.46*	1.10
Mucin-5B	MUC5B	1.05	1.17	0.89	0.92	1.06	0.94	0.85
Mucin-12	MUC12	1.19	1.67*	1.25	1.12	1.03	1.13	0.88
Mucin-13	MUC13	0.86	0.89	1.03	0.88	1.23*	1.21*	1.12
Trefoil factor 1	TFF1	1.63*	2.51*	1.76*	1.48*	1.03	0.79	0.61*
Trefoil factor 2	TFF2	2.40*	3.02*	1.47	1.43	1.41	0.81	0.80
Trefoil factor 3	TFF3	1.20	1.68*	1.23	1.03	1.10	0.99	0.83
Glycoprotein-N-acetylgalactosamine 3-beta-galactosyltransferase 1	C1GALT1	0.94	0.97	1.23	0.96	1.29*	1.38*	1.13
C1GALT1-specific chaperone 1	C1GALT1C1	0.86	0.87	1.02	0.84	1.12	1.19	0.97
CMP-N-acetylneuraminate-beta-galactosamide-alpha-2,3-sialyltransferase 1	ST3GAL1	0.66*	0.63*	0.69	0.73	1.47*	1.46*	1.21
IgGFc-binding protein	FCGBP	1.22	1.34	1.38	1.50	0.66	0.63	0.70
Zymogen granule membrane protein 16	ZG16	0.85	1.05	1.62*	1.13	0.98	0.74*	1.02
**C. Basement membrane**								
Laminin subunit alpha-1	LAMA1	0.62*	0.34*	1.26	1.04	1.43*	1.80*	2.47*
Laminin subunit alpha-2	LAMA2	0.75	0.80	0.90	1.29	1.02	1.16	1.33
Laminin subunit alpha-4	LAMA4	0.49*	0.54*	0.46*	0.44*	1.11	1.25	1.38*
Laminin subunit alpha-5	LAMA5	0.55*	0.57*	0.79	0.79	0.86	1.08	1.41*
Laminin subunit beta-1	LAMB1	0.64*	0.34*	1.29	1.05	1.52*	1.79*	2.40*
Laminin subunit beta-2	LAMB2	0.72*	0.49*	1.38*	0.94	1.33*	1.59*	2.21*
Laminin subunit beta-3	LAMB3	1.01	1.06	0.94	1.08	1.04	1.01	0.99
Laminin subunit gamma-1	LAMC1	0.65*	0.40*	1.28	1.05	1.48*	1.74*	2.37*
Laminin subunit gamma-2	LAMC2	1.02	1.04	1.00	1.21	1.14	1.05	1.11
Basement membrane-specific heparan sulfate proteoglycan core protein	HSPG2	0.85	0.73*	1.24	1.03	1.28*	1.57*	1.76*
Nidogen-1	NID1	0.71*	0.53*	1.10	1.02	1.32*	1.57*	2.05*
Nidogen-2	NID2	0.69*	0.50*	1.30	1.11	1.39*	1.76*	2.36*
Cell adhesion molecule 1	CADM1	1.59	1.03	2.99*	3.24*	1.41	2.80*	3.56*

**7B. Proteins presented in Figure 5**		Interventions						
		Control				*With LPS & Cytokines*		
Proteins	Genes	Control	AQ	AQ+MES	MES	***AQ***	***AQ+MES***	***MES***
**A. Complement**								
Complement C2	C2	1.47	1.03	4.13*	7.20*	1.16	4.92*	5.74*

Complement C3	C3	0.35*	0.24*	0.79	1.33*	1.21	1.56*	1.35*
Complement C4-A	C4A	0.55*	0.40*	0.71*	1.19	1.80*	2.12*	2.55*
C4b-binding protein alpha chain	C4BPA	0.67*	0.32*	0.67	0.94	0.71	0.84	1.12
Complement C5	C5	0.55*	0.56*	0.73	0.81	1.72*	2.34*	3.00*
Complement component C7	C7	0.68	0.49*	0.74	0.90	1.88*	2.89*	3.32*
Complement component C8 alpha chain	C8A	0.60*	0.36*	0.69*	0.82	2.09*	2.73*	3.61*
Complement component C8 gamma chain	C8G	0.55*	1.09	1.18	2.49*	1.55*	1.09	0.70*
Complement component C9	C9	0.77	0.65*	1.14	1.16	1.72*	2.56*	2.67*
Complement decay-accelerating factor	CD55	0.75*	0.75*	0.96	0.97	0.95	1.06	0.98
CD59 glycoprotein	CD59	0.77	0.93	0.71*	0.92	1.02	0.98	0.98
Complement factor B	CFB	0.46*	0.52*	0.67	0.77	1.48	1.09	1.12
Complement factor D	CFD	1.23	0.72	1.77*	4.59*	0.72	1.91*	2.53*
Complement factor H	CFH	1.35	0.84	2.75*	36.53*	0.83	2.28*	2.33*
Complement factor I	CFI	0.34*	0.23*	0.68*	0.71*	2.08*	2.05*	3.81*
Clusterin	CLU	0.77	0.64	0.71	1.01	1.24	1.15	1.59*
Collectin-10	COLEC10	0.82	0.50*	0.94	0.93	2.14*	3.08*	3.95*
Collectin-11	COLEC11	0.77	0.21*	0.61*	0.88	2.00*	2.77*	4.07*
Carboxypeptidase B2	CPB2	0.57*	0.13*	0.58*	0.70*	3.06*	3.01*	5.74*
Carboxypeptidase N catalytic chain	CPN1	0.70*	0.39*	0.64*	0.64*	2.17*	3.16*	3.54*
Neutrophil elastase	ELANE	0.77	0.74	1.42	0.64	2.51*	0.70	1.74*
Phospholipid scramblase 1	PLSCR1	0.63*	0.35*	0.65*	0.68*	0.59*	0.97	1.10
Vitronectin	VTN	0.63*	0.34*	0.84	0.96	0.98	1.22	1.75*
**B. Clotting/Fibrinolytic**								
Alpha-2-macroglobulin	A2M	0.53*	0.33*	0.79	0.99	0.94	1.34*	1.85*
Amyloid-beta precursor protein	APP	1.00	0.85	1.04	1.01	0.91	1.00	1.08
Coagulation factor X	F10	0.88	0.37*	0.68*	0.72	2.99*	4.48*	4.95*
Coagulation factor XIII A chain	F13A1	0.70	0.55*	0.78	0.94	0.95	1.13	1.46
Prothrombin	F2	0.66*	0.56*	1.06	0.93	1.39	2.07*	2.53*
Tissue factor	F3	0.75*	0.72*	1.19	1.11	0.92	1.26	1.18
Coagulation factor V	F5	0.57*	0.35*	0.70	0.72	0.93	1.08	1.39*
Fibrinogen alpha chain	FGA	0.47*	0.30*	0.32*	1.21	0.36*	0.50*	1.15
Fibrinogen beta chain	FGB	0.52*	0.29*	1.06	0.87	1.84*	1.84*	3.22*
Fibrinogen gamma chain	FGG	0.39*	0.14*	0.50*	0.92	1.35*	1.32*	2.41*
Plasma kallikrein	KLKB1	1.39	0.82	2.87*	3.97*	1.14	2.63*	3.78*
Kininogen-1	KNG1	0.58*	0.27*	1.33	1.59	0.39*	1.34	1.36
Multiple coagulation factor deficiency protein 2	MCFD2	1.01	1.50*	1.48*	1.13	1.46*	1.70*	1.24
Urokinase-type plasminogen activator	PLAU	0.73	0.70	1.00	0.86	0.97	1.04	1.16

Urokinase plasminogen activator surface receptor	PLAUR	0.76	0.82	0.97	0.91	0.93	1.07	0.91
Plasminogen	PLG	0.69	0.43*	0.91	0.96	1.12	1.03	1.27
Endothelial protein C receptor	PROCR	1.13	1.09	1.16	1.20	1.03	0.95	1.08
Vitamin K-dependent protein Z	PROZ	0.48*	0.39*	0.37*	0.44*	2.41*	1.95*	2.36*
Alpha-1-antitrypsin	SERPINA1	0.34*	0.43*	0.60*	0.48*	0.96	0.83	0.80*
Plasminogen activator inhibitor 2	SERPINB2	0.64*	0.68	0.84	0.74	1.05	1.07	1.09
Antithrombin-III	SERPINC1	0.54*	0.37*	0.65*	0.84	0.78*	1.02	1.23*
Heparin cofactor 2	SERPIND1	0.49*	0.37*	0.85	1.35	1.88*	2.13*	3.62*
Alpha-2-antiplasmin	SERPINF2	0.54*	0.47*	0.88	0.91	1.34	2.77*	3.36*
Thrombomodulin	THBD	0.59*	0.33*	0.37*	0.35*	4.40*	3.93*	4.24*
Thrombospondin-1	THBS1	0.81	0.75*	0.78*	0.95	0.87	1.17	1.86*
von Willebrand factor	VWF	1.69*	0.57*	2.60*	2.79*	0.72	2.12*	3.25*
**C. Antigen presenting**								
Allograft inflammatory factor 1-like	AIF1L	0.50*	0.67*	0.49*	0.67*	0.30*	0.32*	0.58*
HLA class I histocompatibility antigen, A alpha chain	HLA-A	0.25*	0.28*	0.33*	0.29*	1.05	1.14	0.96
HLA class I histocompatibility antigen, B alpha chain	HLA-B	0.13*	0.19*	0.31*	0.20*	0.78	1.04	1.15
HLA class I histocompatibility antigen, C alpha chain	HLA-C	0.29*	0.32*	0.38*	0.36*	1.03	1.24*	1.00
HLA class II histocompatibility antigen, DM alpha chain	HLA-DMA	0.98	1.40	5.38*	4.86*	1.11	5.13*	4.29*
HLA class II histocompatibility antigen, DP alpha 1 chain	HLA-DPA1	0.11*	0.11*	0.10*	0.13*	0.94	0.95	1.06
HLA class II histocompatibility antigen, DP beta 1 chain	HLA-DPB1	0.07*	0.02*	0.15*	0.12*	0.90	1.04	0.99
HLA class II histocompatibility antigen, DR alpha chain	HLA-DRA	0.04*	0.04*	0.06*	0.04*	0.86	1.02	0.88
HLA class II histocompatibility antigen, DRB1 beta chain	HLA-DRB1	0.03*	0.03*	0.03*	0.04*	0.77	0.95	0.93
HLA class II histocompatibility antigen, DR beta 3 chain	HLA-DRB3	0.08*	0.07*	0.08*	0.13*	0.80	0.81	0.93
HLA class II histocompatibility antigen, DR beta 4 chain	HLA-DRB4	1.20	0.85	4.60*	4.99*	1.30	5.60*	4.31*
HLA class II histocompatibility antigen, DR beta 5 chain	HLA-DRB5	0.49*	0.42*	0.49*	0.38*	1.19	0.97	1.08
HLA class I histocompatibility antigen, alpha chain E	HLA-E	0.33*	0.32*	0.44*	0.40*	0.70*	0.67*	0.71*
HLA class I histocompatibility antigen, alpha chain F	HLA-F	0.19*	0.23*	0.28*	0.25*	0.84	1.00	0.96
HLA class I histocompatibility antigen, alpha chain G	HLA-G	0.46*	0.52*	0.66*	0.60*	1.23	1.13	0.88
Putative HLA class I histocompatibility antigen, alpha chain H	HLA-H	0.34*	0.40*	0.55*	0.40*	0.89	0.95	0.82
Intercellular adhesion molecule 1	ICAM1	0.39*	0.32*	0.35*	0.40*	1.02	0.99	1.00

Intercellular adhesion molecule 2	ICAM2	0.43*	0.57*	0.49*	0.64*	0.99	0.75	0.97
Antigen peptide transporter 1	TAP1	0.23*	0.25*	0.29*	0.25*	1.02	1.14	0.95
Antigen peptide transporter 2	TAP2	0.26*	0.25*	0.27*	0.26*	1.08	1.11	0.93
Vascular cell adhesion protein 1	VCAM1	0.49*	0.26*	0.56*	0.62*	1.95*	2.72*	3.30*
CD166 antigen	ALCAM	0.48*	0.53*	0.93	0.83	0.58*	1.02	0.85
**D. Proteasomes**								
Proteasome subunit beta type-5	PSMB5	1.54*	1.39	1.25	1.29	0.93	1.10	0.87
Proteasome subunit beta type-6	PSMB6	1.98*	1.92*	2.02*	1.56*	0.87	1.03	0.79
Proteasome subunit beta type-7	PSMB7	1.82*	1.76*	1.69*	1.55*	0.92	1.00	0.86
Proteasome subunit beta type-8	PSMB8	0.50*	0.69*	0.76*	0.70*	0.81	1.41*	0.96
Proteasome subunit beta type-9	PSMB9	0.49*	0.52*	0.57*	0.56*	0.87	1.07	0.93
Proteasome subunit beta type-10	PSMB10	0.29*	0.30*	0.38*	0.35*	0.83	1.31*	0.91
Proteasome activator complex subunit 1	PSME1	0.48*	0.47*	0.61*	0.56*	0.91	1.07	0.94
Proteasome activator complex subunit 2	PSME2	0.41*	0.44*	0.48*	0.42*	0.95	1.02	0.97

7C. Proteins presented in Figure 6

		Interventions						
		Control				*With LPS & Cytokines*		
Proteins	Genes	Control	AQ	AQ+MES	MES	***AQ***	***AQ+MES***	***MES***
**A. Mineral Uptake**								
Calcium/manganese antiporter SLC30A10	SLC30A10	0.74	2.36*	1.62	0.90	1.64*	1.65*	0.61*
High affinity copper uptake protein 1	SLC31A1	0.86	1.16	1.29	1.00	0.95	1.36	0.99
Zinc transporter ZIP4	SLC39A4	1.18	1.56*	1.36	1.15	1.10	1.36	0.78
Zinc transporter ZIP5	SLC39A5	0.81	1.19	0.55*	0.27*	1.26	0.49*	0.35*
Zinc transporter ZIP6	SLC39A6	0.63*	0.93	0.78	0.71*	1.05	0.99	1.14
Zinc transporter ZIP9	SLC39A9	0.40*	0.52*	0.56*	0.53*	1.84*	1.94*	1.50*
Zinc transporter ZIP10	SLC39A10	0.60*	0.72	0.77	0.75	0.61	1.01	0.82
Molybdate-anion transporter	MFSD5	0.82	1.03	0.97	0.92	1.42	1.39	1.24
Magnesium transporter MRS2 homolog, mitochondrial	MRS2	0.57*	0.75	0.77	0.68*	1.09	1.51*	1.20
Magnesium transporter NIPA3	NIPAL1	1.10	1.33	1.50	1.49	1.31	1.50	1.16
Metallothionein-1E	MT1E	0.97	0.70	1.52*	1.22	0.81	1.22	1.39*
Metallothionein-1H	MT1H	1.04	0.63	0.94	0.80	0.44*	0.57	0.65
**B. Iron Metabolism**								
Serotransferrin	TF	0.97	0.89	1.05	1.10	0.97	1.19	1.11
Lactotransferrin	LTF	0.42*	0.41*	0.74*	0.99	0.85	1.16	1.31*
Transferrin receptor protein 1	TFRC	1.58*	1.65*	1.30*	1.06	1.47*	0.91	0.82*

Ceruloplasmin	CP	0.72*	0.45*	0.75	0.79	2.26*	2.86*	3.19*
Natural resistance-associated macrophage protein 2	DMT1	1.20	1.60*	1.48*	1.00	1.18	1.33	0.87
Mitoferrin-2	MFRN2	0.56*	1.03	1.83*	1.80*	0.72	2.23*	1.41
Hephaestin	HEPH	1.06	1.74*	1.57*	1.11	1.59*	1.30*	0.76*
Solute carrier family 40 member 1	FPN	0.33*	0.54*	0.56*	0.59*	1.45*	1.24	1.23
Ferritin heavy chain	FTH1	0.58*	0.57*	0.47*	0.79	0.75*	0.72*	1.15
Ferritin light chain	FTL	0.51*	0.27*	1.39	2.00*	0.30*	2.11*	4.80*

The values indicate the abundance ratio (fold-change) of proteins from organoids (n=4 subjects) compared to the LPS-Cytokines. Abundance ratios from all the treatment groups are also provided for comparison. These proteins are illustrated in Figures 3, 5, and 6 in relation to the control group.
*Results marked with an asterisk indicate significance compared to the LPS-Cytokines as baseline ($p<0.05$).
